# An interpretable Graph-Regularized Optimal Transport Framework for Diagonal Single-Cell Integrative Analysis

**DOI:** 10.1093/gigascience/giag012

**Published:** 2026-02-09

**Authors:** Zexuan Wang, Qipeng Zhan, Shu Yang, Zhuoping Zhou, Mengyuan Kan, Tianhuan Zhai, Li Shen

**Affiliations:** Graduate Group in Applied Mathematics and Computational Science, University of Pennsylvania, 209 S. 33rd Street Philadelphia, PA 19104-6395, USA; Graduate Group in Applied Mathematics and Computational Science, University of Pennsylvania, 209 S. 33rd Street Philadelphia, PA 19104-6395, USA; Department of Biostatistics, Epidemiology and Informatics, Perelman School of Medicine, University of Pennsylvania, Philadelphia, PA 19104, USA; Graduate Group in Applied Mathematics and Computational Science, University of Pennsylvania, 209 S. 33rd Street Philadelphia, PA 19104-6395, USA; Department of Biostatistics, Epidemiology and Informatics, Perelman School of Medicine, University of Pennsylvania, Philadelphia, PA 19104, USA; Department of Biostatistics, Epidemiology and Informatics, Perelman School of Medicine, University of Pennsylvania, Philadelphia, PA 19104, USA; Department of Biostatistics, Epidemiology and Informatics, Perelman School of Medicine, University of Pennsylvania, Philadelphia, PA 19104, USA

**Keywords:** optimal transport, graph Laplacian, single cell, multi-omics, data integration, interpretable

## Abstract

**Background:**

Recent advancements in single-cell omics technologies have enabled detailed characterization of cellular processes. However, coassay sequencing technologies remain limited, resulting in unpaired single-cell omics datasets with differing feature dimensions.

**Finding:**

We present GROTIA (Graph-Regularized Optimal Transport Framework for Diagonal Single-Cell Integrative Analysis), a computational method to align multi-omics datasets without requiring any prior correspondence information. GROTIA achieves global alignment through optimal transport while preserving local relationships via graph regularization. Additionally, our approach provides interpretability by deriving domain-specific feature importance from partial derivatives, highlighting key biological markers. Moreover, the transport plan between modalities can be leveraged for post-integration clustering, enabling a data-driven approach to discover novel cell subpopulations.

**Conclusions:**

We demonstrate GROTIA’s superior performance on four simulated and four real-world datasets, surpassing state-of-the-art unsupervised alignment methods and confirming the biological significance of the top features identified in each domain.

## Graphical Abstract

**Graphical Abstract:**
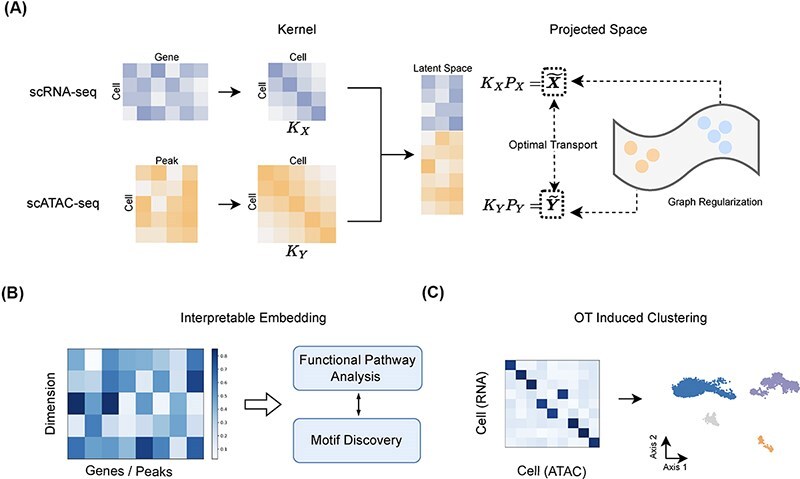

## Introduction

The advancement of single-cell technology offers a comprehensive understanding of cellular heterogeneity and the dynamic evolution of cell states. Various single-cell measurements reveal different aspects: scRNA-seq [1, 2] provides detailed gene expression profiles, while scATAC-seq [3] sheds light on chromatin accessibility in individual cells. Integrating these datasets is crucial as it allows for a more holistic view of cellular mechanisms, enabling the correlation of transcriptional activity with chromatin states to better understand gene regulation and cellular function.

Lots of computational methods have recently been developed to integrate data across multiple modalities [4–9]. A critical challenge for these algorithms is their reliance on correspondence information to identify alignments between paired cells. In practice, such information is often only partially available, hindering the effectiveness of existing strategies [8, 10, 11]. This limitation has led researchers to focus on diagonal integration under semi-supervised settings, where alignment is achieved without direct cell-to-cell correspondences, but cell type labels are still used for hyperparameter tuning. The generalized unsupervised manifold alignment (GUMA) method [12] aligns datasets by optimizing local geometric structures to establish a one-to-one correspondence. Building upon this, the unsupervised topological alignment for single-cell multi-omics integration (UnionCom) algorithm by Cao et al. [7] enables semi-supervised topological alignment, relaxing GUMA’s strict one-to-one mapping requirement. Liu et al. [13] proposed an alternative manifold alignment strategy called MMD-MA, which employs the maximum mean discrepancy (MMD) metric for alignment. Additionally, the single-cell multi-omics alignment with optimal transport (SCOT) method [6] utilizes Gromov–Wasserstein distances to align multi-omic single-cell data. Autoencoder-based approaches have been developed for multimodal data alignment, in which modality-specific autoencoders are trained independently, and their latent representations are subsequently aligned within a common shared latent space [9, 14]. However, even when integration is performed without correspondences, hyperparameters are often tuned using cell label validation, rendering the process semi-supervised. Demetci et al. [6] demonstrated that most methods fail to adapt to fully unsupervised settings when no orthogonal alignment information is available.

Diagonal single-cell multi-omics integration thus faces several key obstacles. First, in the absence of paired samples, one must work under an unpaired assumption, which is common given the practical and financial difficulties of obtaining perfectly matched datasets. Second, integration often relies on shared features (e.g., overlapping genes) that may be missing or poorly represented across modalities. Third, most computational pipelines rely on label-based metrics for hyperparameter tuning, which is problematic in truly unsupervised settings where no external annotations exist. Finally, many existing methods lack an interpretable framework to explain the learned shared embeddings.

Here, we propose GROTIA, a fully unsupervised diagonal integration method that uses optimal transport (OT) and graph regularization to establish alignment without relying on one-to-one correspondences or labeled data. We embed each dataset in a high-dimensional kernel space to capture cell–cell similarities, then learn mappings that transform each dataset into a shared lower-dimensional space for direct comparison. Our framework preserves local geometry via graph Laplacian regularization while performing global alignment through OT, thereby avoiding the need for label-based hyperparameter tuning. In addition, we provide gradient-based sensitivity analyses to highlight key biological genes and peaks that drive the alignment, clear interpretability of the learned latent representation.

**Figure 1:**
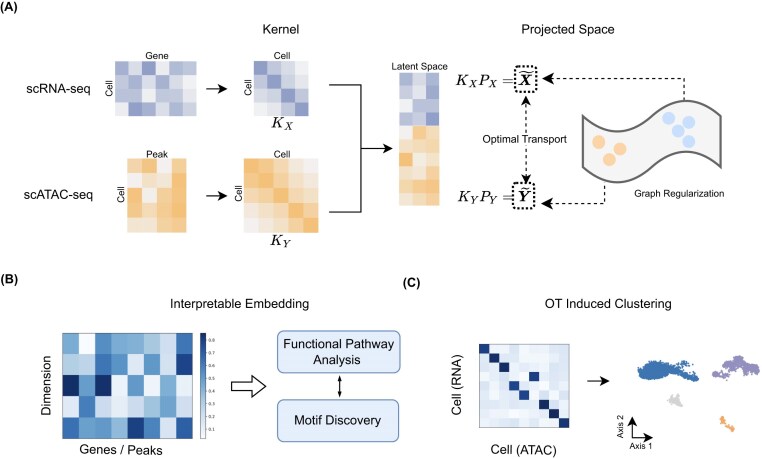
Overview of the GROTIA framework for multi-omics single-cell integration. (A) Schematic design: for each single-cell modality (e.g., scRNA-seq, scATAC-seq), GROTIA constructs a kernel matrix capturing pairwise cell similarities (e.g., ${K}_X$ and ${K}_Y$). It then learns mapping matrices $P_X$ and $P_Y$ to project cells from each modality’s RKHS into a shared latent space, where distributions are aligned via optimal transport. A graph regularization term preserves local neighborhood structure, ensuring that cells close in the original domain remain similarly positioned after integration. (B) Interpretable embedding: once the shared embedding is obtained, GROTIA provides dimensionwise importance scores for genes or peaks. These scores can be used for downstream analyses such as GO or motif discovery, and provide biological interpretation of each latent dimension. (C) OT-induced co-clustering: GROTIA leverages the cross-modality transport plan, which quantifies how strongly each scRNA cell aligns with each scATAC cell. By simultaneously grouping cells from both modalities according to these alignment strengths, GROTIA identifies co-clusters of subpopulations with closely matched regulatory states in the latent space.

We extensively evaluate our model against SCOT, MMD-MA, UnionCom, UniPort, and scConfluence across four simulated and four real-world datasets in unsupervised and semi-supervised settings. Our Graph-Regularized Optimal Transport (GROTIA) algorithm matches the performance of state-of-the-art methods. The schematic design of our approach is illustrated in Fig. 1.

## Methods

### Simulated datasets

We evaluated the GROTIA algorithm using four simulated datasets: three from Liu et al. [13], specifically designed to test alignment methods with different geometric structures, and one additional dataset from Demetci et al. [6], simulating single-cell RNA sequencing count data via Splatter [15]. Specifically, the first dataset presents a branch structure in two-dimensional space, the second a Swiss roll in three-dimensional space, and the third a circular frustum also in three-dimensional space. Although these datasets originally feature complex topological and geometric structures in low-dimensional spaces, they have been nonlinearly projected into high-dimensional feature spaces of 1,000 and 2,000 dimensions for evaluating alignment methods. The fourth is a synthetic RNA-seq dataset from Demetci et al. [6], consisting of 5,000 cells with either 50 or 500 features. Following the approaches in the original publications, we applied *Z*-score normalization to all features before running alignment algorithms.

### Real-world datasets

We then evaluated the GROTIA algorithm on four real-world datasets, widely recognized as gold-standard benchmarks in multi-omics integration and commonly used for assessing state-of-the-art methods. These include (1) scGEM, which simultaneously profiles gene expression and DNA methylation [16]; (2) a dataset generated by the SNARE-seq assay, linking chromatin accessibility with gene expression [17]; (3) a human peripheral blood mononuclear cell (PBMC) dataset (PBMC 10X) consisting of 9,378 cells per modality [9]; and (4) an additional PBMC 10X dataset containing 11,259 cells per modality [14]. We chose these paired multi-omics datasets specifically to enable diagonal integration with known ground-truth cell correspondences. Importantly, during benchmarking, all methods are provided with unpaired data, and the known cell-pairing information was used only for evaluating alignment accuracy.

The first real-world dataset, named “scGEM,” measures gene expression and DNA methylation in the same cells and was generated using the scGEM assay. It contains human somatic cells reprogrammed to a pluripotent state, forming a continuous developmental trajectory. Cao et al. [7] and Demetci et al. [6] previously employed this dataset to evaluate integration methods. Specifically, it has 177 cells with 34 gene-expression features and 177 cells with 27 DNA methylation features. We used the preprocessed version from Demetci et al. [6].

The second real-world dataset, SNARE-seq, jointly profiles chromatin accessibility and gene expression. The dataset was preprocessed using cisTopic [18], resulting in an ATAC-seq matrix of 1,047 cells by 19 features and an RNA-seq matrix of 1,047 cells by 10 features. Following standard practice, unit normalization was then applied to these matrices. We used this preprocessed SNARE-seq dataset from Demetci et al. [6].

Additionally, we analyzed two multi-omics PBMC datasets from publicly available 10X Genomics sources. The first, preprocessed by UniPort [9], contains 11,259 cells with 28,307 scATAC-seq features and 11,942 scRNA-seq genes. The second, preprocessed by scConfluence [14], includes 9,378 cells with 130,417 scATAC-seq features and 15,417 scRNA-seq genes. We used both PBMC datasets as provided for our integrative analyses.

In addition to the primary datasets described above, we evaluated GROTIA on an additional dataset with unbalanced cell populations to assess robustness, with full details and results provided in the *Supplementary Appendix: Additional Dataset*.

### Problem formulation

We introduce a method to integrate single-cell datasets across different conditions or modalities. Let us consider two datasets, *X* and *Y*, with respective representations $X=$  $\left\lbrace x_1, \ldots , x_{n_x}\right\rbrace \subset \mathscr {X}$ and $Y=\left\lbrace y_1, \ldots , y_{n_y}\right\rbrace \subset \mathscr {Y}$, where $n_x$ and $n_y$ denote the number cells in *X* and *Y*, respectively. We aim to uncover a shared manifold structure between *X* and *Y* without a priori correspondence between the datasets.

To achieve this, we first compute the intra-dataset kernels $K_X$ and $K_Y$, which capture the internal structure of *X* and *Y*, respectively. As long as it is positive definite, each kernel corresponds to an implicit feature mapping $\phi _X$ : $\mathscr {X} \rightarrow \mathscr {H}_X$ and $\phi _Y: \mathscr {Y} \rightarrow \mathscr {H}_Y$, where $\mathscr {H}_X$ and $\mathscr {K}_Y$ are the reproducing kernel Hilbert spaces (RKHS) associated with $K_X$ and $K_Y$. Subsequently, we seek mapping functions $f_X: \mathscr {X}\rightarrow \mathbb {R}^k$ and $f_Y: \mathscr {Y}\rightarrow \mathbb {R}^k$, where *k* is the dimensionality of the shared space. These functions are optimized so that the mapped representations $f_X\left(X\right)$ and $f_Y\left(Y\right)$ are well aligned, thereby discovering the shared manifold structure.

### Kernel representation

To capture the intrinsic geometry of the datasets, we define the intra-dataset kernels $K_X$ and $K_Y$ using Gaussian kernel functions:


(1) \begin{eqnarray*}
K_X(x_i, x_j) &=& \exp \left( -\frac{\Vert x_i - x_j\Vert ^{2}}{2\sigma _X^{2}} \right),\\
K_Y(y_i, y_j) &=& \exp \left( -\frac{\Vert y_i - y_j\Vert ^{2}}{2\sigma _Y^{2}} \right),
\end{eqnarray*}

where $\sigma _X$ and $\sigma _Y$ are bandwidth parameters specific to *X* and *Y*, respectively. These kernels define the feature maps $\phi _X: \mathscr {X} \rightarrow \mathscr {H}_X$ and $\phi _Y: \mathscr {Y} \rightarrow \mathscr {H}_Y$ into their respective RKHS.

We adopt a data-driven approach to determine $\sigma _X$ and $\sigma _Y$ by setting each parameter to the mean of the pairwise Euclidean distances within the corresponding dataset. This heuristic adjusts the bandwidths to reflect the average spatial dispersion of the data points, thereby tuning the kernels to the specific scale of variability in each dataset.

### Optimal transport

For simplicity, we will use the notation: $\widetilde{X}=f_X\left(X\right) \in \mathbb {R}^{n_x \times k}, \widetilde{Y}=f_Y\left(Y\right) \in \mathbb {R}^{n_y \times k}$ to represent the mapped datasets. The Sinkhorn divergence between the projected representations $\widetilde{X}$ and $\widetilde{Y}$ is defined as


(2) \begin{eqnarray*}
\mathcal {L}_{\mathrm{OT}}(\widetilde{X}, \widetilde{Y})=\mathrm{OT}_{\varepsilon }(\widetilde{X}, \widetilde{Y})-\frac{1}{2}\left(\mathrm{OT}_{\varepsilon }(\widetilde{X}, \widetilde{X})+\mathrm{OT}_{\varepsilon }(\widetilde{Y}, \widetilde{Y})\right),
\end{eqnarray*}

where $\mathrm{OT}_{\varepsilon }(\cdot , \cdot )$ denotes the entropically regularized OT cost between two distributions. Next, we define the entropic OT cost between $\widetilde{X}$ and $\widetilde{Y}$. The cost is computed as


(3) \begin{eqnarray*}
\mathrm{OT}_{\varepsilon }(\widetilde{X}, \widetilde{Y})=\min _{T \in \Pi (\mathrm{a}, \mathrm{ b})}\langle C, T\rangle +\varepsilon H(T),
\end{eqnarray*}

where $\Pi (\mathbf {a}, \mathbf {b})=\left\lbrace T \in \mathbb {R}_{+}^{n_x \times n_y} \mid T \mathbf {1}_{n_y}=\mathbf {a}, T^{\top } \mathbf {1}_{n_x}=\mathbf {b}\right\rbrace$. The matrix $T \in \mathbb {R}_{+}^{n_x \times n_y}$ is the transport plan matrix, representing the amount of mass transported from $\widetilde{x}_i$ to $\tilde{y}_j$. The cost matrix $C \in \mathbb {R}^{n_x \times n_y}$ quantifies the pairwise distances between the projected samples. Each element is defined as $C_{\mathrm{ij}}=\left\Vert \widetilde{x}_i-\tilde{y}_j\right\Vert _2^2$. The parameter $\varepsilon \,\gt\,0$ is the entropic regularization parameter that smooths the optimization problem, and $H(T)=-\sum _{i=1}^{n_x} \sum _{j=1}^{n_y} T_{\mathrm{ij}}\left(\log T_{\mathrm{ij}}-1\right)$ is the entropy of the transport plan *T*. The marginal distributions $\mathbf {a} \in \mathbb {R}^{n_x}$ and $\mathbf {b} \in \mathbb {R}^{n_y}$ are typically uniform distributions over the samples: $\mathbf {a}=\frac{1}{n_x} \mathbf {1}_{n_x}, \quad \mathbf {b}=\frac{1}{n_y} \mathbf {1}_{n_y}$, where $\mathbf {1}_{n_x}$ and $\mathbf {1}_{n_y}$ are vectors of ones with lengths $n_x$ and $n_y$, respectively. We utilize OT over MMD due to its benefits, such as non-vanishing gradients and other theoretical advantages [19].

### Graph Laplacian regularization

To capture the local geometric structures of the datasets *X* and *Y*, we construct graph Laplacians based on the *k*-nearest neighbor relationships defined through the Gaussian kernels. These Laplacians serve as regularizers for the mapping functions, enforcing smoothness by ensuring that nearby data points in the RKHS spaces $\mathscr {H}_X$ and $\mathscr {H}_Y$ remain close in the shared latent space.

For each dataset, we begin by constructing a *k*-nearest neighbor graph in the RKHS. Specifically, for dataset *X*, we identify the set of *k*-nearest neighbors for each feature-mapped data point $\phi _X\left(x_i\right)$, denoted as $\mathcal {N}_k\left(\phi _X\left(x_i\right)\right)$, based on the distance metric in $\mathscr {H}_X$. The adjacency matrix $W_X \in \mathbb {R}^{n_x \times n_x}$ is then defined with entries:


(4) \begin{eqnarray*}
[W_X]_{ij} = \left\lbrace \begin{array}{l{@}{\quad}l}
1, & \text{if } \, \phi _X(x_j) \in \mathcal {N}_k (\phi _X(x_i)) \\
& \text{or } \, \phi _X(x_i) \in \mathcal {N}_k (\phi _X(x_j) )\\
0, & \mathrm{otherwise}. \end{array}\right.
\end{eqnarray*}

Similarly, for dataset *Y*, we construct the adjacency matrix $W_Y \in \mathbb {R}^{n_y \times n_y}$. Next, we compute the degree matrices $D_X$ and $D_Y$, which are diagonal matrices where each diagonal entry represents the sum of the edge weights connected to a node: $\left[D_X\right]_{i i}=\sum _{j=1}^{n_x}\left[W_X\right]_{\mathrm{ij}},\left[D_Y\right]_{i i}=\sum _{j=1}^{n_y}\left[W_Y\right]_{\mathrm{ij}}$. The graph Laplacians are then defined as the difference between the degree and adjacency matrices:


(5) \begin{eqnarray*}
L_X=D_X-W_X, \quad L_Y=D_Y-W_Y.
\end{eqnarray*}

To regularize the projected representations $\widetilde{X}$ and $\widetilde{Y}$, we introduce smoothness terms based on the Laplacian quadratic form. Specifically, the smoothness term for $\widetilde{X}$ is given by


(6) \begin{eqnarray*}
\frac{1}{2} \sum _{i=1}^{n_x} \sum _{j=1}^{n_x}{[W_X]}_{\mathrm{ij}}\left\Vert \tilde{x}_i-\tilde{x}_j\right\Vert ^2=\mathrm{Tr}\left(\tilde{X}^{\top } L_X \tilde{X}\right),
\end{eqnarray*}

where $\widetilde{x}_i$ denotes the *i*th row of $\widetilde{X}$. This expression encourages neighboring points in the original data space to have similar representations in the latent space, promoting smoothness in the mappings.

### GROTIA algorithm

To integrate the datasets *X* and *Y* into a shared latent space, we propose the GROTIA algorithm. Our objective is to find the mapping $f_X: \mathscr {X} \rightarrow \mathbb {R}^k, f_Y:\mathscr {Y} \rightarrow \mathbb {R}^k$ that maps the data into a common *k*-dimensional space, effectively aligning their underlying manifold structures. The existence of such mapping is guaranteed by the representer theorem, which states that the optimal mappings can be expressed as finite linear combinations of the kernel functions:


(7) \begin{eqnarray*}
\left[f_X\right]_j\left(x\right)=\sum _{i=1}^{n_x} \alpha _X^{{ij}} K_X\left(x_i, x\right),
\end{eqnarray*}

where $\alpha _X^{{ij}}$ are the learned coefficients, $K_X$ is the kernel function, and $x_i$ are the samples from *X*. The coefficients $\alpha _X^{{ij}}$ are then organized into the matrix $P_X \in \mathbb {R}^{n_x \times k}$, and similarity for *Y* we have $P_Y \in \mathbb {R}^{n_y \times k}$. Thus, the final mapped representations are given by


(8) \begin{eqnarray*}
\widetilde{{X}}=K_X P_X, \quad \widetilde{{Y}}=K_Y P_Y.
\end{eqnarray*}

The optimization problem for the GROTIA algorithm is formulated as


(9) \begin{eqnarray*}
\min _{P_X,P_Y}\,\, \mathcal {L}_{\mathrm{OT}}(\widetilde{X},\widetilde{Y}) &+& \lambda _{\mathrm{topo}}\,\, \bigl [ \mathrm{Tr}(\widetilde{X}^{\!\top } L_X \widetilde{X}) + \mathrm{Tr}(\widetilde{Y}^{\!\top } L_Y \widetilde{Y}) \bigr ] \\
&+& \lambda _{\mathrm{ortho}}\,\, \bigl [ \Vert P_X^{\!\top } K_X P_X - I_k\Vert _F^{2} + \Vert P_Y^{\!\top } K_Y P_Y - I_k\Vert _F^{2} \bigr ].\\
\end{eqnarray*}

The first term $\mathcal {L}_{\mathrm{OT}}(\widetilde{X}, \widetilde{Y})$ aligns the global distributions of the datasets in the latent space using Sinkhorn divergence. Minimizing the Sinkhorn divergence between the latent space representations ensures that the overall structures of *X* and *Y* are closely matched after projection. This captures global structural similarities and facilitates the discovery of shared manifold features between the datasets.

The second term $\mathrm{Tr}\left(\widetilde{X}^{\top } L_X \widetilde{X}\right)$ and $\mathrm{Tr}\left(\widetilde{Y}^{\top } L_Y \widetilde{Y}\right)$ is used preserve the local geometric structures inherent in each dataset. These terms penalize the weighted differences between neighboring points in the latent space. Doing so encourages neighboring points in the $\mathscr {H}_X,\mathscr {H}_Y$ to remain close in the latent space.

To prevent degenerate solutions and ensure that the mappings retain meaningful structure, we impose orthogonality constraints on the mapping matrices through the terms $\left(\Vert P_X^\top K_X P_X - I_k\Vert _F^2 \right)$ and $\left(\Vert P_Y^\top K_Y P_Y - I_k\Vert _F^2\right)$. For details on the effect of this constraint, we refer the reader to the *Supplementary Appendix: Effect of the RKHS Orthogonality Constraint*.

By jointly optimizing this objective function, the GROTIA algorithm effectively balances global alignment and local structure preservation while ensuring that the projections are meaningful and well behaved.

### Interpretable embeddings of GROTIA

In our approach, each domain (scRNA or scATAC) is mapped into a shared, low-dimensional space using kernel-based transformations. Take the scRNA space, for example, let $X \in \mathbb {R}^{n_{x} \times d_x}$ denote the data matrix for one domain, where $n_{x}$ is the number of cells and $d_x$ is the number of features. We construct a radial basis function (RBF) kernel $K \in \mathbb {R}^{n_{x} \times n_{x}}$, with elements $K_{i, j}=\exp (-\gamma _{X}\Vert X_{i, \cdot }-X_{j,}\Vert ^2)$, where $\gamma _{X}$ is bandwidth parameter. Through our optimization procedure, we learn a coefficients matrix $\alpha _X^{\mathrm{ij}}$ such that the *k*-dimensional embedding for the *j*th cell is given by $\left[f_X\right]_j(x)=\sum _{i=1}^{n_x} \alpha _X^{\mathrm{ij}} K_X\left(x_i, x\right)$. Here, $\left[f_X\right]_j(x)$ represents the coordinate of cell *j* in the *k* dimensional learned embedding. An analogous formulation with $\beta$ is employed for the scATAC domain (using its own kernel matrix).

To identify which original features (e.g., genes in scRNA, peaks in scATAC) have the greatest influence on each embedding dimension, we compute partial derivatives of $f_d\left(x_j\right)$ with respect to each feature. Concretely, let $x_{j, g}$ denote the value of feature *g* in cell *j*. Then, for an RBF kernel


(10) \begin{eqnarray*}
\frac{\partial }{\partial x_{j, g}} K_{i, j}=-2 \gamma (x_{j, g}-x_{i, g}) \exp (-\gamma \Vert x_j-x_i\Vert ^2) .
\end{eqnarray*}

Using the chain rule, the partial derivative of the *d*th embedding coordinate with respect to $x_{j, g}$ becomes


(11) \begin{eqnarray*}
\frac{\partial f_d}{\partial x_{j,g}}\bigl (x_j\bigr ) &=& \sum _{i=1}^{N} \alpha _{i,d}\, \frac{\partial K_{i,j}}{\partial x_{j,g}} \\
&=& -2\gamma \sum _{i=1}^{N} \alpha _{i,d}\, (x_{j,g}-x_{i,g})\, K_{i,j}.
\end{eqnarray*}

Thus, a large magnitude of $\left|\frac{\partial f_d}{\partial x_{j, g}}\left(x_j\right)\right|$ indicates that small perturbations in feature *g* for cell *j* induce substantial shifts in the *d*th embedding coordinate. To obtain a global feature-importance measure, we average these derivatives across all cells:


(12) \begin{eqnarray*}
I_{d, g}=\frac{1}{N} \sum _{j=1}^N\left|\frac{\partial f_d}{\partial x_{j, s}}\left(x_j\right)\right|.
\end{eqnarray*}

Features *g* with higher $I_{d, g}$ are deemed more influential in shaping dimension *d*. An identical procedure is applied in the scATAC domain using the learned projection $\beta$ and its kernel $K_Y$.

To identify key molecular drivers in each latent dimension, we first ranked genes by their contribution scores $I_{d, g}$. The highest-ranked genes displayed significant variation in expression closely linked to the biological structure observed within the low-dimensional embedding. Gene ontology (GO) enrichment analyses conducted on these top-ranking genes using g:Profiler [20] with default parameters revealed strong enrichment for cellular metabolism-related processes.

In parallel, we investigated regulatory elements in the chromatin accessibility (ATAC) domain. Similar to the gene-ranking procedure, open chromatin peaks were ordered by their respective contribution scores for each GROTIA-derived dimension. We then identified transcription factor binding sites (TFBSs) by performing de novo motif discovery on the most influential ATAC-seq peaks associated with each latent dimension using GimmeMotifs [21], applying a false discovery rate threshold of <0.001. This analysis yields enriched DNA sequence motifs representing putative TF binding sites in accessible chromatin and, through motif annotation, provides a set of candidate transcription factors (TFs) associated with each motif. Specifically, each de novo TFBS was annotated by matching its position weight matrix to reference motif databases (including JASPAR, HOCOMOCO, CIS-BP, and ENCODE) using Pearson correlation-based similarity scores, thereby assigning candidate TFs to each TFBS based on similarity to known binding preferences. We next linked individual TFBS-containing peaks to proximal genes by assigning each site to the nearest gene within a ±20 kb window [22, 23]. Finally, these candidate target genes were filtered by intersecting them with the top RNA-expressed genes associated with the same latent dimension, yielding dimension-specific TF–gene regulatory pairs.

### Co-cluster using OT plan

GROTIA also enables post-integration analysis to identify data-driven clusters. Specifically, an OT plan is first computed to quantify the flow between two distinct sets of entities (e.g., cells in the RNA and ATAC spaces). We then apply a co-clustering algorithm [24] based on alternating maximization of bipartite graph modularity directly to the resulting transport matrix, thereby simultaneously grouping row and column entities. By treating the OT plan as a bipartite graph, this co-clustering approach identifies latent structural patterns that minimize within-cluster transport costs while maximizing separation between clusters. Following co-clustering, clusters containing fewer than 20 cells are pruned as noise.

### Evaluated metrics

Each alignment method was assessed in two distinct evaluation modes, each paired with two quantitative metrics. Unsupervised mode tuned its hyperparameters solely by minimizing the model’s objective function, deliberately withholding any label information. Semi-supervised mode, in contrast, selected hyperparameters that maximized downstream cell-type classification accuracy on a held-out validation set; crucially, these labels were used only during the tuning phase and were never provided to the model as inputs, preserving the semi-supervised setup.

The first metric is Fraction of Samples Closer Than the True Match (FOSCTTM). For each sample $x_i$ in domain *X*, let the corresponding (true matched) sample in domain *Y* be $y_i^{*}$. We first embed both *X* and *Y* into a common space via embedding functions $f_1$ and $f_2$, respectively. We then define the distance between embedded points using a distance measure $d(\cdot , \cdot )$. The FOSCTTM metric for each sample $x_i$ measures the fraction of samples in *Y* that are closer to $x_i$ (in the embedded space) than its true match $y_i^{*}$. Formally,


(13) \begin{eqnarray*}
R_i = \frac{1}{|Y|-1} \sum_{\substack{j \in Y \\
j \ne i}} 1\left\lbrace d(f_1(x_i), f_2(y_j))\,\lt\, d\left(f_1(x_i), f_2\left(y_i^{*}\right)\right)\right\rbrace,
\end{eqnarray*}

where $\mathbf {1}\lbrace \cdot \rbrace$ is the indicator function, returning 1 if the condition is satisfied and 0 otherwise. The FOSCTTM score for the entire dataset is the average of $R_i$ across all $x_i \in X$ :


(14) \begin{eqnarray*}
\mathrm{FOSCTTM}=\frac{1}{|X|} \sum _{i=1}^{|X|} R_i.
\end{eqnarray*}

A lower FOSCTTM value indicates better alignment, as it means fewer samples in *Y* are closer to $x_i$ than the true match $y_i^{*}$.

The second metric is Label transfer accuracy (LTA) and it evaluates how well cell-type (or other categorical) labels can be transferred from domain *X* to domain *Y* in the integrated space [6]. After embedding both datasets into a shared representation, each point $x_i \in X$ has a known label $L_X\left(x_i\right)$. We define $\text{ kNN}\left(x_i\right)$ as the set of the *k* nearest neighbors of $x_i$ in the embedded representation of *Y*. Let


(15) \begin{eqnarray*}
\hat{L}\left(x_i\right)=\text{ mode}\left\lbrace L_Y(y): y \in \text{ kNN}\left(x_i\right)\right\rbrace,
\end{eqnarray*}

where mode $(\cdot )$ returns the most common label among those *k* neighbors. The LTA score is then computed as


(16) \begin{eqnarray*}
\mathrm{LTA}=\frac{1}{|X|} \sum _{i=1}^{|X|} \mathbf {1}\left\lbrace \hat{L}\left(x_i\right)=L_X\left(x_i\right)\right\rbrace,
\end{eqnarray*}

where $\mathbf {1}(\cdot )$ is the indicator function. A higher LTA indicates that the integrated embedding preserves biological labels more accurately between the two domains.

### Training details

We implemented GROTIA in PyTorch using the Adam optimizer. Whenever the training loss plateaued, the learning rate was reduced by a factor of 0.5. We selected the latent dimension to be either 5 or 8 and observed that GROTIA remained robust to this choice. To preserve local structure via the graph Laplacian, we set the number of nearest neighbors to 5. Three hyperparameters appear in the loss function: $\lambda _{\mathrm{topo}}$, which emphasizes preservation of local geometry within each modality, and $\lambda _{\mathrm{ortho}}$, which controls the strength of the orthogonality constraint. We searched over a grid of $\lambda _{\mathrm{ortho}} \in \lbrace 1, 10^{-1}, 10^{-2}, 10^{-3}\rbrace$ and $\lambda _{\mathrm{topo}} \in \lbrace 10^{-3}, 10^{-4}, 10^{-5}, 10^{-6}, 10^{-7}, 10^{-8}\rbrace$, with the constraint $\lambda _{\mathrm{ortho}} \,\gt\, \lambda _{\mathrm{topo}}$ to ensure that local structure is preserved while the mapping remains close to a projection. Additionally, we varied the reach parameter over $\lbrace 0.1, 1.0, 5.0\rbrace$, where reach controls the degree of unbalancedness in the Sinkhorn loss. A more detailed explanation is provided in the *Supplementary Appendix: Optimization Details*. All experiments were run on a single NVIDIA A100 GPU.

The input to GROTIA follows standard dataset preprocessing practices and does not require method-specific processing. For all simulated datasets, features were *z*-score normalized prior to alignment, following the procedure used in Liu et al. [13]. For the scGEM and SNARE-seq datasets, we downloaded the preprocessed datasets provided by Demetci et al. [6] and applied the same unit-normalization procedure described in that work. For the PBMC-1 and PBMC-2 datasets, we strictly followed the preprocessing pipelines used by the original authors of the corresponding benchmarked methods [9, 14]. Specifically, we downloaded the raw data and applied the preprocessing scripts provided by the respective authors. Consequently, all datasets were used exactly as released by the original benchmarked integration methods to ensure fair and consistent comparison, and no customized or alternative preprocessing pipelines were introduced in this work.

### Baseline settings

To benchmark our method (GROTIA), we compared it against several existing approaches, each downloaded and configured according to the authors’ guidelines. SCOT (v1.0) was obtained from Demetci et al. [6]. We provided the same PCA-preprocessed input to SCOT as to GROTIA, mirroring SCOT’s original publication. We then tuned the hyperparameter based on the recommendations in the SCOT documentation.

We downloaded UnionCom (v0.4.0) and applied the same input as in GROTIA, again following the developers’ suggested preprocessing steps. All hyperparameters were tuned according to the guidance provided in the UnionCom package.

For UniPort (v1.3), which supports diagonal integration (i.e., mode=d) to align datasets without common genes, we used 2,000 highly variable genes from the scRNA-seq data and peaks exceeding a threshold of 1 for the scATAC-seq data. We also employed TF-IDF normalization, replicating the tutorial steps outlined by the UniPort authors. We obtained the MMD-MA (v1.0) PyTorch implementation from Singh et al. [25]. As with GROTIA and SCOT, we provided PCA-preprocessed data and tuned its hyperparameters in accordance with the authors’ guidelines.

Lastly, we downloaded scConfluence (v0.1.1). For the version without prior information, we set $\lambda _{I O T}=0$, forcing a diagonal integration approach. When using scConfluence with prior information, we followed the recommended settings from the authors. We also tuned the remaining hyperparameters according to their instructions, applying identical preprocessing to ensure fair comparisons across all methods. For the four simulated datasets as well as the scGEM and SNARE-seq datasets, the construction of a cross-modality distance matrix is non-trivial. Specifically, the simulated datasets lack meaningful cross-modal feature definitions, and computing such distances for scGEM and SNARE-seq is not feasible.

### Use of large-language models

We used OpenAI ChatGPT only for grammar, spelling, and stylistic refinement of the manuscript. The model did not generate or alter any scientific content, data analysis, interpretations, or conclusions. All AI suggestions were manually reviewed and either accepted or rejected by the authors.

## Results

### GROTIA integrated simulated datasets in both semi and unsupervised settings

We evaluated the GROTIA algorithm using four simulated datasets previously discussed. Performance of the GROTIA algorithm was compared against five benchmarked methods: SCOT, UnionCom, MMD-MA, and two VAE-based approaches, scConfluence and UniPort. Evaluations were conducted under two scenarios: semi-supervised (partial label information available) and unsupervised (no label information available).

**Figure 2:**
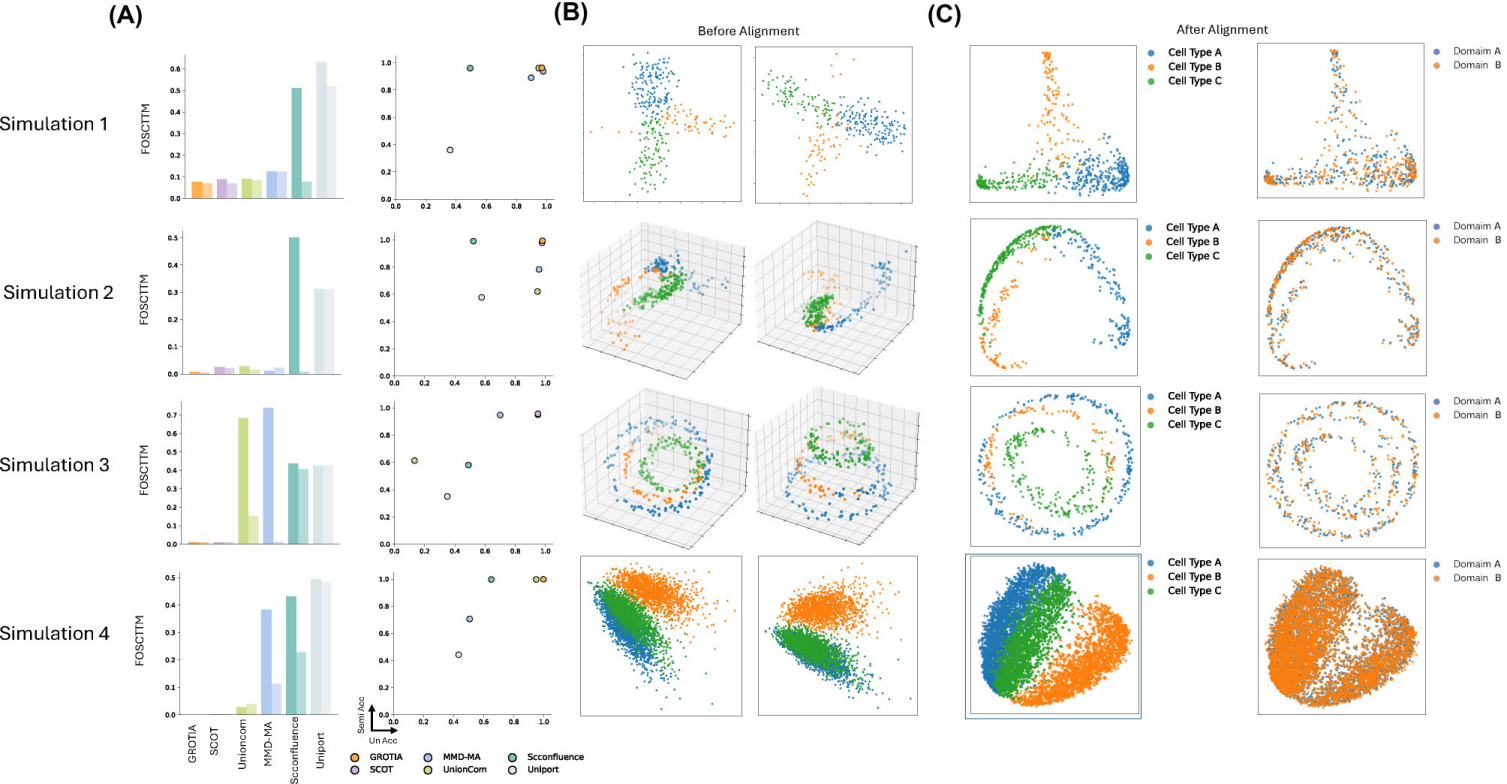
Benchmarking results on simulated datasets. (A) Evaluation of LTA and FOSCTTM across five benchmarked methods under two evaluation modes: semi-supervised and unsupervised. For each method, two bars are shown in the same plot, with the left bar representing the semi-supervised mode and the right bar representing the unsupervised mode for FOSCTTM. LTA is shown with semi-supervised results on the *x*-axis (Semi Acc) and unsupervised results on the *y*-axis (Un Acc). (B) Visualization of simulated datasets before integration. Simulations 1 and 4 are visualized using the first two PCA components, whereas Simulations 2 and 3 are visualized using the first three axes from multidimensional scaling. (C) Visualization after integration under unsupervised alignment. The first column displays data colored by cell type, while the second column shows data colored by domain. All four datasets are visualized using the first two PCA components.

In Fig. 2A, the left column (solid color) and right column (vertical axis) display FOSCTTM and LTA, respectively, under the semi-supervised setting. In the semi-supervised scenario, GROTIA and SCOT demonstrated consistently high performance across all datasets, with UnionCom and MMD-MA closely following. scConfluence and UniPort showed comparatively lower performance, possibly due to their reliance on cross-modality guidance, which was not available in these simulations.

In Fig. 2A, the left column (light color) and right column (horizontal axis) display FOSCTTM and LTA, respectively, under the unsupervised setting. In the unsupervised scenario, where alignment depends solely on intrinsic structural information, GROTIA and SCOT maintained relatively stable performance. Other methods showed varying degrees of accuracy reduction, notably UnionCom on dataset 3, MMD-MA on datasets 3 and 4, scConfluence on datasets 1, 2, and 4, and UniPort on dataset 1. These variations highlight the challenges inherent in unsupervised alignment without label guidance.

In Fig. 2B, the datasets from each domain are shown prior to integration. The left column corresponds to one modality and the right column to the other modality. Figure 2C shows the integrated datasets, colored by cell type on the left and by domain on the right. These results demonstrate that GROTIA effectively aligns the two modalities, with cells of the same type from different modalities well matched and the two domains properly overlapping in the integrated space.

The stable performance of GROTIA can be attributed to their effective use of Wasserstein-based (WD) losses, capturing intrinsic geometric structures. Additionally, GROTIA employs orthogonality constraints within the RKHS, enhancing embedding stability. Although VAE-based methods also utilize WD losses, their embeddings can be susceptible to rotational variations, affecting alignment stability in the absence of label information. Detailed numerical results corresponding to the metric plots are presented in Supplementary Tables A2–A5.

### GROTIA integrated real-word datasets in both semi and unsupervised settings

We then evaluated the GROTIA algorithm on four real-world datasets. We chose these paired multi-omics datasets specifically to enable diagonal integration with known ground-truth cell correspondences. Importantly, during benchmarking, all methods are provided with unpaired data, and the known cell-pairing information was used only for evaluating alignment accuracy.

The benchmarking process used here follows the same procedure described in the simulation study, with one modification: for the PBMC-1 and PBMC-2 datasets, we additionally include scConfluence with prior information. We do not include the prior version of scConfluence for the scGEM and SNARE datasets because the original authors performed dimensionality reduction to process these data, meaning commonly used gene selection methods are not available and the resulting feature space is small.

**Figure 3:**
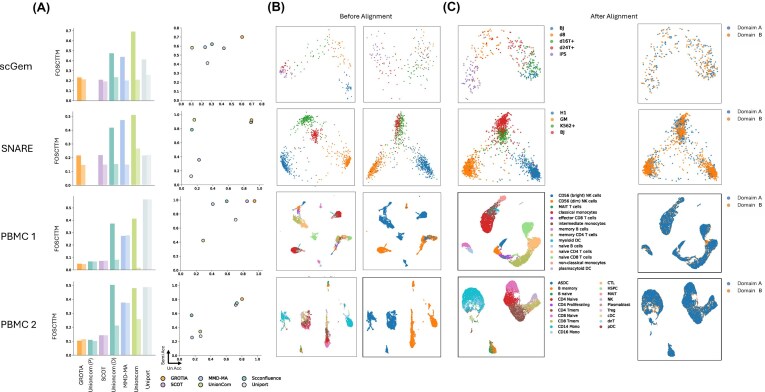
Benchmarking results on real-word datasets. (A) Evaluation of LTA and FOSCTTM across five benchmarked methods under two evaluation modes: semi-supervised and unsupervised. For each method, two bars are shown in the same plot, with the left bar representing the semi-supervised mode and the right bar representing the unsupervised mode for FOSCTTM. LTA is shown with semi-supervised results on the *x*-axis (Semi Acc) and unsupervised results on the *y*-axis (Un Acc). (B) Visualization of real-word datasets before integration. ScGem and SNARE are visualized using the first two PCA components, whereas PBMC 1 and PBMC 2 are visualized using the first two UMAP components. (C) Visualization after integration under unsupervised alignment. The first column displays data colored by cell type, while the second column shows data colored by domain. ScGem and SNARE are visualized using the first two PCA components, whereas PBMC 1 and PBMC 2 are visualized using the first two UMAP components.

In Fig. 3A, the left column (solid color) and right column (vertical axis) display FOSCTTM and LTA, respectively, under the semi-supervised setting. Across the scGEM and SNARE-seq datasets, GROTIA achieves the second-best FOSCTTM in scGEM and the best in SNARE-seq, while attaining the highest LTA in both. SCOT obtains the top FOSCTTM in scGEM but ranks second in the remaining evaluations. scConfluence (default), MMD-MA, UnionCom, and UniPort follow in overall performance. For the two PBMC datasets, GROTIA places second in FOSCTTM for both PBMC-1 and PBMC-2; in PBMC-2, scConfluence with prior information attains the best FOSCTTM. Regarding LTA, GROTIA ranks second in PBMC-1 and first in PBMC-2.

Under the fully unsupervised setting, shown by lighter colors in the left column of Fig. 3A (and the right column’s horizontal axis for LTA), GROTIA demonstrates the strongest overall performance in both FOSCTTM and LTA across all four real-world datasets, except for ranking second in FOSCTTM on scGEM. SCOT and scConfluence exhibit comparable results, followed by UnionCom, MMD-MA, and UniPort. Detailed numerical results corresponding to the metric plots are presented in Supplementary Tables A6–A9.

In Fig. 3B, each modality is visualized separately prior to integration, with the left column showing one domain and the right column showing the other. Figure 3C presents the integrated representation, colored by cell type on the left and by domain on the right. The results indicate that GROTIA successfully aligns the two modalities, bringing cells of the same type from different domains into close correspondence while achieving strong overlap between modalities in the integrated space.

### GROTIA reveals gene-specific contributions and key biological processes in the RNA embedding

GROTIA provides an in-model measure of gene importance, pinpointing which genes drive variation along each latent dimension. The PBMC-1 dataset is used to demonstrate the following experiments. Specifically, we compute partial derivatives of the RBF kernel embeddings with respect to each gene’s expression and then weight these by the projection matrix to obtain a contribution score for every gene–dimension pair. Ranking these scores identifies dimension-specific “signature genes”—those whose expression changes most strongly reposition cells in the low-dimensional space. For additional details, see the “Interpretable embeddings of GROTIA” section.

**Figure 4:**
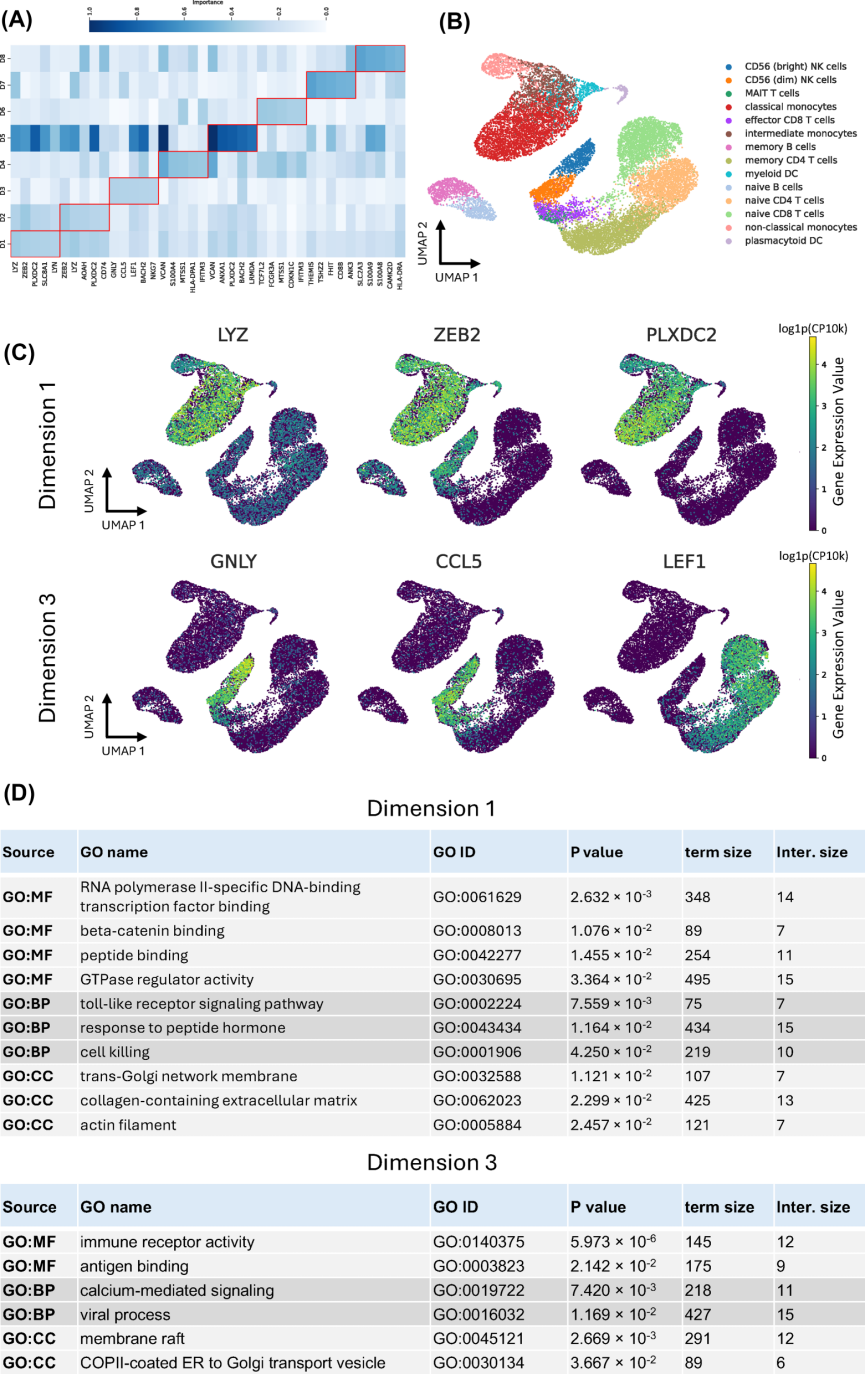
(A) Heatmap of partial derivative-based importance scores for the top five genes in each of the eight GROTIA-derived dimensions (D1–D8). Larger values correspond to higher importance. The top five genes per dimension are outlined in the red box. (B) UMAP projections of Dimension 1 and Dimension 3, highlighting cell-type annotations (top) and the expression distributions of top-ranked genes (middle and bottom). Large values denote higher expression, revealing distinct cellular subsets for Dimension 1 and Dimension 3. (C) Summaries from the GO experiment, showing significant enrichment grouped by molecular function (MF), cellular component (CC), and biological process (BP). Collectively, these panels show how GROTIA’s dimension-wise interpretability links high-impact genes to key biological functions.

Figure 4A displays the overall gene importance scores across all eight GROTIA-derived dimensions. Figure 4B shows UMAP projections of the scRNA-seq data, with gene expression overlaid for genes selected based on their importance in Dimensions 1 and 3 of the learned embedding, illustrating how high-impact genes are distributed across the dataset. For Dimension 1, the top three contributing genes (LYZ, ZEB2, and PLXDC2) are highly expressed in monocytes and myeloid cell populations, consistent with further differentiation within the monocyte lineage [26]. Moreover, Dimension 3 emphasizes GNLY, CCL5, and LEF1—genes enriched in NK cells and T cells, indicating a T cell-specific transcriptional program [26]. Notably, GROTIA requires no a priori matching of features across modalities, so these dimension-specific drivers offer a data-driven method to uncover potential marker genes.

To link the top contributors in Dimension 1 to biological processes, we performed GO enrichment analysis, as shown in Fig. 4C. These genes were enriched for the terms RNA polymerase II-specific DNA-binding TF binding, $\beta$-catenin binding, and peptide binding. The first term suggests that Dimension 1 captures a transcriptional regulatory program active during monocyte and dendritic cell differentiation, involving lineage-defining regulators such as ZEB2, which is essential for monocyte and plasmacytoid DC development [27]. The second highest-ranked term, $\beta$-catenin binding, indicates involvement of Wnt/$\beta$-catenin signaling in monocyte and DC biology; for instance, $\beta$-catenin activation fosters a tolerogenic phenotype in DCs [28], while its aberrant stabilization can obstruct normal monocyte–macrophage differentiation [29]. Finally, enrichment for peptide binding aligns with the antigen processing and presentation roles of monocytes and DCs, consistent with elevated HLA-DR expression in intermediate monocytes [30]. For additional UMAP distributional plots of top genes, see Supplementary Figures A.1 and A.2.

In Dimension 3 of the PBMC transcriptional analysis, we observed GO term enrichment related to immune receptor activity, antigen binding, and calcium-mediated signaling. This suggests that Dimension 3 captures variation in lymphocyte receptor expression and signaling. The GO category immune receptor activity is associated primarily with T lymphocytes, which uniquely express the T-cell receptor complex (e.g., CD3 subunits and TCR $\alpha / \beta$ chains) mediating antigen-specific recognition [31]. Moreover, antigen binding reflects the high expression of immunoglobulin genes by B cells, consistent with their exclusive role in producing antigen-specific antibodies [32]. Finally, calcium-mediated signaling highlights a key activation pathway in T cells, where antigen-receptor engagement triggers Ca^2+^ influx through store-operated Ca^2+^ channels to activate downstream effectors such as the calcineurin–NFAT pathway, a process essential for lymphocyte activation [33]. For additional GO enrichment results corresponding to the remaining dimensions, see Supplementary Tables A10–A15.

### GROTIA identifies high-impact peaks and regulatory mechanisms in the ATAC embedding

**Figure 5:**
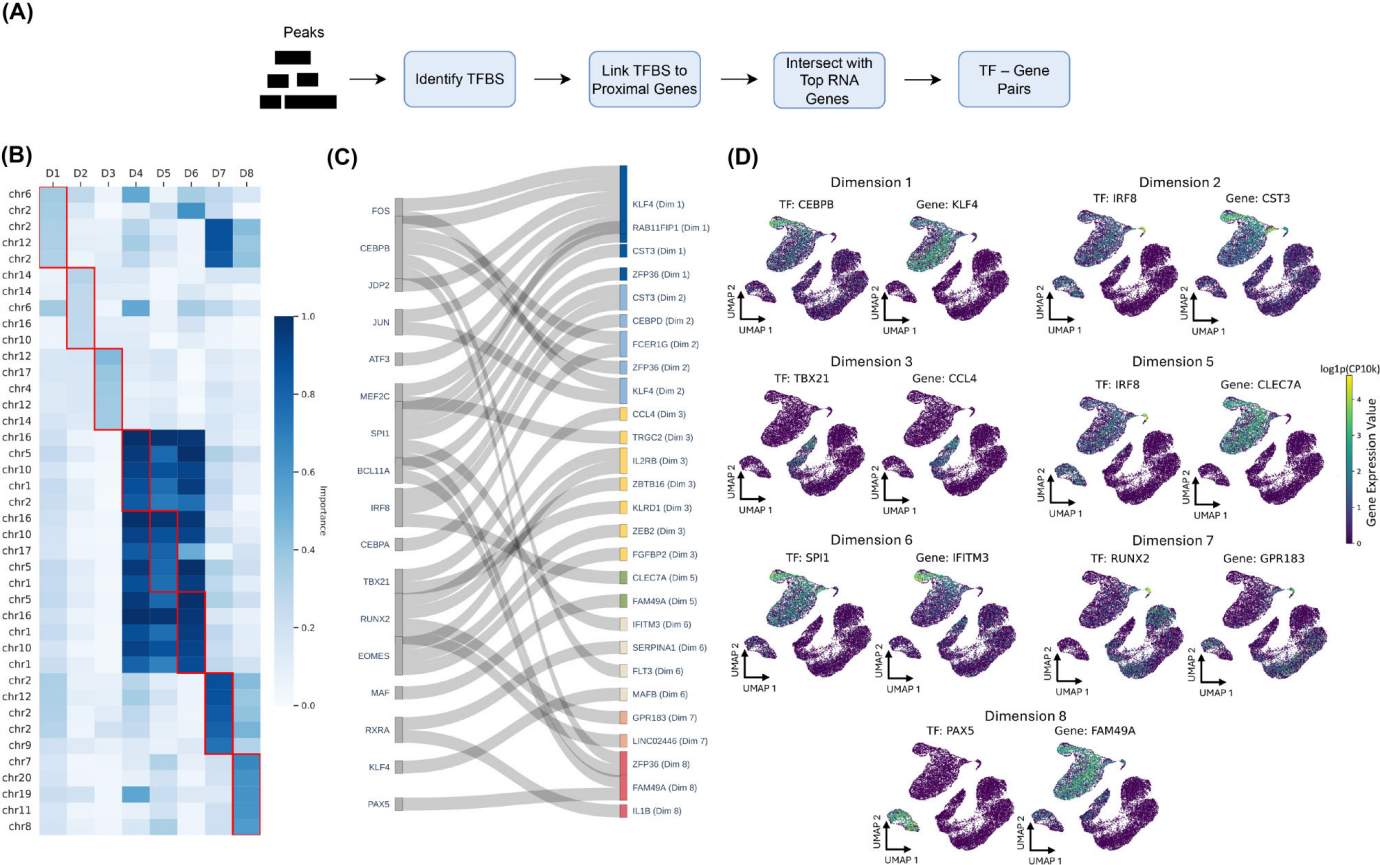
(A) Schematic of the workflow for identifying significant motifs in open chromatin peaks, mapping these motifs to nearby genes, and intersecting them with top RNA space genes to form motif–gene pairs. (B) Heatmap of gradient-based importance scores for the five most influential ATAC peaks in each GROTIA-derived dimension (D1–D8), where larger values indicates greater importance. (C) Sankey diagram illustrating dimension-specific gene–factor pairs. Genes (left) connect to their putative TFs (right), validated through literature. For instance, FOS are implicated as potential regulators of gene KFL4 in Dimensions 1 and CEBPB as potential regulators of gene FCR1G in Dimension 2. (D) UMAP embeddings colored by gene expression values of selected gene–factor pairs from Dimensions 1, 3, 5, 6, 7, and 8. Warmer hues denote higher expression, highlighting dimension-specific regulatory landscapes. Co-expression patterns further support these putative regulatory relationships.

Similarly, in the ATAC domain, we ranked open chromatin peaks by their gradient-based contribution scores and performed motif discovery on the highest-impact peaks (see the “Methods” section). The PBMC-1 dataset is used to demonstrate the following experiments. Mapping each motif to its nearest gene within a 20 kb window revealed putative regulatory relationships linking epigenetic accessibility to transcriptional output. Figure 5A illustrates the procedure for identifying TF–gene pairs from the top-ranked peaks. For further details, please refer to the “Interpretable embeddings of GROTIA” section.

Figure 5B highlights the top-ranked peaks (based on partial derivative-based importance) across the eight GROTIA-derived dimensions (D1–D8), with the most influential peaks in each dimension outlined in red boxes. Figure 5C shows the inferred TF-gene pairs associated with each dimension. Finally, Fig. 5d presents a UMAP projection of the ATAC embedding, overlaid with the identified TF–gene pairs, visually illustrating potential co-expression or repression relationships. In Dimension 1, we identified a CEBPB–KLF4 pair. CEBPB is essential for proper monocyte development, including the survival of certain subsets, and likely induces KLF4 as part of the monocyte differentiation network. Indeed, PU.1 (encoded by SPI1) directly upregulates KLF4, and CEBPB cooperates with PU.1 to drive monopoiesis [34]. In Dimension 2, the IRF8–CST3 pair emerged. IRF8 directly activates CST3 (cystatin C) during macrophage differentiation, mediated by a unique promoter element that overlaps IRF and ETS sites and requires both IRF8 and an ETS partner (e.g., PU.1) [35]. Dimension 3 highlighted TBX21 (T-bet)–CCL4, wherein T-bet binds to and positively regulates CCL4 in Th1 cells. Genome-wide ChIP-chip experiments in human T cells confirmed CCL4 as a direct T-bet target, revealing T-bet binding in regulatory regions that activate CCL4 transcription [36]. In Dimension 5, we found IRF8–CLEC7A. Genome-wide binding studies have identified CLEC7A as an IRF8 target in human myeloid cells [37], and co-expression networks further link CLEC7A with an IRF8-centered module [38]. Consistently, aging human microglia upregulate CLEC7A alongside other “activated” microglial genes under the control of an IRF8/SPI1 (PU.1)/RUNX1/TAL1 network [39]. In Dimension 6, the SPI1(PU.1)–IFITM3 pair indicates that PU.1 controls a broad antiviral gene program in macrophages, with IFITM3 explicitly cited as a PU.1-regulated antiviral factor [40]. Dimension 7 highlighted RUNX2–GPR183. Finally, in Dimension 8, the PAX5–FAM49A pair was supported by evidence of PAX5 ChIP-seq peaks near FAM49A [41]. Notably, FAM49A is expressed at lower levels in PAX5-positive pro-B cells and is de-repressed in PAX5-deficient cells, indicating that PAX5 normally suppresses Fam49a expression during early B-cell development [42].

### GROTIA enables identification of cellular subpopulation on integrated space

Beyond simply projecting scRNA and scATAC profiles into a shared space, GROTIA provides a natural framework for discovering subpopulations in an unsupervised manner. The PBMC-1 dataset is used to demonstrate the following experiments. While many datasets come with predefined labels (e.g., annotated cell types), these annotations may be incomplete or coarse, especially when new subtleties or states exist that were not recognized during initial labeling. By clustering cells in GROTIA’s integrated latent space, we recover cell population structure that closely matches expert annotations and enable inference of corresponding cell labels in a second modality when annotations are available in only one modality.

**Figure 6:**
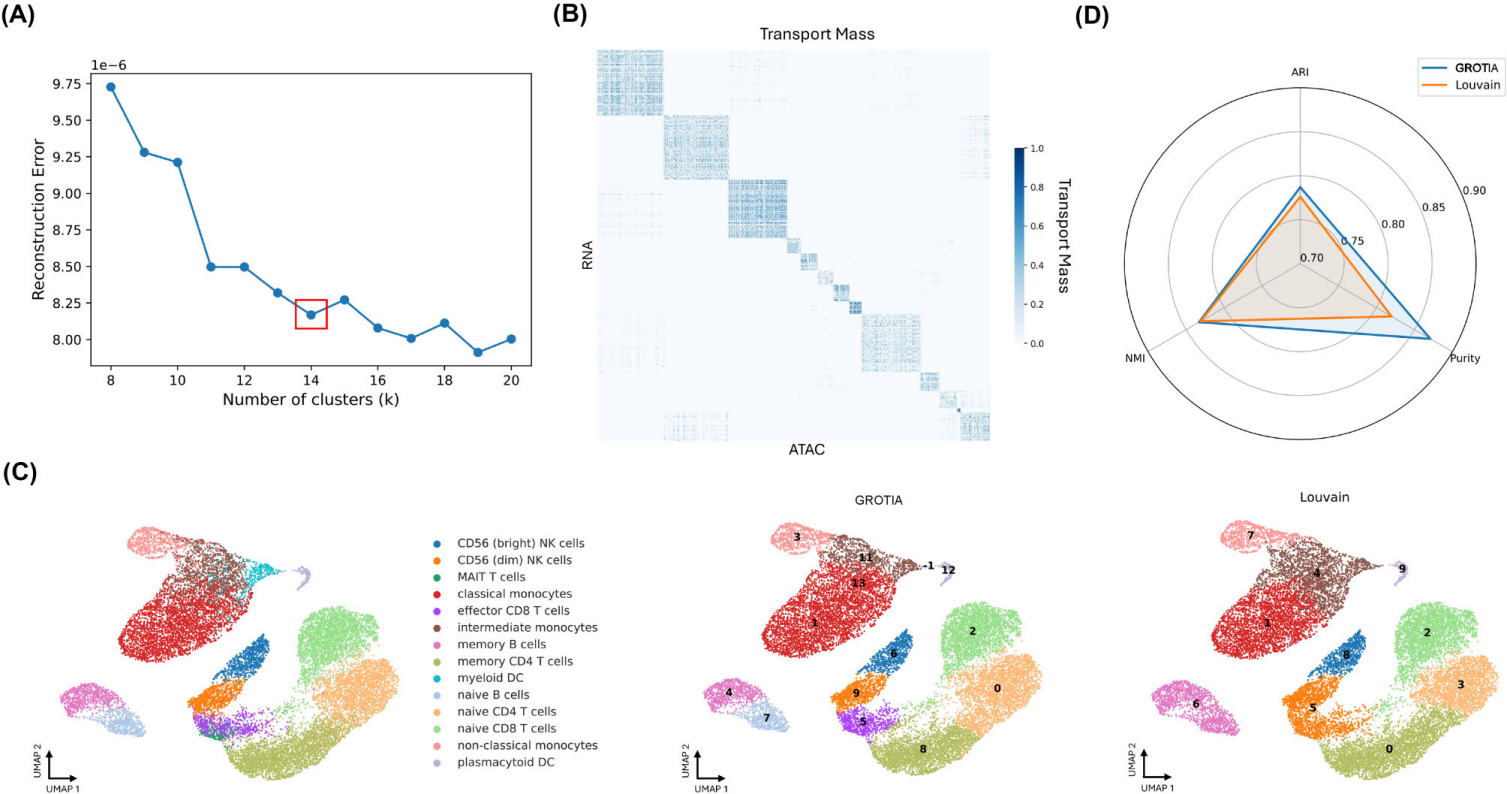
(A) Reconstruction error plotted against the number of clusters *k*, with the chosen *k* marked by a box. This optimal *k* balances clustering granularity and data fidelity. (B) Heatmap of the transport mass after co-clustering, illustrating how GROTIA aligns cells from scRNA and scATAC. The block-diagonal pattern indicates coherent groupings across both modalities. (C) UMAP projections of the integrated dataset, colored by true cell-type annotations (left), and clustered with GROTIA (center) or Louvain (right). For each method, predicted clusters are labeled by the ground-truth cluster with which they most overlap. GROTIA produces distinct subpopulations consistent with known cell-type boundaries. (D) Radar plot comparing GROTIA and Louvain on three clustering metrics (ARI, NMI, and purity). GROTIA demonstrates higher or comparable performance, indicating its ability to robustly identify meaningful subpopulations.

To determine the optimal number of clusters *k*, we plot the reconstruction error for various *k*-values and look for a distinct elbow (Fig. 6A). Beyond this point, further increasing *k* offers minimal improvement in accuracy while potentially fragmenting biologically coherent groups. We therefore select the *k* at the bump, yielding a robust trade-off between clustering granularity and data fidelity. We then visualize the resulting assignments alongside the original cell-type labels on a UMAP projection (Fig. 6C). Notably, GROTIA identifies distinct subpopulations that align well with known major cell types, yet can also isolate refined subclusters reflecting subtle transcriptional and epigenetic differences.

Finally, to benchmark the quality of GROTIA’s partitioning, we compare against a conventional community detection algorithm (Louvain) using three standard clustering metrics: adjusted Rand index (ARI), normalized mutual information (NMI), and purity (Fig. 6d). We tuned the resolution parameter over the grid resolution $\in \lbrace 0.1,\, 0.3,\, 0.4,\, 0.5,\, 0.6,\, 1.0,\, 1.5\rbrace$ and selected the value that maximized the sum of ARI, NMI, and purity with respect to the ground truth cell type labels on that dataset.

Our method achieves comparable or better performance, demonstrating that GROTIA’s alignment-based approach not only reconciles multi-omic data but also preserves biologically meaningful structures when clustering.

## Discussion

We present GROTIA, a novel algorithm for unsupervised single-cell multi-omics integration that combines OT for global alignment with graph regularization to preserve local structure. Benchmarking against state-of-the-art methods in both unsupervised and semi-supervised settings, GROTIA delivers comparable or superior performance while offering a computationally efficient solution (see *Supplementary Appendix: Metric Performance*). Critically, our framework also includes an in-model interpretability mechanism, allowing users to identify which genes or peaks drive each dimension of the integrated embedding. This enables targeted downstream analyses—such as GO enrichment or motif discovery—to reveal meaningful biological processes in the RNA and ATAC spaces.

Beyond aligning multi-omics data, GROTIA leverages its transport plan for post-integration clustering, offering a data-driven approach to refine or correct misannotations in existing labels. Notably, unlike methods that require shared features across modalities, GROTIA only assumes that cells (rather than individual genes or peaks) follow a similar distribution if they belong to the same type or lineage—thus broadening its applicability to complex datasets.

Looking ahead, we plan to extend GROTIA to time-series multi-omics data, where paired measurements across multiple time points are becoming increasingly common. Furthermore, we will deepen our driver-gene analyses to decode how specific features shape the integrated embedding, with the goal of uncovering more nuanced regulatory processes across different cell states. Also, extending GROTIA with kernel approximations (e.g., Nyström or random features) or with mini-batch and sparse variants to reduce memory usage is an important direction for future work.

## Availability of source code and requirements

Lists the following:

- Project name: GROTIA.
- Project home page: https://github.com/PennShenLab/GROTIA.
- Operating system(s): Platform independent.
- Programming language: Python 3.8 or higher.
- License: MIT License.
- Package management: GitHub.
- Hardware requirements: CPU required; GPU optional.
- RRID:SCR_027088.

## Additional files

**Supplementary Figure S1**: UMAP Plot of Top Genes for Dimensions 2, 4, and 5.

**Supplementary Figure S2**: UMAP Plot of Top Genes for Dimensions 6, 7, and 8.

**Supplementary Figure S3**: Hyperparameter robustness with respect to latent dimension. Label transfer accuracy (*k* = 5) is shown for SNARE, scGEM, PBMC-1, and PBMC-2 across latent embedding dimensions $k \in \lbrace 5, 8, 16, 32\rbrace$.

**Supplementary Figure S4**: Hyperparameter grid over regularization strengths. Label transfer accuracy (*k* = 5) is shown as a function of the orthogonality weight $\lambda _{\mathrm{topo}}$ and the graph Laplacian weight $\lambda _{\mathrm{reg}}$ (both on logarithmic scales) for a fixed latent dimension and reach. GROTIA attains high accuracy across a broad plateau in $(\lambda _{\mathrm{topo}}, \lambda _{\mathrm{reg}})$, indicating robustness of performance to these regularization strengths.

**Supplementary Figure S5**: Robustness with respect to latent dimension. For each latent dimension $p \in \lbrace 5, 8\rbrace$, the violin shows the distribution of label transfer accuracy (*k* = 5) across all combinations of $(\lambda _{\mathrm{topo}}, \lambda _{\mathrm{reg}}, \mathrm{reach})$ in the grid search. Accuracy remains high and comparable for both values of *p*, indicating that GROTIA does not require fine tuning of the latent dimensionality.

**Supplementary Figure S6**: Robustness with respect to the Sinkhorn interaction scale. For each value of the reach parameter $\in \lbrace 0.1, 1.0, 5.0\rbrace$, the violin shows the distribution of label transfer accuracy (*k* = 5) across all combinations of $(p, \lambda _{\mathrm{topo}}, \lambda _{\mathrm{reg}})$ in the grid search. The similar, high-accuracy distributions indicate that GROTIA is insensitive to the precise choice of reach.

**Supplementary Table S1**: Computational performance of benchmarked methods on the PBMC-1 dataset (9,378 cells), reporting wall-clock runtime (minutes) and peak GPU memory usage.

**Supplementary Table S2**: Alignment performance by FOSCTTM under unsupervised setting (first 4 columns: Simulation 1, Simulation 2, Simulation 3, Synthetic RNA-seq).

**Supplementary Table S3**: Alignment performance by FOSCTTM under unsupervised setting (last 2 columns: scGEM and SNAREseq).

**Supplementary Table S4**: Alignment performance by label transfer accuracy $(k=5)$ under unsupervised setting (first 4 columns: Simulation 1, Simulation 2, Simulation 3, and Synthetic RNA-seq).

**Supplementary Table S5**: Alignment performance by label transfer accuracy $(k=5)$ under unsupervised setting (last 2 columns: scGEM and SNAREseq).

**Supplementary Table S6**: Alignment performance by FOSCTTM (the lower the better) under semi-supervised setting for the first four datasets.

**Supplementary Table S7**: Alignment performance by FOSCTTM (the lower the better) under semi-supervised setting for scGEM, SNAREseq, PBMC10X, and PBMC.

**Supplementary Table S8**: Alignment performance by label transfer accuracy ($k=5$) (the higher the better) under semi-supervised setting for the first four datasets.

**Supplementary Table S9**: Alignment performance by label transfer accuracy ($k=5$) (the higher the better) under semi-supervised setting for scGEM, SNAREseq, PBMC10X, and PBMC.

**Supplementary Table S10**: Gene Ontology enrichment analysis for Dimension 2.

**Supplementary Table S11**: Gene Ontology enrichment analysis for Dimension 4.

**Supplementary Table S12**: Gene Ontology enrichment analysis for Dimension 5.

**Supplementary Table S13**: Gene Ontology enrichment analysis for Dimension 6.

**Supplementary Table S14**: Gene Ontology enrichment analysis for Dimension 7.

**Supplementary Table S15**: Gene Ontology enrichment analysis for Dimension 8.

**Supplementary Table S16**: RKHS Gram diagnostics with and without the orthogonality penalty (mean over 5 seeds). Here, $\lambda _{\mathrm{ortho}}=0$ corresponds to no orthogonality regularization and $\lambda _{\mathrm{ortho}}=1$ to the default setting.

## List of abbreviations

FOSCTTM: Fraction of Samples Closer Than the True Match; GO: Gene Ontology; GUMA: generalized unsupervised manifold alignment; GROTIA: Graph-Regularized Optimal Transport Framework for Diagonal Single-Cell Integrative Analysis; LTA: label transfer accuracy; MMD: maximum mean discrepancy; PBMC: peripheral blood mononuclear cell; SCOT: single-cell multi-omics alignment with optimal transport; TF: transcription factor; TSS: transcription start sites; UnionCom: unsupervised topological alignment for single-cell multi-omics integration; WD: Wasserstein-based.

## Supplementary Material

giag012_Supplemental_File

giag012_Authors_Response_To_Reviewer_Comments_original_submission

giag012_GIGA-D-25-00229_original_submission

giag012_GIGA-D-25-00229_Revision_1

giag012_Reviewer_1_Report_original_submission Reviewer 1 -- 7/142025

giag012_Reviewer_2_Report_original_submission Reviewer 2 -- 9/5/2025

giag012_Reviewer_2_Report_Revision_1 Reviewer 2 -- 1/19/2026

## Data Availability

All additional supporting data are available in the GigaScience repository, GigaDB [43]. Machine learning annotations have been deposited in the DOME registry [44].
