# Supplementary material for: An interpretable Graph-Regularized Optimal Transport Framework for Diagonal Single-Cell Integrative Analysis
Source: Gigascience. 2026 Feb 9;15:giag012. doi: 10.1093/gigascience/giag012 (PMC12970605; doi:10.1093/gigascience/giag012)
Supplement: giag012_Supplemental_File [file giag012_supplemental_file.pdf]

Appendix

This appendix includes supplementary materials for "*An Interpretable Graph-Regularized Optimal Transport Framework for Diagonal Single-Cell Integrative Analysis*" by Zexuan Wang, Qipeng Zhan, Shu Yang, Zhuoping Zhou, Mengyuan Kan, Tianhua Zhai and Li Shen.

Additional Datasets

To evaluate robustness under unbalanced cell population settings, we considered a benchmark scenario in which one shared cell population is entirely missing from one modality. Specifically, we used a paired scRNA–scATAC dataset in which the scRNA-seq modality contains HeLa and HCT cell lines, while the scATAC-seq modality contains HeLa, HCT, and K562 cell lines. This setup creates an unbalanced integration scenario with mosaic structure and missing observations across modalities, mimicking realistic diagonal integration settings. Full details of the dataset could be found at Samaran et al. [14].

Quantitative results on the cell-line dataset demonstrate that GROTIA is robust to variability in cell population proportions (A3, A5, A7, A9). In the unsupervised setting, GROTIA achieves the highest label-transfer accuracy (0.954), slightly outperforming the next best method, UnionCom (0.947). Its FOSCTTM score (0.318) is the second best and close to the best score achieved by UnionCom (0.287), indicating competitive alignment quality without supervision.

In the semi-supervised setting, GROTIA attains a label-transfer accuracy of 0.985, matching the best-performing methods (MMD-MA and scConfluence with prior, both 0.985). Its FOSCTTM score (0.323) is also comparable to other strong baselines (0.280 for UnionCom and 0.272 for scConfluence with prior). Overall, these results show that even when the two modalities differ in feature space and cell-type composition, GROTIA reliably recovers shared cell-state structure without requiring shared features, while appropriately handling cell populations present in only one modality.

Metric performance

Table A1. Computational performance of benchmarked methods on the PBMC-1 dataset (9,378 cells), reporting wall-clock runtime (minutes) and peak GPU memory usage.

Method	GROTIA	UniPort	scConfluence	Unioncom	SCOT	MMD-MA
**Runtime (min)**	15	17	20.44	65	185	470
**Peak GPU Memory Allocated (GB)**	9.8	8.8	3.8	4.3	25.9	11.5
**Peak CPU Resident Memory (GB)**	5.2	0.4	1.5	13.2	0.2	1.0

Table A2. Alignment performance by FOSCTTM under unsupervised setting (First 4 columns: Simulation 1, Simulation 2, Simulation 3, Synthetic RNA-seq).

	Simulation 1	Simulation 2	Simulation 3	Synthetic RNA-seq
SCOT	0.088	0.025	**0.009**	0.001
MMD-MA	0.125	0.012	0.739	0.384
UnionCom	0.091	0.028	0.684	0.028
Uniport	0.632	0.313	0.426	0.495
Scconfluence	0.512	0.501	0.437	0.431
GROTIA(Ours)	**0.077**	**0.008**	**0.009**	**5e-5**

Table A3. Alignment performance by FOSCTTM under unsupervised setting (Last 2 columns: scGEM and SNAREseq).

	scGEM	SNAREseq	pbmc10X	pmbc	cell line
SCOT	**0.209**	0.218	0.072	0.142	0.439
MMD-MA	0.437	0.473	0.273	0.376	0.392
UnionCom	0.691	0.510	0.412	0.480	**0.287**
Uniport	0.412	0.216	0.566	0.487	-
Scconfluence(Diagonal)	0.474	0.418	0.372	0.503	0.488
Scconfluence(Prior)	-	-	0.067	0.110	0.387
GROTIA(Ours)	0.215	**0.216**	**0.049**	**0.104**	0.318

Table A4. Alignment performance by label transfer accuracy ($k = 5$) under unsupervised setting (First 4 columns: Simulation 1, Simulation 2, Simulation 3, Synthetic RNA-seq).

	Simulation 1	Simulation 2	Simulation 3	Synthetic RNA-seq
SCOT	**0.977**	0.977	**0.950**	**0.996**
MMD-MA	0.897	0.957	0.700	0.506
UnionCom	0.947	0.947	0.133	0.948
Uniport	0.36	0.577	0.35	0.433
Scconfluence	0.493	0.523	0.490	0.650
GROTIA(Ours)	0.967	**0.980**	**0.950**	**0.996**

Table A5. Alignment performance by label transfer accuracy ($k = 5$) under unsupervised setting (Last 2 columns: scGEM and SNAREseq).

	scGEM	SNAREseq	pbmc10X	pmbc	cell line
SCOT	0.423	0.852	0.894	0.722	0.680
MMD-MA	0.237	0.412	0.224	0.161	0.882
UnionCom	0.107	0.288	0.164	0.266	0.947
Uniport	0.260	0.705	0.123	0.271	-
Scconfluence(Diagonal)	0.305	0.590	0.132	0.155	0.744
Scconfluence(Prior)	-	-	0.891	0.735	0.748
GROTIA(Ours)	**0.588**	**0.947**	**0.897**	**0.802**	**0.954**

Table A6. Alignment performance by FOSCTTM (The lower the better) under semi-supervised setting for the first four datasets.

	Simulation 1	Simulation 2	Simulation 3	Synthetic RNA-seq
SCOT	0.070	0.022	**0.009**	0.001
MMD-MA	0.124	0.023	0.012	0.112
UnionCom	0.083	0.016	0.152	0.038
Uniport	0.520	0.313	0.426	0.485
Scconfluence	0.077	0.007	0.407	0.228
GROTIA(Ours)	**0.069**	**0.005**	**0.009**	**1e-6**

Table A7. Alignment performance by FOSCTTM (The lower the better) under semi-supervised setting for scGEM, SNAREseq, pbmc10X, and pbmc.

	scGEM	SNAREseq	pbmc10X	pbmc	cell line
SCOT	**0.192**	0.150	0.073	0.142	0.439
MMD-MA	0.201	0.150	0.277	0.374	0.378
UnionCom	0.209	0.265	**0.017**	0.258	0.280
Uniport	0.259	0.220	0.566	0.487	-
Scconfluence (Diagnola)	0.234	0.154	0.080	0.211	0.367
Scconfluence(Prior)	–	–	0.067	**0.101**	**0.272**
GROTIA(Ours)	0.213	**0.148**	0.045	0.113	0.323

Table A8. Alignment performance by label transfer accuracy ($k = 5$) (The higher the better) under semi-supervised setting for the first four datasets.

	Simulation 1	Simulation 2	Simulation 3	Synthetic RNA-seq
SCOT	0.937	0.977	**0.957**	**0.998**
MMD-MA	0.890	0.783	0.947	0.706
UnionCom	0.960	0.620	0.613	0.997
Uniport	0.360	0.577	0.350	0.442
Scconfluence	0.960	0.990	0.580	0.997
GROTIA(Ours)	**0.963**	**0.993**	0.950	**0.998**

Table A9. Alignment performance by label transfer accuracy ($k = 5$) (The higher the better) under semi-supervised setting for scGEM, SNAREseq, pbmc10X, and pbmc.

	scGEM	SNAREseq	pbmc10X	pbmc	cell line
SCOT	0.576	0.982	0.894	0.722	0.679
MMD-MA	0.588	0.942	0.357	0.258	**0.985**
UnionCom	0.582	0.423	**0.926**	0.343	0.980
Uniport	0.412	0.719	0.123	0.276	-
Scconfluence(Diagonal)	0.621	0.982	0.786	0.574	0.836
Scconfluence(Prior)	–	–	0.891	0.747	**0.985**
GROTIA(Ours)	**0.700**	**0.986**	0.922	**0.805**	**0.985**

Additional Gene Ontology enrichment results

Table A10. Gene Ontology enrichment analysis for Dimension 2

source	GO name	GO ID	p_value	term size	inter. size
GO:MF	calcium-dependent protein binding	GO:0048306	4.566e − 4	80	8
GO:MF	GTPase regulator activity	GO:0030695	2.350e − 3	495	17
GO:BP	toll-like receptor signaling pathway	GO:0002224	8.450e − 3	75	7
GO:BP	[l]positive regulation of NF-kappaB				
transcription factor activity	GO:0051092	2.748e − 2	124	8	
GO:BP	receptor internalization	GO:0031623	3.464e − 2	128	8
GO:CC	membrane raft	GO:0045121	4.975e − 4	291	13
GO:CC	trans-Golgi network membrane	GO:0032588	1.304e − 3	107	8
GO:CC	collagen-containing extracellular matrix	GO:0062023	2.733e − 2	425	13
GO:CC	actin filament	GO:0005884	2.747e − 2	121	7
GO:CC	cell leading edge	GO:0031252	3.002e − 2	429	13

Table A11. Gene Ontology enrichment analysis for Dimension 4

source	GO name	GO ID	p_value	term size	inter. size
GO:MF	immune receptor activity	GO:0140375	6.904e − 6	145	12
GO:BP	[l]positive regulation of protein-				
containing complex assembly	GO:0031334	3.732e − 3	199	11	
GO:BP	cell killing	GO:0001906	9.412e − 3	219	11
GO:BP	ruffle organization	GO:0031529	2.118e − 2	56	6
GO:CC	[l]COPII-coated ER to Golgi				
transport vesicle	GO:0030134	2.685e − 5	89	9	
GO:CC	ruffle	GO:0001726	1.041e − 2	182	9
GO:CC	focal adhesion	GO:0005925	2.953e − 2	423	13

Table A12. Gene Ontology enrichment analysis for Dimension 5

source	GO name	GO ID	p_value	term size	inter. size
GO:MF	GTPase regulator activity	GO:0030695	2.006e − 5	495	20
GO:MF	phospholipid binding	GO:0005543	1.540e − 3	484	17
GO:BP	[l]negative regulation of				
protein phosphorylation	GO:0001933	1.810e − 4	270	14	
GO:BP	[l]positive regulation of NF-kappaB				
transcription factor activity	GO:0051092	2.258e − 2	124	8	
GO:BP	regulation of GTPase activity	GO:0043087	2.717e − 2	210	10

Table A13. Gene Ontology enrichment analysis for Dimension 6

source	GO name	GO ID	p_value	term size	inter. size
GO:MF	[l]DNA-binding transcription				
factor binding	GO:0140297	$3.481e-2$	490	15	
GO:MF	immune receptor activity	GO:0140375	$3.850e-2$	145	8
GO:BP	phagocytosis	GO:0006909	$6.492e-6$	234	15
GO:BP	cellular response to metal ion	GO:0071248	$5.224e-4$	200	12
GO:BP	icosanoid biosynthetic process	GO:0046456	$1.485e-3$	57	7
GO:BP	cell killing	GO:0001906	$9.412e-3$	219	11
GO:BP	[l]positive regulation of				
interleukin-1 beta production	GO:0032731	$3.171e-2$	60	6	

Table A14. Gene Ontology enrichment analysis for Dimension 7

source	GO name	GO ID	p_value	term size	inter. size
GO:MF	immune receptor activity	GO:0140375	$3.729e-5$	145	11
GO:BP	cell-matrix adhesion	GO:0007160	$4.671e-2$	236	10
GO:CC	T cell receptor complex	GO:0042101	$4.214e-3$	136	8

Table A15. Gene Ontology enrichment analysis for Dimension 8

source	GO name	GO ID	p_value	term size	inter. size
GO:MF	immune receptor activity	GO:0140375	$6.680e-5$	145	11
GO:BP	cell killing	GO:0001906	$1.217e-4$	219	13
GO:BP	phagocytosis	GO:0006909	$1.248e-2$	234	11
GO:BP	[l]regulation of metal ion transport	GO:0010959	$4.048e-2$	369	13

Additional gene importance results

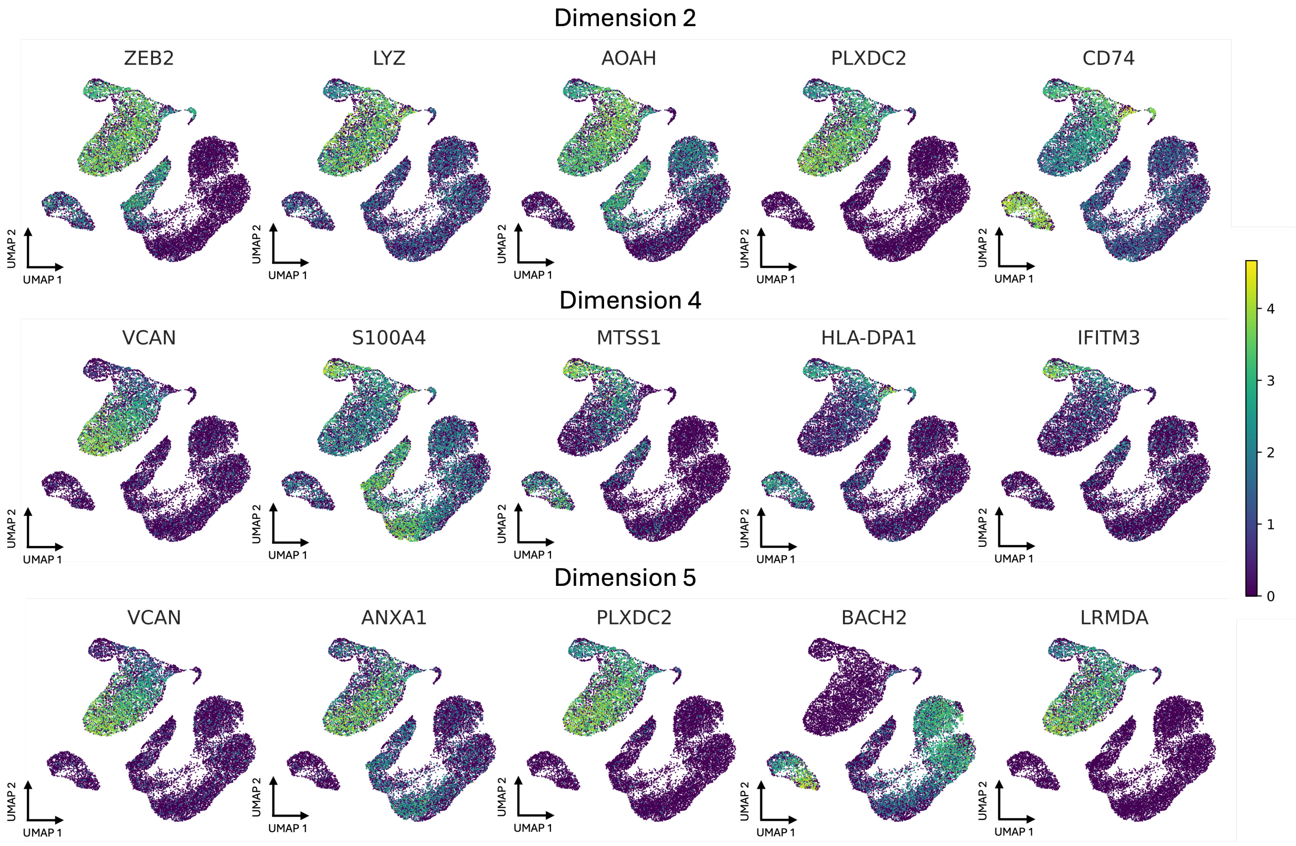

Figure A.1. UMAP Plot of Top Genes for Dimensions 2, 4, and 5.

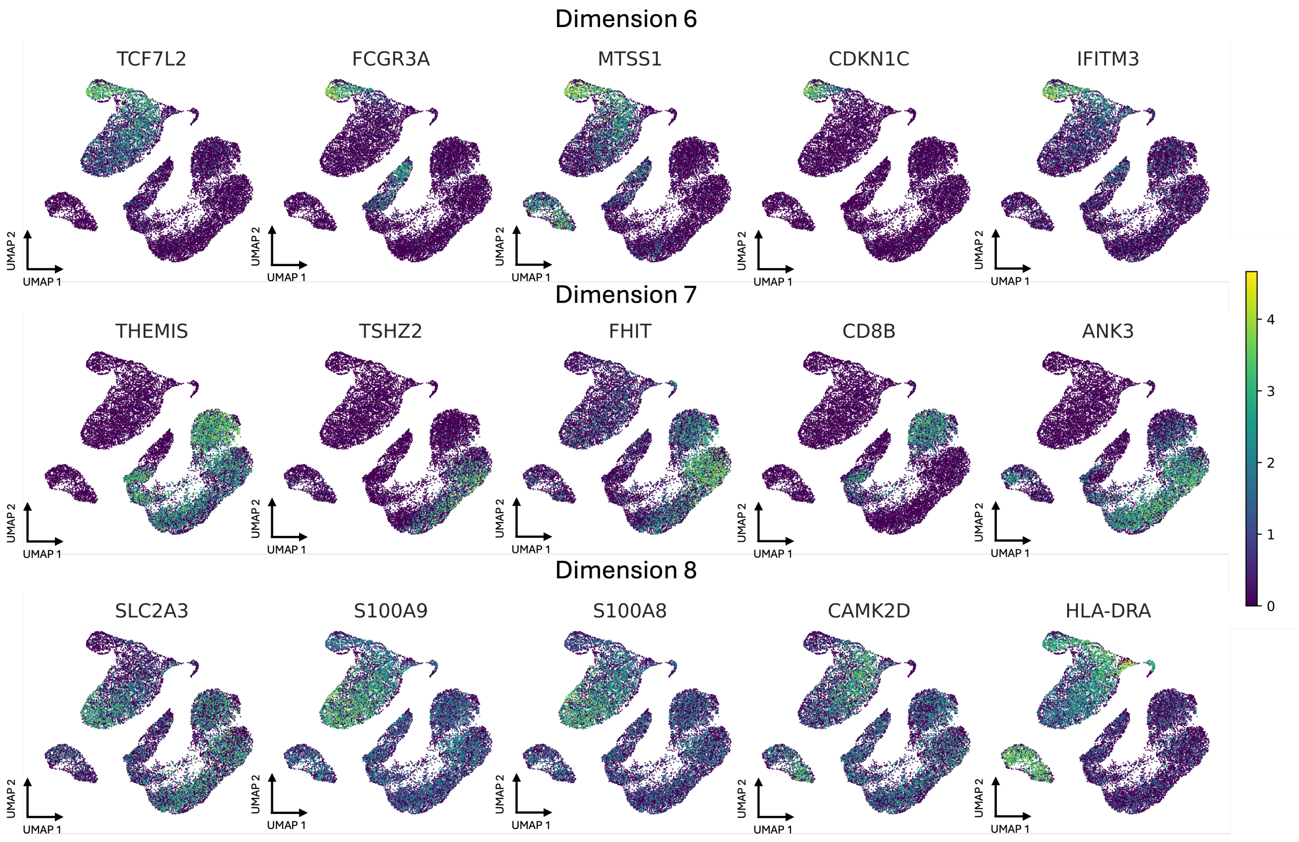

Figure A.2. UMAP Plot of Top Genes for Dimensions 6, 7, and 8.

Optimization Details

GROTIA Optimization

Algorithm 1 GROTIA optimization

Require: Datasets X, Y or precomputed kernels K_X, K_Y; graph Laplacians L_X, L_Y; latent dimension p; weights $\lambda_{\text{topo}}, \lambda_{\text{reg}}$; GeomLoss parameters (blur, reach, scaling); learning rate η; iterations T.

Initialize coefficients $\alpha \in R^{n_x \times p}$, $\beta \in R^{n_y \times p}$ with kernel PCA.
for $t = 1$ to T **do**
  $\widetilde{X} \leftarrow K_X \alpha, \quad \widetilde{Y} \leftarrow K_Y \beta$
  $L_{\text{OT}} \leftarrow \text{Sinkhorn}(\widetilde{X}, \widetilde{Y})$ {GeomLoss `SamplesLoss`}
  $L_{\text{ortho}} \leftarrow \|\alpha^\top K_X \alpha - I_p\|_F^2 + \|\beta^\top K_Y \beta - I_p\|_F^2$
  $L_{\text{graph}} \leftarrow \text{tr}(\widetilde{X}^\top L_X \widetilde{X}) + \text{tr}(\widetilde{Y}^\top L_Y \widetilde{Y})$
  $L \leftarrow L_{\text{OT}} + \lambda_{\text{topo}} L_{\text{ortho}} + \lambda_{\text{reg}} L_{\text{graph}}$
  $\alpha \leftarrow \alpha - \eta \frac{\partial L}{\partial \alpha}, \quad \beta \leftarrow \beta - \eta \frac{\partial L}{\partial \beta}$
end for
return $\widetilde{X} = K_X \alpha, \ \widetilde{Y} = K_Y \beta$

In all experiments we use the implementation summarized in Algorithm 1. For each modality we first compute a Gaussian kernel with bandwidth parameter $\gamma = \frac{1}{2\,\text{med}^2}$ where med is the median of all pairwise Euclidean distances between cells; the resulting RBF kernels are then centered before optimization. We construct k-nearest-neighbor graphs ($k = 5$) on the feature representations using correlation distance and build unnormalized Laplacians L_X, L_Y from the symmetrized adjacency matrices. The Sinkhorn optimal transport term L_{OT} is implemented via the `GeomLoss SamplesLoss` with `loss="sinkhorn"`, $p = 2$, and fixed internal parameters `blur` = 0.01 and `scaling` = 0.8. We optimize the total loss implemented implemented with Adam, double-precision arithmetic, a ReduceLROnPlateau scheduler, and early stopping if the loss does not improve for 10 checks or the learning rate falls below 10^{-6}. The learning rate is set to $\eta = 10^{-3}$ for datasets with fewer than 5,000 cells and $\eta = 10^{-5}$ for larger datasets.

We treat a small set of hyperparameters as tunable and fix all others to the defaults above. Specifically, for each dataset we perform a grid search over

$$p \in \{5, 8\}, \quad \lambda_{\text{topo}} \in \{1, 10^{-1}, 10^{-2}, 10^{-3}\}, \quad \lambda_{\text{reg}} \in \{10^{-3}, 10^{-4}, 10^{-5}, 10^{-6}, 10^{-7}, 10^{-8}\}, \quad \text{reach} \in \{0.1, 1.0, 5.0\},$$

where p is the latent dimension, λ_{topo} controls the RKHS orthogonality penalty, λ_{reg} controls the graph Laplacian smoothness, and `reach` is the `GeomLoss` parameter governing the effective interaction scale of the Sinkhorn loss. All other parameters (e.g. `blur`, `scaling`, learning rate, number of iterations, graph construction settings) are kept fixed at their default values. The selected configuration for each dataset and the corresponding performance metrics from this grid are reported in the appendix.

Effect of the RKHS Orthogonality Constraint

In GROTIA, the orthogonality penalty

$$\lambda_{\mathrm{ortho}}\left(\|P_X^\top K_X P_X - I_k\|_F^2 + \|P_Y^\top K_Y P_Y - I_k\|_F^2\right)$$

is introduced to regularize the latent functions in the Reproducing Kernel Hilbert Spaces (RKHSs) of the two modalities. Here $K_X \in R^{n_x \times n_x}$ and $K_Y \in R^{n_y \times n_y}$ denote the kernel Gram matrices, and the columns of P_X and P_Y define RKHS functions

$$f_j^X(\cdot) = \sum_{i=1}^{n_x} (P_X)_{ij} K_X(x_i, \cdot), \qquad f_j^Y(\cdot) = \sum_{i=1}^{n_y} (P_Y)_{ij} K_Y(y_i, \cdot).$$

By standard kernel theory,

$$\langle f_a^X, f_b^X \rangle_{\mathcal{H}_X} = (P_X)_a^\top K_X (P_X)_b,$$

so the matrices

$$G_X := P_X^\top K_X P_X, \qquad G_Y := P_Y^\top K_Y P_Y$$

are precisely the RKHS Gram matrices of the learned latent functions: $(G_X)_{ab} = \langle f_a^X, f_b^X \rangle_{\mathcal{H}_X}$ and similarly for Y. The penalty therefore drives G_X and G_Y towards the identity, i.e. it encourages the latent functions to form approximately orthonormal systems in their respective RKHSs.

If K_X is positive definite and $G_X = I_k$, then $\langle f_a^X, f_b^X \rangle_{\mathcal{H}_X} = \delta_{ab}$ and $\{f_j^X\}_{j=1}^k$ is an orthonormal system in $\mathcal{H}_X$ with $\mathrm{rank}(G_X) = k$. Moreover,

$$\|G_X - I_k\|_F^2 \;=\; \sum_{i=1}^{k} (\lambda_i(G_X) - 1)^2$$

in terms of the eigenvalues $\lambda_i(G_X)$, so the orthogonality penalty directly discourages both small eigenvalues (rank degeneracy) and large off–diagonal entries (correlation between latent functions).

To quantify the effect of this term, we compared models trained with and without the orthogonality penalty by setting $\lambda_{\mathrm{ortho}} = 0$ versus $\lambda_{\mathrm{ortho}} = 1$, while keeping all other hyperparameters fixed. For each setting we ran the optimization from multiple random seeds and evaluated, for both G_X and G_Y: (i) the eigenvalue spectrum and an effective rank, and (ii) the off–diagonal structure of G as a measure of redundancy between axes. In addition, it helps avoid collapsed embeddings induced by the graph Laplacian terms.

Prevention of rank degeneracy in RKHS

If the orthogonality penalty were minimized exactly, we would have $G_X = I_k$ and $G_Y = I_k$, so the latent functions would form orthonormal bases of k-dimensional subspaces in $\mathcal{H}_X$ and $\mathcal{H}_Y$, respectively. In particular, all eigenvalues of G_X and G_Y would be equal to 1 and the representations would be full-rank in RKHS.

Empirically, when the orthogonality penalty was removed ($\lambda_{\mathrm{ortho}} = 0$), the eigenvalue spectra of G_X and G_Y were highly anisotropic: almost all spectral mass was concentrated in a single dominant eigenvalue, while the remaining eigenvalues were several orders of magnitude smaller. In our experiments with $k = 8$, we obtained for modality X

$$\lambda(G_X) \approx \left(1.7\times10^{-4},\, 5.5\times10^{-4},\, 1.0\times10^{-3},\, 2.0\times10^{-3},\, 2.6\times10^{-3},\, 3.1\times10^{-3},\, 4.2\times10^{-2},\, 8.8\times10^{-1}\right),$$

and for modality Y

$$\lambda(G_Y) \approx \left(2.0\times10^{-4},\, 7.0\times10^{-4},\, 1.0\times10^{-3},\, 1.7\times10^{-3},\, 3.3\times10^{-3},\, 4.0\times10^{-3},\, 3.7\times10^{-2},\, 7.6\times10^{-1}\right).$$

Thus, although we nominally learn $k = 8$ latent functions, almost all RKHS energy lies in a single direction and several eigenvalues are two–three orders of magnitude smaller than the largest; the effective rank

$$\text{eff-rank}(G) := \#\{\lambda_i(G) > 10^{-3}\}$$

is only 6 for both modalities, indicating a near rank-deficient representation in RKHS.

In contrast, when the orthogonality term was active ($\lambda_{\mathrm{ortho}} = 1$), all eigenvalues of G_X and G_Y were numerically equal to 1 up to floating-point precision (approximately 0.9999998–0.99999999), and the effective rank matched the target $k = 8$ in all runs. This confirms that the orthogonality penalty empirically enforces a non-degenerate, k-dimensional RKHS representation and prevents the model from collapsing most of the RKHS energy into a single direction.

Table A16. RKHS Gram diagnostics with and without the orthogonality penalty (mean over 5 seeds). Here $\lambda_{\mathrm{ortho}} = 0$ corresponds to no orthogonality regularization and $\lambda_{\mathrm{ortho}} = 1$ to the default setting.

λ_{ortho}	Modality	eff_rank	$\|G_{\mathrm{off}}\|_F$	mean$\|G_{\mathrm{off}}\|$	max$\|G_{\mathrm{off}}\|$
0	X	6.0	7.55×10^{-1}	6.66×10^{-2}	2.50×10^{-1}
0	Y	6.0	6.51×10^{-1}	5.73×10^{-2}	2.11×10^{-1}
1	X	8.0	2.67×10^{-15}	2.35×10^{-16}	9.12×10^{-16}
1	Y	8.0	2.98×10^{-15}	2.76×10^{-16}	9.92×10^{-16}

Reduction of redundancy between latent axes

The off–diagonal entries of G_X and G_Y encode the RKHS inner products between distinct latent functions. Large off–diagonal values indicate that different latent axes are strongly correlated or nearly collinear in RKHS, and therefore redundant. To quantify this, we considered the Frobenius norm of the off–diagonal part,

$$\|G_{\mathrm{off}}\|_F := \Big\|G - \mathrm{diag}\big(\mathrm{diag}(G)\big)\Big\|_F = \sqrt{\sum_{a\neq b} G_{ab}^2},$$

as well as the mean and maximum absolute off–diagonal entries.

Without the orthogonality penalty ($\lambda_{\mathrm{ortho}} = 0$), we observed substantial off–diagonal mass in both G_X and G_Y. For modality X, the mean absolute off–diagonal entry was approximately 6.7×10^{-2}, with a maximum of 2.5×10^{-1} and $\|G_{\mathrm{off}}\|_F \approx 7.6 \times 10^{-1}$. For modality Y, the corresponding values were approximately 5.7×10^{-2}, 2.1×10^{-1}, and 6.5×10^{-1}. These numbers confirm that, in the absence of the orthogonality term, the learned latent functions are strongly correlated in RKHS, and several axes effectively capture overlapping structure.

When $\lambda_{\mathrm{ortho}} = 1$, the off–diagonal entries of G_X and G_Y collapsed to numerical zero: $\|G_{\mathrm{off}}\|_F$ was on the order of 10^{-15}, and the mean absolute off–diagonal entries were on the order of 10^{-16} for both modalities. This is consistent with the intended effect of the penalty: different latent functions become RKHS-orthogonal in practice, and the model no longer allocates capacity to redundant, collinear directions.

For convenience, Table A16 summarizes the rank and redundancy diagnostics averaged over random seeds.

Avoiding collapsed embeddings

It also prevents embedding collapse due to graph Laplacian regularization. Without the orthogonality penalty (i.e. with $\lambda_{\mathrm{ortho}} = 0$), the GROTIA objective reduces to

$$\mathcal{L}_{\mathrm{OT}}(\widetilde{X}, \widetilde{Y}) \;+\; \lambda\Big[\mathrm{Tr}(\widetilde{X}^\top L_X \widetilde{X}) + \mathrm{Tr}(\widetilde{Y}^\top L_Y \widetilde{Y})\Big].$$

Both the optimal transport loss $\mathcal{L}_{\mathrm{OT}}$ and the graph Laplacian terms are nonnegative. In particular, if we take

$$\widetilde{X} = 0, \qquad \widetilde{Y} = 0 \quad \Longleftrightarrow \quad P_X = 0,\; P_Y = 0,$$

then every term in the expression above is exactly zero. Thus $(P_X, P_Y) = (0, 0)$, i.e. a fully collapsed embedding where all cells are mapped to the origin, is one of the global minimizers of the graph-regularized OT objective when the orthogonality constraint is absent.

The RKHS orthogonality penalty is specifically designed to rule out such collapsed solutions. It adds

$$\lambda_{\mathrm{ortho}}\big(\|P_X^\top K_X P_X - I_k\|_F^2 + \|P_Y^\top K_Y P_Y - I_k\|_F^2\big)$$

to the objective. At the collapsed solution $P_X = 0, P_Y = 0$ we have

$$P_X^\top K_X P_X = 0, \qquad P_Y^\top K_Y P_Y = 0,$$

so the penalty becomes

$$\lambda_{\mathrm{ortho}}\big(\|0 - I_k\|_F^2 + \|0 - I_k\|_F^2\big) = 2\lambda_{\mathrm{ortho}}\,\|I_k\|_F^2,$$

which is large and strictly positive. In contrast, for non-collapsed embeddings where the latent RKHS functions are close to orthonormal (so that $P_X^\top K_X P_X \approx I_k$ and $P_Y^\top K_Y P_Y \approx I_k$), this penalty is close to zero.

Consequently, once the orthogonality term is active ($\lambda_{\mathrm{ortho}} > 0$), the trivial collapsed solution $\widetilde{X} = \widetilde{Y} = 0$ is no longer optimal: any embedding that satisfies the orthogonality constraint reasonably well will achieve a strictly lower total objective value. In this simple sense, the RKHS orthogonality constraint stabilizes the model against collapse and enforces a non-degenerate latent embedding.

Hyperparameter Robustness Analysis

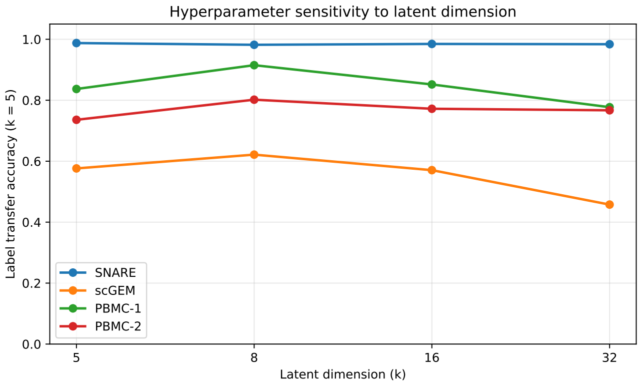

Figure A.3. Hyperparameter robustness with respect to latent dimension. Label transfer accuracy (k = 5) is shown for SNARE, scGEM, PBMC-1, and PBMC-2 across latent embedding dimensions $k \in \{5, 8, 16, 32\}$.

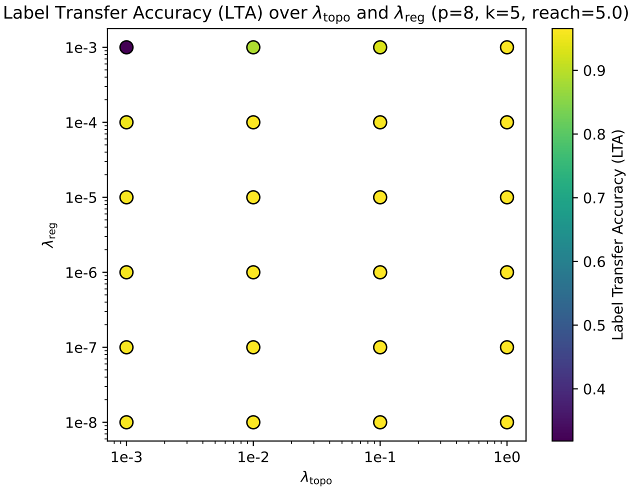

Figure A.4. Hyperparameter grid over regularization strengths. Label transfer accuracy (k = 5) is shown as a function of the orthogonality weight λ_{topo} and the graph Laplacian weight λ_{reg} (both on logarithmic scales) for a fixed latent dimension and reach. GROTIA attains high accuracy across a broad plateau in $(\lambda_{topo}, \lambda_{reg})$, indicating robustness of performance to these regularization strengths.

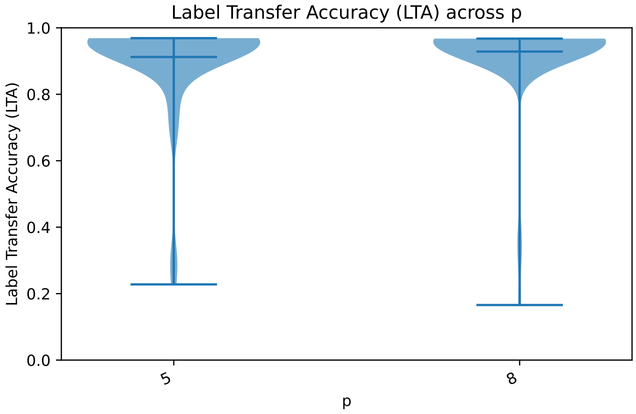

Figure A.5. Robustness with respect to latent dimension. For each latent dimension $p \in \{5, 8\}$, the violin shows the distribution of label transfer accuracy (k = 5) across all combinations of $(\lambda_{topo}, \lambda_{reg}, \text{reach})$ in the grid search. Accuracy remains high and comparable for both values of p, indicating that GROTIA does not require fine tuning of the latent dimensionality.

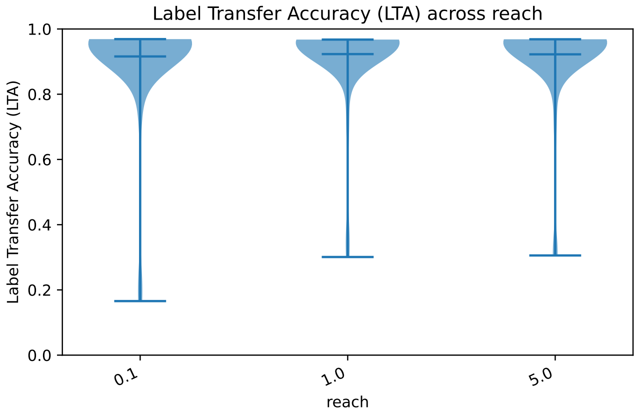

Figure A.6. Robustness with respect to the Sinkhorn interaction scale. For each value of the `reach` parameter $\in \{0.1, 1.0, 5.0\}$, the violin shows the distribution of label transfer accuracy (k = 5) across all combinations of $(p, \lambda_{topo}, \lambda_{reg})$ in the grid search. The similar, high-accuracy distributions indicate that GROTIA is insensitive to the precise choice of `reach`.

In all experiments, we tuned GROTIA's hyperparameters by monitoring Label Transfer Accuracy (LTA, $k = 5$). First, we examined the effect of the latent dimension p on each dataset (SNARE, scGEM, PBMC-1, PBMC-2; Fig. A.3). As shown in Fig. A.3, latent dimensions $p = 5$ and $p = 8$ consistently achieve the best or near-best label transfer accuracy across all evaluated datasets, whereas performance gradually degrades at higher dimensions ($p = 16$ and $p = 32$). We therefore restrict p to 5 or 8 in all reported experiments with GROTIA. Next, we examine the effect of hyperparameters on the SNARE dataset. To assess sensitivity to the regularization strengths, we then performed a grid search over λ_{topo} and λ_{reg} for a representative setting (Fig. A.4). The resulting 2D map shows a wide plateau of configurations with LTA > 0.9, confirming that performance is stable across several orders of magnitude in both penalties, except for a small corner with very strong graph regularization and very weak orthogonality. Finally, the violin plots (Figs. A.5 and A.6) summarize robustness by aggregating LTA across all combinations of the remaining hyperparameters: for both choices of p and for all values of the Sinkhorn reach parameter (reach $\in \{0.1, 1.0, 5.0\}$), the distributions are tightly concentrated near high LTA values, with only rare low-performing outliers. Together, these diagnostics indicate that GROTIA is robust to hyperparameter choices and that our chosen defaults lie in a broad region of stable performance.
