# Supplementary material for: An interpretable Graph-Regularized Optimal Transport Framework for Diagonal Single-Cell Integrative Analysis
Source: Gigascience. 2026 Feb 9;15:giag012. doi: 10.1093/gigascience/giag012 (PMC12970605; doi:10.1093/gigascience/giag012)

GigaScience

An Interpretable Graph-Regularized Optimal Transport Framework for Diagonal Single-Cell Integrative Analysis

--Manuscript Draft--

**Manuscript Number:**	GIGA-D-25-00229
**Full Title:**	An Interpretable Graph-Regularized Optimal Transport Framework for Diagonal Single-Cell Integrative Analysis
**Article Type:**	Technical Note
**Funding Information:**	National Institutes of Health (R01 AG071470) — Dr. Li Shen<br>National Institutes of Health (U19 AG074879) — Dr. Li Shen<br>National Institutes of Health (U01 AG066833) — Dr. Li Shen<br>National Institutes of Health (U01 AG068057) — Dr. Li Shen
**Abstract:**	Background: Recent advancements in single-cell omics technologies have enabled detailed characterization of cellular processes. However, coassay sequencing technologies remain limited, resulting in un-paired single-cell omics datasets with differing feature dimensions; Finding: we present GROTIA (Graph-Regularized Optimal Transport Framework for Diagonal Single-Cell Integrative Analysis), a computational method to align multi-omics datasets without requiring any prior correspondence information. GROTIA achieves global alignment through optimal transport while preserving local relationships via graph regularization. Additionally, our approach provides interpretability by deriving domain-specific feature importance from partial derivatives, highlighting key biological markers. Moreover, the transport plan between modalities can be leveraged for post-integration clustering, enabling a data-driven approach to discover novel cell subpopulations; Conclusions: We demonstrate GROTIA's superior performance on four simulated and four real-world datasets, surpassing state-of-the-art unsupervised alignment methods and confirming the biological significance of the top features identified in each domain. The software is available at https://github.com/PennShenLab/GROTIA.
**Corresponding Author:**	Li Shen, PhD<br>University of Pennsylvania<br>Philadelphia, PA UNITED STATES
**Corresponding Author Secondary Information:**	
**Corresponding Author's Institution:**	University of Pennsylvania
**Corresponding Author's Secondary Institution:**	
**First Author:**	Zexuan Wang
**First Author Secondary Information:**	
**Order of Authors:**	Zexuan Wang<br>Qipeng Zhan<br>Shu Yang<br>Zhuoping Zhou<br>Mengyuan Kan<br>Tianhua Zhai<br>Li Shen
**Order of Authors Secondary Information:**	

Powered by Editorial Manager® and ProduXion Manager® from Aries Systems Corporation

Additional Information:	
**Question**	**Response**
Are you submitting this manuscript to a special series or article collection?	No
**Experimental design and statistics**<br><br>Full details of the experimental design and statistical methods used should be given in the Methods section, as detailed in our Minimum Standards Reporting Checklist. Information essential to interpreting the data presented should be made available in the figure legends.<br><br>Have you included all the information requested in your manuscript?	Yes
**Resources**<br><br>A description of all resources used, including antibodies, cell lines, animals and software tools, with enough information to allow them to be uniquely identified, should be included in the Methods section. Authors are strongly encouraged to cite Research Resource Identifiers (RRIDs) for antibodies, model organisms and tools, where possible.<br><br>Have you included the information requested as detailed in our Minimum Standards Reporting Checklist?	Yes
**Availability of data and materials**<br><br>All datasets and code on which the conclusions of the paper rely must be either included in your submission or deposited in publicly available repositories (where available and ethically appropriate), referencing such data using a unique identifier in the references and in the “Availability of Data and Materials” section of your manuscript.	Yes

Powered by Editorial Manager® and ProduXion Manager® from Aries Systems Corporation

Have you have met the above requirement as detailed in our Minimum Standards Reporting Checklist?	
GigaScience has policies and guidelines in place for the use of generative AI-writing tools such as ChatGPT. If you have used such writing tools to assist with writing the manuscript this must be declared and cited in the text. Authors should not list AI-writing tools and other AI-assisted technologies as an author or co-author and should acknowledge that they are fully responsible for text generated or refined by AI-writing tools.<p><br><br>A summary of use (particularly in the introduction or among methods) needs to be included at the end of the paper, and the outputs should also be included as a supplementary file hosted in GigaDB or other open repositories. Please read our guidelines for more information. <p><br><br>By submitting to GigaScience, you are aware of the journal's AI-writing tools policy, and if you have declared use of such tools below, you have acknowledged this where appropriate in your manuscript and have made a summary of use and outputs available. <p><br><b>AI-assisted writing tools have been used in the preparation of this manuscript?	Yes

Manuscript

Click here to access/download;Manuscript;Main_Manuscript.pdf

GigaScience, 2023, 1–16

doi: xx.xxxx/xxxx
Manuscript in Preparation
Paper

PAPER

An Interpretable Graph-Regularized Optimal Transport Framework for Diagonal Single-Cell Integrative Analysis

Zexuan Wang[1,†], Qipeng Zhan[1†], Shu Yang[2], Zhuoping Zhou[1], Mengyuan Kan[2], Tianhuan Zhai[2] and Li Shen[2,*]

[1]Graduate Group in Applied Mathematics and Computational Science, University of Pennsylvania, Philadelphia, PA, USA and [2]Department of Biostatistics, Epidemiology and Informatics, Perelman School of Medicine, University of Pennsylvania, Philadelphia, PA, USA

[*]Correspondence: li.shen@pennmedicine.upenn.edu
[†]These authors contributed equally to this work.

Abstract

Background: Recent advancements in single-cell omics technologies have enabled detailed characterization of cellular processes. However, coassay sequencing technologies remain limited, resulting in un-paired single-cell omics datasets with differing feature dimensions; **Finding** we present GROTIA (Graph-Regularized Optimal Transport Framework for Diagonal Single-Cell Integrative Analysis), a computational method to align multi-omics datasets without requiring any prior correspondence information. GROTIA achieves global alignment through optimal transport while preserving local relationships via graph regularization. Additionally, our approach provides interpretability by deriving domain-specific feature importance from partial derivatives, highlighting key biological markers. Moreover, the transport plan between modalities can be leveraged for post-integration clustering, enabling a data-driven approach to discover novel cell subpopulations; **Conclusions**: We demonstrate GROTIA's superior performance on four simulated and four real-world datasets, surpassing state-of-the-art unsupervised alignment methods and confirming the biological significance of the top features identified in each domain. The software is available at `https://github.com/PennShenLab/GROTIA`.

Key words: Optimal Transport; Graph Laplacian; Single Cell; Multi Omics; Data Integration; Interpretable.

Introduction

The advancement of single-cell technology offers a comprehensive understanding of cellular heterogeneity and the dynamic evolution of cell states. Various single-cell measurements reveal different aspects: scRNA-seq [1, 2] provides detailed gene expression profiles, while scATAC-seq [3] sheds light on chromatin accessibility in individual cells. Integrating these datasets is crucial as it allows for a more holistic view of cellular mechanisms, enabling the correlation of transcriptional activity with chromatin states to better understand gene regulation and cellular function.

Lots of computational methods have recently been developed to integrate data across multiple modalities [4, 5]. A critical challenge for these algorithms is their reliance on correspondence information to identify alignments between paired cells. In practice, such information is often only partially available, hindering the effectiveness of existing strategies [6, 7, 8]. This limitation has led researchers to focus on diagonal integration under semi-supervised settings, where alignment is achieved without direct cell-to-cell correspondences, but cell type labels are still used for hyperparameter tuning. The Generalized Unsupervised Manifold Alignment (GUMA) method [9] aligns datasets by optimizing local geometric structures to establish a one-to-one correspondence. Building upon this, the Unsupervised topological alignment for single-cell multi-

Compiled on: June 12, 2025.
Draft manuscript prepared by the author.

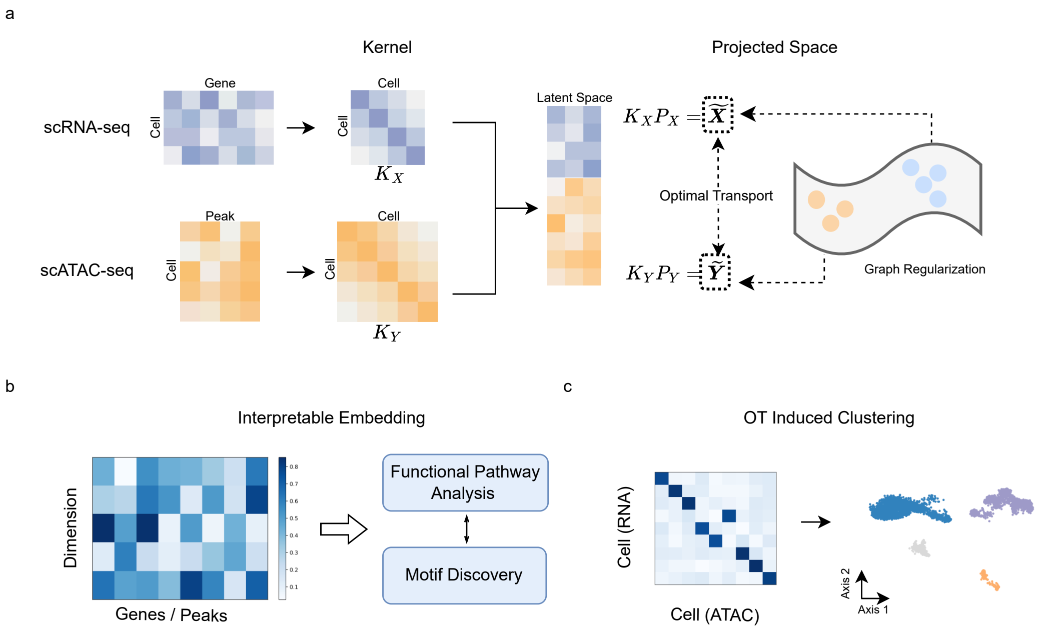

Figure 1. Overview of the GROTIA Framework for Multi-Omics Single-Cell Integration. (a) Schematic Design: For each single-cell modality (e.g., scRNA-seq, scATAC-seq), GROTIA constructs a kernel matrix capturing pairwise cell similarities (e.g., K_X and K_Y). It then learns mapping matrices P_X and P_Y to project cells from each modality's RKHS into a shared latent space, where distributions are aligned via optimal transport. A graph regularization term preserves local neighborhood structure, ensuring that cells close in the original domain remain similarly positioned after integration. (b) Interpretable Embedding: Once the shared embedding is obtained, GROTIA provides dimensionwise importance scores for genes or peaks. These scores can be used for downstream analyses such as Gene Ontology (GO) or motif discovery, provide biological interpretation of each latent dimension. (c) OT-Induced Co-Clustering: GROTIA leverages the cross-modality transport plan, which quantifies how strongly each scRNA cell aligns with each scATAC cell. By simultaneously grouping cells from both modalities according to these alignment strengths, GROTIA identifies co-clusters of subpopulations with closely matched regulatory states in the latent space.

omics integration (UnionCom) algorithm by Cao et al. [10] enables semi-supervised topological alignment, relaxing GUMA's strict one-to-one mapping requirement. Liu et al. [11] proposed an alternative manifold alignment strategy called MMD-MA, which employs the Maximum Mean Discrepancy (MMD) metric for alignment. Additionally, the Single-Cell Multi-Omics Alignment with Optimal Transport (SCOT) method [12] utilizes Gromov-Wasserstein distances to align multi-omic single-cell data. Autoencoder based method have also been proposed to align data across modality to use per ae per modality and align them in shared latent space [13, 14]. However, even when integration is performed without correspondences, hyperparameters are often tuned using cell label validation, rendering the process semi-supervised. Demetci et al. [12] demonstrated that most methods fail to adapt to fully unsupervised settings when no orthogonal alignment information is available.

Diagonal single-cell multi-omics integration thus faces several key obstacles. First, in the absence of paired samples, one must work under an unpaired assumption, which is common given the practical and financial difficulties of obtaining perfectly matched datasets. Second, integration often relies on shared features (e.g., overlapping genes) that may be missing or poorly represented across modalities. Third, most computational pipelines rely on label-based metrics for hyperparameter tuning, which is problematic in truly unsupervised settings where no external annotations exist. Finally, many existing methods lack an interpretable framework to explain the learned shared embeddings.

Here, we propose GROTIA, a fully unsupervised diagonal integration method that uses optimal transport and graph regularization to establish alignment without relying on one-to-one correspondences or labeled data. We embed each dataset in a high-dimensional kernel space to capture cell–cell similarities, then learn mappings that transform each dataset into a shared lower-dimensional space for direct comparison. Our framework preserves local geometry via graph Laplacian regularization while performing global alignment through optimal transport, thereby avoiding the need for label-based hyperparameter tuning. In addition, we provide gradient-based sensitivity analyses to highlight key biological genes and peaks that drive the alignment, clear interpretability of the learned latent representation.

We extensively evaluate our model against SCOT, MMD-MA, UnionCom, Uniport, Scconfluence across four simulated and four real-world datasets in unsupervised and semi-supervised settings. Our Graph-Regularized Optimal Transport (GROTIA) algorithm matches the performance of state-of-the-art methods. The schematic design of our approach is illustrated in **Figure 1**. The source code for GROTIA is publicly available at `https://github.com/PennShenLab/GROTIA`.

Methods

Simulated Datasets

We evaluated the GROTIA algorithm using four simulated datasets: three from Liu et al. [11], specifically designed to test alignment methods with different geometric structures, and one additional dataset from Demetci et al. [12], simulating single-cell RNA sequencing count data via Splatter [15]. Specifically, the first dataset presents a branch structure in two-dimensional space, the second a Swiss roll in three-dimensional space, and the third a circular frustum also in three-dimensional space. Although these datasets originally feature complex topological and geometric structures in low-dimensional spaces, they have been nonlinearly projected into high-dimensional feature spaces of 1000 and 2000 dimensions for evaluating alignment methods. The fourth is a synthetic RNA-seq

dataset from Demetci et al. [12], consisting of 5,000 cells with either 50 or 500 features. Following the approaches in the original publications, we applied Z-score normalization to all features before running alignment algorithms.

Real World Datasets

We then evaluated the GROTIA algorithm on four real-world datasets, widely recognized as gold-standard benchmarks in multi-omics integration and commonly used for assessing state-of-the-art methods. These include: (1) scGEM, which simultaneously profiles gene expression and DNA methylation [16]. ; (2) a dataset generated by the SNARE-seq assay, linking chromatin accessibility with gene expression [17].; (3) a human PBMC dataset (PBMC 10X) consisting of 9,378 cells per modality [14].; and (4) an additional PBMC 10X dataset containing 11,259 cells per modality [13]. We chose these paired multi-omics datasets specifically to enable diagonal integration with known ground-truth cell correspondences. Importantly, during benchmarking, all methods are provided with unpaired data, and the known cell-pairing information was used only for evaluating alignment accuracy.

The first real-world dataset, named "scGEM," measures gene expression and DNA methylation in the same cells and was generated using the scGEM assay. It contains human somatic cells reprogrammed to a pluripotent state, forming a continuous developmental trajectory. Cao et al. [10] and Demetci et al. [12] previously employed this dataset to evaluate integration methods. Specifically, it has 177 cells with 34 gene-expression features and 177 cells with 27 DNA methylation features. We used the preprocessed version from Demetci et al. [12].

The second real-world dataset, SNARE-seq, jointly profiles chromatin accessibility and gene expression. The dataset was preprocessed using cisTopic [18], resulting in an ATAC-seq matrix of 1,047 cells by 19 features and an RNA-seq matrix of 1,047 cells by 10 features. Following standard practice, unit normalization was then applied to these matrices. We used this preprocessed SNARE-seq dataset from Demetci et al. [12].

Additionally, we analyzed two multi-omics peripheral blood mononuclear cell (PBMC) datasets from publicly available 10x Genomics sources. The first, preprocessed by UniPort [14], contains 11,259 cells with 28,307 scATAC-seq features and 11,942 scRNA-seq genes. The second, preprocessed by scConfluence [13], includes 9,378 cells with 130,417 scATAC-seq features and 15,417 scRNA-seq genes. We used both PBMC datasets as provided for our integrative analyses.

Problem Formulation

We introduce a method to integrate single-cell datasets across different conditions or modalities. Let us consider two datasets, X and Y, with respective representations $X = \{x_1, \ldots, x_{n_x}\} \subset \mathcal{X}$ and $Y = \left\{y_1, \ldots, y_{n_y}\right\} \subset \mathcal{Y}$, where n_x and n_y denote the number cells in X and Y, respectively. We aim to uncover a shared manifold structure between X and Y without a priori correspondence between the datasets.

To achieve this, we first compute the intra-dataset kernels K_X and K_Y, which capture the internal structure of X and Y, respectively. As long as it is positive definite, each kernel corresponds to an implicit feature mapping $\phi_X : \mathcal{X} \to \mathcal{H}_X$ and $\phi_Y : \mathcal{Y} \to \mathcal{H}_Y$, where $\mathcal{H}_X$ and $\mathcal{K}_Y$ are the Reproducing Kernel Hilbert Spaces (RKHS) associated with K_X and K_Y. Subsequently, we seek mapping functions $f_X : \mathcal{X} \to R^k$ and $f_Y : \mathcal{Y} \to R^k$, where k is the dimensionality of the shared space. These functions are optimized so that the mapped representations $f_X(X)$ and $f_Y(Y)$ are well-aligned, thereby discovering the shared manifold structure.

Kernel Representation

To capture the intrinsic geometry of the datasets, we define the intra-dataset kernels K_X and K_Y using Gaussian kernel functions:

$$K_X(x_i, x_j) = \exp\left(-\frac{\|x_i - x_j\|^2}{2\sigma_X^2}\right), \quad K_Y(y_i, y_j) = \exp\left(-\frac{\|y_i - y_j\|^2}{2\sigma_Y^2}\right). \tag{1}$$

where σ_X and σ_Y are bandwidth parameters specific to X and Y, respectively. These kernels define the feature maps $\phi_X : \mathcal{X} \to \mathcal{H}_X$ and $\phi_Y : \mathcal{Y} \to \mathcal{H}_Y$ into their respective Reproducing Kernel Hilbert Spaces (RKHS).

We adopt a data-driven approach to determine σ_X and σ_Y by setting each parameter to the mean of the pairwise Euclidean distances within the corresponding dataset. This heuristic adjusts the bandwidths to reflect the average spatial dispersion of the data points, thereby tuning the kernels to the specific scale of variability in each dataset.

Optimal Transport

For simplicity, we will use the notation: $\widetilde{X} = f_X(X) \in R^{n_x \times k}$, $\widetilde{Y} = f_Y(Y) \in R^{n_y \times k}$ to represent the mapped datasets. The Sinkhorn divergence between the projected representations $\widetilde{X}$ and $\widetilde{Y}$ is defined as:

$$\mathcal{L}_{\mathrm{OT}}(\widetilde{X}, \widetilde{Y}) = \mathrm{OT}_\varepsilon(\widetilde{X}, \widetilde{Y}) - \frac{1}{2}\left(\mathrm{OT}_\varepsilon(\widetilde{X}, \widetilde{X}) + \mathrm{OT}_\varepsilon(\widetilde{Y}, \widetilde{Y})\right) \tag{2}$$

where $\mathrm{OT}_\varepsilon(\cdot,\cdot)$ denotes the entropically regularized optimal transport cost between two distributions. Next, we define the entropic optimal transport cost between $\widetilde{X}$ and $\widetilde{Y}$. The cost is computed as:

$$\mathrm{OT}_\varepsilon(\widetilde{X}, \widetilde{Y}) = \min_{T \in \Pi(\mathbf{a}, \mathbf{b})} \langle C, T \rangle + \varepsilon H(T) \tag{3}$$

where $\Pi(\mathbf{a}, \mathbf{b}) = \left\{T \in R_+^{n_x \times n_y} \mid T\mathbf{1}_{n_y} = \mathbf{a}, T^\top \mathbf{1}_{n_x} = \mathbf{b}\right\}$. The matrix $T \in R_+^{n_x \times n_y}$ is the transport plan matrix, representing the amount of mass transported from $\widetilde{x}_i$ to $\widetilde{y}_j$. The cost matrix $C \in R^{n_x \times n_y}$ quantifies the pairwise distances between the projected samples. Each element is defined as: $C_{ij} = \left\|\widetilde{x}_i - \widetilde{y}_j\right\|_2^2$. The parameter $\varepsilon > 0$ is the entropic regularization parameter that smooths the optimization problem and $H(T) = -\sum_{i=1}^{n_x}\sum_{j=1}^{n_y} T_{ij}\left(\log T_{ij} - 1\right)$ is the entropy of the transport plan T. The marginal distributions $\mathbf{a} \in R^{n_x}$ and $\mathbf{b} \in R^{n_y}$ are typically uniform distributions over the samples: $\mathbf{a} = \frac{1}{n_x}\mathbf{1}_{n_x}$, $\mathbf{b} = \frac{1}{n_y}\mathbf{1}_{n_y}$, where $\mathbf{1}_{n_x}$ and $\mathbf{1}_{n_y}$ are vectors of ones with lengths n_x and n_y, respectively. We utilize optimal transport over maximum mean discrepancy due to its benefits, such as non-vanishing gradients and other theoretical advantages [19].

Graph Laplacian Regularization

To capture the local geometric structures of the datasets X and Y, we construct graph Laplacians based on the k-nearest neighbor relationships defined through the Gaussian kernels. These Laplacians serve as regularizers for the mapping functions, enforcing smoothness by ensuring that nearby data points in the RKHS spaces $\mathcal{H}_X$ and $\mathcal{H}_Y$ remain close in the shared latent space.

For each dataset, we begin by constructing a k-nearest neighbor graph in the RKHS. Specifically, for dataset X, we identify the set of k-nearest neighbors for each feature-mapped data point $\phi_X(x_i)$, denoted as $\mathcal{N}_k(\phi_X(x_i))$, based on the distance metric in $\mathcal{H}_X$. The adjacency matrix $W_X \in R^{n_x \times n_x}$ is then defined with entries:

$$[W_X]_{ij} = \begin{cases} 1, & \text{if } \phi_X(x_j) \in \mathcal{N}_k(\phi_X(x_i)) \\ & \text{or } \phi_X(x_i) \in \mathcal{N}_k(\phi_X(x_j)) \\ 0, & \text{otherwise.} \end{cases} \quad (4)$$

Similarly, for dataset Y, we construct the adjacency matrix $W_Y \in R^{n_y \times n_y}$. Next, we compute the degree matrices D_X and D_Y, which are diagonal matrices where each diagonal entry represents the sum of the edge weights connected to a node: $[D_X]_{ii} = \sum_{j=1}^{n_x} [W_X]_{ij}$, $[D_Y]_{ii} = \sum_{j=1}^{n_y} [W_Y]_{ij}$. The graph Laplacians are then defined as the difference between the degree and adjacency matrices:

$$L_X = D_X - W_X, \quad L_Y = D_Y - W_Y \quad (5)$$

To regularize the projected representations $\widetilde{X}$ and $\widetilde{Y}$, we introduce smoothness terms based on the Laplacian quadratic form. Specifically, the smoothness term for $\widetilde{X}$ is given by:

$$\frac{1}{2}\sum_{i=1}^{n_x}\sum_{j=1}^{n_x} [W_X]_{ij} \left\|\widetilde{x}_i - \widetilde{x}_j\right\|^2 = \mathrm{Tr}\left(\widetilde{X}^\top L_X \widetilde{X}\right) \quad (6)$$

where $\widetilde{x}_i$ denotes the i-th row of $\widetilde{X}$. This expression encourages neighboring points in the original data space to have similar representations in the latent space, promoting smoothness in the mappings.

GROTIA Algorithm

To integrate the datasets X and Y into a shared latent space, we propose the Graph-Regularized Optimal Transport (GROTIA) algorithm. Our objective is to find the mapping $f_X : \mathcal{X} \to R^k, f_Y : \mathcal{Y} \to R^k$ that maps the data into a common k-dimensional space, effectively aligning their underlying manifold structures. The existence of such mapping is guaranteed by the representer theorem, which states that the optimal mappings can be expressed as finite linear combinations of the kernel functions:

$$[f_X]_j(x) = \sum_{i=1}^{n_x} \alpha_X^{ij} K_X(x_i, x) \quad (7)$$

where α_X^{ij} are the learned coefficients, K_X is the kernel function, and x_i are the samples from X. The coefficients α_X^{ij} are then organized into the matrix $P_X \in R^{n_x \times k}$, and similarity for Y we have $P_Y \in R^{n_y \times k}$. Thus, the final mapped representations are given by:

$$\widetilde{X} = K_X P_X, \quad \widetilde{Y} = K_Y P_Y \quad (8)$$

The optimization problem for the GROTIA algorithm is formulated as:

$$\begin{aligned} \min_{P_X, P_Y} \; & \mathcal{L}_{\mathrm{OT}}(\widetilde{X}, \widetilde{Y}) \\ & + \lambda \left[\mathrm{Tr}(\widetilde{X}^\top L_X \widetilde{X}) + \mathrm{Tr}(\widetilde{Y}^\top L_Y \widetilde{Y})\right] \\ & + \rho \left[\|P_X^\top K_X P_X - I_k\|_F^2 + \|P_Y^\top K_Y P_Y - I_k\|_F^2\right]. \end{aligned} \quad (9)$$

The first term $\mathcal{L}_{\mathrm{OT}}(\widetilde{X}, \widetilde{Y})$ aligns the global distributions of the datasets in the latent space using sinkhorn divergence. Minimizing the Sinkhorn divergence between the latent space representations ensures that the overall structures of X and Y are closely matched after projection. This captures global structural similarities and facilitates the discovery of shared manifold features between the datasets.

The second term $\mathrm{Tr}\left(\widetilde{X}^\top L_X \widetilde{X}\right)$ and $\mathrm{Tr}\left(\widetilde{Y}^\top L_Y \widetilde{Y}\right)$ is used preserve the local geometric structures inherent in each dataset. These terms penalize the weighted differences between neighboring points in the latent space. Doing so encourages neighboring points in the $\mathcal{H}_X, \mathcal{H}_Y$ to remain close in the latent space.

To prevent degenerate solutions and ensure that the mappings retain meaningful structure, we impose orthogonality constraints on the mapping matrices through the terms $\left(\|P_X^\top K_X P_X - I_k\|_F^2\right)$ and $\left(\|P_Y^\top K_Y P_Y - I_k\|_F^2\right)$.

By jointly optimizing this objective function, the GROTIA algorithm effectively balances global alignment and local structure preservation while ensuring that the projections are meaningful and well-behaved.

Interpretable Embeddings of GROTIA

In our approach, each domain (scRNA or scATAC) is mapped into a shared, low-dimensional space using kernel-based transformations. Take the scRNA space for example, let $X \in R^{n_x \times d_x}$ denote the data matrix for one domain, where n_x is the number of cells and d_x is the number of features. We construct a radial basis function (RBF) kernel $K \in R^{n_x \times n_x}$, with elements $K_{i,j} = \exp\left(-\gamma_X \left\|X_{i,\cdot} - X_{j,}\right\|^2\right)$ where γ_X is bandwidth parameter. Through our optimization procedure, we learn a coefficients matrix α_X^{ij} such that the k-dimensional embedding for the j-th cell is given by $[f_X]_j(x) = \sum_{i=1}^{n_x} \alpha_X^{ij} K_X(x_i, x)$. Here, $[f_X]_j(x)$ represents the coordinate of cell j in the k dimensional learned embedding. An analogous formulation with β is employed for the scATAC domain (using its own kernel matrix).

To identify which original features (e.g., genes in scRNA, peaks in scATAC) have the greatest influence on each embedding dimension, we compute partial derivatives of $f_d\left(x_j\right)$ with respect to each feature. Concretely, let $x_{j,g}$ denote the value of feature g in cell j. Then, for an RBF kernel

$$\frac{\partial}{\partial x_{j,g}} K_{i,j} = -2\gamma\left(x_{j,g} - x_{i,g}\right)\exp\left(-\gamma\left\|x_j - x_i\right\|^2\right). \quad (10)$$

Using the chain rule, the partial derivative of the d-th embedding coordinate with respect to $x_{j,g}$ becomes

$$\begin{aligned} \frac{\partial f_d}{\partial x_{j,g}}(x_j) &= \sum_{i=1}^{N} \alpha_{i,d} \frac{\partial K_{i,j}}{\partial x_{j,g}} \\ &= -2\gamma \sum_{i=1}^{N} \alpha_{i,d}(x_{j,g} - x_{i,g}) K_{i,j}. \end{aligned} \quad (11)$$

Thus, a large magnitude of $\left|\frac{\partial f_d}{\partial x_{j,g}}\left(x_j\right)\right|$ indicates that small perturbations in feature g for cell j induce substantial shifts in the d-th embedding coordinate. To obtain a global feature-importance measure, we average these derivatives across all cells:

$$I_{d,g} = \frac{1}{N}\sum_{j=1}^{N}\left|\frac{\partial f_d}{\partial x_{j,s}}\left(x_j\right)\right| \quad (12)$$

Features g with higher $I_{d,g}$ are deemed more influential in shaping dimension d. An identical procedure is applied in the scATAC domain using the learned projection β and its kernel K_Y.

To identify key molecular drivers in each latent dimension, we first ranked genes by their contribution scores $I_{d,g}$. The highest-ranked genes displayed significant variation in expres-

sion closely linked to the biological structure observed within the low-dimensional embedding. Gene ontology (GO) enrichment analyses conducted on these top-ranking genes using g:Profiler [20] with default parameters revealed strong enrichment for cellular metabolism-related processes.

In parallel, we investigated regulatory elements in the chromatin accessibility (ATAC) domain. Similar to the gene-ranking procedure, open chromatin peaks were ordered by their respective contribution scores for each GROTIA-derived dimension. We then performed de novo motif discovery on the most influential peaks using GimmeMotifs [21], applying a false discovery rate (FDR) threshold of <0.001. Each discovered motif was mapped to its nearest genes within a 20 kb window around their transcription start sites (TSS), a range widely used to capture most enhancer/promoter interactions [22, 23]. This strategy identified candidate transcription factor (TF)-gene pairs whose regulatory relationships may underlie the dimension-specific chromatin states observed in the low-dimensional embedding.

Co-Cluster Using Optimal Transport Plan

GROTIA also enables a post-integration analysis that yields data-driven clusters. Specifically, an optimal transport plan is first computed to quantify the cost or flow between two distinct sets of entities (e.g., cells in RNA and ATAC spaces). We then apply a co-clustering algorithm [24] directly to the resulting transport matrix to simultaneously group row and column entities. Treating the OT plan as a bipartite graph, the co-clustering approach identifies latent structural patterns, minimizing within-group transport costs while maximizing between-group separations.

Evaluated metrics

Each alignment method was assessed in two distinct evaluation modes, each paired with two quantitative metrics. Unsupervised mode tuned its hyper-parameters solely by minimizing the model's objective function, deliberately withholding any label information. Semi-supervised mode, in contrast, selected hyper-parameters that maximized downstream cell-type classification accuracy on a held-out validation set; crucially, these labels were used only during the tuning phase and were never provided to the model as inputs, preserving the semi-supervised setup.

The first metric is Fraction of Samples Closer Than the True Match (FOSCTTM). For each sample x_i in domain X, let the corresponding (true matched) sample in domain Y be y_i^*. We first embed both X and Y into a common space via embedding functions f_1 and f_2, respectively. We then define the distance between embedded points using a distance measure $d(\cdot,\cdot)$. The FOSCTTM metric for each sample x_i measures the fraction of samples in Y that are closer to x_i (in the embedded space) than its true match y_i^*. Formally,

$$R_i = \frac{1}{|Y|-1} \sum_{\substack{j \in Y \\ j \neq i}} 1\left\{ d\left(f_1(x_i), f_2\left(y_j\right)\right) < d\left(f_1(x_i), f_2\left(y_i^*\right)\right) \right\} \quad (13)$$

where $\mathbf{1}\{\cdot\}$ is the indicator function, returning 1 if the condition is satisfied and 0 otherwise. The FOSCTTM score for the entire dataset is the average of R_i across all $x_i \in X$:

$$\text{FOSCTTM} = \frac{1}{|X|} \sum_{i=1}^{|X|} R_i \quad (14)$$

A lower FOSCTTM value indicates better alignment, as it means fewer samples in Y are closer to x_i than the true match y_i^*.

The second metric is Label transfer accuracy (LTA) and it evaluates how well cell-type (or other categorical) labels can be transferred from domain X to domain Y in the integrated space. After embedding both datasets into a shared representation, each point $x_i \in X$ has a known label $L_X(x_i)$. We define $\text{kNN}(x_i)$ as the set of the k nearest neighbors of x_i in the embedded representation of Y. Let

$$\hat{L}(x_i) = \text{mode}\left\{L_Y(y) : y \in \text{kNN}(x_i)\right\} \quad (15)$$

where mode $(\cdot)$ returns the most common label among those k neighbors. The LTA score is then computed as:

$$\text{LTA} = \frac{1}{|X|} \sum_{i=1}^{|X|} \mathbf{1}\left\{\hat{L}(x_i) = L_X(x_i)\right\} \quad (16)$$

where $\mathbf{1}(\cdot)$ is the indicator function. A higher LTA indicates that the integrated embedding preserves biological labels more accurately between the two domains.

Training Details

We implemented GROTIA in PyTorch, using the Adam optimizer with a learning rate of 0.0001. Whenever the training loss plateaued, the learning rate was reduced by a factor of 0.5 . We selected the latent dimension to be either 5 or 8 and observed that GROTIA remained robust to this choice. To preserve local structure via the graph Laplacian, we set the number of nearest neighbors to 5. Two hyperparameters appear in the loss function: λ, which controls distribution matching between modalities, and ρ, which emphasizes local geometry within each modality. We searched over a grid of $\lambda \in \left\{1, 10^{-1}, 10^{-2}, 10^{-3}\right\}$ and $\rho \in \left\{10^{-3}, 10^{-4}, 10^{-5}, 10^{-6}\right\}$, with the constraint $\lambda > \rho$ to ensure that local structure is preserved and the mapping remains close to a projection.

Although GROTIA does not require identical features or genes across different modalities, it relies on the assumption of a shared underlying biology (e.g., cells of common lineages) so that datasets can be meaningfully aligned. As a standard preprocessing step to remove noisy cells and genes, we applied the following procedure to scRNA-seq data: (1) filter out cells containing fewer than 200 detected genes, (2) remove genes found in fewer than three cells, (3) log-normalize expression values by scaling each cell's total expression to 10,000 , (4) identify the 2,000 most highly variable genes, and (5) use the first 50 principal components of the processed data. For scATAC-seq data, we used a similar approach: (1) filter out cells with fewer than 200 detected genes, (2) remove genes present in fewer than three cells, (3) apply TF-IDF normalization, and (4) use the first 50 principal components.

Baseline Settings

To benchmark our method (GROTIA), we compared it against several existing approaches, each downloaded and configured according to the authors' guidelines. SCOT (v1.0) was obtained from `https://github.com/rsinghlab/SCOT`. We provided the same PCA-preprocessed input to SCOT as to GROTIA, mirroring SCOT's original publication. We then tuned the hyperparameter based on the recommendations in the SCOT documentation.

We downloaded UnionCom (v0.4.0) and applied the same input as in GROTIA, again following the developers' suggested preprocessing steps. All hyperparameters were tuned according to the guidance provided in the UnionCom package.

For UniPort (v1.3), which supports diagonal integration (i.e., mode=d) to align datasets without common genes, we used 2,000 highly variable genes from the scRNA-seq data and peaks exceeding a threshold of 1 for the scATAC-seq data. We also employed TF-IDF normalization, replicating the tutorial steps outlined by the UniPort authors. We obtained the MMD-MA (v1.0) PyTorch implementa-

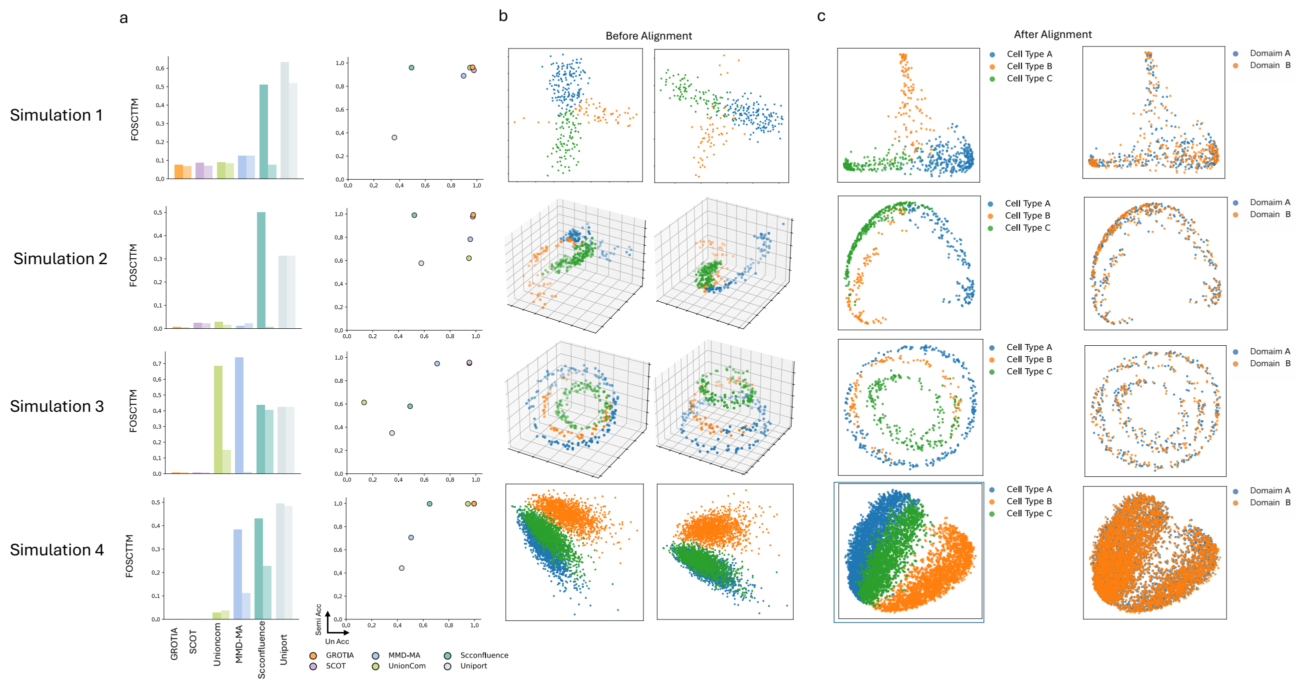

Figure 2. Benchmarking results on simulated datasets. a) Evaluation of Label Transfer Accuracy and Fraction of Samples Closer Than the True Match (FOSCTTM) across five benchmarked methods under two evaluation modes: semi-supervised and unsupervised. For each method, two bars are shown in the same plot, with the left bar representing the semi-supervised mode and the right bar representing the unsupervised mode for FOSCTTM. Label Transfer Accuracy is shown with semi-supervised results on the x-axis and unsupervised results on the y-axis. b) Visualization of simulated datasets before integration. Simulations 1 and 4 are visualized using the first two PCA components, whereas Simulations 2 and 3 are visualized using the first three axes from multidimensional scaling (MDS). c) Visualization after integration. The first column displays data colored by cell type, while the second column shows data colored by domain. All four datasets are visualized using the first two PCA components.

tion from `https://bitbucket.org/noblelab/2020_mmdma_pytorch/src`. As with GROTIA and SCOT, we provided PCA-preprocessed data and tuned its hyperparameters in accordance with the authors' guidelines.

Lastly, we downloaded scConfluence (v0.1.1). For the version without prior information, we set $\lambda_{IOT} = 0$, forcing a diagonal integration approach. When using scConfluence with prior information, we followed the recommended settings from the authors. We also tuned the remaining hyperparameters according to their instructions, applying identical preprocessing to ensure fair comparisons across all methods.

Results

GROTIA integrated simulated datasets in both semi and unsuperviseed setting

We evaluated the GROTIA algorithm using four simulated datasets previously discussed. Performance of the GROTIA algorithm was compared against five benchmarked methods: SCOT, Unioncom, MMD-MA, and two VAE-based approaches, Scconfluence and Uniport. Evaluations were conducted under two scenarios: semi-supervised (partial label information available) and unsupervised (no label information available).

In Figure 2a, the left column (solid color) and right column (vertical axis) display FOSCTTM and label transfer accuracy, respectively, under the semi-supervised setting. In the semi-supervised scenario, GROTIA and SCOT demonstrated consistently high performance across all datasets, with Unioncom and MMD-MA closely following. Scconfluence and Uniport showed comparatively lower performance, possibly due to their reliance on cross-modality guidance, which was not available in these simulations. In Figure 2b, each dataset from each domain is shown prior to integration, while Figure 2c shows the integrated dataset, colored by cell type on the left and by domain on the right.

In Figure 2a, the left column (solid color) and right column (vertical axis) display FOSCTTM and label transfer accuracy, respectively, under the semi-supervised setting. In the unsupervised scenario, where alignment depends solely on intrinsic structural information, GROTIA and SCOT maintained relatively stable performance. Other methods showed varying degrees of accuracy reduction, notably Unioncom on dataset 3, MMD-MA on datasets 3 and 4, Scconfluence on datasets 1, 2, and 4, and Uniport on dataset 1. These variations highlight the challenges inherent in unsupervised alignment without label guidance.

The stable performance of GROTIA can be attributed to their effective use of Wasserstein-based (WD) losses, capturing intrinsic geometric structures. Additionally, GROTIA employs orthogonality constraints within the Reproducing Kernel Hilbert Space (RKHS), enhancing embedding interpretability and stability. Although VAE-based methods also utilize WD losses, their embeddings can be susceptible to rotational variations, affecting alignment stability in the absence of label information. Detailed numerical results corresponding to the metric plots are presented in Supplementary Tables A2-A5.

GROTIA integrated real word datasets in both semi and unsupervised setting

We then evaluated the GROTIA algorithm on four real-world datasets. We chose these paired multi-omics datasets specifically to enable diagonal integration with known ground-truth cell correspondences. Importantly, during benchmarking, all methods are provided with unpaired data, and the known cell-pairing information was used only for evaluating alignment accuracy.

The benchmarking process used here follows the same procedure described in the simulation study, with one modification: for the PBMC-1 and PBMC-2 datasets, we additionally include scConfluence with prior information. We do not include the prior version

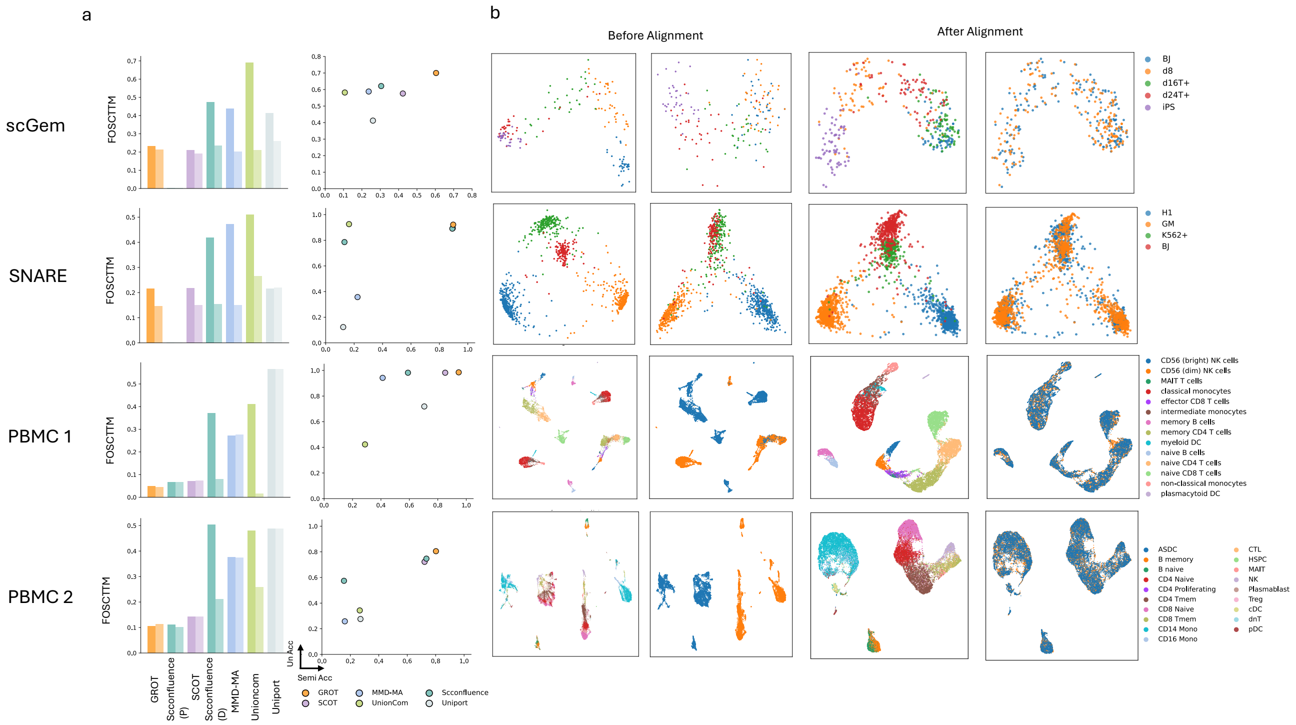

Figure 3. Benchmarking results on real word datasets. a) Evaluation of Label Transfer Accuracy and Fraction of Samples Closer Than the True Match (FOSCTTM) across five benchmarked methods under two evaluation modes: semi-supervised and unsupervised. For each method, two bars are shown in the same plot, with the left bar representing the semi-supervised mode and the right bar representing the unsupervised mode for FOSCTTM. Label Transfer Accuracy is shown with semi-supervised results on the x-axis and unsupervised results on the y-axis. b) Visualization of real word datasets before integration. ScGem and SNARE are visualized using the first two PCA components, whereas PBMC 1 and PBMC 2 are visualized using the first two UMAP Components. c) Visualization after integration. The first column displays data colored by cell type, while the second column shows data colored by domain. ScGem and SNARE are visualized using the first two PCA components, whereas PBMC 1 and PBMC 2 are visualized using the first two UMAP Components.

of scConfluence for the scGEM and SNARE datasets because the original authors performed dimensionality reduction to process these data, meaning commonly used gene selection methods are not available and the resulting feature space is small.

In Figure 3a, the left column (solid color) and right column (vertical axis) display FOSCTTM and label transfer accuracy, respectively, under the semi-supervised setting. Across the scGEM and SNARE-seq datasets, GROTIA achieves the second-best FOSCTTM in scGEM and the best in SNARE-seq, while attaining the highest label transfer accuracy in both. SCOT obtains the top FOSCTTM in scGEM but ranks second in the remaining evaluations. scConfluence (default), MMD-MA, UnionCom, and UniPort follow in overall performance. For the two PBMC datasets, GROTIA places second in FOSCTTM for both PBMC-1 and PBMC-2; in PBMC-2, scConfluence with prior information attains the best FOSCTTM. Regarding label transfer accuracy, GROTIA ranks second in PBMC-1 and first in PBMC-2. In Figure 3b, each dataset from each domain is shown prior to integration, while Figure 3c shows the integrated dataset, colored by cell type on the left and by domain on the right.

Under the fully unsupervised setting, shown by lighter colors in the left column of Figure 3a (and the right column's horizontal axis for label transfer accuracy), GROTIA demonstrates the strongest overall performance in both FOSCTTM and label transfer accuracy across all four real-world datasets, except for ranking second in FOSCTTM on scGEM. SCOT and scConfluence exhibit comparable results, followed by UnionCom, MMD-MA, and UniPort. Detailed numerical results corresponding to the metric plots are presented in Supplementary Tables A6-A9.

GROTIA Reveals Gene-Specific Contributions and Key Biological Processes in the RNA Embedding

GROTIA provides an in-model measure of gene importance, pinpointing which genes drive variation along each latent dimension. Specifically, we compute partial derivatives of the RBF kernel embeddings with respect to each gene's expression and then weight these by the projection matrix to obtain a contribution score for every gene–dimension pair. Ranking these scores identifies dimension-specific "signature genes"—those whose expression changes most strongly reposition cells in the low-dimensional space. For additional details, see Section .

Figure 4a displays the overall gene importance scores across all eight GROTIA-derived dimensions. Figure 4b presents UMAP visualizations of the top gene expression patterns for Dimensions 1 and 3, illustrating how high-impact genes are distributed across the dataset. For Dimension 1, the top three contributing genes (LYZ, ZEB2, PLXDC2) are highly expressed in monocytes and myeloid cell populations, consistent with further differentiation within the monocyte lineage. Moreover, Dimension 3 emphasizes GNLY, CCL5, and LEF1—genes enriched in NK cells and T cells, indicating a T cell–specific transcriptional program. Notably, GROTIA requires no a priori matching of features across modalities, so these dimension-specific drivers offer an unbiased method to uncover potential marker genes.

To link the top contributors in Dimension 1 to biological processes, we performed Gene Ontology (GO) enrichment analysis, as shown in figure Fig. 4c. These genes were enriched for the terms RNA polymerase II-specific DNA-binding transcription factor binding, β-catenin binding, and peptide binding. The first term suggests that Dimension 1 captures a transcriptional regulatory program active during monocyte and dendritic cell differentiation, involving lineage-defining regulators such as ZEB2, which is es-

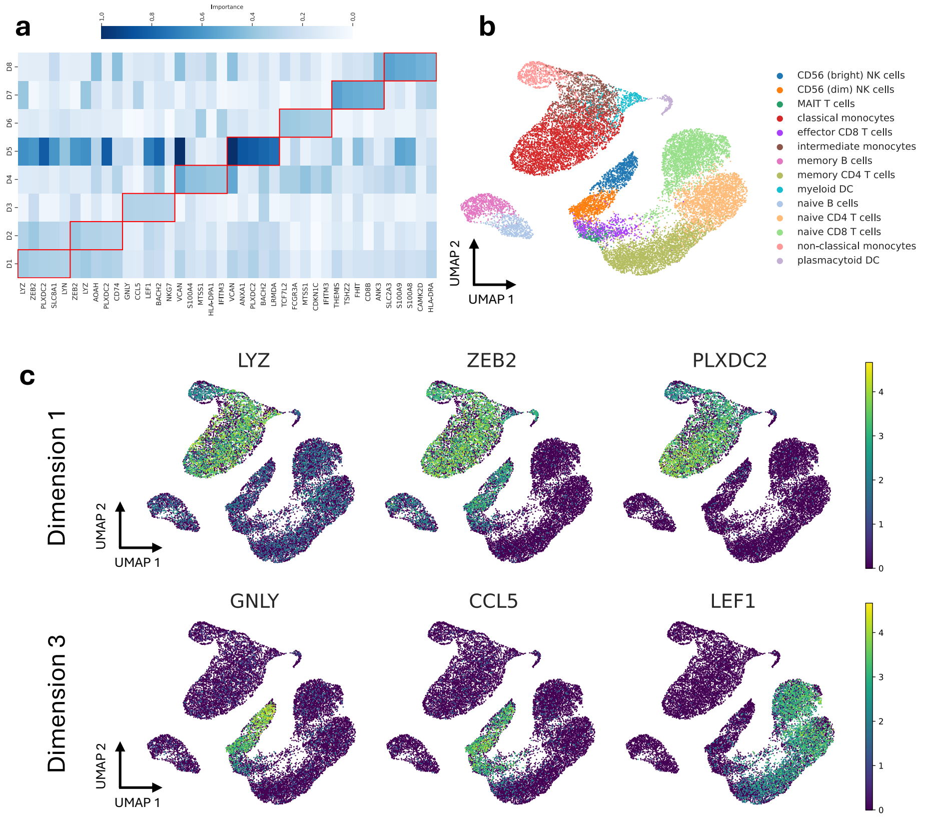

d

Dimension 1

Source	GO name	GO ID	P value	term size	Inter. size
GO:MF	RNA polymerase II-specific DNA-binding transcription factor binding	GO:0061629	2.632×10^{-3}	348	14
GO:MF	beta-catenin binding	GO:0008013	1.076×10^{-2}	89	7
GO:MF	peptide binding	GO:0042277	1.455×10^{-2}	254	11
GO:MF	GTPase regulator activity	GO:0030695	3.364×10^{-2}	495	15
GO:BP	toll-like receptor signaling pathway	GO:0002224	7.559×10^{-3}	75	7
GO:BP	response to peptide hormone	GO:0043434	1.164×10^{-2}	434	15
GO:BP	cell killing	GO:0001906	4.250×10^{-2}	219	10
GO:CC	trans-Golgi network membrane	GO:0032588	1.121×10^{-2}	107	7
GO:CC	collagen-containing extracellular matrix	GO:0062023	2.299×10^{-2}	425	13
GO:CC	actin filament	GO:0005884	2.457×10^{-2}	121	7

Dimension 3

Source	GO name	GO ID	P value	term size	Inter. size
GO:MF	immune receptor activity	GO:0140375	5.973×10^{-6}	145	12
GO:MF	antigen binding	GO:0003823	2.142×10^{-2}	175	9
GO:BP	calcium-mediated signaling	GO:0019722	7.420×10^{-3}	218	11
GO:BP	viral process	GO:0016032	1.169×10^{-2}	427	15
GO:CC	membrane raft	GO:0045121	2.669×10^{-3}	291	12
GO:CC	COPII-coated ER to Golgi transport vesicle	GO:0030134	3.667×10^{-2}	89	6

Figure 4. (a) Heatmap of partial derivative–based importance scores for the top five genes in each of the eight GROTIA-derived dimensions (D1–D8). Darker blue indicates higher importance. The top five genes per dimension are outlined in the red box. (b) UMAP projections of Dimension 1 and Dimension 3, highlighting cell-type annotations (top) and the expression distributions of top-ranked genes (middle and bottom). Warmer hues denote higher expression, revealing distinct cellular subsets for Dimension 1 and Dimension 3. (c) Summaries from the Gene Ontology experiment, showing significant enrichment grouped by Molecular Function (MF), Cellular Component (CC), and Biological Process (BP). Collectively, these panels show how GROTIA's dimension-wise interpretability links high-impact genes to key biological functions.

sential for monocyte and plasmacytoid DC development [25]. The second highest-ranked term, β-catenin binding, indicates involvement of Wnt/β-catenin signaling in monocyte and DC biology; for instance, β-catenin activation fosters a tolerogenic phenotype in DCs [26], while its aberrant stabilization can obstruct normal monocyte–macrophage differentiation [27]. Finally, enrichment for peptide binding aligns with the antigen processing and presentation roles of monocytes and DCs, consistent with elevated HLA-DR expression in intermediate monocytes [28]. For additional UMAP distributional plots of top genes, see Supplementary Figures A.1 and A.2.

In Dimension 3 of the PBMC transcriptional analysis, we observed GO term enrichment related to immune receptor activity, antigen binding, and calcium-mediated signaling. This suggests that Dimension 3 captures variation in lymphocyte receptor expression and signaling. The GO category immune receptor activity is associated primarily with T lymphocytes, which uniquely express the T-cell receptor complex (for example, CD3 subunits and TCR α/β chains) mediating antigen-specific recognition [29]. Moreover, antigen binding reflects the high expression of immunoglobulin genes by B cells, consistent with their exclusive role in producing antigen-specific antibodies [30]. Finally, calcium-mediated signaling highlights a key activation pathway in T cells, where antigen-receptor engagement triggers Ca^{2+} influx through store-operated Ca^{2+} channels to activate downstream effectors such as the calcineurin–NFAT pathway, a process essential for lymphocyte activation [31]. For additional Gene Ontology enrichment results corresponding to the remaining dimensions, see Supplementary Tables A10–A15.

GROTIA Identifies High-Impact Peaks and Regulatory Mechanisms in the ATAC Embedding

Similarly, in the ATAC domain, we ranked open chromatin peaks by their gradient-based contribution scores and performed motif discovery on the highest-impact peaks (see Methods). Mapping each motif to its nearest gene within a 20 kb window revealed putative regulatory relationships linking epigenetic accessibility to transcriptional output. Figure 5a illustrates the procedure for identifying transcription factor–gene pairs from the top-ranked peaks. For further details, please refer to Section .

Figure 5b highlights the top-ranked peaks (based on partial derivative–based importance) across the eight GROTIA-derived dimensions (D1–D8), with the most influential peaks in each dimension outlined in red boxes. Figure 5c shows the inferred transcription factor–gene pairs associated with each dimension. Finally, Figure 5d offers a UMAP projection of these pairs, visually illustrating potential co-expression or repression relationships. In dimension 1, we identified a CEBPB–KLF4 pair. CEBPB is essential for proper monocyte development, including the survival of certain subsets, and likely induces KLF4 as part of the monocyte differentiation network. Indeed, PU.1 (encoded by SPI1) directly upregulates KLF4, and CEBPB cooperates with PU.1 to drive monopoiesis [32]. In dimension 2, the IRF8–CST3 pair emerged. IRF8 directly activates CST3 (cystatin C) during macrophage differentiation, mediated by a unique promoter element that overlaps IRF and ETS sites and requires both IRF8 and an ETS partner (e.g., PU.1) [33]. Dimension 3 highlighted TBX21 (T-bet)–CCL4, wherein T-bet binds to and positively regulates CCL4 in Th1 cells. Genome-wide ChIP-chip experiments in human T cells confirmed CCL4 as a direct T-bet target, revealing T-bet binding in regulatory regions that activate CCL4 transcription [34]. In dimension 5, we found IRF8–CLEC7A. Genome-wide binding studies have identified CLEC7A as an IRF8 target in human myeloid cells [35], and co-expression networks further link CLEC7A with an IRF8-centered module. Consistently, aging human microglia upregulate CLEC7A alongside other "activated" microglial genes under the control of an IRF8/SPI1/RUNX1/TAL1 network [36]. In dimension 6, the SPI1–IFITM3 pair indicates that PU.1 controls a broad antiviral gene program in macrophages, with IFITM3 explicitly citetd as a PU.1-regulated antiviral factor [37]. Dimension 7 highlighted RUNX2–GPR183. Finally, in dimension 8, the PAX5–FAM49A pair was supported by evidence of PAX5 ChIP-seq peaks near FAM49A [38]. Notably, FAM49A is expressed at lower levels in PAX5-positive pro–B cells and is de-repressed in PAX5-deficient cells, indicating that PAX5 normally suppresses Fam49a expression during early B-cell development [39].

GROTIA enables identification of cellular subpopulation on integrated space

Beyond simply projecting scRNA and scATAC profiles into a shared space, GROTIA provides a natural framework for discovering subpopulations in an unsupervised manner. While many datasets come with predefined labels (e.g., annotated cell types), these annotations may be incomplete or coarse, especially when new subtleties or states exist that were not recognized during initial labeling. By clustering cells within GROTIA's integrated representation, we can uncover finer structures and novel cellular states that may otherwise remain masked by legacy annotations.

To determine the optimal number of clusters k, we plot the reconstruction error for various k-values and look for a distinct elbow (Fig. 6a). Beyond this point, further increasing k offers minimal improvement in accuracy while potentially fragmenting biologically coherent groups. We therefore select the k at the bump, yielding a robust trade-off between clustering granularity and data fidelity. We then visualize the resulting assignments alongside the original cell-type labels on a UMAP projection (Fig. 6c). Notably, GROTIA identifies distinct subpopulations that align well with known major cell types, yet can also isolate refined subclusters reflecting subtle transcriptional and epigenetic differences.

Finally, to benchmark the quality of GROTIA's partitioning, we compare against a conventional community detection algorithm (Louvain) using three standard clustering metrics: Adjusted Rand Index (ARI), Normalized Mutual Information (NMI), and Purity (Fig. 6d). Our method achieves comparable or better performance, demonstrating that GROTIA's alignment-based approach not only reconciles multi-omic data but also preserves biologically meaningful structures when clustering.

Discussion

We present GROTIA, a novel algorithm for unsupervised single-cell multi-omics integration that combines optimal transport for global alignment with graph regularization to preserve local structure. Benchmarking against state-of-the-art methods in both unsupervised and semi-supervised settings, GROTIA delivers comparable or superior performance while offering a computationally efficient solution. Critically, our framework also includes an in-model interpretability mechanism, allowing users to identify which genes or peaks drive each dimension of the integrated embedding. This enables targeted downstream analyses—such as Gene Ontology enrichment or motif discovery—to reveal meaningful biological processes in the RNA and ATAC spaces.

Beyond aligning multi-omics data, GROTIA leverages its transport plan for post-integration clustering, offering a data-driven approach to refine or correct misannotations in existing labels. Notably, unlike methods that require shared features across modalities, GROTIA only assumes that cells (rather than individual genes or peaks) follow a similar distribution if they belong to the same type or lineage—thus broadening its applicability to complex datasets.

Looking ahead, we plan to extend GROTIA to time-series multi-omics data, where paired measurements across multiple time points are becoming increasingly common. Furthermore, we will

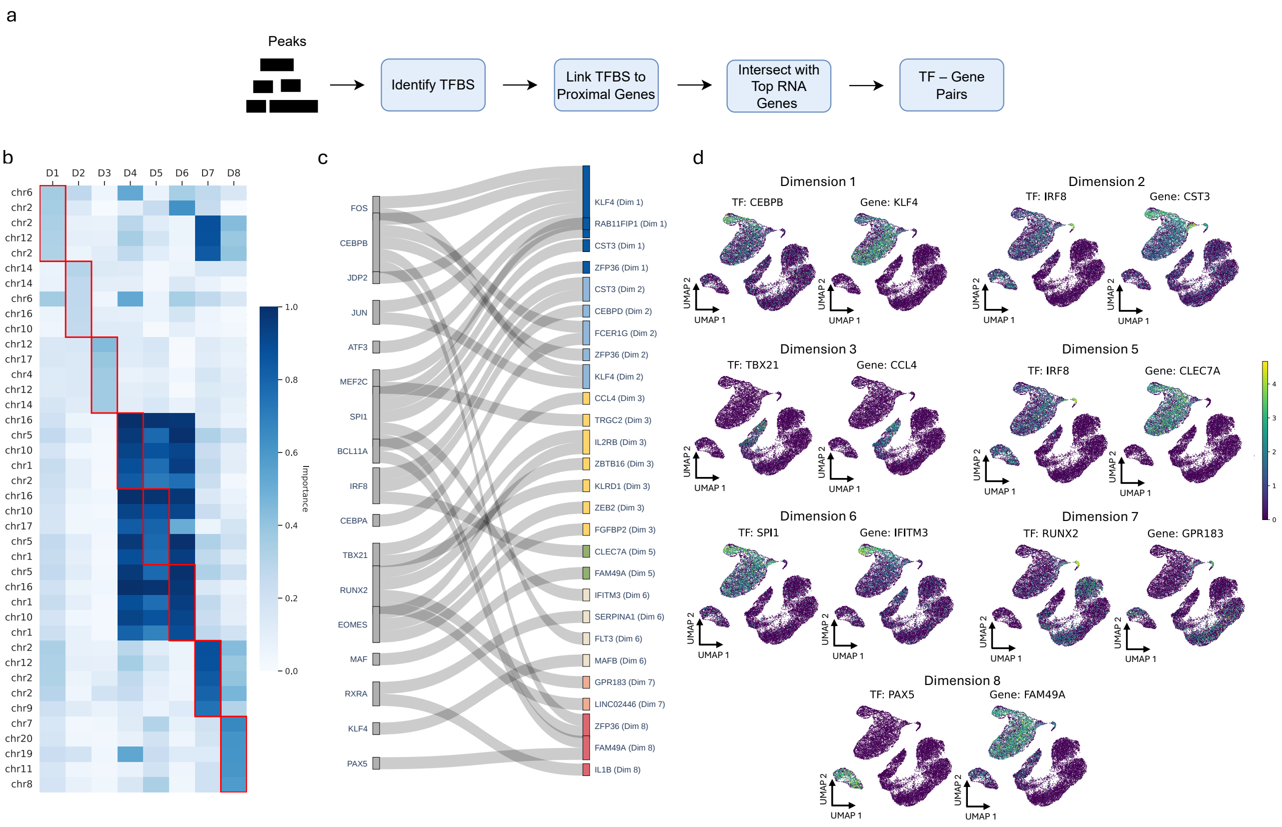

Figure 5. (a) Schematic of the workflow for identifying significant motifs in open chromatin peaks, mapping these motifs to nearby genes, and intersecting them with top RNA space genes to form motif–gene pairs. (b) Heatmap of gradient-based importance scores for the five most influential ATAC peaks in each GROTIA-derived dimension (D1–D8), where darker blue indicates greater importance. (c) Sankey diagram illustrating dimension-specific gene–factor pairs. Genes (left) connect to their putative transcription factors (right), validated through literature. For instance, FOS are implicated as potential regulators of Gene KFL4 in Dimensions 1 and CEBPB as potential regulators of Gene FCR1G in dimension 2. (d) UMAP embeddings colored by accessibility values of selected gene–factor pairs from Dimensions 1, 3, 5, 6, 7, and 8. Warmer hues denote higher accessibility, highlighting dimension-specific regulatory landscapes. Co-expression patterns further support these putative regulatory relationships.

deepen our driver-gene analyses to decode how specific features shape the integrated embedding, with the goal of uncovering more nuanced regulatory processes across different cell states.

Availability of source code and requirements (optional, if code is present)

Lists the following:

- Project name: GROTIA
- Project home page: `https://github.com/PennShenLab/GROTIA`
- Operating system(s): Platform independent
- Programming language: Python 3.8 or higher
- License: License: MIT License

This needs to be under an Open Source Initiative approved license where practicable compiled running software is made available. If the code is not hosted in a repository the *GigaScience* GitHub repository is also available for this purpose.

Data Availability

The GROTIA algorithm is freely available at `https://github.com/PennShenLab/GROTIA`. All data used in this manuscript is publicly available and can be found at Liu et al. [11], Cheow et al. [16], Demetci et al. [12], Chen et al. [17], Cao et al. [14], and Samaran et al. [13].

Competing Interests

The authors declared no potential conflicts of interest with respect to the research, authorship, and/or publication of this article.

List of abbreviations

Fraction of Samples Closer Than the True Match (FOSCTTM); Gene Ontology (GO); Generalized Unsupervised Manifold Alignment (GUMA); GROTIA (Graph-Regularized Optimal Transport Framework for Diagonal Single-Cell Integrative Analysis); Label Transfer Accuracy (LTA); Maximum Mean Discrepancy (MMD); Peripheral Blood Mononuclear Cell (PBMC); Single-Cell Multi-Omics Alignment with Optimal Transport (SCOT); Transcription Factor (TF); Transcription Start Sites (TSS); Unsupervised Topological Alignment for Single-Cell Multi-Omics Integration (UnionCom); Wasserstein-based (WD).

Funding

This work is supported in part by NIH Grants R01 AG071470, U19 AG074879, U01 AG066833 and U01 AG068057.

Author contributions statement

Conceptualization, Z.W., Q.Z., M.K., and L.S.; Methodology, Z.W., Q.Z., and L.S.; Resources, L.S.; Formal analysis, Z.W., Q.Z., S.Y., Z.Z., M.K., T.Z., and L.S.; Writing-Original Draft, Z.W., Q.Z., Z.Z., and

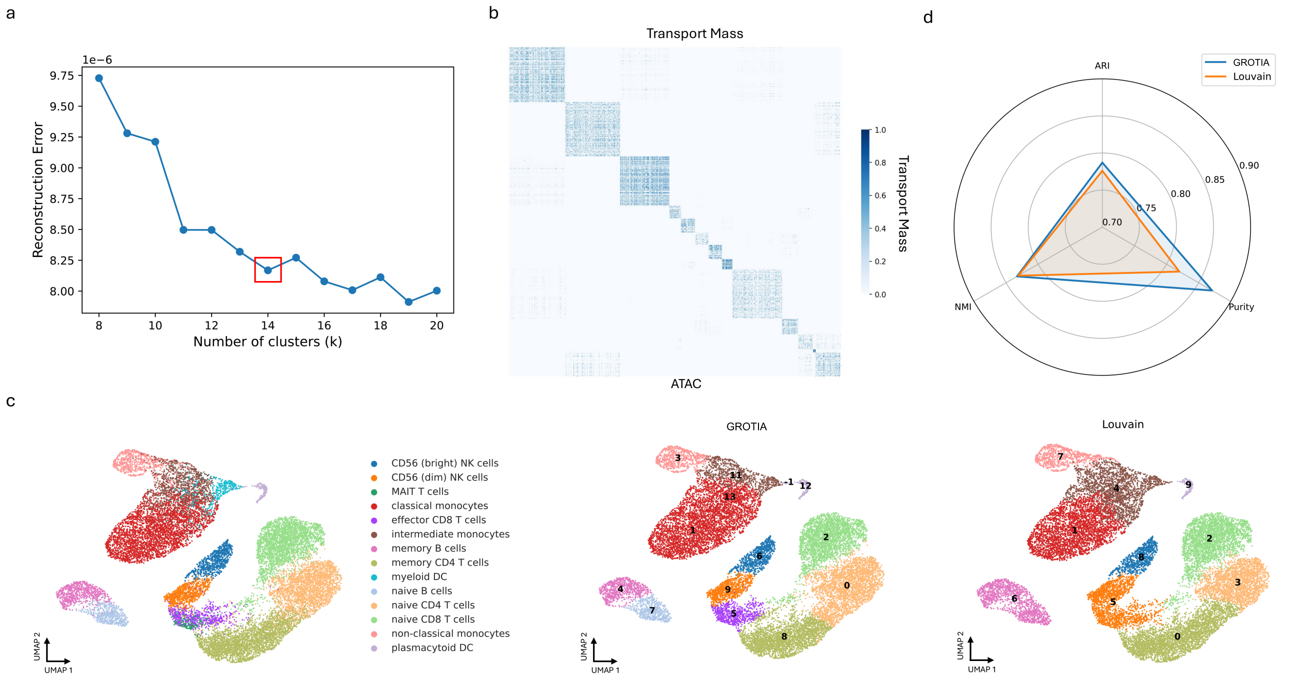

Figure 6. (a) Reconstruction error plotted against the number of clusters k, with the chosen k marked by a red box. This optimal k balances clustering granularity and data fidelity. (b) Heatmap of the transport mass after co-clustering, illustrating how GROTIA aligns cells from scRNA and scATAC. The block-diagonal pattern indicates coherent groupings across both modalities. (c) UMAP projections of the integrated dataset, colored by true cell-type annotations (left), and clustered with GROTIA (center) or Louvain (right). For each method, predicted clusters are labeled by the ground-truth cluster with which they most overlap. GROTIA produces distinct subpopulations consistent with known cell-type boundaries. (d) Radar plot comparing GROTIA (orange) and Louvain (blue) on three clustering metrics (ARI, NMI, and Purity). GROTIA demonstrates higher or comparable performance, indicating its ability to robustly identify meaningful subpopulations.

L.S.; Funding acquisition, L.S.; Writing-Review and Editing, Z.W., Q.Z., S.Y., Z.Z., M.K., T.Z., and L.S.

References

1. Heumos L, Schaar AC, Lance C, Litinetskaya A, Drost F, Zappia L, et al. Best practices for single-cell analysis across modalities. Nature Reviews Genetics 2023;24(8):550–572.
2. Haque A, Engel J, Teichmann SA, Lönnberg T. A practical guide to single-cell RNA-sequencing for biomedical research and clinical applications. Genome medicine 2017;9:1–12.
3. Grandi FC, Modi H, Kampman L, Corces MR. Chromatin accessibility profiling by ATAC-seq. Nature protocols 2022;17(6):1518–1552.
4. Li B, Nabavi S. scGEMOC, A Graph Embedded Contrastive Learning Single-cell Multiomics Clustering Model. In: 2023 IEEE International Conference on Bioinformatics and Biomedicine (BIBM) IEEE; 2023. p. 2075–2080.
5. Huizing GJ, Deutschmann IM, Peyré G, Cantini L. Paired single-cell multi-omics data integration with Mowgli. Nature Communications 2023;14(1):7711.
6. Barkas N, Petukhov V, Nikolaeva D, Lozinsky Y, Demharter S, Khodosevich K, et al. Joint analysis of heterogeneous single-cell RNA-seq dataset collections. Nature methods 2019;16(8):695–698.
7. Halpern KB, Shenhav R, Massalha H, Toth B, Egozi A, Massasa EE, et al. Paired-cell sequencing enables spatial gene expression mapping of liver endothelial cells. Nature biotechnology 2018;36(10):962–970.
8. Amodio M, Krishnaswamy S. MAGAN: Aligning biological manifolds. In: International conference on machine learning PMLR; 2018. p. 215–223.
9. Cui Z, Chang H, Shan S, Chen X. Generalized unsupervised manifold alignment. Advances in Neural Information Processing Systems 2014;27.
10. Cao K, Bai X, Hong Y, Wan L. Unsupervised topological alignment for single-cell multi-omics integration. Bioinformatics 2020;36(Supplement_1):i48–i56.
11. Liu J, Huang Y, Singh R, Vert JP, Noble WS. Jointly embedding multiple single-cell omics measurements. In: Algorithms in bioinformatics:... International Workshop, WABI..., proceedings. WABI (Workshop), vol. 143 NIH Public Access; 2019. .
12. Demetci P, Santorella R, Sandstede B, Noble WS, Singh R. SCOT: single-cell multi-omics alignment with optimal transport. Journal of computational biology 2022;29(1):3–18.
13. Samaran J, Peyré G, Cantini L. scConfluence: single-cell diagonal integration with regularized Inverse Optimal Transport on weakly connected features. Nature Communications 2024;15(1):7762.
14. Cao K, Gong Q, Hong Y, Wan L. A unified computational framework for single-cell data integration with optimal transport. Nature Communications 2022;13(1):7419.
15. Zappia L, Phipson B, Oshlack A. Splatter: simulation of single-cell RNA sequencing data. Genome biology 2017;18(1):174.
16. Cheow LF, Courtois ET, Tan Y, Viswanathan R, Xing Q, Tan RZ, et al. Single-cell multimodal profiling reveals cellular epigenetic heterogeneity. Nature methods 2016;13(10):833–836.
17. Chen S, Lake BB, Zhang K. High-throughput sequencing of the transcriptome and chromatin accessibility in the same cell. Nature biotechnology 2019;37(12):1452–1457.
18. Bravo González-Blas C, Minnoye L, Papasokrati D, Aibar S, Hulselmans G, Christiaens V, et al. cisTopic: cis-regulatory topic modeling on single-cell ATAC-seq data. Nature methods 2019;16(5):397–400.
19. Feydy J, Séjourné T, Vialard FX, Amari Si, Trouvé A, Peyré G. Interpolating between optimal transport and mmd using sinkhorn divergences. In: The 22nd International Conference on Artificial Intelligence and Statistics PMLR; 2019. p. 2681–2690.
20. Kolberg L, Raudvere U, Kuzmin I, Adler P, Vilo J, Peterson H. g:

Profiler—interoperable web service for functional enrichment analysis and gene identifier mapping (2023 update). Nucleic acids research 2023;51(W1):W207–W212.
21. Bruse N, Heeringen SJv. GimmeMotifs: an analysis framework for transcription factor motif analysis. BioRxiv 2018;p. 474403.
22. Kan M, Diwadkar AR, Shuai H, Joo J, Wang AL, Ong MS, et al. Multiomics analysis identifies BIRC3 as a novel glucocorticoid response–associated gene. Journal of Allergy and Clinical Immunology 2022;149(6):1981–1991.
23. Diwadkar AR, Kan M, Himes BE. Facilitating analysis of publicly available ChIP-Seq data for integrative studies. In: AMIA Annual Symposium Proceedings, vol. 2019; 2020. p. 371.
24. Role F, Morbieu S, Nadif M. Coclust: a python package for co-clustering. Journal of Statistical Software 2019;88:1–29.
25. Wu X, Briseño CG, Grajales-Reyes GE, Haldar M, Iwata A, Kretzer NM, et al. Transcription factor Zeb2 regulates commitment to plasmacytoid dendritic cell and monocyte fate. Proceedings of the National Academy of Sciences 2016;113(51):14775–14780.
26. Swafford D, Manicassamy S. Wnt signaling in dendritic cells: its role in regulation of immunity and tolerance. Discovery medicine 2015;19(105):303.
27. Sheng Y, Ju W, Huang Y, Li J, Ozer H, Qiao X, et al. Activation of wnt/β-catenin signaling blocks monocyte–macrophage differentiation through antagonizing PU. 1-targeted gene transcription. Leukemia 2016;30(10):2106–2109.
28. Lee J, Tam H, Adler L, Ilstad-Minnihan A, Macaubas C, Mellins ED. The MHC class II antigen presentation pathway in human monocytes differs by subset and is regulated by cytokines. PloS one 2017;12(8):e0183594.
29. Jones N, Vincent EE, Cronin JG, Panetti S, Chambers M, Holm SR, et al. Akt and STAT5 mediate naïve human CD4+ T-cell early metabolic response to TCR stimulation. Nature Communications 2019;10(1):2042.
30. Li K, Zhang C, Zhou R, Cheng M, Ling R, Xiong G, et al. Single cell analysis unveils B cell-dominated immune subtypes in HNSCC for enhanced prognostic and therapeutic stratification. International Journal of Oral Science 2024;16(1):29.
31. Vig M, Kinet JP. Calcium signaling in immune cells. Nature immunology 2009;10(1):21–27.
32. Feinberg MW, Wara AK, Cao Z, Lebedeva MA, Rosenbauer F, Iwasaki H, et al. The Kruppel-like factor KLF4 is a critical regulator of monocyte differentiation. The EMBO journal 2007;26(18):4138–4148.
33. Tamura T, Thotakura P, Tanaka TS, Ko MS, Ozato K. Identification of target genes and a unique cis element regulated by IRF-8 in developing macrophages. Blood 2005;106(6):1938–1947.
34. Jenner RG, Townsend MJ, Jackson I, Sun K, Bouwman RD, Young RA, et al. The transcription factors T-bet and GATA-3 control alternative pathways of T-cell differentiation through a shared set of target genes. Proceedings of the National Academy of Sciences 2009;106(42):17876–17881.
35. Diamant I, Clarke DJ, Evangelista JE, Lingam N, Ma'ayan A. Harmonizome 3.0: integrated knowledge about genes and proteins from diverse multi-omics resources. Nucleic Acids Research 2025;53(D1):D1016–D1028.
36. Yeh H, Ikezu T. Transcriptional and epigenetic regulation of microglia in health and disease. Trends in molecular medicine 2019;25(2):96–111.
37. Virgilio MC, Ramnani B, Chen T, Disbennett WM, Lubow J, Welch JD, et al. HIV-1 Vpr combats the PU. 1-driven antiviral response in primary human macrophages. Nature Communications 2024;15(1):5514.
38. Stelzer G, Rosen N, Plaschkes I, Zimmerman S, Twik M, Fishilevich S, et al. The GeneCards suite: from gene data mining to disease genome sequence analyses. Current protocols in bioinformatics 2016;54(1):1–30.
39. Silberstein L, Goncalves KA, Kharchenko PV, Turcotte R, Kfoury Y, Mercier F, et al. Proximity-based differential single-cell analysis of the niche to identify stem/progenitor cell regulators. Cell stem cell 2016;19(4):530–543.

Appendix

Table A1. Comparison of runtime performance (minutes) for benchmarked methods using the PBMC dataset (9,378 cells).

**Method**	GROTIA	UniPort	scConfluence	Unioncom	SCOT	MMD-MA
**Runtime (min)**	15	17	20.44	65	185	470

Table A2. Alignment performance by FOSCTTM under unsupervised setting (First 4 columns: Simulation 1, Simulation 2, Simulation 3, Synthetic RNA-seq).

	Simulation 1	Simulation 2	Simulation 3	Synthetic RNA-seq
SCOT	0.088	0.025	**0.009**	0.001
MMD-MA	0.125	0.012	0.739	0.384
UnionCom	0.091	0.028	0.684	0.028
Uniport	0.632	0.313	0.426	0.495
Scconfluence	0.512	0.501	0.437	0.431
GROTIA(Ours)	**0.077**	**0.008**	**0.009**	**5e-5**

Table A3. Alignment performance by FOSCTTM under unsupervised setting (Last 2 columns: scGEM and SNAREseq).

	scGEM	SNAREseq	pbmc10X	pmbc
SCOT	**0.209**	0.218	0.072	0.142
MMD-MA	0.437	0.473	0.273	0.376
UnionCom	0.691	0.510	0.412	0.480
Uniport	0.412	0.216	0.566	0.487
Scconfluence(Diagonal)	0.474	0.418	0.372	0.503
Scconfluence(Prior)	-	-	0.067	0.110
GROTIA(Ours)	0.215	**0.216**	**0.049**	**0.104**

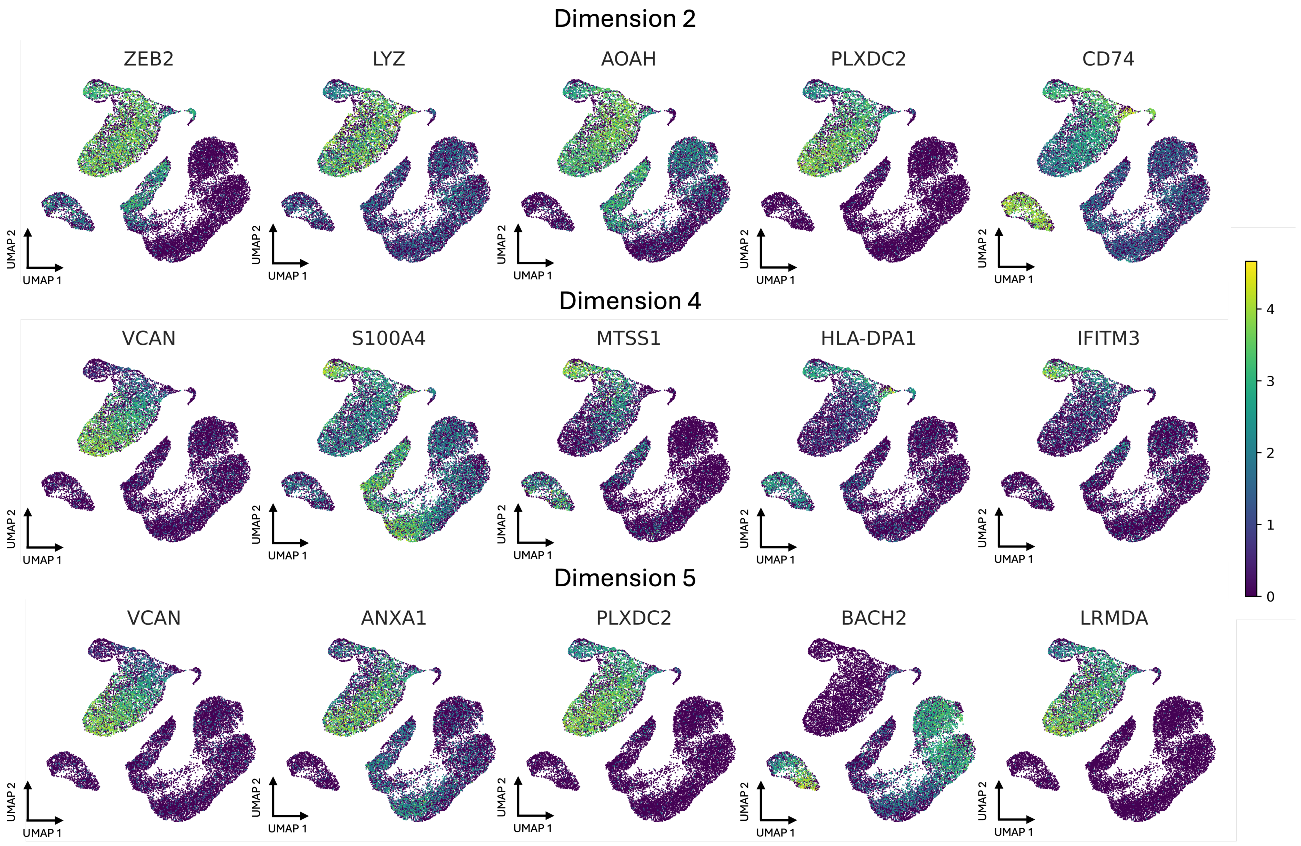

Figure A.1. UMAP Plot of Top Genes for Dimensions 2, 4, and 5.

Table A4. Alignment performance by label transfer accuracy ($k = 5$) under unsupervised setting (First 4 columns: Simulation 1, Simulation 2, Simulation 3, Synthetic RNA-seq).

	Simulation 1	Simulation 2	Simulation 3	Synthetic RNA-seq
SCOT	**0.977**	0.977	**0.950**	**0.996**
MMD-MA	0.897	0.957	0.700	0.506
UnionCom	0.947	0.947	0.133	0.948
Uniport	0.36	0.577	0.35	0.433
Scconfluence	0.493	0.523	0.490	0.650
GROTIA(Ours)	0.967	**0.980**	**0.950**	**0.996**

Table A5. Alignment performance by label transfer accuracy ($k = 5$) under unsupervised setting (Last 2 columns: scGEM and SNAREseq).

	scGEM	SNAREseq	pbmc10X	pmbc
SCOT	0.423	0.852	0.894	0.722
MMD-MA	0.237	0.412	0.224	0.161
UnionCom	0.107	0.288	0.164	0.266
Uniport	0.260	0.705	0.123	0.271
Scconfluence(Diagonal)	0.305	0.590	0.132	0.155
Scconfluence(Prior)	-	-	0.891	0.735
GROTIA(Ours)	**0.588**	**0.947**	**0.897**	**0.802**

Table A6. Alignment performance by FOSCTTM (The lower the better) under semi-supervised setting for the first four datasets.

	Simulation 1	Simulation 2	Simulation 3	Synthetic RNA-seq
SCOT	0.070	0.022	**0.009**	0.001
MMD-MA	0.124	0.023	0.012	0.112
UnionCom	0.083	0.016	0.152	0.038
Uniport	0.520	0.313	0.426	0.485
Scconfluence	0.077	0.007	0.407	0.228
GROTIA(Ours)	**0.069**	**0.005**	**0.009**	**1e-6**

Table A7. Alignment performance by FOSCTTM (The lower the better) under semi-supervised setting for scGEM, SNAREseq, pbmc10X, and pbmc.

	scGEM	SNAREseq	pbmc10X	pbmc
SCOT	**0.192**	0.150	0.073	0.142
MMD-MA	0.201	0.150	0.277	0.374
UnionCom	0.209	0.265	**0.017**	0.258
Uniport	0.259	0.220	0.566	0.487
Scconfluence (Diagnola)	0.234	0.154	0.080	0.211
Scconfluence(Prior)	–	–	0.067	**0.101**
GROTIA(Ours)	0.213	**0.148**	0.045	0.113

Table A8. Alignment performance by label transfer accuracy ($k = 5$) (The higher the better) under semi-supervised setting for the first four datasets.

	Simulation 1	Simulation 2	Simulation 3	Synthetic RNA-seq
SCOT	0.937	0.977	**0.957**	**0.998**
MMD-MA	0.890	0.783	0.947	0.706
UnionCom	0.960	0.620	0.613	0.997
Uniport	0.360	0.577	0.350	0.442
Scconfluence	0.960	0.990	0.580	0.997
GROTIA(Ours)	**0.963**	**0.993**	0.950	**0.998**

Table A9. Alignment performance by label transfer accuracy ($k = 5$) (The higher the better) under semi-supervised setting for scGEM, SNAREseq, pbmc10X, and pbmc.

	scGEM	SNAREseq	pbmc10X	pbmc
SCOT	0.576	0.982	0.894	0.722
MMD-MA	0.588	0.942	0.357	0.258
UnionCom	0.582	0.423	**0.926**	0.343
Uniport	0.412	0.719	0.123	0.276
Scconfluence(Diagonal)	0.621	0.982	0.786	0.574
Scconfluence(Prior)	–	–	0.891	0.747
GROTIA(Ours)	**0.700**	**0.986**	0.922	**0.805**

Table A10. Gene Ontology enrichment analysis for Dimension 2

source	GO name	GO ID	p_value	term size	inter. size
GO:MF	calcium-dependent protein binding	GO:0048306	$4.566e-4$	80	8
GO:MF	GTPase regulator activity	GO:0030695	$2.350e-3$	495	17
GO:BP	toll-like receptor signaling pathway	GO:0002224	$8.450e-3$	75	7
GO:BP	[l]positive regulation of NF-kappaB				
transcription factor activity	GO:0051092	$2.748e-2$	124	8	
GO:BP	receptor internalization	GO:0031623	$3.464e-2$	128	8
GO:CC	membrane raft	GO:0045121	$4.975e-4$	291	13
GO:CC	trans-Golgi network membrane	GO:0032588	$1.304e-3$	107	8
GO:CC	collagen-containing extracellular matrix	GO:0062023	$2.733e-2$	425	13
GO:CC	actin filament	GO:0005884	$2.747e-2$	121	7
GO:CC	cell leading edge	GO:0031252	$3.002e-2$	429	13

Table A11. Gene Ontology enrichment analysis for Dimension 4

source	GO name	GO ID	p_value	term size	inter. size
GO:MF	immune receptor activity	GO:0140375	$6.904e-6$	145	12
GO:BP	[l]positive regulation of protein-				
containing complex assembly	GO:0031334	$3.732e-3$	199	11	
GO:BP	cell killing	GO:0001906	$9.412e-3$	219	11
GO:BP	ruffle organization	GO:0031529	$2.118e-2$	56	6
GO:CC	[l]COPII-coated ER to Golgi				
transport vesicle	GO:0030134	$2.685e-5$	89	9	
GO:CC	ruffle	GO:0001726	$1.041e-2$	182	9
GO:CC	focal adhesion	GO:0005925	$2.953e-2$	423	13

Table A12. Gene Ontology enrichment analysis for Dimension 5

source	GO name	GO ID	p_value	term size	inter. size
GO:MF	GTPase regulator activity	GO:0030695	$2.006e-5$	495	20
GO:MF	phospholipid binding	GO:0005543	$1.540e-3$	484	17
GO:BP	[l]negative regulation of				
protein phosphorylation	GO:0001933	$1.810e-4$	270	14	
GO:BP	[l]positive regulation of NF-kappaB				
transcription factor activity	GO:0051092	$2.258e-2$	124	8	
GO:BP	regulation of GTPase activity	GO:0043087	$2.717e-2$	210	10

Table A13. Gene Ontology enrichment analysis for Dimension 6

source	GO name	GO ID	p_value	term size	inter. size
GO:MF	[l]DNA-binding transcription				
factor binding	GO:0140297	$3.481e-2$	490	15	
GO:MF	immune receptor activity	GO:0140375	$3.850e-2$	145	8
GO:BP	phagocytosis	GO:0006909	$6.492e-6$	234	15
GO:BP	cellular response to metal ion	GO:0071248	$5.224e-4$	200	12
GO:BP	icosanoid biosynthetic process	GO:0046456	$1.485e-3$	57	7
GO:BP	cell killing	GO:0001906	$9.412e-3$	219	11
GO:BP	[l]positive regulation of				
interleukin-1 beta production	GO:0032731	$3.171e-2$	60	6	

Table A14. Gene Ontology enrichment analysis for Dimension 7

source	GO name	GO ID	p_value	term size	inter. size
GO:MF	immune receptor activity	GO:0140375	$3.729e-5$	145	11
GO:BP	cell-matrix adhesion	GO:0007160	$4.671e-2$	236	10
GO:CC	T cell receptor complex	GO:0042101	$4.214e-3$	136	8

Table A15. Gene Ontology enrichment analysis for Dimension 8

source	GO name	GO ID	p_value	term size	inter. size
GO:MF	immune receptor activity	GO:0140375	$6.680e-5$	145	11
GO:BP	cell killing	GO:0001906	$1.217e-4$	219	13
GO:BP	phagocytosis	GO:0006909	$1.248e-2$	234	11
GO:BP	[l]regulation of metal ion transport	GO:0010959	$4.048e-2$	369	13

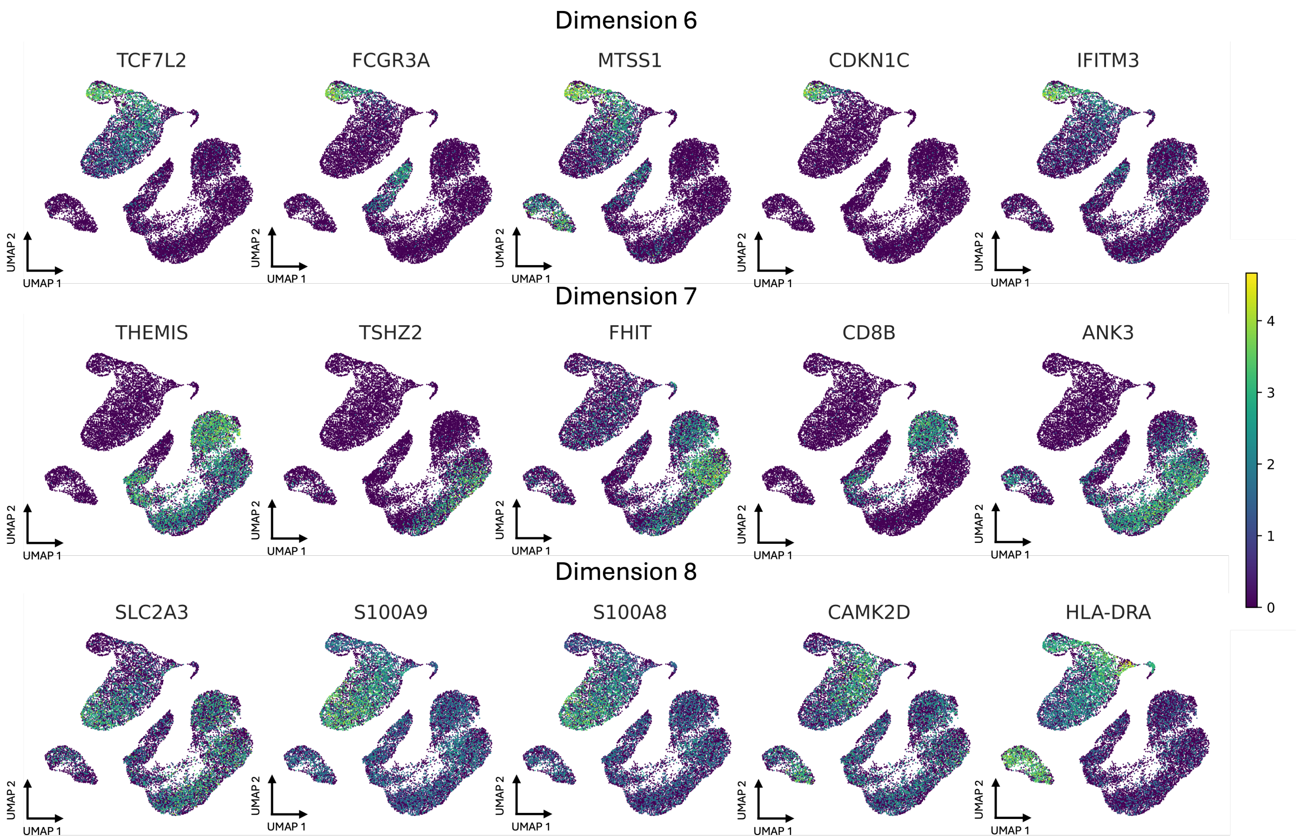

Figure A.2. UMAP Plot of Top Genes for Dimensions 6, 7, and 8.

Click here to access/download
Supplementary Material
Supplementary.pdf

Cover Letter Click here to access/download;Personal Cover;Cover_Letter.docx

Jun 10, 2025

Dear Respected Editors,

I am pleased to submit our manuscript entitled "**An Interpretable Graph-Regularized Optimal Transport Framework for Diagonal Single-Cell Integrative Analysis**" for consideration in ***GigaScience***. This manuscript represents original work conducted by our research team and presents a novel approach to addressing the challenges associated with multi-omics integration in single cells.

Recent advancements in single-cell omics technologies have enabled detailed characterization of cellular processes. However, co-assay sequencing technologies remain limited, resulting in unpaired single-cell omics datasets with differing feature dimensions. Most state-of-the-art methods overcome this by leveraging biological priors—for instance, mapping chromatin peaks to nearby gene promoters to infer gene activity—so all modalities can be projected into a shared feature space. However, these approaches assume that most features can be reliably connected across modalities. In our submission, we introduce GROTIA, an interpretable framework for aligning multi-omics datasets without requiring feature correspondence.

GROTIA uses optimal transport for global alignment across multiple omics modalities while preserving local relationships through graph regularization. It also offers interpretability by computing domain-specific feature importance via partial derivatives, addressing a gap in current methods that often lack interpretability. Moreover, the transport plan between modalities can be leveraged for post-integration clustering, enabling a data-driven approach to discover novel cell subpopulations. We demonstrate that GROTIA outperforms state-of-the-art unsupervised alignment methods on four simulated and four real-world datasets. We also validate the biological relevance of its top-ranked features in each domain.

The innovative approach presented in our work has bridged the gap in single-cell multi-omics by enabling unpaired data integration without feature correspondence. Its in-model interpretability clarifies how key features drive cross-modal alignment. We believe these contributions align well with the scope of ***GigaScience***

Thank you for your time and consideration.

Sincerely,

Li Shen, Ph.D., FAIMBE, FACMI
Professor of Informatics and Radiology
Perelman School of Medicine, University of Pennsylvania

B306 Richards Building, 3700 Hamilton Walk • Philadelphia, PA 19104-6021 •
Li.Shen@pennmedicine.upenn.edu

Graphical Abstract

Click here to access/download;Graphical Abstract;Graphical Abstract.pdf

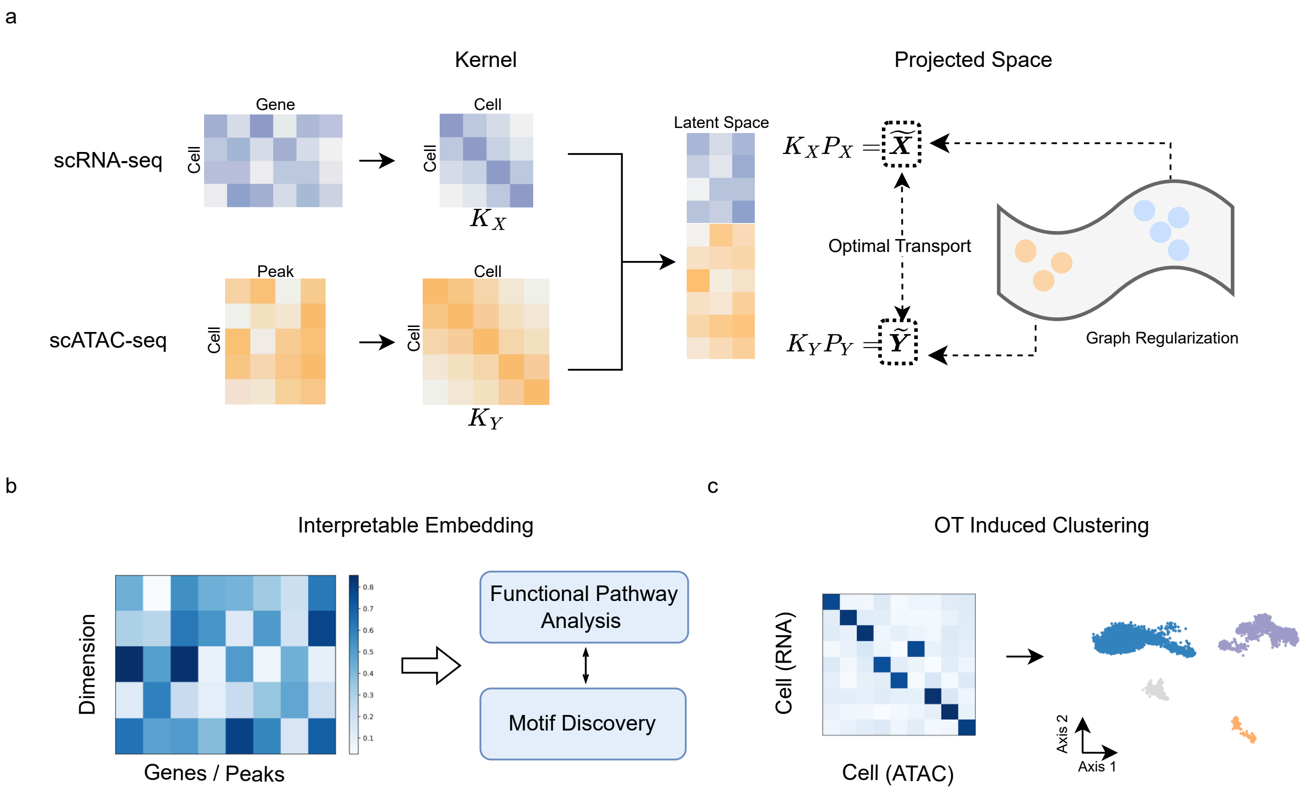

Supplement: giag012_GIGA-D-25-00229_original_submission [file giag012_giga-d-25-00229_original_submission.pdf]
