# Supplementary material for: An interpretable Graph-Regularized Optimal Transport Framework for Diagonal Single-Cell Integrative Analysis
Source: Gigascience. 2026 Feb 9;15:giag012. doi: 10.1093/gigascience/giag012 (PMC12970605; doi:10.1093/gigascience/giag012)

# GigaScience


## An Interpretable Graph-Regularized Optimal Transport Framework for Diagonal Single-Cell Integrative Analysis

--Manuscript Draft--

| | |
|---|---|
| **Manuscript Number:** | GIGA-D-25-00229R1 |
| **Full Title:** | An Interpretable Graph-Regularized Optimal Transport Framework for Diagonal Single-Cell Integrative Analysis |
| **Article Type:** | Technical Note |
| **Funding Information:** | National Institutes of Health (R01 AG071470) — Dr. Li Shen<br>National Institutes of Health (U19 AG074879) — Dr. Li Shen<br>National Institutes of Health (U01 AG066833) — Dr. Li Shen<br>National Institutes of Health (U01 AG068057) — Dr. Li Shen |
| **Abstract:** | Background: Recent advancements in single-cell omics technologies have enabled detailed characterization of cellular processes. However, coassay sequencing technologies remain limited, resulting in un-paired single-cell omics datasets with differing feature dimensions; Finding: we present GROTIA (Graph-Regularized Optimal Transport Framework for Diagonal Single-Cell Integrative Analysis), a computational method to align multi-omics datasets without requiring any prior correspondence information. GROTIA achieves global alignment through optimal transport while preserving local relationships via graph regularization. Additionally, our approach provides interpretability by deriving domain-specific feature importance from partial derivatives, highlighting key biological markers. Moreover, the transport plan between modalities can be leveraged for post-integration clustering, enabling a data-driven approach to discover novel cell subpopulations; Conclusions: We demonstrate GROTIA's superior performance on four simulated and four real-world datasets, surpassing state-of-the-art unsupervised alignment methods and confirming the biological significance of the top features identified in each domain. The software is available at https://github.com/PennShenLab/GROTIA. |
| **Corresponding Author:** | Li Shen, PhD<br>University of Pennsylvania<br>Philadelphia, PA UNITED STATES |
| **Corresponding Author Secondary Information:** | |
| **Corresponding Author's Institution:** | University of Pennsylvania |
| **Corresponding Author's Secondary Institution:** | |
| **First Author:** | Zexuan Wang |
| **First Author Secondary Information:** | |
| **Order of Authors:** | Zexuan Wang<br>Qipeng Zhan<br>Shu Yang<br>Zhuoping Zhou<br>Mengyuan Kan<br>Tianhua Zhai<br>Li Shen |
| **Order of Authors Secondary Information:** | |

*Powered by Editorial Manager® and ProduXion Manager® from Aries Systems Corporation*

| | |
|---|---|
| **Response to Reviewers:** | We sincerely appreciate the editors and reviewers for their valuable comments, which have significantly improved the quality of our manuscript. All comments have been thoughtfully addressed in the revised manuscript. To aid the reviewing process, we have highlighted the revised parts in blue and provided detailed point-by-point responses below.<br><br>************************************<br>Reviewers' Comments to Author:<br>************************************<br>Reviewer: 1<br>************************************<br><br>Comments:<br><br>The manuscript presents a well-motivated and technically elegant approach to diagonal single-cell data integration, combining optimal transport with graph-based regularization to achieve a balance between global and local structure alignment. The method addresses an important challenge in single-cell data integration, where existing approaches still leave room for improvement. Its embedding design offers the potential for interpretable feature-level insights, a particularly desirable quality in single-cell multi-omics integration where biological interpretability is especially important.<br><br>Response:<br><br>Thank you for this positive and encouraging feedback. We appreciate the reviewer's recognition of the motivation, technical design, and interpretability of our approach, and we are glad that the balance between global and local structure alignment, as well as the potential for feature-level biological insights, is viewed as a meaningful contribution to single-cell multi-omics integration.<br><br>—----------------------------------------<br><br>Comments:<br><br>That said, the manuscript would be substantially strengthened by deeper validation and a clearer demonstration of reproducibility. Some claims would benefit from stronger empirical support in the presented results, and a more thorough evaluation of the method's added value relative to unimodal alternatives, particularly in the context of marker gene discovery and the identification of cell types or subpopulations, could further enhance the manuscript. Additionally, the impact of key parameter choices, such as kernel bandwidth selection, the number of nearest neighbors (k), and sensitivity to hyperparameters (λ, ρ), should be more fully explored, reported, or justified. Reproducibility could be improved by providing scripts and a computational environment or container to replicate all analyses and figures presented in the manuscript. Usability would also be improved by providing the method as an installable Python package, rather than limiting implementation to a Jupyter Notebook.<br><br>Response:<br><br>Thank you for these constructive suggestions. We agree that stronger validation, clearer reproducibility, and improved usability would further strengthen the manuscript. Many of the points raised here (e.g., additional empirical support, comparison to unimodal alternatives for marker discovery and subpopulation identification, sensitivity to key parameters such as kernel bandwidth, k, and hyperparameters including λ and ρ, and improvements to code organization, environment specification, and packaging) overlap with the reviewer's detailed comments. We therefore address each item step by step in our responses below, and have revised the manuscript and repository accordingly to improve clarity, transparency, and reproducibility.<br><br>—----------------------------------------<br><br>Comments:<br><br>Overall, the manuscript introduces a compelling methodological framework with |

meaningful potential for applications in single-cell integration. The suggestions that follow are intended to help the authors strengthen their contribution in alignment with GigaScience's emphasis on openness, reproducibility, and FAIR principles. I hope these suggestions will help strengthen the support for the authors' conclusions, clarify the reasoning behind key arguments, and improve the clarity and interpretability of the figures and descriptions.

Response:

Thank you for this constructive feedback. We appreciate the reviewer's emphasis on openness, reproducibility, and clarity, and have revised the manuscript and supporting materials accordingly to strengthen the presentation and better support our conclusions.

—----------------------------------------

Detailed Comments:

1. Reproducibility

Reproducibility is impeded by the absence of clearly organized scripts or workflow files to regenerate the results, figures, and tables presented in the manuscript. While some outputs are shown or alluded to in the Jupyter Notebook found in the linked GitHub repository, they are not clearly cross-referenced with the paper's results, making it difficult to confirm how specific figures or tables were produced. Furthermore, no computational environment specification is provided, which makes replication with confidence impossible. Certain aspects of the manuscript fall short of best practices for transparent and reproducible research. Analysis scripts are incomplete or undocumented, and key portions of the software pipeline are either insufficiently described or lack proper attribution. These limitations hinder reproducibility and reduce reusability. Figures would also benefit from clearer annotation. Collectively, these shortcomings detract from alignment with the FAIR principles emphasized by GigaScience. Reproducibility would be significantly improved by packaging the software, versioning the code, defining and documenting the computational environment, and depositing all components of the analysis pipeline, including preprocessing scripts, evaluation code, and figure generation, in a publicly accessible repository.

Response:

We thank the reviewer for their comments regarding reproducibility and would like to clarify one important point. Although the code is distributed in Jupyter Notebook format, all experiments were designed and executed in Google Colab, rather than in a locally configured Jupyter environment. Google Colab provides a standardized and stable execution environment, which substantially reduces system-dependent variability and supports reproducibility.

In response to the reviewer's comments, we have taken several concrete steps to strengthen reproducibility and reusability; all corresponding code updates are available at https://github.com/PennShenLab/GROTIA :
- We now provide an explicit environment.txt file specifying all required packages and versions.
- We include fully documented and self-contained notebooks implementing our integration method, covering interpretable embeddings for both RNA and ATAC modalities as well as OT-based co-clustering.
- We include scripts for running all baseline methods used in the manuscript to facilitate fair comparison and independent verification.
We have added detailed explanations for each function within the notebooks, complemented by in-code documentation comments.

—----------------------------------------

Comments:

Powered by Editorial Manager® and ProduXion Manager® from Aries Systems Corporation

Additionally, while it is generally clear how the data were collected and curated, the rationale for using preprocessed datasets, particularly those sourced from external repositories, could be more clearly explained. The data are shared via a Google Drive link provided in the GitHub repository, which is convenient, though it may benefit from a more transparent and persistent form of distribution. The manuscript states that "All data used in this manuscript is publicly available and can be found at Liu et al. [11], Cheow et al. [16], Demetci et al. [12], Chen et al. [17], Cao et al. [14], and Samaran et al. [13].", but it appears that preprocessed versions of these datasets were used, rather than the original raw data. Clarifying this point would help improve transparency and reproducibility.

Response:

We clarify that all datasets were used exactly as released by the original benchmarked integration methods to ensure fair and consistent comparison, and no customized or alternative preprocessing pipelines were introduced in this work.

Specifically, datasets from Liu et al. [11], Cheow et al. [16], Demetci et al. [12], and Chen et al. [17] are only publicly available in preprocessed form as provided by the original authors, and we use these versions without modification. In contrast, datasets from Cao et al. [14] and Samaran et al. [13] are released as raw data, and we preprocess them strictly following the procedures described by the original authors, using the same steps and parameters as documented in their tutorials.

This is now explicitly stated in the Training Details section:

“The input to GROTIA follows standard dataset preprocessing practices and does not require method-specific processing. For all simulated datasets, features were z-score normalized prior to alignment, following the procedure used in \citet{liu2019jointly}. For the scGEM and SNARE-seq datasets, we downloaded the preprocessed datasets provided by \citet{demetci2022scot} and applied the same unit-normalization procedure described in that work. For the PBMC-1 and PBMC-2 datasets, we strictly followed the preprocessing pipelines used by the original authors of the corresponding benchmarked methods \citep{samaran2024scconfluence,cao2022unified}. Specifically, we downloaded the raw data and applied the preprocessing scripts provided by the respective authors. Consequently, all datasets were used exactly as released by the original benchmarked integration methods to ensure fair and consistent comparison, and no customized or alternative preprocessing pipelines were introduced in this work.”

—---------------------------------------

Comments:

The manuscript also describes custom preprocessing procedures for scRNA-seq and scATAC-seq data, including PCA, TF-IDF normalization, and gene filtering, that appear inconsistent with the properties of the datasets used. Without access to preprocessing scripts or further clarification, it is unclear whether these procedures were performed as described. Clarifying these discrepancies would strengthen transparency and ensure fair benchmarking comparisons. In addition, to improve transparency and reproducibility, it would be helpful to provide the scripts or commands used to run these baseline methods, along with the evaluation code for computing the reported metrics.

Response:

We clarify that the preprocessing steps described in the manuscript for scRNA-seq and scATAC-seq data—including PCA, TF-IDF normalization, and gene filtering—are not custom procedures introduced by our work, but instead strictly follow the preprocessing pipelines used by the original authors of the corresponding benchmarked methods

This is now explicitly stated in the Training Details section (same as previous response):

“The input to GROTIA follows standard dataset preprocessing practices and does not

require method-specific processing. For all simulated datasets, features were z-score normalized prior to alignment, following the procedure used in \citet{liu2019jointly}. For the scGEM and SNARE-seq datasets, we downloaded the preprocessed datasets provided by \citet{demetci2022scot} and applied the same unit-normalization procedure described in that work. For the PBMC-1 and PBMC-2 datasets, we strictly followed the preprocessing pipelines used by the original authors of the corresponding benchmarked methods \citep{samaran2024scconfluence,cao2022unified}. Specifically, we downloaded the raw data and applied the preprocessing scripts provided by the respective authors. Consequently, all datasets were used exactly as released by the original benchmarked integration methods to ensure fair and consistent comparison, and no customized or alternative preprocessing pipelines were introduced in this work."

—---------------------------------------

Comments:

Finally, several methodological details underlying downstream analyses are insufficiently described to allow confident reproduction or interpretation. For instance, it is unclear which dataset was used to obtain the results in "GROTIA Reveals Gene-Specific Contributions and Key Biological Processes in the RNA Embedding" and Figure 4 and 5. Additionally, the motif discovery step using GimmeMotifs should be expanded, since it is currently not entirely clear how motifs were matched to known transcription factors, and the process described in the text does not fully align with what is shown in Figure 5A. Clarifying these points would help improve the reproducibility and interpretability of the manuscript's key biological findings.

Response:

Regarding downstream analyses and motif discovery, we thank the reviewer for highlighting the need for additional clarification and reproducibility.

First, we clarify that all analyses presented in "GROTIA Reveals Gene-Specific Contributions and Key Biological Processes in the RNA Embedding", as well as Figures 4 and 5, are conducted on the PBMC-1 dataset.

In the revised manuscript, we add the following statement
"The PBMC-1 dataset is used to demonstrate the following experiments."
at the beginning of the three sections respectively titled
- "GROTIA Reveals Gene-Specific Contributions and Key Biological Processes in the RNA Embedding",
- "GROTIA Identifies High-Impact Peaks and Regulatory Mechanisms in the ATAC Embedding", and
- "GROTIA Enables Identification of Cellular Subpopulations in the Integrated Space":

Second, regarding motif discovery, we confirm that the use of GimmeMotifs is fully consistent with the workflow illustrated in Figure 5A. To improve transparency and alignment between the text and the figure, We have revised the Interpretable Embeddings of GROTIA section to explicitly detail each step of the motif discovery and annotation process, as follows:

"We then identified transcription factor binding sites (TFBS) by performing de novo motif discovery on the most influential ATAC-seq peaks associated with each latent dimension using GimmeMotifs \citep{bruse2018gimmemotifs}, applying a false discovery rate (FDR) threshold of < 0.001. This analysis yields enriched DNA sequence motifs representing putative TF binding sites in accessible chromatin and, through motif annotation, provides a set of candidate transcription factors (TFs) associated with each motif. Specifically, each de novo transcription factors binding site (TFBS) was annotated by matching its position weight matrix (PWM) to reference motif databases (including JASPAR, HOCOMOCO, CIS-BP, and ENCODE) using Pearson correlation–based similarity scores, thereby assigning candidate transcription factors (TFs) to each TFBS based on similarity to known binding preferences. We next linked individual TFBS-containing peaks to proximal genes by assigning each site to the nearest gene within a ±20 kb window \citep{kan2022multiomics, diwadkar2020facilitating}. Finally, these candidate target genes were filtered by

Powered by Editorial Manager® and ProduXion Manager® from Aries Systems Corporation

intersecting them with the top RNA-expressed genes associated with the same latent dimension, yielding dimension-specific TF–gene regulatory pairs.”

—---------------------------------------

Comments:

2. Usability

The code repository is easy to find on GitHub, available under the MIT license, following the link presented in the manuscript. However, the currently presented implementation is provided as a Jupyter Notebook that demonstrates the basic usage of the method, and technically allows users to replicate the process using their own data. Usability is currently limited by sparse documentation and could benefit from guidance on input requirements, parameter configuration, and expected output formats. To improve usability, the authors should supplement the notebook with detailed explanations, comments, and a README or user guide that explains how to prepare input data, adjust key parameters, interpret outputs, and run the method on other datasets. Wrapping core functionality into a small, importable Python module or script would further reduce friction for adoption and integration into pipelines.

Response:

We thank the reviewer for the constructive suggestions regarding code usability and documentation. Although the implementation is presented in Jupyter Notebook format, the workflow is designed to run on Google Colab, providing a stable and environment-independent execution setting that lowers the barrier for reproduction and modification.

In response to the reviewer’s comments, we have substantially improved the usability and documentation of the codebase. We have expanded the documentation to clearly describe input requirements, parameter configurations, and expected output formats, and each notebook now includes step-by-step guidance for running the method and interpreting the results. In addition, all core functionalities have been refactored into standalone, importable .py modules, with major functions consolidated into a dedicated GROTIA_utils.py file that is directly imported by the notebooks. This modular design enables flexible reuse of individual components and easier integration into custom analysis pipelines. All of the above changes are available at https://github.com/PennShenLab/GROTIA.

—---------------------------------------

Comments:

3. Attribution and Software Transparency

The GitHub repository includes an evals.py script originally authored by the creators of SCOT (Pinar Demetci, Rebecca Santorella, and Ritambhara Singh), with attribution preserved within the file. However, the manuscript itself does not mention that components of the evaluation pipeline were adapted from this prior work. Given that this script supports benchmarking comparisons central to the paper's conclusions, explicit acknowledgment in the text would improve transparency and ensure appropriate credit is given.

Response:

We thank the reviewer for pointing this out. The evals.py script in our repository is adapted from the evaluation code released with SCOT \citep{demetci2022scot}, with attribution preserved in the source file. We have now explicitly acknowledged this in the manuscript under the Evaluated Metrics section as followed:
“The second metric is Label transfer accuracy (LTA) and it evaluates how well cell-type (or other categorical) labels can be transferred from domain $X$ to domain $Y$ in the integrated space \citep{demetci2022scot}.”

—---------------------------------------

Powered by Editorial Manager® and ProduXion Manager® from Aries Systems Corporation

Comments:

4a. Support for Claims and Biological Interpretation

Several key claims would benefit from additional evidence or clarification. I divide this into subsections "4a. Methodological Claims," "4b. Biological Interpretation," and "4c. Clustering Evaluation" for extra clarity and readability.

4a. Methodological Claims
- The claim "we selected the latent dimension to be either 5 or 8 and observed that GROTIA remained robust to this choice" is not substantiated by any reported results or sensitivity analysis.

Response:

The choice of latent dimension was guided by prior kernel-based integration methods, particularly MMD-MA, which commonly uses a latent dimension of 5. Following this established practice, we selected latent dimensions of 5 or 8 in our main experiments.

To explicitly assess robustness, we additionally evaluated a broader range of latent dimensions (5, 8, 16, and 32). We now include a Hyperparameter Robustness Analysis section in the Appendix. As shown in Figure A.3, latent dimensions of 5 and 8 consistently achieve the best or near-best label transfer accuracy across all evaluated datasets (SNARE, scGEM, PBMC-1, and PBMC-2). While performance gradually degrades at higher dimensions (16 and 32), no abrupt instability is observed, indicating that GROTIA is robust to reasonable variations in latent dimension.

We have revised the Training Details section to explicitly reference this analysis, stating:
“A more detailed explanation is provided in the Appendix: Optimization Details.”

—---------------------------------------

Comments:

- The claim that GROTIA is computationally efficient would be more compelling if runtime comparisons included system specifications, analysis on larger (potentially synthetic) datasets, memory usage, and scalability assessments across CPU and GPU modes. Directly referencing Table A1 for the current runtime evaluation and adding the additional metrics mentioned above would provide a more comprehensive evaluation.

Response:

We thank the reviewer for this constructive suggestion. To address this point, we have added wall-clock runtime, peak GPU memory allocated, and peak CPU resident memory (RSS) for all benchmarked methods on the PBMC-1 dataset (9,378 cells) in the Appendix: Metric Performance section.

As shown in Table A1, while GROTIA does not achieve lower memory usage than batched methods such as UniPort and scConfluence, it remains substantially more efficient than other full-matrix approaches (e.g., SCOT, UnionCom, and MMD-MA) in both runtime and memory consumption. This highlights that, despite adopting a full-matrix formulation, GROTIA achieves a favorable efficiency–accuracy trade-off at practical single-cell scales. Providing a more detailed GPU scalability analysis is a promising direction for future work.

In addition, we revised the Discussion section as follows:

“Benchmarking against state-of-the-art methods in both unsupervised and semi-supervised settings, GROTIA delivers comparable or superior performance while offering a computationally efficient solution (see Appendix: Metric Performance).”
We have also added system configuration details to the Training Details section, as follows:

Powered by Editorial Manager® and ProduXion Manager® from Aries Systems Corporation

“All experiments were run on a single NVIDIA A100 GPU.”

—---------------------------------------

Comments:

- The manuscript asserts "Notably, unlike methods that require shared features across modalities, GROTIA only assumes that cells (rather than individual genes or peaks) follow a similar distribution if they belong to the same type or lineage—thus broadening its applicability to complex datasets." This claim would be more convincing if supported by analyses on more complex datasets, such as those with technical variability across origin sites, donors, or protocols; mosaic structures with missing observations; nested batch effects; or significant differences in data quality. Additionally, this statement may appear in tension with the claim that GROTIA depends on the presence of a shared underlying biology, which would not hold in many complex or heterogeneous settings. Clarifying how "complexity" is defined in the context of GROTIA's assumptions, and empirically substantiating the method's generalizability to such settings would improve both the precision and credibility of this claim.

Response:

We thank the reviewer for pointing out that our statement about “not requiring shared features” and “complex datasets” needed clearer justification. In GROTIA, we work with two modalities X and Y that can live in completely different feature spaces (for example, RNA expression vs chromatin accessibility). For each modality, we construct a cell–cell similarity matrix (a kernel matrix) from the high-dimensional feature profiles. What GROTIA does assume is shared cell-state structure, not shared features. Specifically, cells of the same type or lineage should form coherent neighborhoods in each modality, and the relative arrangement of these neighborhoods (which types are closer or more distinct) should be comparable across modalities. The kernels and graph regularization preserve this intra- and inter-type structure in both domains, and OT matches regions with similar internal geometry and relative position. This also explains how GROTIA behaves when one modality has more cell types than the other.

To further validate this behavior, we include an additional benchmark in which the RNA modality contains HeLa and HCT cancer cell lines, while the ATAC modality contains HeLa, HCT, and K562. This “2-versus-3 lineage” configuration allows us to assess whether GROTIA can correctly align the shared HeLa and HCT populations without spuriously matching the K562 population, which is present only in one modality.

We have described this setting in the Real World Datasets section as follows:

“In addition to the primary datasets described above, we evaluated GROTIA on an additional dataset with unbalanced cell populations to assess robustness, with full details and results provided in the Appendix: Additional Dataset.”

In the Appendix: Additional Dataset, we revised the description as follows:

“To evaluate robustness under unbalanced cell population settings, we considered a benchmark scenario in which one shared cell population is entirely missing from one modality. Specifically, we used a paired scRNA–scATAC dataset in which the scRNA-seq modality contains HeLa and HCT cell lines, while the scATAC-seq modality contains HeLa, HCT, and K562 cell lines. This setup creates an unbalanced integration scenario with mosaic structure and missing observations across modalities, mimicking realistic diagonal integration settings. Full details of the dataset could be found at \citet{samaran2024scconfluence}.

To evaluate robustness under unbalanced cell population settings, we considered a benchmark scenario in which one shared cell population is entirely missing from one modality. Specifically, we used a paired scRNA–scATAC dataset in which the scRNA-seq modality contains HeLa and HCT cell lines, while the scATAC-seq modality contains HeLa, HCT, and K562 cell lines. This setup creates an unbalanced integration scenario with mosaic structure and missing observations across modalities, mimicking realistic diagonal integration settings. Full details of the dataset could be found at

Powered by Editorial Manager® and ProduXion Manager® from Aries Systems Corporation

\citet{samaran2024scconfluence}.

Quantitative results on the cell-line dataset demonstrate that GROTIA is robust to variability in cell population proportions. In the unsupervised setting, GROTIA achieves the highest label-transfer accuracy (0.954), slightly outperforming the next best method, UnionCom (0.947). Its FOSCTTM score (0.318) is the second best and close to the best score achieved by UnionCom (0.287), indicating competitive alignment quality without supervision.

In the semi-supervised setting, GROTIA attains a label-transfer accuracy of 0.985, matching the best-performing methods (MMD-MA and scConfluence with prior, both 0.985). Its FOSCTTM score (0.323) is also comparable to other strong baselines (0.280 for UnionCom and 0.272 for scConfluence with prior). Overall, these results show that even when the two modalities differ in feature space and cell-type composition, GROTIA reliably recovers shared cell-state structure without requiring shared features, while appropriately handling cell populations present in only one modality."

—---------------------------------------

Comments:

- While the manuscript assesses alignment quality using Fraction of Samples Closer Than the True Match (FOSCTTM) and Label Transfer Accuracy (LTA), capturing local alignment and biological label concordance, these metrics do not directly evaluate preservation of global structure. Since GROTIA is designed to balance both global and local alignment, it would be helpful to include an explicit global alignment metric to confirm that this objective is being met. Some of the provided figures (e.g., Fig. 2c, right panel, and Fig. 3b after alignment) suggest global structure is preserved, but incorporating a dedicated metric or discussion would strengthen the evidence and provide a more complete evaluation of alignment quality.

Response:

We thank the reviewer for this comment. Both FOSCTTM and label transfer accuracy (LTA) are best viewed as local–global hybrid metrics, and they are widely used in the single-cell integration literature to evaluate complementary aspects of alignment quality.

FOSCTTM evaluates local neighborhood correctness by comparing, for each cell, the distance to its true cross-modal match against other samples, and reports the average score across all cells, providing a dataset-level summary of local alignment fidelity. LTA is computed via local kNN voting but assesses global biological consistency by measuring cross-modal agreement of cell-type identities across the entire dataset. Developing more explicit and comprehensive global geometric alignment metrics is an important direction for future work.

—---------------------------------------

Comments:

- Likewise, the manuscript states that GROTIA employs orthogonality constraints within the Reproducing Kernel Hilbert Space (RKHS) to enhance interpretability and stability. The use of these constraints for interpretability is illustrated through feature importance analyses; however, there is no direct comparison showing that this approach yields improved interpretability relative to unimodal analyses. Additionally, the effect of orthogonality constraints on embedding stability is not clearly assessed. Providing empirical evidence that these constraints improve the consistency of the embeddings or the quality of feature discovery, particularly in relation to single-modality methods, would help confirm the added value of this design choice and support several of the broader claims made regarding marker gene discovery and cell population characterization.

Response:

Powered by Editorial Manager® and ProduXion Manager® from Aries Systems Corporation

We appreciate the reviewer’s comment. In GROTIA, the orthogonality constraints imposed in the RKHS were previously described as improving interpretability; however, we agree that this wording may be misleading. We have therefore revised the manuscript to remove claims of improved interpretability and instead emphasize the role of these constraints in enhancing embedding stability.

Specifically, the RKHS orthogonality constraint helps prevent rank degeneracy, reduces redundancy between latent axes, and avoids collapsed embeddings arising from graph Laplacian regularization. The effects of this constraint are now explicitly analyzed in the Appendix: Effect of the RKHS Orthogonality Constraint. We have added the following context to the GROTIA Algorithm section:

“For details on the effect of this constraint, we refer the reader to the Appendix: Effect of the RKHS Orthogonality Constraint.”

—---------------------------------------

Comments:

- The decision to exclude scConfluence from the scGEM and SNARE evaluations due to prior dimensionality reduction could be better substantiated. Since raw data for both datasets are publicly available (e.g., SNARE-seq on GEO, scGEM on SRA), it would be helpful to explain why reprocessing the data was not feasible or appropriate.

Response:

We thank the reviewer for pointing this out and for noting that raw scGEM and SNARE-seq data are publicly available. We agree that, in principle, scConfluence can be run in a “prior-informed” mode when one can meaningfully define a shared gene space (as done in the original paper via gene activity matrices for ATAC). However, in the specific case of scGEM, SNARE-seq, and our simulated datasets, we were not able to construct a sensible shared feature set that would make this configuration well defined and comparable across methods.

In line with the scConfluence paper, we attempted to follow their strategy and derive gene-level activity scores for ATAC and then subset both modalities to a common set of genes. However, the original article uses a heterogeneous collection of dataset-specific pipelines and tools for this step (Maestro, Signac, Cicero, etc.), and does not provide an explicit, unified recipe or code for constructing gene activities and shared genes for scGEM, SNARE-seq, or for the kinds of simulated data we consider. When we tried to reproduce a similar procedure for these datasets, we did not obtain a non-trivial, biologically meaningful overlap of genes that we were confident using as a prior. At the same time, choosing one particular peak-to-gene mapping tool and parameterization only for scConfluence (and only for these datasets) would introduce an extra layer of method- and dataset-specific engineering that no other baseline benefits from, making the comparison less fair and less reproducible.

We therefore adopted the following consistent strategy in our benchmarks: (i) for datasets where a natural shared feature space exists and the necessary metadata for gene-level alignment are already available (e.g. the PBMC1 and PBMC2 datasets used in the paper), we run scConfluence in its prior-informed mode and also report its diagonal (no-prior) variant; (ii) for scGEM, SNARE-seq, and the remaining simulated datasets, where a robust, interpretable shared gene set cannot be defined in a principled way, we only include the diagonal configuration of scConfluence, which uses the same standardized inputs as the other methods and does not rely on an additional peak-to-gene prior.

Finally, we note that all other benchmarked methods in our study are used in a fully unsupervised, no-prior setting. Adding a feature-level prior is therefore not required for a fair comparison. We chose to report the prior-informed configuration of scConfluence only in those datasets where it is naturally and reproducibly defined, to illustrate that GROTIA remains competitive even when a competing method is allowed to leverage additional prior information, while keeping the main benchmark results strictly

Powered by Editorial Manager® and ProduXion Manager® from Aries Systems Corporation

comparable across methods in a prior-free, unsupervised regime.

We have added the following explanation to the Baseline Settings section:

“For the four simulated datasets as well as the scGEM and SNARE-seq datasets, the construction of a cross-modality distance matrix is non-trivial. Specifically, the simulated datasets lack meaningful cross-modal feature definitions, and computing such distances for scGEM and SNARE-seq is not feasible.”

—---------------------------------------

Comments:

4b. Biological Interpretation
- The reasoning in the statement "Notably, GROTIA requires no a priori matching of features across modalities, so these dimension-specific drivers offer an unbiased method to uncover potential marker genes" is somewhat unclear. While the method's ability to operate without explicit feature matching is a strength, it would be helpful to clarify how this property directly leads to unbiased marker discovery. In particular, elaborating on how the dimension-specific drivers compare to features identified through unimodal or matched-feature approaches, would strengthen the interpretation.

Response:

We thank the reviewer for this helpful comment and agree that our original wording was not sufficiently precise. In particular, the term “unbiased” was potentially misleading. In the revised manuscript, we have removed this shorthand and replaced it with “data-driven,” by which we mean agnostic to pre-defined cross-modal feature matching or marker selection. Concretely, GROTIA is trained without constructing peak-to-gene mappings, without restricting to a hand-picked shared gene set, and without supplying curated marker lists. Integration is performed purely at the cell level based on within-modality structure. Once the joint latent space is learned, we compute dimension-specific drivers by scoring all features in each modality with respect to each latent dimension. The resulting rankings are therefore determined by the learned joint geometry of the data, rather than by an externally engineered shared feature space.

To address the comparison requested by the reviewer, we provide the following explanation:
- Unimodal marker discovery (e.g. RNA-only workflows) identifies features using a single modality and does not perform multi-omic integration, so its markers cannot reflect relationships that become apparent only when multiple modalities are considered jointly.
- Matched-feature methods typically require both modalities to be represented in a common feature space (for example, by computing gene-activity matrices for ATAC and subsetting to “shared genes”). In these approaches, marker discovery is restricted to that engineered shared subset and can be influenced by the particular peak-to-gene mapping strategy, tool choice, and preprocessing parameters.
- GROTIA’s dimension-specific drivers are computed after multi-omic alignment, over the full original feature set of each modality, and without relying on any specific cross-modal feature matching pipeline or prior marker set. This avoids constraining marker discovery to a particular constructed shared gene space and provides a data-driven way to rank features along each latent dimension.

Because unimodal and matched-feature methods operate in fundamentally different feature spaces (single-modality genes versus engineered shared gene sets), their marker lists are not directly comparable to GROTIA’s dimension-specific drivers in a one-to-one fashion. Instead, we validate GROTIA’s feature rankings by examining internal multi-omic consistency. In particular, as shown in Fig. 5, for representative latent dimensions a large fraction of the top ATAC peaks lie in the vicinity of the top RNA genes for the same dimension, and these peak–gene pairs tend to be positively correlated across cells. This supports our interpretation that GROTIA provides data-driven, axis-wise feature importance derived from the learned joint geometry, without relying on pre-defined cross-modal feature matches or prior marker selection.

Powered by Editorial Manager® and ProduXion Manager® from Aries Systems Corporation

—---------------------------------------

Comments:

- Several statements related to cell-type-specific gene expression, such as "LYZ, ZEB2, PLXDC2 are highly expressed in monocytes…", would benefit from appropriate citations. This applies to other claims throughout the manuscript regarding gene specificity for particular lineages or subtypes.

Response:

We thank the reviewer for this helpful suggestion. In the revised manuscript, we have added citations to established marker-gene resources, primarily drawing from the Human Protein Atlas database.

—---------------------------------------

Comments:

4c. Clustering Evaluation

- The claim that GROTIA achieves "comparable or better performance" than Louvain clustering is not fully supported. While ARI/NMI scores of 0.75-0.8 indicate reasonable alignment with reference annotations, clarity on how ground truth (reference) labels were defined, whether Louvain resolution parameters were tuned, and which dataset(s) were used would strengthen this comparison. Additionally, specifying which co-clustering algorithm was used from the cited Python package, along with its parameter settings, would improve reproducibility and interpretability.

Response:

We thank the reviewer for these detailed comments and agree that our original description of the Louvain and co-clustering baselines was not sufficiently specific. We have clarified the following points in the revised manuscript.
First, the “reference” or “ground truth” labels used to compute ARI, NMI, and Purity correspond to the cell-type annotations provided by the original studies for each dataset, as described in the Data section. The PBMC-1 dataset is used as a representative example to illustrate this evaluation procedure.

Second, for the Louvain baseline, we used the standard Louvain implementation from the Python ecosystem applied to the same cell–cell graph used in our other analyses. The resolution parameter was tuned over the grid {0.1, 0.3, 0.4, 0.5, 0.6, 1.0, 1.5}, and the value that maximized the sum of ARI, NMI, and Purity with respect to the ground truth cell-type labels was selected for each dataset. In the revised manuscript, we explicitly specify which datasets the reported ARI/NMI values (0.75 - 0.8) correspond to and list the associated resolution choices. This clarification ensures that the comparison between GROTIA and the Louvain baseline reflects a clearly defined tuning protocol rather than a single untuned run.

We have added the following clarification to the section GROTIA enables identification of cellular subpopulations in the integrated space:

“We tuned the resolution parameter over the grid resolution $\in$ {0.1, 0.3, 0.4, 0.5, 0.6, 1.0, 1.5} and selected the value that maximized the sum of ARI, NMI, and Purity with respect to the ground truth cell type labels on that dataset.”

Third, for the co-clustering baseline, we used the algorithm “co-clustering by alternated maximization of bipartite graph modularity” from the cited Python package. In this method, the only nontrivial hyperparameter is the number of clusters. As described in the manuscript, we determine the number of clusters for each dataset using an elbow criterion on the modularity curve, and then prune any resulting clusters with fewer than 20 cells as noise.

We have revised the section “Co-Clustering Using the Optimal Transport Plan” as

Powered by Editorial Manager® and ProduXion Manager® from Aries Systems Corporation

follows:

“GROTIA also enables post-integration analysis to identify data-driven clusters. Specifically, an optimal transport (OT) plan is first computed to quantify the flow between two distinct sets of entities (e.g., cells in the RNA and ATAC spaces). We then apply a co-clustering algorithm \citep{role2019coclust} based on alternating maximization of bipartite graph modularity directly to the resulting transport matrix, thereby simultaneously grouping row and column entities. By treating the OT plan as a bipartite graph, this co-clustering approach identifies latent structural patterns that minimize within-cluster transport costs while maximizing separation between clusters. Following co-clustering, clusters containing fewer than 20 cells are pruned as noise.”

—---------------------------------------

Comments:

- The claims that GROTIA can uncover finer structures and novel cellular states, as well as identify refined subpopulations aligned with major cell types, are intriguing but would benefit from additional support. As currently presented, the results do not highlight specific novel cell populations or provide examples of newly discovered subclusters.

Response:

We thank the reviewer for this comment and agree that, as originally written, the phrases “uncover finer structures and novel cellular states” and “identify refined subpopulations aligned with major cell types” could be interpreted as stronger biological claims than what is directly supported by our current analyses.

Our intent was not to claim that GROTIA has already identified entirely new or previously unreported cell states in these datasets. Rather, we aimed to emphasize two more specific and empirically supported properties of the method. First, the integrated latent space produced by GROTIA enables finer, unsupervised refinement of known cell populations, as evidenced by improved NMI, ARI, and Purity scores relative to existing methods, indicating closer agreement between unsupervised clustering results and expert cell-type annotations. Second, GROTIA supports practical analysis settings in which cell-type annotations are available in only one modality. This is facilitated by the co-clustering procedure applied to the optimal transport plan, which jointly groups cells across modalities and allows cell population structure to be inferred in the unannotated modality through its alignment with the annotated one.

We have therefore removed the statement “By clustering cells within GROTIA’s integrated representation, we can uncover finer structures and novel cellular states that may otherwise remain masked by legacy annotations.”

In its place, we have added the following sentence to the section “GROTIA enables identification of cellular subpopulations in the integrated space”:

“By clustering cells in GROTIA’s integrated latent space, we recover cell population structure that closely matches expert annotations and enable inference of corresponding cell labels in a second modality when annotations are available in only one modality.”

—---------------------------------------

Comments:

5. Writing, organization, tables, and figures, and minor notes

- There is a typo in the heading "GROTIA integrated simulated datasets in both semi and unsuperviseed setting" where unsuperviseed should be unsupervised.

Response:

We thank the reviewer for pointing out this typo. We have corrected “unsuperviseed” to “unsupervised” in the revised manuscript.

—--------------------------------------

Comments:

-- Under this heading, the section describing Figure 2a in paragraph two and paragraph three largely overlap.

Response:

We thank the reviewer for pointing this out. We have revised the manuscript to remove the redundancy between the second and third paragraphs under this heading and clarified the description of Fig. 2a to avoid overlapping content.

—--------------------------------------

Comments:

- The results and interpretation of Figure 2 panel b and c are not described to the reader. The same is true for Figure 3 panels b and c.

Response:

We thank the reviewer for this comment. We have added detailed descriptions and interpretations of Fig. 2 panels b and c, as well as Fig. 3 panels b and c, in the revised manuscript to clarify the results shown in these figures.

—--------------------------------------

Comments:

- In Figure 3, the method is still labeled as GROT instead of GROTIA; this should be updated for consistency.

Response:

We thank the reviewer for pointing this out. We have corrected the label in Figure 3 from GROT to GROTIA to ensure consistency throughout the manuscript.

—--------------------------------------

Comments:

- In Figure 3, the abbreviations Semi Acc and Un Acc are not defined in the legend and should be clearly explained.

Response:

Label Transfer Accuracy is shown with semi-supervised results on the x-axis (Semi Acc) and unsupervised results on the y-axis (Un Acc)

—--------------------------------------

Comments:

- In Figure 3, the visual layout in panel b differs between datasets and may be confusing for scGEM and SNARE-seq, the left and right columns represent cell types from each modality, whereas for PBMC, they reflect cell type and modality origin from a single, combined dataset. The PBMC-style presentation is more effective for visually assessing global alignment and should either be used consistently or more clearly explained.

Response:

We thank the reviewer for this helpful observation. We agree that the visual layouts in Fig. 3b differs across datasets. For the scGEM and SNARE-seq datasets, we intentionally retained the original visualization style used in the corresponding benchmark methods in order to maintain consistency with prior work and to facilitate direct comparison when readers refer to the original references.

—---------------------------------------

Comments:

- In Figure 3, legends are also missing descriptions of the color schemes used to denote modality.

Response:

We thank the reviewer for pointing this out. We have updated the legend of Figure 3 to explicitly describe the color scheme used to denote different modalities, ensuring that the figure is self-contained and easier to interpret.

—---------------------------------------

Comments:

- In panel c of both Figures 2 and 3, it should be specified whether the results correspond to semi-supervised or unsupervised alignment.

Response:

We thank the reviewer for this comment. We have clarified in the figure legends that the results shown in panel c of both Figures 2 and 3 correspond to the unsupervised alignment setting.

—---------------------------------------

Comments:

- In statements such as "Figure 4b presents UMAP visualizations of the top gene expression patterns for Dimensions 1 and 3", the wording could be clarified to avoid confusion. Specifically, it would help to state that gene expression patterns are overlaid on a UMAP projection of the scRNA-seq data, and that the genes visualized were selected based on their importance in Dimensions 1 and 3 of the RBF kernel embeddings (not UMAP axes).

Response:

We thank the reviewer for this helpful suggestion. We have revised the wording to clarify that gene expression patterns are overlaid on a UMAP projection of the scRNA-seq data, and that the genes shown were selected based on their importance in Dimensions 1 and 3 of the RBF kernel embeddings rather than the UMAP axes.

—---------------------------------------

Comments:

- In Figure 4 panel a, it appears that several genes from D1-4 have higher importance in D5. Is this due to scaling, or does it have some biological interpretation?

Response:

We thank the reviewer for this observation. This effect arises from the way Fig. 4a is constructed to provide a diagonal-style comparison across latent dimensions. Some genes therefore appear more than once across different dimensions, and higher

Powered by Editorial Manager® and ProduXion Manager® from Aries Systems Corporation

| | |
|---|---|
| | importance values outside the highlighted (red-boxed) dimension correspond to the same genes selected within the box. This design choice was made to facilitate visual comparison across dimensions<br><br>—---------------------------------------<br><br>Comments:<br><br>- In Figure 4, panel c, the colorbar should be labeled.<br><br>Response:<br><br>We thank the reviewer for pointing this out. We have added an explicit label to the colorbar in Figure 4c in the revised manuscript.<br><br>—---------------------------------------<br><br>Comments:<br><br>- In Figure 5, panel a, only chromosome identifiers are shown, making the peak information incomplete and difficult to interpret. Including specific peak coordinates would improve clarity.<br><br>Response:<br><br>We thank the reviewer for this comment. Figure 5a is intended as an illustrative overview rather than a detailed genomic annotation, and therefore displays only chromosome-level identifiers to maintain visual clarity.<br><br>—---------------------------------------<br><br>Comments:<br><br>- In ... |
| **Additional Information:** | |
| **Question** | **Response** |
| Are you submitting this manuscript to a special series or article collection? | No |
| **Experimental design and statistics**<br><br>Full details of the experimental design and statistical methods used should be given in the Methods section, as detailed in our Minimum Standards Reporting Checklist. Information essential to interpreting the data presented should be made available in the figure legends.<br><br>Have you included all the information requested in your manuscript? | Yes |
| **Resources** | Yes |

Powered by Editorial Manager® and ProduXion Manager® from Aries Systems Corporation

| | |
|---|---|
| A description of all resources used, including antibodies, cell lines, animals and software tools, with enough information to allow them to be uniquely identified, should be included in the Methods section. Authors are strongly encouraged to cite Research Resource Identifiers (RRIDs) for antibodies, model organisms and tools, where possible.<br><br>Have you included the information requested as detailed in our Minimum Standards Reporting Checklist? | |
| **Availability of data and materials**<br><br>All datasets and code on which the conclusions of the paper rely must be either included in your submission or deposited in publicly available repositories (where available and ethically appropriate), referencing such data using a unique identifier in the references and in the "Availability of Data and Materials" section of your manuscript.<br><br>Have you have met the above requirement as detailed in our Minimum Standards Reporting Checklist? | Yes |
| GigaScience has policies and guidelines in place for the use of generative AI-writing tools such as ChatGPT. If you have used such writing tools to assist with writing the manuscript this must be declared and cited in the text. Authors should not list AI-writing tools and other AI-assisted technologies as an author or co-author and should acknowledge that they are fully responsible for text generated or refined by AI-writing tools.<p><br><br>A summary of use (particularly in the introduction or among methods) needs to be included at the end of the paper, and the outputs should also be included as a supplementary file hosted in GigaDB or other open repositories. Please <a href=https://academic.oup.com/gigascience/pages/editorial_policies_and_reporting_standards target="_new" > read our guidelines for more information. </a> <p> | Yes |

| | |
|---|---|
| By submitting to GigaScience, you are aware of the journal's AI-writing tools policy, and if you have declared use of such tools below, you have acknowledged this where appropriate in your manuscript and have made a summary of use and outputs available. </b><p> <b>AI-assisted writing tools have been used in the preparation of this manuscript? | |

Powered by Editorial Manager® and ProduXion Manager® from Aries Systems Corporation

Manuscript

Click here to access/download;Manuscript;GigaScience_GROTIA_Final_Ver

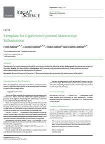

*GigaScience*, 2023, 1–22

doi: xx.xxxx/xxxx
**Manuscript in Preparation**
**Paper**

PAPER

# An Interpretable Graph-Regularized Optimal Transport Framework for Diagonal Single-Cell Integrative Analysis

Zexuan Wang[1,†], Qipeng Zhan[1†], Shu Yang[2], Zhuoping Zhou[1], Mengyuan Kan[2], Tianhuan Zhai[2] and Li Shen[2,*]

[1]Graduate Group in Applied Mathematics and Computational Science, University of Pennsylvania, Philadelphia, PA, USA and [2]Department of Biostatistics, Epidemiology and Informatics, Perelman School of Medicine, University of Pennsylvania, Philadelphia, PA, USA

[*]Correspondence: li.shen@pennmedicine.upenn.edu
[†]These authors contributed equally to this work.

## Abstract

**Background**: Recent advancements in single-cell omics technologies have enabled detailed characterization of cellular processes. However, coassay sequencing technologies remain limited, resulting in un-paired single-cell omics datasets with differing feature dimensions; **Finding** we present GROTIA (Graph-Regularized Optimal Transport Framework for Diagonal Single-Cell Integrative Analysis), a computational method to align multi-omics datasets without requiring any prior correspondence information. GROTIA achieves global alignment through optimal transport while preserving local relationships via graph regularization. Additionally, our approach provides interpretability by deriving domain-specific feature importance from partial derivatives, highlighting key biological markers. Moreover, the transport plan between modalities can be leveraged for post-integration clustering, enabling a data-driven approach to discover novel cell subpopulations; **Conclusions**: We demonstrate GROTIA's superior performance on four simulated and four real-world datasets, surpassing state-of-the-art unsupervised alignment methods and confirming the biological significance of the top features identified in each domain.

**Key words**: Optimal Transport; Graph Laplacian; Single Cell; Multi Omics; Data Integration; Interpretable.

## Introduction

The advancement of single-cell technology offers a comprehensive understanding of cellular heterogeneity and the dynamic evolution of cell states. Various single-cell measurements reveal different aspects: scRNA-seq [1, 2] provides detailed gene expression profiles, while scATAC-seq [3] sheds light on chromatin accessibility in individual cells. Integrating these datasets is crucial as it allows for a more holistic view of cellular mechanisms, enabling the correlation of transcriptional activity with chromatin states to better understand gene regulation and cellular function.

Lots of computational methods have recently been developed to integrate data across multiple modalities [4, 5, 6, 7, 8, 9]. A critical challenge for these algorithms is their reliance on correspondence information to identify alignments between paired cells. In practice, such information is often only partially available, hindering the effectiveness of existing strategies [10, 11, 8]. This limitation has led researchers to focus on diagonal integration under semi-supervised settings, where alignment is achieved without direct cell-to-cell correspondences, but cell type labels are still used for hyperparameter tuning. The Generalized Unsupervised Manifold Alignment (GUMA) method [12] aligns datasets by optimizing local geometric structures to establish a one-to-one correspondence. Building upon this, the Unsupervised topological alignment for single-cell multi-omics integration (UnionCom) algorithm by Cao et al. [7] enables semi-supervised topological alignment, relaxing

**Compiled on:** January 28, 2026.
Draft manuscript prepared by the author.

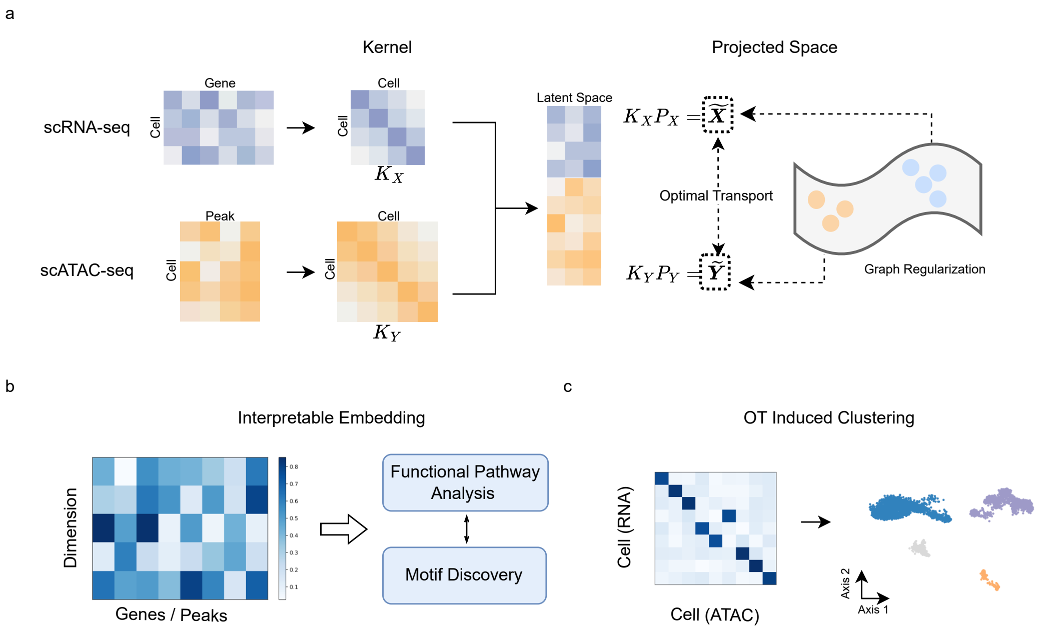


**Figure 1.** Overview of the GROTIA Framework for Multi-Omics Single-Cell Integration. (a) Schematic Design: For each single-cell modality (e.g., scRNA-seq, scATAC-seq), GROTIA constructs a kernel matrix capturing pairwise cell similarities (e.g., $K_X$ and $K_Y$ ). It then learns mapping matrices $P_X$ and $P_Y$ to project cells from each modality's RKHS into a shared latent space, where distributions are aligned via optimal transport. A graph regularization term preserves local neighborhood structure, ensuring that cells close in the original domain remain similarly positioned after integration. (b) Interpretable Embedding: Once the shared embedding is obtained, GROTIA provides dimensionwise importance scores for genes or peaks. These scores can be used for downstream analyses such as Gene Ontology (GO) or motif discovery, provide biological interpretation of each latent dimension. (c) OT-Induced Co-Clustering: GROTIA leverages the cross-modality transport plan, which quantifies how strongly each scRNA cell aligns with each scATAC cell. By simultaneously grouping cells from both modalities according to these alignment strengths, GROTIA identifies co-clusters of subpopulations with closely matched regulatory states in the latent space.

GUMA's strict one-to-one mapping requirement. Liu et al. [13] proposed an alternative manifold alignment strategy called MMD-MA, which employs the Maximum Mean Discrepancy (MMD) metric for alignment. Additionally, the Single-Cell Multi-Omics Alignment with Optimal Transport (SCOT) method [6] utilizes Gromov-Wasserstein distances to align multi-omic single-cell data. Autoencoder based method have also been proposed to align data across modality to use per ae per modality and align them in shared latent space [14, 9]. However, even when integration is performed without correspondences, hyperparameters are often tuned using cell label validation, rendering the process semi-supervised. Demetci et al. [6] demonstrated that most methods fail to adapt to fully unsupervised settings when no orthogonal alignment information is available.

Diagonal single-cell multi-omics integration thus faces several key obstacles. First, in the absence of paired samples, one must work under an unpaired assumption, which is common given the practical and financial difficulties of obtaining perfectly matched datasets. Second, integration often relies on shared features (e.g., overlapping genes) that may be missing or poorly represented across modalities. Third, most computational pipelines rely on label-based metrics for hyperparameter tuning, which is problematic in truly unsupervised settings where no external annotations exist. Finally, many existing methods lack an interpretable framework to explain the learned shared embeddings.

Here, we propose GROTIA, a fully unsupervised diagonal integration method that uses optimal transport and graph regularization to establish alignment without relying on one-to-one correspondences or labeled data. We embed each dataset in a high-dimensional kernel space to capture cell–cell similarities, then learn mappings that transform each dataset into a shared lower-dimensional space for direct comparison. Our framework preserves local geometry via graph Laplacian regularization while performing global alignment through optimal transport, thereby avoiding the need for label-based hyperparameter tuning. In addition, we provide gradient-based sensitivity analyses to highlight key biological genes and peaks that drive the alignment, clear interpretability of the learned latent representation.

We extensively evaluate our model against SCOT, MMD-MA, UnionCom, Uniport, Scconfluence across four simulated and four real-world datasets in unsupervised and semi-supervised settings. Our Graph-Regularized Optimal Transport (GROTIA) algorithm matches the performance of state-of-the-art methods. The schematic design of our approach is illustrated in **Figure 1**.

## Methods

### Simulated Datasets

We evaluated the GROTIA algorithm using four simulated datasets: three from Liu et al. [13], specifically designed to test alignment methods with different geometric structures, and one additional dataset from Demetci et al. [6], simulating single-cell RNA sequencing count data via Splatter [15]. Specifically, the first dataset presents a branch structure in two-dimensional space, the second a Swiss roll in three-dimensional space, and the third a circular frustum also in three-dimensional space. Although these datasets originally feature complex topological and geometric structures in low-dimensional spaces, they have been nonlinearly projected into high-dimensional feature spaces of 1000 and 2000 dimensions for evaluating alignment methods. The fourth is a synthetic RNA-seq dataset from Demetci et al. [6], consisting of 5,000 cells with either 50 or 500 features. Following the approaches in the original publications, we applied Z-score normalization to all features before

running alignment algorithms.

## Real World Datasets

We then evaluated the GROTIA algorithm on four real-world datasets, widely recognized as gold-standard benchmarks in multi-omics integration and commonly used for assessing state-of-the-art methods. These include: (1) scGEM, which simultaneously profiles gene expression and DNA methylation [16]. ; (2) a dataset generated by the SNARE-seq assay, linking chromatin accessibility with gene expression [17].; (3) a human PBMC dataset (PBMC 10X) consisting of 9,378 cells per modality [9].; and (4) an additional PBMC 10X dataset containing 11,259 cells per modality [14]. We chose these paired multi-omics datasets specifically to enable diagonal integration with known ground-truth cell correspondences. Importantly, during benchmarking, all methods are provided with unpaired data, and the known cell-pairing information was used only for evaluating alignment accuracy.

The first real-world dataset, named "scGEM," measures gene expression and DNA methylation in the same cells and was generated using the scGEM assay. It contains human somatic cells reprogrammed to a pluripotent state, forming a continuous developmental trajectory. Cao et al. [7] and Demetci et al. [6] previously employed this dataset to evaluate integration methods. Specifically, it has 177 cells with 34 gene-expression features and 177 cells with 27 DNA methylation features. We used the preprocessed version from Demetci et al. [6].

The second real-world dataset, SNARE-seq, jointly profiles chromatin accessibility and gene expression. The dataset was preprocessed using cisTopic [18], resulting in an ATAC-seq matrix of 1,047 cells by 19 features and an RNA-seq matrix of 1,047 cells by 10 features. Following standard practice, unit normalization was then applied to these matrices. We used this preprocessed SNARE-seq dataset from Demetci et al. [6].

Additionally, we analyzed two multi-omics peripheral blood mononuclear cell (PBMC) datasets from publicly available 10x Genomics sources. The first, preprocessed by UniPort [9], contains 11,259 cells with 28,307 scATAC-seq features and 11,942 scRNA-seq genes. The second, preprocessed by scConfluence [14], includes 9,378 cells with 130,417 scATAC-seq features and 15,417 scRNA-seq genes. We used both PBMC datasets as provided for our integrative analyses.

In addition to the primary datasets described above, we evaluated GROTIA on an additional dataset with unbalanced cell populations to assess robustness, with full details and results provided in the *Appendix: Additional Dataset*.

## Problem Formulation

We introduce a method to integrate single-cell datasets across different conditions or modalities. Let us consider two datasets, $X$ and $Y$, with respective representations $X = \{x_1, \ldots, x_{n_X}\} \subset \mathcal{X}$ and $Y = \left\{y_1, \ldots, y_{n_Y}\right\} \subset \mathcal{Y}$, where $n_X$ and $n_Y$ denote the number cells in $X$ and $Y$, respectively. We aim to uncover a shared manifold structure between $X$ and $Y$ without a priori correspondence between the datasets.

To achieve this, we first compute the intra-dataset kernels $K_X$ and $K_Y$, which capture the internal structure of $X$ and $Y$, respectively. As long as it is positive definite, each kernel corresponds to an implicit feature mapping $\phi_X : \mathcal{X} \to \mathcal{H}_X$ and $\phi_Y : \mathcal{Y} \to \mathcal{H}_Y$, where $\mathcal{H}_X$ and $\mathcal{K}_Y$ are the Reproducing Kernel Hilbert Spaces (RKHS) associated with $K_X$ and $K_Y$. , we seek mapping functions $f_X : \mathcal{X} \to R^k$ and $f_Y : \mathcal{Y} \to R^k$, where $k$ is the dimensionality of the shared space. These functions are optimized so that the mapped representations $f_X(X)$ and $f_Y(Y)$ are well-aligned, thereby discovering the shared manifold structure.

## Kernel Representation

To capture the intrinsic geometry of the datasets, we define the intra-dataset kernels $K_X$ and $K_Y$ using Gaussian kernel functions:

$$K_X(x_i, x_j) = \exp\left(-\frac{\|x_i - x_j\|^2}{2\sigma_X^2}\right), \quad K_Y(y_i, y_j) = \exp\left(-\frac{\|y_i - y_j\|^2}{2\sigma_Y^2}\right). \tag{1}$$

where $\sigma_X$ and $\sigma_Y$ are bandwidth parameters specific to $X$ and $Y$, respectively. These kernels define the feature maps $\phi_X : \mathcal{X} \to \mathcal{H}_X$ and $\phi_Y : \mathcal{Y} \to \mathcal{H}_Y$ into their respective Reproducing Kernel Hilbert Spaces (RKHS).

We adopt a data-driven approach to determine $\sigma_X$ and $\sigma_Y$ by setting each parameter to the mean of the pairwise Euclidean distances within the corresponding dataset. This heuristic adjusts the bandwidths to reflect the average spatial dispersion of the data points, thereby tuning the kernels to the specific scale of variability in each dataset.

## Optimal Transport

For simplicity, we will use the notation: $\widetilde{X} = f_X(X) \in R^{n_X \times k}$, $\widetilde{Y} = f_Y(Y) \in R^{n_Y \times k}$ to represent the mapped datasets. The Sinkhorn divergence between the projected representations $\widetilde{X}$ and $\widetilde{Y}$ is defined as:

$$\mathcal{L}_{OT}(\widetilde{X}, \widetilde{Y}) = \mathrm{OT}_\varepsilon(\widetilde{X}, \widetilde{Y}) - \frac{1}{2}\left(\mathrm{OT}_\varepsilon(\widetilde{X}, \widetilde{X}) + \mathrm{OT}_\varepsilon(\widetilde{Y}, \widetilde{Y})\right) \tag{2}$$

where $\mathrm{OT}_\varepsilon(\cdot, \cdot)$ denotes the entropically regularized optimal transport cost between two distributions. Next, we define the entropic optimal transport cost between $\widetilde{X}$ and $\widetilde{Y}$. The cost is computed as:

$$\mathrm{OT}_\varepsilon(\widetilde{X}, \widetilde{Y}) = \min_{T \in \Pi(\mathbf{a}, \mathbf{b})} \langle C, T \rangle + \varepsilon H(T) \tag{3}$$

where $\Pi(\mathbf{a}, \mathbf{b}) = \left\{T \in R_+^{n_X \times n_Y} \mid T\mathbf{1}_{n_Y} = \mathbf{a}, T^\top \mathbf{1}_{n_X} = \mathbf{b}\right\}$. The matrix $T \in R_+^{n_X \times n_Y}$ is the transport plan matrix, representing the amount of mass transported from $\widetilde{x}_i$ to $\widetilde{y}_j$. The cost matrix $C \in R^{n_X \times n_Y}$ quantifies the pairwise distances between the projected samples. Each element is defined as: $C_{ij} = \left\|\widetilde{x}_i - \widetilde{y}_j\right\|_2^2$. The parameter $\varepsilon > 0$ is the entropic regularization parameter that smooths the optimization problem and $H(T) = -\sum_{i=1}^{n_X} \sum_{j=1}^{n_Y} T_{ij}\left(\log T_{ij} - 1\right)$ is the entropy of the transport plan $T$. The marginal distributions $\mathbf{a} \in R^{n_X}$ and $\mathbf{b} \in R^{n_Y}$ are typically uniform distributions over the samples: $\mathbf{a} = \frac{1}{n_X}\mathbf{1}_{n_X}$, $\mathbf{b} = \frac{1}{n_Y}\mathbf{1}_{n_Y}$, where $\mathbf{1}_{n_X}$ and $\mathbf{1}_{n_Y}$ are vectors of ones with lengths $n_X$ and $n_Y$, respectively. We utilize optimal transport over maximum mean discrepancy due to its benefits, such as non-vanishing gradients and other theoretical advantages [19].

## Graph Laplacian Regularization

To capture the local geometric structures of the datasets $X$ and $Y$, we construct graph Laplacians based on the $k$-nearest neighbor relationships defined through the Gaussian kernels. These Laplacians serve as regularizers for the mapping functions, enforcing smoothness by ensuring that nearby data points in the RKHS spaces $\mathcal{H}_X$ and $\mathcal{H}_Y$ remain close in the shared latent space.

For each dataset, we begin by constructing a $k$-nearest neighbor graph in the RKHS. Specifically, for dataset $X$, we identify the set of $k$-nearest neighbors for each feature-mapped data point $\phi_X(x_i)$, denoted as $\mathcal{N}_k(\phi_X(x_i))$, based on the distance metric in $\mathcal{H}_X$. The adjacency matrix $W_X \in R^{n_X \times n_X}$ is then defined with entries:

$$[W_X]_{ij} = \begin{cases} 1, & \text{if } \phi_X(x_j) \in \mathcal{N}_k(\phi_X(x_i)) \\ & \text{or } \phi_X(x_i) \in \mathcal{N}_k(\phi_X(x_j)) \\ 0, & \text{otherwise.} \end{cases} \tag{4}$$

Similarly, for dataset $Y$, we construct the adjacency matrix $W_Y \in R^{n_y \times n_y}$. Next, we compute the degree matrices $D_X$ and $D_Y$, which are diagonal matrices where each diagonal entry represents the sum of the edge weights connected to a node: $[D_X]_{ii} = \sum_{j=1}^{n_x} [W_X]_{ij}$, $[D_Y]_{ii} = \sum_{j=1}^{n_y} [W_Y]_{ij}$. The graph Laplacians are then defined as the difference between the degree and adjacency matrices:

$$L_X = D_X - W_X, \quad L_Y = D_Y - W_Y \tag{5}$$

To regularize the projected representations $\widetilde{X}$ and $\widetilde{Y}$, we introduce smoothness terms based on the Laplacian quadratic form. Specifically, the smoothness term for $\widetilde{X}$ is given by:

$$\frac{1}{2}\sum_{i=1}^{n_x}\sum_{j=1}^{n_x} [W_X]_{ij} \left\|\widetilde{x}_i - \widetilde{x}_j\right\|^2 = \mathrm{Tr}\left(\widetilde{X}^\top L_X \widetilde{X}\right) \tag{6}$$

where $\widetilde{x}_i$ denotes the $i$-th row of $\widetilde{X}$. This expression encourages neighboring points in the original data space to have similar representations in the latent space, promoting smoothness in the mappings.

## GROTIA Algorithm

To integrate the datasets $X$ and $Y$ into a shared latent space, we propose the Graph-Regularized Optimal Transport (GROTIA) algorithm. Our objective is to find the mapping $f_X : \mathcal{X} \to R^k, f_Y : \mathcal{Y} \to R^k$ that maps the data into a common $k$-dimensional space, effectively aligning their underlying manifold structures. The existence of such mapping is guaranteed by the representer theorem, which states that the optimal mappings can be expressed as finite linear combinations of the kernel functions:

$$[f_X]_j(x) = \sum_{i=1}^{n_x} \alpha_X^{ij} K_X(x_i, x) \tag{7}$$

where $\alpha_X^{ij}$ are the learned coefficients, $K_X$ is the kernel function, and $x_i$ are the samples from $X$. The coefficients $\alpha_X^{ij}$ are then organized into the matrix $P_X \in R^{n_x \times k}$, and similarity for Y we have $P_Y \in R^{n_y \times k}$. Thus, the final mapped representations are given by:

$$\widetilde{X} = K_X P_X, \quad \widetilde{Y} = K_Y P_Y \tag{8}$$

The optimization problem for the GROTIA algorithm is formulated as:

$$\begin{aligned} &\min_{P_X, P_Y} \mathcal{L}_{OT}(\widetilde{X}, \widetilde{Y}) \\ &+ \lambda_{topo} \left[\mathrm{Tr}(\widetilde{X}^\top L_X \widetilde{X}) + \mathrm{Tr}(\widetilde{Y}^\top L_Y \widetilde{Y})\right] \\ &+ \lambda_{ortho} \left[\|P_X^\top K_X P_X - I_k\|_F^2 + \|P_Y^\top K_Y P_Y - I_k\|_F^2\right]. \end{aligned} \tag{9}$$

The first term $\mathcal{L}_{OT}(\widetilde{X}, \widetilde{Y})$ aligns the global distributions of the datasets in the latent space using sinkhorn divergence. Minimizing the Sinkhorn divergence between the latent space representations ensures that the overall structures of $X$ and $Y$ are closely matched after projection. This captures global structural similarities and facilitates the discovery of shared manifold features between the datasets.

The second term $\mathrm{Tr}\left(\widetilde{X}^\top L_X \widetilde{X}\right)$ and $\mathrm{Tr}\left(\widetilde{Y}^\top L_Y \widetilde{Y}\right)$ is used preserve the local geometric structures inherent in each dataset. These terms penalize the weighted differences between neighboring points in the latent space. Doing so encourages neighboring points in the $\mathcal{H}_X, \mathcal{H}_Y$ to remain close in the latent space.

To prevent degenerate solutions and ensure that the mappings retain meaningful structure, we impose orthogonality constraints on the mapping matrices through the terms $\left(\|P_X^\top K_X P_X - I_k\|_F^2\right)$ and $\left(\|P_Y^\top K_Y P_Y - I_k\|_F^2\right)$. For details on the effect of this constraint, we refer the reader to the *Appendix: Effect of the RKHS Orthogonality Constraint*.

By jointly optimizing this objective function, the GROTIA algorithm effectively balances global alignment and local structure preservation while ensuring that the projections are meaningful and well-behaved.

## Interpretable Embeddings of GROTIA

In our approach, each domain (scRNA or scATAC) is mapped into a shared, low-dimensional space using kernel-based transformations. Take the scRNA space for example, let $X \in R^{n_x \times d_x}$ denote the data matrix for one domain, where $n_x$ is the number of cells and $d_x$ is the number of features. We construct a radial basis function (RBF) kernel $K \in R^{n_x \times n_x}$, with elements $K_{i,j} = \exp\left(-\gamma_X \left\|X_{i,\cdot} - X_{j,\cdot}\right\|^2\right)$ where $\gamma_X$ is bandwidth parameter. Through our optimization procedure, we learn a coefficients matrix $\alpha_X^{ij}$ such that the $k$-dimensional embedding for the $j$-th cell is given by $[f_X]_j(x) = \sum_{i=1}^{n_x} \alpha_X^{ij} K_X(x_i, x)$. Here, $[f_X]_j(x)$ represents the coordinate of cell $j$ in the $k$ dimensional learned embedding. An analogous formulation with $\beta$ is employed for the scATAC domain (using its own kernel matrix).

To identify which original features (e.g., genes in scRNA, peaks in scATAC) have the greatest influence on each embedding dimension, we compute partial derivatives of $f_d\left(x_j\right)$ with respect to each feature. Concretely, let $x_{j,g}$ denote the value of feature $g$ in cell $j$. Then, for an RBF kernel

$$\frac{\partial}{\partial x_{j,g}} K_{i,j} = -2\gamma\left(x_{j,g} - x_{i,g}\right) \exp\left(-\gamma \left\|x_j - x_i\right\|^2\right). \tag{10}$$

Using the chain rule, the partial derivative of the $d$-th embedding coordinate with respect to $x_{j,g}$ becomes

$$\begin{aligned} \frac{\partial f_d}{\partial x_{j,g}}(x_j) &= \sum_{i=1}^{N} \alpha_{i,d} \frac{\partial K_{i,j}}{\partial x_{j,g}} \\ &= -2\gamma \sum_{i=1}^{N} \alpha_{i,d}(x_{j,g} - x_{i,g}) K_{i,j}. \end{aligned} \tag{11}$$

Thus, a large magnitude of $\left|\frac{\partial f_d}{\partial x_{j,g}}\left(x_j\right)\right|$ indicates that small perturbations in feature $g$ for cell $j$ induce substantial shifts in the $d$-th embedding coordinate. To obtain a global feature-importance measure, we average these derivatives across all cells:

$$I_{d,g} = \frac{1}{N}\sum_{j=1}^{N} \left|\frac{\partial f_d}{\partial x_{j,s}}\left(x_j\right)\right| \tag{12}$$

Features $g$ with higher $I_{d,g}$ are deemed more influential in shaping dimension $d$. An identical procedure is applied in the scATAC domain using the learned projection $\beta$ and its kernel $K_Y$.

To identify key molecular drivers in each latent dimension,

we first ranked genes by their contribution scores $I_{d,g}$. The highest-ranked genes displayed significant variation in expression closely linked to the biological structure observed within the low-dimensional embedding. Gene ontology (GO) enrichment analyses conducted on these top-ranking genes using g:Profiler [20] with default parameters revealed strong enrichment for cellular metabolism-related processes.

In parallel, we investigated regulatory elements in the chromatin accessibility (ATAC) domain. Similar to the gene-ranking procedure, open chromatin peaks were ordered by their respective contribution scores for each GROTIA-derived dimension. We then identified transcription factor binding sites (TFBS) by performing de novo motif discovery on the most influential ATAC-seq peaks associated with each latent dimension using GimmeMotifs [21], applying a false discovery rate (FDR) threshold of < 0.001. This analysis yields enriched DNA sequence motifs representing putative TF binding sites in accessible chromatin and, through motif annotation, provides a set of candidate transcription factors (TFs) associated with each motif. Specifically, each de novo transcription factors binding site (TFBS) was annotated by matching its position weight matrix (PWM) to reference motif databases (including JASPAR, HOCOMOCO, CIS-BP, and ENCODE) using Pearson correlation–based similarity scores, thereby assigning candidate transcription factors (TFs) to each TFBS based on similarity to known binding preferences. We next linked individual TFBS-containing peaks to proximal genes by assigning each site to the nearest gene within a ±20 kb window [22, 23]. Finally, these candidate target genes were filtered by intersecting them with the top RNA-expressed genes associated with the same latent dimension, yielding dimension-specific TF–gene regulatory pairs.

## Co-Cluster Using Optimal Transport Plan

GROTIA also enables post-integration analysis to identify data-driven clusters. Specifically, an optimal transport (OT) plan is first computed to quantify the flow between two distinct sets of entities (e.g., cells in the RNA and ATAC spaces). We then apply a co-clustering algorithm [24] based on alternating maximization of bipartite graph modularity directly to the resulting transport matrix, thereby simultaneously grouping row and column entities. By treating the OT plan as a bipartite graph, this co-clustering approach identifies latent structural patterns that minimize within-cluster transport costs while maximizing separation between clusters. Following co-clustering, clusters containing fewer than 20 cells are pruned as noise.

## Evaluated metrics

Each alignment method was assessed in two distinct evaluation modes, each paired with two quantitative metrics. Unsupervised mode tuned its hyper-parameters solely by minimizing the model's objective function, deliberately withholding any label information. Semi-supervised mode, in contrast, selected hyper-parameters that maximized downstream cell-type classification accuracy on a held-out validation set; crucially, these labels were used only during the tuning phase and were never provided to the model as inputs, preserving the semi-supervised setup.

The first metric is Fraction of Samples Closer Than the True Match (FOSCTTM). For each sample $x_i$ in domain $X$, let the corresponding (true matched) sample in domain $Y$ be $y_i^*$. We first embed both $X$ and $Y$ into a common space via embedding functions $f_1$ and $f_2$, respectively. We then define the distance between embedded points using a distance measure $d(\cdot,\cdot)$. The FOSCTTM metric for each sample $x_i$ measures the fraction of samples in $Y$ that are closer to $x_i$ (in the embedded space) than its true match $y_i^*$. Formally,

$$R_i = \frac{1}{|Y|-1} \sum_{\substack{j\in Y \\ j\neq i}} \mathbf{1}\left\{ d\left(f_1(x_i), f_2\left(y_j\right)\right) < d\left(f_1(x_i), f_2(y_i^*)\right)\right\} \quad (13)$$

where $\mathbf{1}\{\cdot\}$ is the indicator function, returning 1 if the condition is satisfied and 0 otherwise. The FOSCTTM score for the entire dataset is the average of $R_i$ across all $x_i \in X$:

$$\text{FOSCTTM} = \frac{1}{|X|} \sum_{i=1}^{|X|} R_i \quad (14)$$

A lower FOSCTTM value indicates better alignment, as it means fewer samples in $Y$ are closer to $x_i$ than the true match $y_i^*$.

The second metric is Label transfer accuracy (LTA) and it evaluates how well cell-type (or other categorical) labels can be transferred from domain $X$ to domain $Y$ in the integrated space [6]. After embedding both datasets into a shared representation, each point $x_i \in X$ has a known label $L_X(x_i)$. We define $\text{kNN}(x_i)$ as the set of the $k$ nearest neighbors of $x_i$ in the embedded representation of $Y$. Let

$$\hat{L}(x_i) = \text{mode}\left\{L_Y(y) : y \in \text{kNN}(x_i)\right\} \quad (15)$$

where mode $(\cdot)$ returns the most common label among those $k$ neighbors. The LTA score is then computed as:

$$\text{LTA} = \frac{1}{|X|} \sum_{i=1}^{|X|} \mathbf{1}\left\{\hat{L}(x_i) = L_X(x_i)\right\} \quad (16)$$

where $\mathbf{1}(\cdot)$ is the indicator function. A higher LTA indicates that the integrated embedding preserves biological labels more accurately between the two domains.

## Training Details

We implemented GROTIA in PyTorch using the Adam optimizer. Whenever the training loss plateaued, the learning rate was reduced by a factor of 0.5. We selected the latent dimension to be either 5 or 8 and observed that GROTIA remained robust to this choice. To preserve local structure via the graph Laplacian, we set the number of nearest neighbors to 5. Three hyperparameters appear in the loss function: $\lambda_{\text{topo}}$, which emphasizes preservation of local geometry within each modality, and $\lambda_{\text{ortho}}$, which controls the strength of the orthogonality constraint. We searched over a grid of $\lambda_{\text{ortho}} \in \{1, 10^{-1}, 10^{-2}, 10^{-3}\}$ and $\lambda_{\text{topo}} \in \{10^{-3}, 10^{-4}, 10^{-5}, 10^{-6}, 10^{-7}, 10^{-8}\}$, with the constraint $\lambda_{\text{ortho}} > \lambda_{\text{topo}}$ to ensure that local structure is preserved while the mapping remains close to a projection. Additionally, we varied the `reach` parameter over $\{0.1, 1.0, 5.0\}$, where `reach` controls the degree of unbalancedness in the Sinkhorn loss. A more detailed explanation is provided in the *Appendix: Optimization Details.* All experiments were run on a single NVIDIA A100 GPU.

The input to GROTIA follows standard dataset preprocessing practices and does not require method-specific processing. For all simulated datasets, features were z-score normalized prior to alignment, following the procedure used in Liu et al. [13]. For the scGEM and SNARE-seq datasets, we downloaded the preprocessed datasets provided by Demetci et al. [6] and applied the same unit-normalization procedure described in that work. For the PBMC-1 and PBMC-2 datasets, we strictly followed the preprocessing pipelines used by the original authors of the corresponding benchmarked methods [14, 9]. Specifically, we downloaded the raw data and applied the preprocessing scripts provided by the respective authors. Consequently, all datasets were used exactly as released by

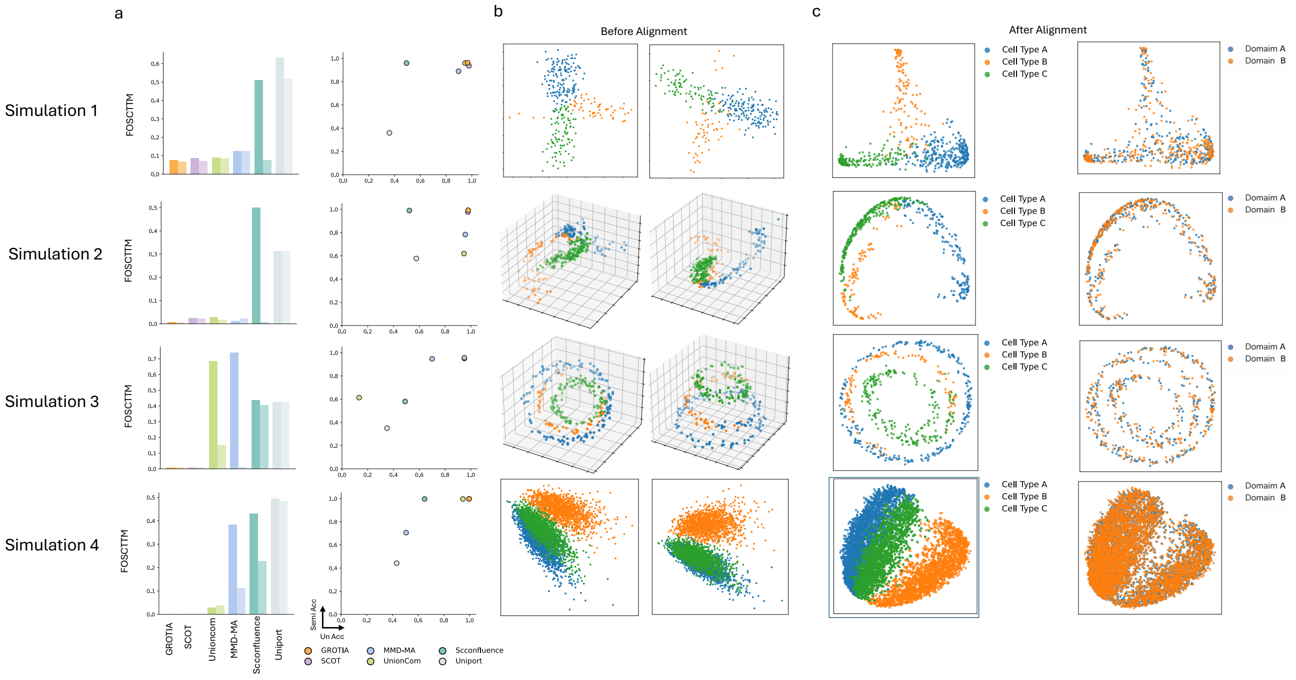

**Figure 2.** Benchmarking results on simulated datasets. a) Evaluation of Label Transfer Accuracy and Fraction of Samples Closer Than the True Match (FOSCTTM) across five benchmarked methods under two evaluation modes: semi-supervised and unsupervised. For each method, two bars are shown in the same plot, with the left bar representing the semi-supervised mode and the right bar representing the unsupervised mode for FOSCTTM. Label Transfer Accuracy is shown with semi-supervised results on the x-axis (Semi Acc) and unsupervised results on the y-axis (Un Acc). b) Visualization of simulated datasets before integration. Simulations 1 and 4 are visualized using the first two PCA components, whereas Simulations 2 and 3 are visualized using the first three axes from multidimensional scaling (MDS). c) Visualization after integration under unsupervised alignment. The first column displays data colored by cell type, while the second column shows data colored by domain. All four datasets are visualized using the first two PCA components.

the original benchmarked integration methods to ensure fair and consistent comparison, and no customized or alternative preprocessing pipelines were introduced in this work.

## Baseline Settings

To benchmark our method (GROTIA), we compared it against several existing approaches, each downloaded and configured according to the authors' guidelines. SCOT (v1.0) was obtained from Demetci et al. [6]. We provided the same PCA-preprocessed input to SCOT as to GROTIA, mirroring SCOT's original publication. We then tuned the hyperparameter based on the recommendations in the SCOT documentation.

We downloaded UnionCom (v0.4.0) and applied the same input as in GROTIA, again following the developers' suggested preprocessing steps. All hyperparameters were tuned according to the guidance provided in the UnionCom package.

For UniPort (v1.3), which supports diagonal integration (i.e., mode=d ) to align datasets without common genes, we used 2,000 highly variable genes from the scRNA-seq data and peaks exceeding a threshold of 1 for the scATAC-seq data. We also employed TF-IDF normalization, replicating the tutorial steps outlined by the UniPort authors. We obtained the MMD-MA (v1.0) PyTorch implementation from Singh et al. [25]. As with GROTIA and SCOT, we provided PCA-preprocessed data and tuned its hyperparameters in accordance with the authors' guidelines.

Lastly, we downloaded scConfluence (v0.1.1). For the version without prior information, we set $\lambda_{IOT} = 0$, forcing a diagonal integration approach. When using scConfluence with prior information, we followed the recommended settings from the authors. We also tuned the remaining hyperparameters according to their instructions, applying identical preprocessing to ensure fair comparisons across all methods. For the four simulated datasets as well as the scGEM and SNARE-seq datasets, the construction of a cross-modality distance matrix is non-trivial. Specifically, the simulated datasets lack meaningful cross-modal feature definitions, and computing such distances for scGEM and SNARE-seq is not feasible.

## Use of Large-Language Models

We used OpenAI ChatGPT only for grammar, spelling, and stylistic refinement of the manuscript. The model did not generate or alter any scientific content, data analysis, interpretations, or conclusions. All AI suggestions were manually reviewed and either accepted or rejected by the authors.

# Results

## GROTIA integrated simulated datasets in both semi and unsupervised setting

We evaluated the GROTIA algorithm using four simulated datasets previously discussed. Performance of the GROTIA algorithm was compared against five benchmarked methods: SCOT, Unioncom, MMD-MA, and two VAE-based approaches, Scconfluence and Uniport. Evaluations were conducted under two scenarios: semi-supervised (partial label information available) and unsupervised (no label information available).

In Figure 2a, the left column (solid color) and right column (vertical axis) display FOSCTTM and label transfer accuracy, respectively, under the semi-supervised setting. In the semi-supervised scenario, GROTIA and SCOT demonstrated consistently high performance across all datasets, with Unioncom and MMD-MA closely following. Scconfluence and Uniport showed comparatively lower performance, possibly due to their reliance on cross-modality guidance, which was not available in these simulations.

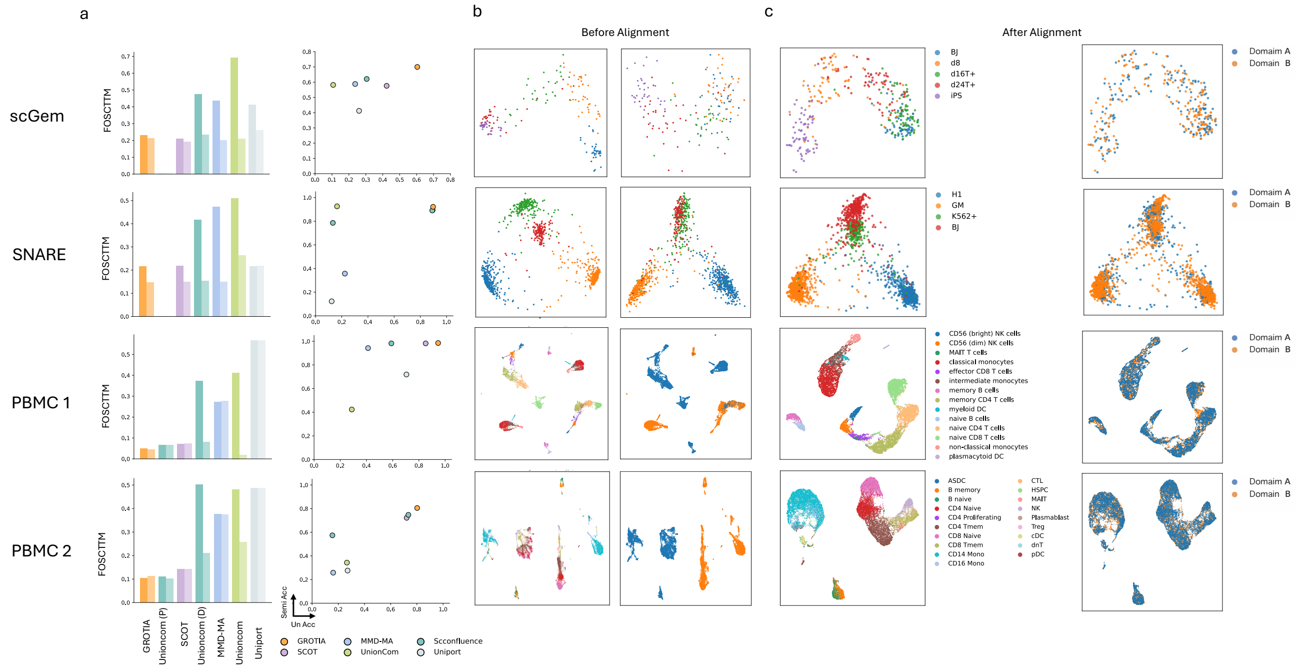


**Figure 3.** Benchmarking results on real word datasets. a) Evaluation of Label Transfer Accuracy and Fraction of Samples Closer Than the True Match (FOSCTTM) across five benchmarked methods under two evaluation modes: semi-supervised and unsupervised. For each method, two bars are shown in the same plot, with the left bar representing the semi-supervised mode and the right bar representing the unsupervised mode for FOSCTTM. Label Transfer Accuracy is shown with semi-supervised results on the x-axis (Semi Acc) and unsupervised results on the y-axis (Un Acc). b) Visualization of real word datasets before integration. ScGem and SNARE are visualized using the first two PCA components, whereas PBMC 1 and PBMC 2 are visualized using the first two UMAP Components. c) Visualization after integration under unsupervised alignment. The first column displays data colored by cell type, while the second column shows data colored by domain. ScGem and SNARE are visualized using the first two PCA components, whereas PBMC 1 and PBMC 2 are visualized using the first two UMAP Components.

In Figure 2a, the left column (light color) and right column (horizontal axis) display FOSCTTM and label transfer accuracy, respectively, under the unsupervised setting. In the unsupervised scenario, where alignment depends solely on intrinsic structural information, GROTIA and SCOT maintained relatively stable performance. Other methods showed varying degrees of accuracy reduction, notably Unioncom on dataset 3, MMD-MA on datasets 3 and 4, Scconfluence on datasets 1, 2, and 4, and Uniport on dataset 1. These variations highlight the challenges inherent in unsupervised alignment without label guidance.

In Figure 2b, the datasets from each domain are shown prior to integration. The left column corresponds to one modality and the right column to the other modality. Figure 2c shows the integrated datasets, colored by cell type on the left and by domain on the right. These results demonstrate that GROTIA effectively aligns the two modalities, with cells of the same type from different modalities well matched and the two domains properly overlapping in the integrated space.

The stable performance of GROTIA can be attributed to their effective use of Wasserstein-based (WD) losses, capturing intrinsic geometric structures. Additionally, GROTIA employs orthogonality constraints within the Reproducing Kernel Hilbert Space (RKHS), enhancing embedding stability. Although VAE-based methods also utilize WD losses, their embeddings can be susceptible to rotational variations, affecting alignment stability in the absence of label information. Detailed numerical results corresponding to the metric plots are presented in Supplementary Tables A2-A5.

## GROTIA integrated real word datasets in both semi and unsupervised setting

We then evaluated the GROTIA algorithm on four real-world datasets. We chose these paired multi-omics datasets specifically to enable diagonal integration with known ground-truth cell correspondences. Importantly, during benchmarking, all methods are provided with unpaired data, and the known cell-pairing information was used only for evaluating alignment accuracy.

The benchmarking process used here follows the same procedure described in the simulation study, with one modification: for the PBMC-1 and PBMC-2 datasets, we additionally include scConfluence with prior information. We do not include the prior version of scConfluence for the scGEM and SNARE datasets because the original authors performed dimensionality reduction to process these data, meaning commonly used gene selection methods are not available and the resulting feature space is small.

In Figure 3a, the left column (solid color) and right column (vertical axis) display FOSCTTM and label transfer accuracy, respectively, under the semi-supervised setting. Across the scGEM and SNARE-seq datasets, GROTIA achieves the second-best FOSCTTM in scGEM and the best in SNARE-seq, while attaining the highest label transfer accuracy in both. SCOT obtains the top FOSCTTM in scGEM but ranks second in the remaining evaluations. scConfluence (default), MMD-MA, UnionCom, and UniPort follow in overall performance. For the two PBMC datasets, GROTIA places second in FOSCTTM for both PBMC-1 and PBMC-2; in PBMC-2, scConfluence with prior information attains the best FOSCTTM. Regarding label transfer accuracy, GROTIA ranks second in PBMC-1 and first in PBMC-2.

Under the fully unsupervised setting, shown by lighter colors in the left column of Figure 3a (and the right column's horizontal axis for label transfer accuracy), GROTIA demonstrates the strongest overall performance in both FOSCTTM and label transfer accuracy across all four real-world datasets, except for ranking second in FOSCTTM on scGEM. SCOT and scConfluence exhibit comparable results, followed by UnionCom, MMD-MA, and UniPort. Detailed numerical results corresponding to the metric plots are presented in Supplementary Tables A6-A9.

In Figure 3b, each modality is visualized separately prior to integration, with the left column showing one domain and the right

column showing the other. Figure 3c presents the integrated representation, colored by cell type on the left and by domain on the right. The results indicate that GROTIA successfully aligns the two modalities, bringing cells of the same type from different domains into close correspondence while achieving strong overlap between modalities in the integrated space.

## GROTIA Reveals Gene-Specific Contributions and Key Biological Processes in the RNA Embedding

GROTIA provides an in-model measure of gene importance, pinpointing which genes drive variation along each latent dimension. The PBMC-1 dataset is used to demonstrate the following experiments. Specifically, we compute partial derivatives of the RBF kernel embeddings with respect to each gene's expression and then weight these by the projection matrix to obtain a contribution score for every gene–dimension pair. Ranking these scores identifies dimension-specific "signature genes"—those whose expression changes most strongly reposition cells in the low-dimensional space. For additional details, see Section Interpretable Embeddings of GROTIA.

Figure 4a displays the overall gene importance scores across all eight GROTIA-derived dimensions. Figure 4b shows UMAP projections of the scRNA-seq data, with gene expression overlaid for genes selected based on their importance in Dimensions 1 and 3 of the learned embedding, illustrating how high-impact genes are distributed across the dataset. For Dimension 1, the top three contributing genes (LYZ, ZEB2, PLXDC2) are highly expressed in monocytes and myeloid cell populations, consistent with further differentiation within the monocyte lineage[26]. Moreover, Dimension 3 emphasizes GNLY, CCL5, and LEF1—genes enriched in NK cells and T cells, indicating a T cell–specific transcriptional program [26]. Notably, GROTIA requires no a priori matching of features across modalities, so these dimension-specific drivers offer an data-driven method to uncover potential marker genes.

To link the top contributors in Dimension 1 to biological processes, we performed Gene Ontology (GO) enrichment analysis, as shown in figure Fig. 4c. These genes were enriched for the terms RNA polymerase II-specific DNA-binding transcription factor binding, β-catenin binding, and peptide binding. The first term suggests that Dimension 1 captures a transcriptional regulatory program active during monocyte and dendritic cell differentiation, involving lineage-defining regulators such as ZEB2, which is essential for monocyte and plasmacytoid DC development [27]. The second highest-ranked term, β-catenin binding, indicates involvement of Wnt/β-catenin signaling in monocyte and DC biology; for instance, β-catenin activation fosters a tolerogenic phenotype in DCs [28], while its aberrant stabilization can obstruct normal monocyte–macrophage differentiation [29]. Finally, enrichment for peptide binding aligns with the antigen processing and presentation roles of monocytes and DCs, consistent with elevated HLA-DR expression in intermediate monocytes [30]. For additional UMAP distributional plots of top genes, see Supplementary Figures A.1 and A.2.

In Dimension 3 of the PBMC transcriptional analysis, we observed GO term enrichment related to immune receptor activity, antigen binding, and calcium-mediated signaling. This suggests that Dimension 3 captures variation in lymphocyte receptor expression and signaling. The GO category immune receptor activity is associated primarily with T lymphocytes, which uniquely express the T-cell receptor complex (for example, CD3 subunits and TCR α/β chains) mediating antigen-specific recognition [31]. Moreover, antigen binding reflects the high expression of immunoglobulin genes by B cells, consistent with their exclusive role in producing antigen-specific antibodies [32]. Finally, calcium-mediated signaling highlights a key activation pathway in T cells, where antigen-receptor engagement triggers $Ca^{2+}$ influx through store-operated $Ca^{2+}$ channels to activate downstream effectors such as the calcineurin–NFAT pathway, a process essential for lymphocyte activation [33]. For additional Gene Ontology enrichment results corresponding to the remaining dimensions, see Supplementary Tables A10–A15.

## GROTIA Identifies High-Impact Peaks and Regulatory Mechanisms in the ATAC Embedding

Similarly, in the ATAC domain, we ranked open chromatin peaks by their gradient-based contribution scores and performed motif discovery on the highest-impact peaks (see Methods). The PBMC-1 dataset is used to demonstrate the following experiments. Mapping each motif to its nearest gene within a 20 kb window revealed putative regulatory relationships linking epigenetic accessibility to transcriptional output. Figure 5a illustrates the procedure for identifying transcription factor–gene pairs from the top-ranked peaks. For further details, please refer to Section Interpretable Embedding of GROTIA.

Figure 5b highlights the top-ranked peaks (based on partial derivative–based importance) across the eight GROTIA-derived dimensions (D1–D8), with the most influential peaks in each dimension outlined in red boxes. Figure 5c shows the inferred transcription factor–gene pairs associated with each dimension. Finally, Figure 5d presents a UMAP projection of the ATAC embedding, overlaid with the identified TF–gene pairs, visually illustrating potential co-expression or repression relationships. In dimension 1, we identified a CEBPB–KLF4 pair. CEBPB is essential for proper monocyte development, including the survival of certain subsets, and likely induces KLF4 as part of the monocyte differentiation network. Indeed, PU.1 (encoded by SPI1) directly upregulates KLF4, and CEBPB cooperates with PU.1 to drive monopoiesis [34]. In dimension 2, the IRF8–CST3 pair emerged. IRF8 directly activates CST3 (cystatin C) during macrophage differentiation, mediated by a unique promoter element that overlaps IRF and ETS sites and requires both IRF8 and an ETS partner (e.g., PU.1) [35]. Dimension 3 highlighted TBX21 (T-bet)–CCL4, wherein T-bet binds to and positively regulates CCL4 in Th1 cells. Genome-wide ChIP-chip experiments in human T cells confirmed CCL4 as a direct T-bet target, revealing T-bet binding in regulatory regions that activate CCL4 transcription [36]. In dimension 5, we found IRF8–CLEC7A. Genome-wide binding studies have identified CLEC7A as an IRF8 target in human myeloid cells [37], and co-expression networks further link CLEC7A with an IRF8-centered module[38]. Consistently, aging human microglia upregulate CLEC7A alongside other "activated" microglial genes under the control of an IRF8/SPI1 (PU.1)/RUNX1/TAL1 network [39]. In dimension 6, the SPI1(PU.1)–IFITM3 pair indicates that PU.1 controls a broad antiviral gene program in macrophages, with IFITM3 explicitly citetd as a PU.1-regulated antiviral factor [40]. Dimension 7 highlighted RUNX2–GPR183. Finally, in dimension 8, the PAX5–FAM49A pair was supported by evidence of PAX5 ChIP-seq peaks near FAM49A [41]. Notably, FAM49A is expressed at lower levels in PAX5-positive pro–B cells and is de-repressed in PAX5-deficient cells, indicating that PAX5 normally suppresses Fam49a expression during early B-cell development [42].

## GROTIA enables identification of cellular subpopulation on integrated space

Beyond simply projecting scRNA and scATAC profiles into a shared space, GROTIA provides a natural framework for discovering subpopulations in an unsupervised manner. The PBMC-1 dataset is used to demonstrate the following experiments. While many datasets come with predefined labels (e.g., annotated cell types), these annotations may be incomplete or coarse, especially when new subtleties or states exist that were not recognized during initial labeling. By clustering cells in GROTIA's integrated latent space, we

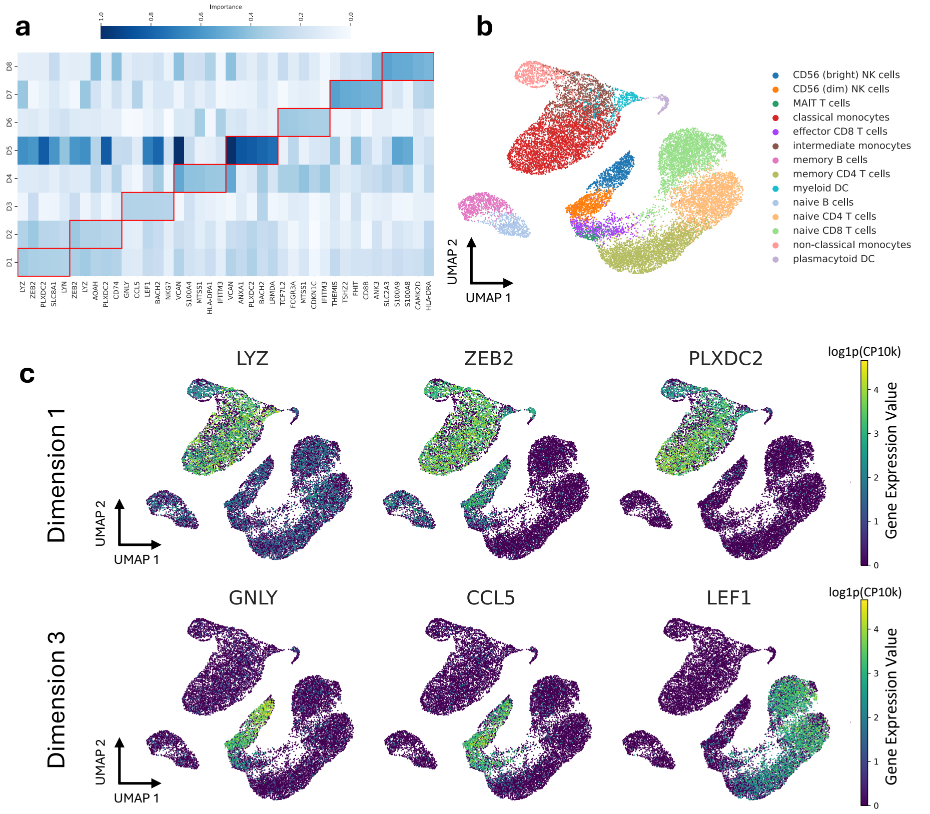


**d**

Dimension 1

| Source | GO name | GO ID | P value | term size | Inter. size |
|---|---|---|---|---|---|
| **GO:MF** | RNA polymerase II-specific DNA-binding transcription factor binding | GO:0061629 | $2.632 \times 10^{-3}$ | 348 | 14 |
| **GO:MF** | beta-catenin binding | GO:0008013 | $1.076 \times 10^{-2}$ | 89 | 7 |
| **GO:MF** | peptide binding | GO:0042277 | $1.455 \times 10^{-2}$ | 254 | 11 |
| **GO:MF** | GTPase regulator activity | GO:0030695 | $3.364 \times 10^{-2}$ | 495 | 15 |
| **GO:BP** | toll-like receptor signaling pathway | GO:0002224 | $7.559 \times 10^{-3}$ | 75 | 7 |
| **GO:BP** | response to peptide hormone | GO:0043434 | $1.164 \times 10^{-2}$ | 434 | 15 |
| **GO:BP** | cell killing | GO:0001906 | $4.250 \times 10^{-2}$ | 219 | 10 |
| **GO:CC** | trans-Golgi network membrane | GO:0032588 | $1.121 \times 10^{-2}$ | 107 | 7 |
| **GO:CC** | collagen-containing extracellular matrix | GO:0062023 | $2.299 \times 10^{-2}$ | 425 | 13 |
| **GO:CC** | actin filament | GO:0005884 | $2.457 \times 10^{-2}$ | 121 | 7 |

Dimension 3

| Source | GO name | GO ID | P value | term size | Inter. size |
|---|---|---|---|---|---|
| **GO:MF** | immune receptor activity | GO:0140375 | $5.973 \times 10^{-6}$ | 145 | 12 |
| **GO:MF** | antigen binding | GO:0003823 | $2.142 \times 10^{-2}$ | 175 | 9 |
| **GO:BP** | calcium-mediated signaling | GO:0019722 | $7.420 \times 10^{-3}$ | 218 | 11 |
| **GO:BP** | viral process | GO:0016032 | $1.169 \times 10^{-2}$ | 427 | 15 |
| **GO:CC** | membrane raft | GO:0045121 | $2.669 \times 10^{-3}$ | 291 | 12 |
| **GO:CC** | COPII-coated ER to Golgi transport vesicle | GO:0030134 | $3.667 \times 10^{-2}$ | 89 | 6 |

**Figure 4.** (a) Heatmap of partial derivative–based importance scores for the top five genes in each of the eight GROTIA-derived dimensions (D1–D8). Darker blue indicates higher importance. The top five genes per dimension are outlined in the red box. (b) UMAP projections of Dimension 1 and Dimension 3, highlighting cell-type annotations (top) and the expression distributions of top-ranked genes (middle and bottom). Warmer hues denote higher expression, revealing distinct cellular subsets for Dimension 1 and Dimension 3. (c) Summaries from the Gene Ontology experiment, showing significant enrichment grouped by Molecular Function (MF), Cellular Component (CC), and Biological Process (BP). Collectively, these panels show how GROTIA's dimension-wise interpretability links high-impact genes to key biological functions.

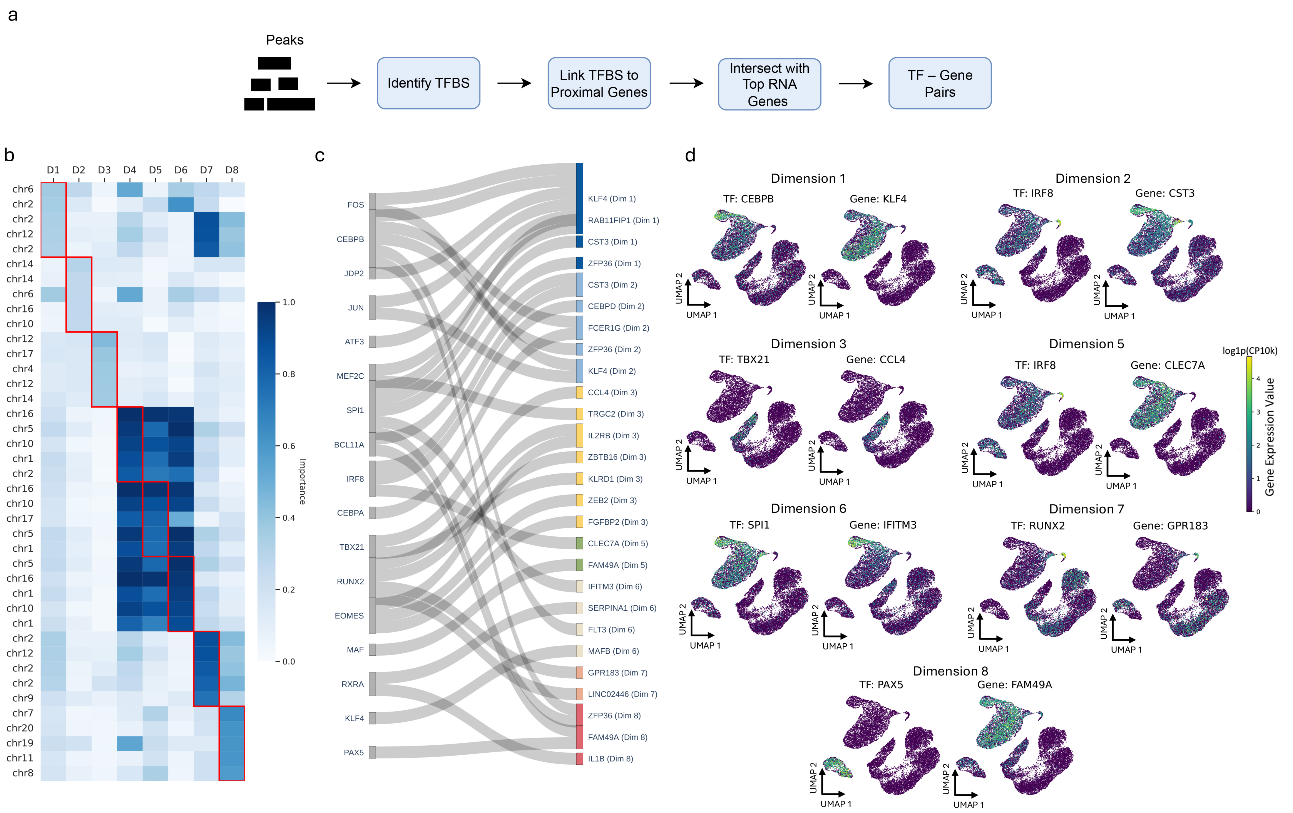


**Figure 5.** (a) Schematic of the workflow for identifying significant motifs in open chromatin peaks, mapping these motifs to nearby genes, and intersecting them with top RNA space genes to form motif–gene pairs. (b) Heatmap of gradient-based importance scores for the five most influential ATAC peaks in each GROTIA-derived dimension (D1–D8), where darker blue indicates greater importance. (c) Sankey diagram illustrating dimension-specific gene–factor pairs. Genes (left) connect to their putative transcription factors (right), validated through literature. For instance, FOS are implicated as potential regulators of gene KFL4 in Dimensions 1 and CEBPB as potential regulators of Gene FCR1G in dimension 2. (d) UMAP embeddings colored by gene expression values of selected gene–factor pairs from Dimensions 1, 3, 5, 6, 7, and 8. Warmer hues denote higher expression, highlighting dimension-specific regulatory landscapes. Co-expression patterns further support these putative regulatory relationships.

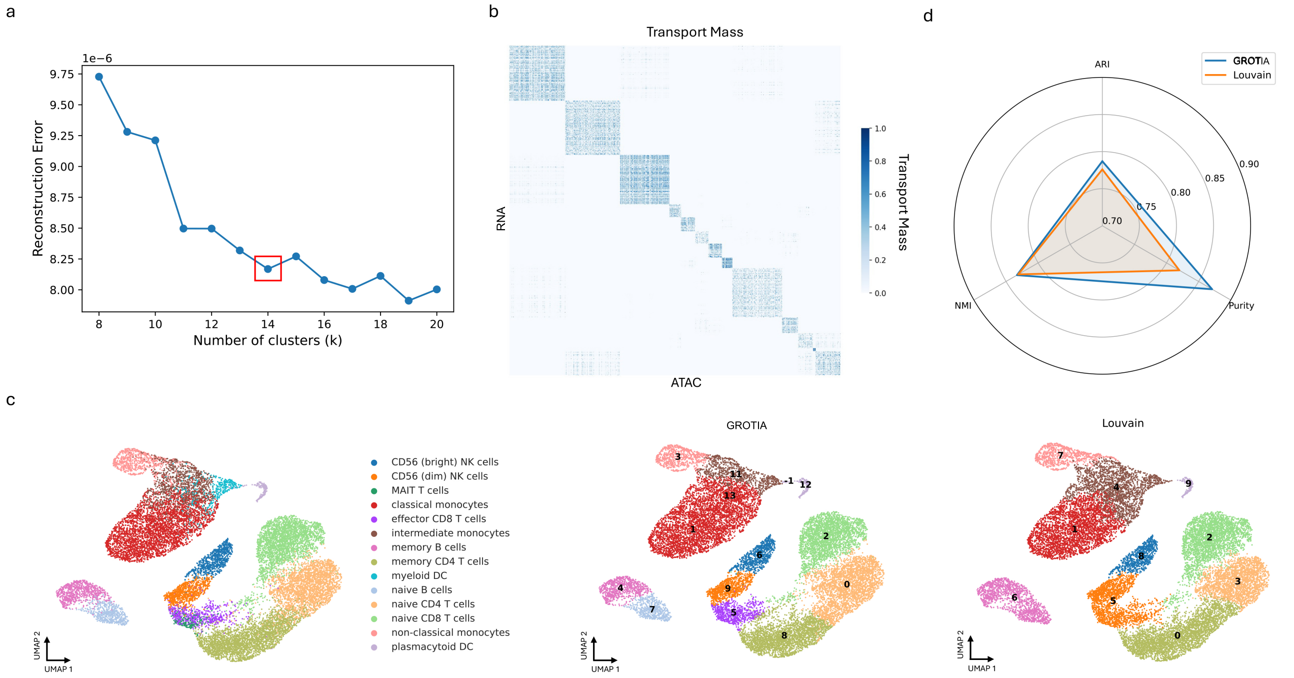


**Figure 6.** (a) Reconstruction error plotted against the number of clusters *k*, with the chosen *k* marked by a red box. This optimal *k* balances clustering granularity and data fidelity. (b) Heatmap of the transport mass after co-clustering, illustrating how GROTIA aligns cells from scRNA and scATAC. The block-diagonal pattern indicates coherent groupings across both modalities. (c) UMAP projections of the integrated dataset, colored by true cell-type annotations (left), and clustered with GROTIA (center) or Louvain (right). For each method, predicted clusters are labeled by the ground-truth cluster with which they most overlap. GROTIA produces distinct subpopulations consistent with known cell-type boundaries. (d) Radar plot comparing GROTIA (orange) and Louvain (blue) on three clustering metrics (ARI, NMI, and Purity). GROTIA demonstrates higher or comparable performance, indicating its ability to robustly identify meaningful subpopulations.

recover cell population structure that closely matches expert annotations and enable inference of corresponding cell labels in a second modality when annotations are available in only one modality.

To determine the optimal number of clusters $k$, we plot the reconstruction error for various $k$-values and look for a distinct elbow (Fig. 6a). Beyond this point, further increasing $k$ offers minimal improvement in accuracy while potentially fragmenting biologically coherent groups. We therefore select the $k$ at the bump, yielding a robust trade-off between clustering granularity and data fidelity. We then visualize the resulting assignments alongside the original cell-type labels on a UMAP projection (Fig. 6c). Notably, GROTIA identifies distinct subpopulations that align well with known major cell types, yet can also isolate refined subclusters reflecting subtle transcriptional and epigenetic differences.

Finally, to benchmark the quality of GROTIA's partitioning, we compare against a conventional community detection algorithm (Louvain) using three standard clustering metrics: Adjusted Rand Index (ARI), Normalized Mutual Information (NMI), and Purity (Fig. 6d). We tuned the resolution parameter over the grid resolution $\in \{0.1, 0.3, 0.4, 0.5, 0.6, 1.0, 1.5\}$ and selected the value that maximized the sum of ARI, NMI, and Purity with respect to the ground truth cell type labels on that dataset.

Our method achieves comparable or better performance, demonstrating that GROTIA's alignment-based approach not only reconciles multi-omic data but also preserves biologically meaningful structures when clustering.

## Discussion

We present GROTIA, a novel algorithm for unsupervised single-cell multi-omics integration that combines optimal transport for global alignment with graph regularization to preserve local structure. Benchmarking against state-of-the-art methods in both unsupervised and semi-supervised settings, GROTIA delivers comparable or superior performance while offering a computationally efficient solution (see *Appendix: Metric Performance*). Critically, our framework also includes an in-model interpretability mechanism, allowing users to identify which genes or peaks drive each dimension of the integrated embedding. This enables targeted downstream analyses—such as Gene Ontology enrichment or motif discovery—to reveal meaningful biological processes in the RNA and ATAC spaces.

Beyond aligning multi-omics data, GROTIA leverages its transport plan for post-integration clustering, offering a data-driven approach to refine or correct misannotations in existing labels. Notably, unlike methods that require shared features across modalities, GROTIA only assumes that cells (rather than individual genes or peaks) follow a similar distribution if they belong to the same type or lineage—thus broadening its applicability to complex datasets.

Looking ahead, we plan to extend GROTIA to time-series multi-omics data, where paired measurements across multiple time points are becoming increasingly common. Furthermore, we will deepen our driver-gene analyses to decode how specific features shape the integrated embedding, with the goal of uncovering more nuanced regulatory processes across different cell states. Also, extending GROTIA with kernel approximations (e.g., Nyström or random features) or with mini-batch and sparse variants to reduce memory usage is an important direction for future work.

## Availability of source code and requirements

Lists the following:

- Project name: GROTIA.
- Project home page: `https://github.com/PennShenLab/GROTIA`.
- Operating system(s): Platform independent.
- Programming language: Python 3.8 or higher.
- License: MIT License.
- Package management: GitHub.
- Hardware requirements: CPU required; GPU optional.
- RRID:SCR_027088.

## Data Availability

All additional supporting data are available in the GigaScience repository, GigaDB [43]. Machine learning annotations have been deposited in the DOME registry[44].

## Competing Interests

The authors declared no potential conflicts of interest with respect to the research, authorship, and/or publication of this article.

## List of abbreviations

Fraction of Samples Closer Than the True Match (FOSCTTM); Gene Ontology (GO); Generalized Unsupervised Manifold Alignment (GUMA); GROTIA (Graph-Regularized Optimal Transport Framework for Diagonal Single-Cell Integrative Analysis); Label Transfer Accuracy (LTA); Maximum Mean Discrepancy (MMD); Peripheral Blood Mononuclear Cell (PBMC); Single-Cell Multi-Omics Alignment with Optimal Transport (SCOT); Transcription Factor (TF); Transcription Start Sites (TSS); Unsupervised Topological Alignment for Single-Cell Multi-Omics Integration (UnionCom); Wasserstein-based (WD).

## Funding

This work is supported in part by NIH Grants R01 AG071470, U19 AG074879, U01 AG066833 and U01 AG068057.

## Author contributions statement

Conceptualization, Z.W., Q.Z., M.K., and L.S.; Methodology, Z.W., Q.Z., and L.S.; Resources, L.S.; Formal analysis, Z.W., Q.Z., S.Y., Z.Z., M.K., T.Z., and L.S.; Writing-Original Draft, Z.W., Q.Z., Z.Z., and L.S.; Funding acquisition, L.S.; Writing-Review and Editing, Z.W., Q.Z., S.Y., Z.Z., M.K., T.Z., and L.S.

## References

1. Heumos L, Schaar AC, Lance C, Litinetskaya A, Drost F, Zappia L, et al. Best practices for single-cell analysis across modalities. Nature Reviews Genetics 2023;24(8):550–572.
2. Haque A, Engel J, Teichmann SA, Lönnberg T. A practical guide to single-cell RNA-sequencing for biomedical research and clinical applications. Genome medicine 2017;9:1–12.
3. Grandi FC, Modi H, Kampman L, Corces MR. Chromatin accessibility profiling by ATAC-seq. Nature protocols 2022;17(6):1518–1552.
4. Li B, Nabavi S. scGEMOC, A Graph Embedded Contrastive Learning Single-cell Multiomics Clustering Model. In: 2023 IEEE International Conference on Bioinformatics and Biomedicine (BIBM) IEEE; 2023. p. 2075–2080.
5. Huizing GJ, Deutschmann IM, Peyré G, Cantini L. Paired single-cell multi-omics data integration with Mowgli. Nature Communications 2023;14(1):7711.
6. Demetci P, Santorella R, Sandstede B, Noble WS, Singh R. SCOT: single-cell multi-omics alignment with optimal transport. Journal of computational biology 2022;29(1):3–18.

7. Cao K, Bai X, Hong Y, Wan L. Unsupervised topological alignment for single-cell multi-omics integration. Bioinformatics 2020;36(Supplement_1):i48–i56.
8. Amodio M, Krishnaswamy S. MAGAN: Aligning biological manifolds. In: International conference on machine learning PMLR; 2018. p. 215–223.
9. Cao K, Gong Q, Hong Y, Wan L. A unified computational framework for single-cell data integration with optimal transport. Nature Communications 2022;13(1):7419.
10. Barkas N, Petukhov V, Nikolaeva D, Lozinsky Y, Demharter S, Khodosevich K, et al. Joint analysis of heterogeneous single-cell RNA-seq dataset collections. Nature methods 2019;16(8):695–698.
11. Halpern KB, Shenhav R, Massalha H, Toth B, Egozi A, Massasa EE, et al. Paired-cell sequencing enables spatial gene expression mapping of liver endothelial cells. Nature biotechnology 2018;36(10):962–970.
12. Cui Z, Chang H, Shan S, Chen X. Generalized unsupervised manifold alignment. Advances in Neural Information Processing Systems 2014;27.
13. Liu J, Huang Y, Singh R, Vert JP, Noble WS. Jointly embedding multiple single-cell omics measurements. In: Algorithms in bioinformatics:... International Workshop, WABI..., proceedings. WABI (Workshop), vol. 143 NIH Public Access; 2019. .
14. Samaran J, Peyré G, Cantini L. scConfluence: single-cell diagonal integration with regularized Inverse Optimal Transport on weakly connected features. Nature Communications 2024;15(1):7762.
15. Zappia L, Phipson B, Oshlack A. Splatter: simulation of single-cell RNA sequencing data. Genome biology 2017;18(1):174.
16. Cheow LF, Courtois ET, Tan Y, Viswanathan R, Xing Q, Tan RZ, et al. Single-cell multimodal profiling reveals cellular epigenetic heterogeneity. Nature methods 2016;13(10):833–836.
17. Chen S, Lake BB, Zhang K. High-throughput sequencing of the transcriptome and chromatin accessibility in the same cell. Nature biotechnology 2019;37(12):1452–1457.
18. Bravo González-Blas C, Minnoye L, Papasokrati D, Aibar S, Hulselmans G, Christiaens V, et al. cisTopic: cis-regulatory topic modeling on single-cell ATAC-seq data. Nature methods 2019;16(5):397–400.
19. Feydy J, Séjourné T, Vialard FX, Amari Si, Trouvé A, Peyré G. Interpolating between optimal transport and mmd using sinkhorn divergences. In: The 22nd International Conference on Artificial Intelligence and Statistics PMLR; 2019. p. 2681–2690.
20. Kolberg L, Raudvere U, Kuzmin I, Adler P, Vilo J, Peterson H. g: Profiler—interoperable web service for functional enrichment analysis and gene identifier mapping (2023 update). Nucleic acids research 2023;51(W1):W207–W212.
21. Bruse N, Heeringen SJv. GimmeMotifs: an analysis framework for transcription factor motif analysis. BioRxiv 2018;p. 474403.
22. Kan M, Diwadkar AR, Shuai H, Joo J, Wang AL, Ong MS, et al. Multiomics analysis identifies BIRC3 as a novel glucocorticoid response–associated gene. Journal of Allergy and Clinical Immunology 2022;149(6):1981–1991.
23. Diwadkar AR, Kan M, Himes BE. Facilitating analysis of publicly available ChIP-Seq data for integrative studies. In: AMIA Annual Symposium Proceedings, vol. 2019; 2020. p. 371.
24. Role F, Morbieu S, Nadif M. Coclust: a python package for co-clustering. Journal of Statistical Software 2019;88:1–29.
25. Singh R, Demetci P, Bonora G, Ramani V, Lee C, Fang H, et al. Unsupervised manifold alignment for single-cell multi-omics data. In: Proceedings of the 11th ACM International Conference on Bioinformatics, Computational Biology and Health Informatics; 2020. p. 1–10.
26. Uhlén M, Fagerberg L, Hallström BM, Lindskog C, Oksvold P, Mardinoglu A, et al. Tissue-based map of the human proteome. Science 2015;347(6220):1260419.
27. Wu X, Briseño CG, Grajales-Reyes GE, Haldar M, Iwata A, Kretzer NM, et al. Transcription factor Zeb2 regulates commitment to plasmacytoid dendritic cell and monocyte fate. Proceedings of the National Academy of Sciences 2016;113(51):14775–14780.
28. Swafford D, Manicassamy S. Wnt signaling in dendritic cells: its role in regulation of immunity and tolerance. Discovery medicine 2015;19(105):303.
29. Sheng Y, Ju W, Huang Y, Li J, Ozer H, Qiao X, et al. Activation of wnt/β-catenin signaling blocks monocyte–macrophage differentiation through antagonizing PU. 1-targeted gene transcription. Leukemia 2016;30(10):2106–2109.
30. Lee J, Tam H, Adler L, Ilstad-Minnihan A, Macaubas C, Mellins ED. The MHC class II antigen presentation pathway in human monocytes differs by subset and is regulated by cytokines. PloS one 2017;12(8):e0183594.
31. Jones N, Vincent EE, Cronin JG, Panetti S, Chambers M, Holm SR, et al. Akt and STAT5 mediate naïve human CD4+ T-cell early metabolic response to TCR stimulation. Nature Communications 2019;10(1):2042.
32. Li K, Zhang C, Zhou R, Cheng M, Ling R, Xiong G, et al. Single cell analysis unveils B cell-dominated immune subtypes in HNSCC for enhanced prognostic and therapeutic stratification. International Journal of Oral Science 2024;16(1):29.
33. Vig M, Kinet JP. Calcium signaling in immune cells. Nature immunology 2009;10(1):21–27.
34. Feinberg MW, Wara AK, Cao Z, Lebedeva MA, Rosenbauer F, Iwasaki H, et al. The Kruppel-like factor KLF4 is a critical regulator of monocyte differentiation. The EMBO journal 2007;26(18):4138–4148.
35. Tamura T, Thotakura P, Tanaka TS, Ko MS, Ozato K. Identification of target genes and a unique cis element regulated by IRF-8 in developing macrophages. Blood 2005;106(6):1938–1947.
36. Jenner RG, Townsend MJ, Jackson I, Sun K, Bouwman RD, Young RA, et al. The transcription factors T-bet and GATA-3 control alternative pathways of T-cell differentiation through a shared set of target genes. Proceedings of the National Academy of Sciences 2009;106(42):17876–17881.
37. Diamant I, Clarke DJ, Evangelista JE, Lingam N, Ma'ayan A. Harmonizome 3.0: integrated knowledge about genes and proteins from diverse multi-omics resources. Nucleic Acids Research 2025;53(D1):D1016–D1028.
38. Wehrspaun CC, Haerty W, Ponting CP. Microglia recapitulate a hematopoietic master regulator network in the aging human frontal cortex. Neurobiology of Aging 2015;36(8):2443–e9.
39. Yeh H, Ikezu T. Transcriptional and epigenetic regulation of microglia in health and disease. Trends in molecular medicine 2019;25(2):96–111.
40. Virgilio MC, Ramnani B, Chen T, Disbennett WM, Lubow J, Welch JD, et al. HIV-1 Vpr combats the PU. 1-driven antiviral response in primary human macrophages. Nature Communications 2024;15(1):5514.
41. Stelzer G, Rosen N, Plaschkes I, Zimmerman S, Twik M, Fishilevich S, et al. The GeneCards suite: from gene data mining to disease genome sequence analyses. Current protocols in bioinformatics 2016;54(1):1–30.
42. Silberstein L, Goncalves KA, Kharchenko PV, Turcotte R, Kfoury Y, Mercier F, et al. Proximity-based differential single-cell analysis of the niche to identify stem/progenitor cell regulators. Cell stem cell 2016;19(4):530–543.
43. Wang Z, Zhan Q, Yang S, Zhou Z, Kan M, Zhai T, et al., Supporting data for "An Interpretable Graph-Regularized Optimal Transport Framework for Diagonal Single-Cell Integrative Analysis". GigaScience Database; 2026. https://doi.org/10.5524/102801.
44. Zexuan Wang SYZZMKTZ Qipeng Zhan, Shen L. An Interpretable Graph-Regularized Optimal Transport Framework for Diagonal Single-Cell Integrative Analysis. GigaScience 2025;https://registry.dome-ml.org/review/79ooxhtwjr.

# Appendix

This appendix includes supplementary materials for "*An Interpretable Graph-Regularized Optimal Transport Framework for Diagonal Single-Cell Integrative Analysis*" by Zexuan Wang, Qipeng Zhan, Shu Yang, Zhuoping Zhou, Mengyuan Kan, Tianhua Zhai and Li Shen.

## Additional Datasets

To evaluate robustness under unbalanced cell population settings, we considered a benchmark scenario in which one shared cell population is entirely missing from one modality. Specifically, we used a paired scRNA–scATAC dataset in which the scRNA-seq modality contains HeLa and HCT cell lines, while the scATAC-seq modality contains HeLa, HCT, and K562 cell lines. This setup creates an unbalanced integration scenario with mosaic structure and missing observations across modalities, mimicking realistic diagonal integration settings. Full details of the dataset could be found at Samaran et al. [14].

Quantitative results on the cell-line dataset demonstrate that GROTIA is robust to variability in cell population proportions ( A3, A5, A7, A9). In the unsupervised setting, GROTIA achieves the highest label-transfer accuracy (0.954), slightly outperforming the next best method, UnionCom (0.947). Its FOSCTTM score (0.318) is the second best and close to the best score achieved by UnionCom (0.287), indicating competitive alignment quality without supervision.

In the semi-supervised setting, GROTIA attains a label-transfer accuracy of 0.985, matching the best-performing methods (MMD-MA and scConfluence with prior, both 0.985). Its FOSCTTM score (0.323) is also comparable to other strong baselines (0.280 for UnionCom and 0.272 for scConfluence with prior). Overall, these results show that even when the two modalities differ in feature space and cell-type composition, GROTIA reliably recovers shared cell-state structure without requiring shared features, while appropriately handling cell populations present in only one modality.

## Metric performance

**Table A1.** Computational performance of benchmarked methods on the PBMC-1 dataset (9,378 cells), reporting wall-clock runtime (minutes) and peak GPU memory usage.

| Method | GROTIA | UniPort | scConfluence | Unioncom | SCOT | MMD-MA |
|---|---|---|---|---|---|---|
| **Runtime (min)** | 15 | 17 | 20.44 | 65 | 185 | 470 |
| **Peak GPU Memory Allocated (GB)** | 9.8 | 8.8 | 3.8 | 4.3 | 25.9 | 11.5 |
| **Peak CPU Resident Memory (GB)** | 5.2 | 0.4 | 1.5 | 13.2 | 0.2 | 1.0 |

**Table A2.** Alignment performance by FOSCTTM under unsupervised setting (First 4 columns: Simulation 1, Simulation 2, Simulation 3, Synthetic RNA-seq).

| | Simulation 1 | Simulation 2 | Simulation 3 | Synthetic RNA-seq |
|---|---|---|---|---|
| SCOT | 0.088 | 0.025 | **0.009** | 0.001 |
| MMD-MA | 0.125 | 0.012 | 0.739 | 0.384 |
| UnionCom | 0.091 | 0.028 | 0.684 | 0.028 |
| Uniport | 0.632 | 0.313 | 0.426 | 0.495 |
| Scconfluence | 0.512 | 0.501 | 0.437 | 0.431 |
| GROTIA(Ours) | **0.077** | **0.008** | **0.009** | **5e-5** |

**Table A3.** Alignment performance by FOSCTTM under unsupervised setting (Last 2 columns: scGEM and SNAREseq).

| | scGEM | SNAREseq | pbmc10X | pmbc | cell line |
|---|---|---|---|---|---|
| SCOT | **0.209** | 0.218 | 0.072 | 0.142 | 0.439 |
| MMD-MA | 0.437 | 0.473 | 0.273 | 0.376 | 0.392 |
| UnionCom | 0.691 | 0.510 | 0.412 | 0.480 | **0.287** |
| Uniport | 0.412 | 0.216 | 0.566 | 0.487 | - |
| Scconfluence(Diagonal) | 0.474 | 0.418 | 0.372 | 0.503 | 0.488 |
| Scconfluence(Prior) | - | - | 0.067 | 0.110 | 0.387 |
| GROTIA(Ours) | 0.215 | **0.216** | **0.049** | **0.104** | 0.318 |

**Table A4.** Alignment performance by label transfer accuracy ($k = 5$) under unsupervised setting (First 4 columns: Simulation 1, Simulation 2, Simulation 3, Synthetic RNA-seq).

| | Simulation 1 | Simulation 2 | Simulation 3 | Synthetic RNA-seq |
|---|---|---|---|---|
| SCOT | **0.977** | 0.977 | **0.950** | **0.996** |
| MMD-MA | 0.897 | 0.957 | 0.700 | 0.506 |
| UnionCom | 0.947 | 0.947 | 0.133 | 0.948 |
| Uniport | 0.36 | 0.577 | 0.35 | 0.433 |
| Scconfluence | 0.493 | 0.523 | 0.490 | 0.650 |
| GROTIA(Ours) | 0.967 | **0.980** | **0.950** | **0.996** |

**Table A5.** Alignment performance by label transfer accuracy ($k = 5$) under unsupervised setting (Last 2 columns: scGEM and SNAREseq).

| | scGEM | SNAREseq | pbmc10X | pmbc | cell line |
|---|---|---|---|---|---|
| SCOT | 0.423 | 0.852 | 0.894 | 0.722 | 0.680 |
| MMD-MA | 0.237 | 0.412 | 0.224 | 0.161 | 0.882 |
| UnionCom | 0.107 | 0.288 | 0.164 | 0.266 | 0.947 |
| Uniport | 0.260 | 0.705 | 0.123 | 0.271 | - |
| Scconfluence(Diagonal) | 0.305 | 0.590 | 0.132 | 0.155 | 0.744 |
| Scconfluence(Prior) | - | - | 0.891 | 0.735 | 0.748 |
| GROTIA(Ours) | **0.588** | **0.947** | **0.897** | **0.802** | **0.954** |

**Table A6.** Alignment performance by FOSCTTM (The lower the better) under semi-supervised setting for the first four datasets.

| | Simulation 1 | Simulation 2 | Simulation 3 | Synthetic RNA-seq |
|---|---|---|---|---|
| SCOT | 0.070 | 0.022 | **0.009** | 0.001 |
| MMD-MA | 0.124 | 0.023 | 0.012 | 0.112 |
| UnionCom | 0.083 | 0.016 | 0.152 | 0.038 |
| Uniport | 0.520 | 0.313 | 0.426 | 0.485 |
| Scconfluence | 0.077 | 0.007 | 0.407 | 0.228 |
| GROTIA(Ours) | **0.069** | **0.005** | **0.009** | **1e-6** |

**Table A7.** Alignment performance by FOSCTTM (The lower the better) under semi-supervised setting for scGEM, SNAREseq, pbmc10X, and pbmc.

| | scGEM | SNAREseq | pbmc10X | pbmc | cell line |
|---|---|---|---|---|---|
| SCOT | **0.192** | 0.150 | 0.073 | 0.142 | 0.439 |
| MMD-MA | 0.201 | 0.150 | 0.277 | 0.374 | 0.378 |
| UnionCom | 0.209 | 0.265 | **0.017** | 0.258 | 0.280 |
| Uniport | 0.259 | 0.220 | 0.566 | 0.487 | - |
| Scconfluence (Diagnola) | 0.234 | 0.154 | 0.080 | 0.211 | 0.367 |
| Scconfluence(Prior) | – | – | 0.067 | **0.101** | **0.272** |
| GROTIA(Ours) | 0.213 | **0.148** | 0.045 | 0.113 | 0.323 |

**Table A8.** Alignment performance by label transfer accuracy ($k = 5$) (The higher the better) under semi-supervised setting for the first four datasets.

| | Simulation 1 | Simulation 2 | Simulation 3 | Synthetic RNA-seq |
|---|---|---|---|---|
| SCOT | 0.937 | 0.977 | **0.957** | **0.998** |
| MMD-MA | 0.890 | 0.783 | 0.947 | 0.706 |
| UnionCom | 0.960 | 0.620 | 0.613 | 0.997 |
| Uniport | 0.360 | 0.577 | 0.350 | 0.442 |
| Scconfluence | 0.960 | 0.990 | 0.580 | 0.997 |
| GROTIA(Ours) | **0.963** | **0.993** | 0.950 | **0.998** |

**Table A9.** Alignment performance by label transfer accuracy ($k = 5$) (The higher the better) under semi-supervised setting for scGEM, SNAREseq, pbmc10X, and pbmc.

| | scGEM | SNAREseq | pbmc10X | pbmc | cell line |
|---|---|---|---|---|---|
| SCOT | 0.576 | 0.982 | 0.894 | 0.722 | 0.679 |
| MMD-MA | 0.588 | 0.942 | 0.357 | 0.258 | **0.985** |
| UnionCom | 0.582 | 0.423 | **0.926** | 0.343 | 0.980 |
| Uniport | 0.412 | 0.719 | 0.123 | 0.276 | - |
| Scconfluence(Diagonal) | 0.621 | 0.982 | 0.786 | 0.574 | 0.836 |
| Scconfluence(Prior) | – | – | 0.891 | 0.747 | **0.985** |
| GROTIA(Ours) | **0.700** | **0.986** | 0.922 | **0.805** | **0.985** |

## Additional Gene Ontology enrichment results

**Table A10.** Gene Ontology enrichment analysis for Dimension 2

| source | GO name | GO ID | p_value | term size | inter. size |
|---|---|---|---|---|---|
| GO:MF | calcium-dependent protein binding | GO:0048306 | 4.566e − 4 | 80 | 8 |
| GO:MF | GTPase regulator activity | GO:0030695 | 2.350e − 3 | 495 | 17 |
| GO:BP | toll-like receptor signaling pathway | GO:0002224 | 8.450e − 3 | 75 | 7 |
| GO:BP | [l]positive regulation of NF-kappaB | | | | |
| transcription factor activity | GO:0051092 | 2.748e − 2 | 124 | 8 | |
| GO:BP | receptor internalization | GO:0031623 | 3.464e − 2 | 128 | 8 |
| GO:CC | membrane raft | GO:0045121 | 4.975e − 4 | 291 | 13 |
| GO:CC | trans-Golgi network membrane | GO:0032588 | 1.304e − 3 | 107 | 8 |
| GO:CC | collagen-containing extracellular matrix | GO:0062023 | 2.733e − 2 | 425 | 13 |
| GO:CC | actin filament | GO:0005884 | 2.747e − 2 | 121 | 7 |
| GO:CC | cell leading edge | GO:0031252 | 3.002e − 2 | 429 | 13 |

**Table A11.** Gene Ontology enrichment analysis for Dimension 4

| source | GO name | GO ID | p_value | term size | inter. size |
|---|---|---|---|---|---|
| GO:MF | immune receptor activity | GO:0140375 | 6.904e − 6 | 145 | 12 |
| GO:BP | [l]positive regulation of protein- | | | | |
| containing complex assembly | GO:0031334 | 3.732e − 3 | 199 | 11 | |
| GO:BP | cell killing | GO:0001906 | 9.412e − 3 | 219 | 11 |
| GO:BP | ruffle organization | GO:0031529 | 2.118e − 2 | 56 | 6 |
| GO:CC | [l]COPII-coated ER to Golgi | | | | |
| transport vesicle | GO:0030134 | 2.685e − 5 | 89 | 9 | |
| GO:CC | ruffle | GO:0001726 | 1.041e − 2 | 182 | 9 |
| GO:CC | focal adhesion | GO:0005925 | 2.953e − 2 | 423 | 13 |

**Table A12.** Gene Ontology enrichment analysis for Dimension 5

| source | GO name | GO ID | p_value | term size | inter. size |
|---|---|---|---|---|---|
| GO:MF | GTPase regulator activity | GO:0030695 | 2.006e − 5 | 495 | 20 |
| GO:MF | phospholipid binding | GO:0005543 | 1.540e − 3 | 484 | 17 |
| GO:BP | [l]negative regulation of | | | | |
| protein phosphorylation | GO:0001933 | 1.810e − 4 | 270 | 14 | |
| GO:BP | [l]positive regulation of NF-kappaB | | | | |
| transcription factor activity | GO:0051092 | 2.258e − 2 | 124 | 8 | |
| GO:BP | regulation of GTPase activity | GO:0043087 | 2.717e − 2 | 210 | 10 |

**Table A13.** Gene Ontology enrichment analysis for Dimension 6

| source | GO name | GO ID | p_value | term size | inter. size |
|---|---|---|---|---|---|
| GO:MF | [l]DNA-binding transcription | | | | |
| factor binding | GO:0140297 | $3.481e-2$ | 490 | 15 | |
| GO:MF | immune receptor activity | GO:0140375 | $3.850e-2$ | 145 | 8 |
| GO:BP | phagocytosis | GO:0006909 | $6.492e-6$ | 234 | 15 |
| GO:BP | cellular response to metal ion | GO:0071248 | $5.224e-4$ | 200 | 12 |
| GO:BP | icosanoid biosynthetic process | GO:0046456 | $1.485e-3$ | 57 | 7 |
| GO:BP | cell killing | GO:0001906 | $9.412e-3$ | 219 | 11 |
| GO:BP | [l]positive regulation of | | | | |
| interleukin-1 beta production | GO:0032731 | $3.171e-2$ | 60 | 6 | |

**Table A14.** Gene Ontology enrichment analysis for Dimension 7

| source | GO name | GO ID | p_value | term size | inter. size |
|---|---|---|---|---|---|
| GO:MF | immune receptor activity | GO:0140375 | $3.729e-5$ | 145 | 11 |
| GO:BP | cell-matrix adhesion | GO:0007160 | $4.671e-2$ | 236 | 10 |
| GO:CC | T cell receptor complex | GO:0042101 | $4.214e-3$ | 136 | 8 |

**Table A15.** Gene Ontology enrichment analysis for Dimension 8

| source | GO name | GO ID | p_value | term size | inter. size |
|---|---|---|---|---|---|
| GO:MF | immune receptor activity | GO:0140375 | $6.680e-5$ | 145 | 11 |
| GO:BP | cell killing | GO:0001906 | $1.217e-4$ | 219 | 13 |
| GO:BP | phagocytosis | GO:0006909 | $1.248e-2$ | 234 | 11 |
| GO:BP | [l]regulation of metal ion transport | GO:0010959 | $4.048e-2$ | 369 | 13 |

## Additional gene importance results

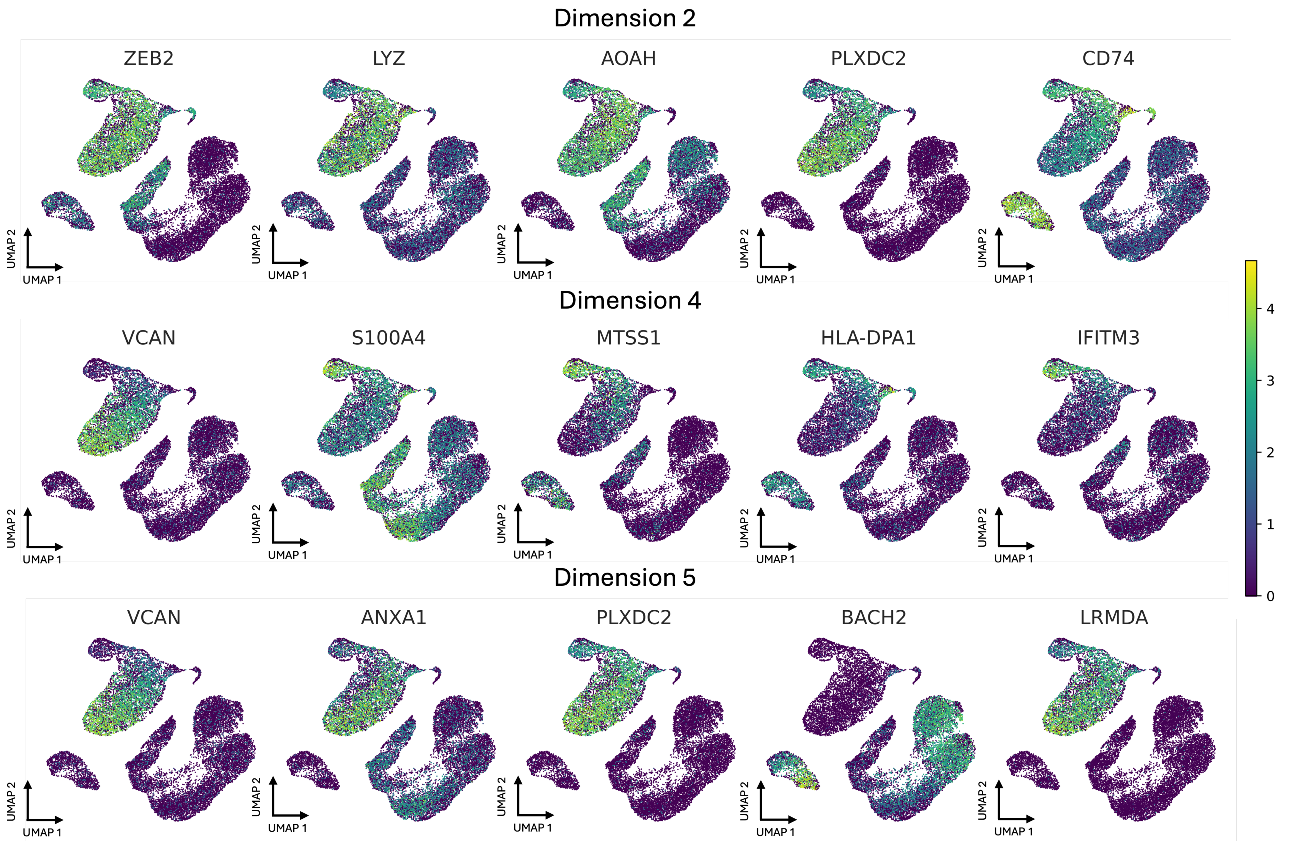


**Figure A.1.** UMAP Plot of Top Genes for Dimensions 2, 4, and 5.

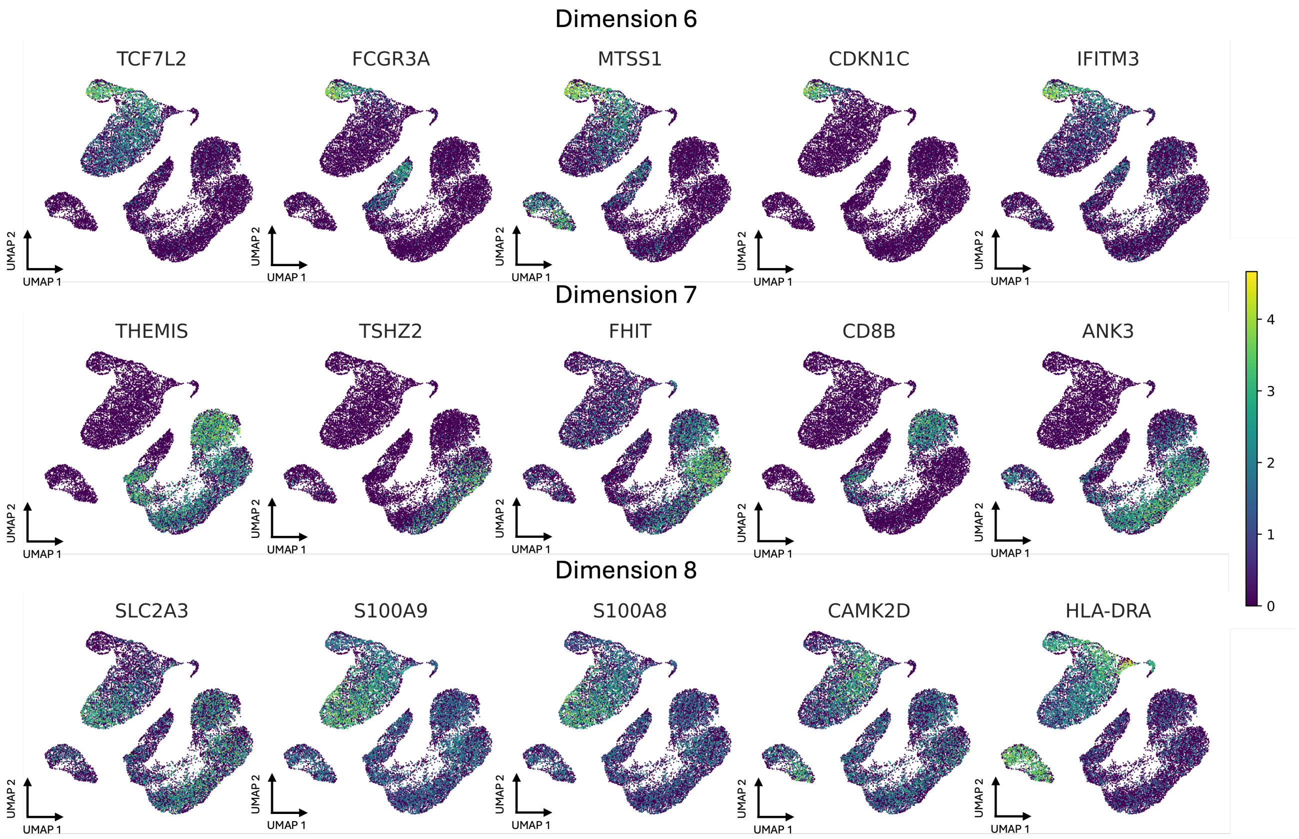


**Figure A.2.** UMAP Plot of Top Genes for Dimensions 6, 7, and 8.

## Optimization Details

### *GROTIA Optimization*

**Algorithm 1** GROTIA optimization

**Require:** Datasets $X, Y$ or precomputed kernels $K_X, K_Y$; graph Laplacians $L_X, L_Y$; latent dimension $p$; weights $\lambda_{\text{topo}}, \lambda_{\text{reg}}$; GeomLoss parameters (blur, reach, scaling); learning rate $\eta$; iterations $T$.

Initialize coefficients $\alpha \in R^{n_x \times p}$, $\beta \in R^{n_y \times p}$ with kernel PCA.
**for** $t = 1$ to $T$ **do**
 $\widetilde{X} \leftarrow K_X \alpha, \quad \widetilde{Y} \leftarrow K_Y \beta$
 $L_{\text{OT}} \leftarrow \text{Sinkhorn}(\widetilde{X}, \widetilde{Y})$ {GeomLoss `SamplesLoss`}
 $L_{\text{ortho}} \leftarrow \|\alpha^\top K_X \alpha - I_p\|_F^2 + \|\beta^\top K_Y \beta - I_p\|_F^2$
 $L_{\text{graph}} \leftarrow \text{tr}(\widetilde{X}^\top L_X \widetilde{X}) + \text{tr}(\widetilde{Y}^\top L_Y \widetilde{Y})$
 $L \leftarrow L_{\text{OT}} + \lambda_{\text{topo}} L_{\text{ortho}} + \lambda_{\text{reg}} L_{\text{graph}}$
 $\alpha \leftarrow \alpha - \eta \frac{\partial L}{\partial \alpha}, \quad \beta \leftarrow \beta - \eta \frac{\partial L}{\partial \beta}$
**end for**
**return** $\widetilde{X} = K_X \alpha, \ \widetilde{Y} = K_Y \beta$

In all experiments we use the implementation summarized in Algorithm 1. For each modality we first compute a Gaussian kernel with bandwidth parameter $\gamma = \frac{1}{2\,\text{med}^2}$ where med is the median of all pairwise Euclidean distances between cells; the resulting RBF kernels are then centered before optimization. We construct $k$-nearest-neighbor graphs ($k = 5$) on the feature representations using correlation distance and build unnormalized Laplacians $L_X, L_Y$ from the symmetrized adjacency matrices. The Sinkhorn optimal transport term $L_{\text{OT}}$ is implemented via the `GeomLoss SamplesLoss` with `loss="sinkhorn"`, $p = 2$, and fixed internal parameters `blur` = 0.01 and `scaling` = 0.8. We optimize the total loss implemented implemented with Adam, double-precision arithmetic, a ReduceLROnPlateau scheduler, and early stopping if the loss does not improve for 10 checks or the learning rate falls below $10^{-6}$. The learning rate is set to $\eta = 10^{-3}$ for datasets with fewer than 5,000 cells and $\eta = 10^{-5}$ for larger datasets.

We treat a small set of hyperparameters as tunable and fix all others to the defaults above. Specifically, for each dataset we perform a grid search over

$$p \in \{5, 8\}, \quad \lambda_{\text{topo}} \in \{1, 10^{-1}, 10^{-2}, 10^{-3}\}, \quad \lambda_{\text{reg}} \in \{10^{-3}, 10^{-4}, 10^{-5}, 10^{-6}, 10^{-7}, 10^{-8}\}, \quad \text{reach} \in \{0.1, 1.0, 5.0\},$$

where $p$ is the latent dimension, $\lambda_{\text{topo}}$ controls the RKHS orthogonality penalty, $\lambda_{\text{reg}}$ controls the graph Laplacian smoothness, and `reach` is the `GeomLoss` parameter governing the effective interaction scale of the Sinkhorn loss. All other parameters (e.g. `blur`, `scaling`, learning rate, number of iterations, graph construction settings) are kept fixed at their default values. The selected configuration for each dataset and the corresponding performance metrics from this grid are reported in the appendix.

### *Effect of the RKHS Orthogonality Constraint*

In GROTIA, the orthogonality penalty

$$\lambda_{\text{ortho}} \left( \|P_X^\top K_X P_X - I_k\|_F^2 + \|P_Y^\top K_Y P_Y - I_k\|_F^2 \right)$$

is introduced to regularize the latent functions in the Reproducing Kernel Hilbert Spaces (RKHSs) of the two modalities. Here $K_X \in R^{n_x \times n_x}$ and $K_Y \in R^{n_y \times n_y}$ denote the kernel Gram matrices, and the columns of $P_X$ and $P_Y$ define RKHS functions

$$f_j^X(\cdot) = \sum_{i=1}^{n_x} (P_X)_{ij} K_X(x_i, \cdot), \qquad f_j^Y(\cdot) = \sum_{i=1}^{n_y} (P_Y)_{ij} K_Y(y_i, \cdot).$$

By standard kernel theory,

$$\langle f_a^X, f_b^X \rangle_{\mathcal{H}_X} = (P_X)_a^\top K_X (P_X)_b,$$

so the matrices

$$G_X := P_X^\top K_X P_X, \qquad G_Y := P_Y^\top K_Y P_Y$$

are precisely the RKHS Gram matrices of the learned latent functions: $(G_X)_{ab} = \langle f_a^X, f_b^X \rangle_{\mathcal{H}_X}$ and similarly for $Y$. The penalty therefore drives $G_X$ and $G_Y$ towards the identity, i.e. it encourages the latent functions to form approximately orthonormal systems in their respective RKHSs.

If $K_X$ is positive definite and $G_X = I_k$, then $\langle f_a^X, f_b^X \rangle_{\mathcal{H}_X} = \delta_{ab}$ and $\{f_j^X\}_{j=1}^k$ is an orthonormal system in $\mathcal{H}_X$ with $\text{rank}(G_X) = k$. Moreover,

$$\|G_X - I_k\|_F^2 = \sum_{i=1}^{k} (\lambda_i(G_X) - 1)^2$$

in terms of the eigenvalues $\lambda_i(G_X)$, so the orthogonality penalty directly discourages both small eigenvalues (rank degeneracy) and large off–diagonal entries (correlation between latent functions).

To quantify the effect of this term, we compared models trained with and without the orthogonality penalty by setting $\lambda_{\text{ortho}} = 0$ versus $\lambda_{\text{ortho}} = 1$, while keeping all other hyperparameters fixed. For each setting we ran the optimization from multiple random seeds and evaluated, for both $G_X$ and $G_Y$: (i) the eigenvalue spectrum and an effective rank, and (ii) the off–diagonal structure of $G$ as a measure of redundancy between axes. In addition, it helps avoid collapsed embeddings induced by the graph Laplacian terms.

### *Prevention of rank degeneracy in RKHS*

If the orthogonality penalty were minimized exactly, we would have $G_X = I_k$ and $G_Y = I_k$, so the latent functions would form orthonormal bases of $k$-dimensional subspaces in $\mathcal{H}_X$ and $\mathcal{H}_Y$, respectively. In particular, all eigenvalues of $G_X$ and $G_Y$ would be equal to 1 and the representations would be full-rank in RKHS.

Empirically, when the orthogonality penalty was removed ($\lambda_{\text{ortho}} = 0$), the eigenvalue spectra of $G_X$ and $G_Y$ were highly anisotropic: almost all spectral mass was concentrated in a single dominant eigenvalue, while the remaining eigenvalues were several orders of magnitude smaller. In our experiments with $k = 8$, we obtained for modality $X$

$$\lambda(G_X) \approx \left(1.7 \times 10^{-4},\ 5.5 \times 10^{-4},\ 1.0 \times 10^{-3},\ 2.0 \times 10^{-3},\ 2.6 \times 10^{-3},\ 3.1 \times 10^{-3},\ 4.2 \times 10^{-2},\ 8.8 \times 10^{-1}\right),$$

and for modality $Y$

$$\lambda(G_Y) \approx \left(2.0 \times 10^{-4},\ 7.0 \times 10^{-4},\ 1.0 \times 10^{-3},\ 1.7 \times 10^{-3},\ 3.3 \times 10^{-3},\ 4.0 \times 10^{-3},\ 3.7 \times 10^{-2},\ 7.6 \times 10^{-1}\right).$$

Thus, although we nominally learn $k = 8$ latent functions, almost all RKHS energy lies in a single direction and several eigenvalues are two–three orders of magnitude smaller than the largest; the effective rank

$$\text{eff-rank}(G) := \#\{\lambda_i(G) > 10^{-3}\}$$

is only 6 for both modalities, indicating a near rank-deficient representation in RKHS.

In contrast, when the orthogonality term was active ($\lambda_{\text{ortho}} = 1$), all eigenvalues of $G_X$ and $G_Y$ were numerically equal to 1 up to floating-point precision (approximately 0.9999998–0.99999999), and the effective rank matched the target $k = 8$ in all runs. This confirms that the orthogonality penalty empirically enforces a non-degenerate, $k$-dimensional RKHS representation and prevents the model from collapsing most of the RKHS energy into a single direction.

**Table A16.** RKHS Gram diagnostics with and without the orthogonality penalty (mean over 5 seeds). Here $\lambda_{\text{ortho}} = 0$ corresponds to no orthogonality regularization and $\lambda_{\text{ortho}} = 1$ to the default setting.

| $\lambda_{\text{ortho}}$ | Modality | eff_rank | $\|G_{\text{off}}\|_F$ | mean$\|G_{\text{off}}\|$ | max$\|G_{\text{off}}\|$ |
|---|---|---|---|---|---|
| 0 | $X$ | 6.0 | $7.55 \times 10^{-1}$ | $6.66 \times 10^{-2}$ | $2.50 \times 10^{-1}$ |
| 0 | $Y$ | 6.0 | $6.51 \times 10^{-1}$ | $5.73 \times 10^{-2}$ | $2.11 \times 10^{-1}$ |
| 1 | $X$ | 8.0 | $2.67 \times 10^{-15}$ | $2.35 \times 10^{-16}$ | $9.12 \times 10^{-16}$ |
| 1 | $Y$ | 8.0 | $2.98 \times 10^{-15}$ | $2.76 \times 10^{-16}$ | $9.92 \times 10^{-16}$ |

***Reduction of redundancy between latent axes***

The off–diagonal entries of $G_X$ and $G_Y$ encode the RKHS inner products between distinct latent functions. Large off–diagonal values indicate that different latent axes are strongly correlated or nearly collinear in RKHS, and therefore redundant. To quantify this, we considered the Frobenius norm of the off–diagonal part,

$$\|G_{\text{off}}\|_F := \left\| G - \operatorname{diag}(\operatorname{diag}(G)) \right\|_F = \sqrt{\sum_{a \neq b} G_{ab}^2},$$

as well as the mean and maximum absolute off–diagonal entries.

Without the orthogonality penalty ($\lambda_{\text{ortho}} = 0$), we observed substantial off–diagonal mass in both $G_X$ and $G_Y$. For modality $X$, the mean absolute off–diagonal entry was approximately $6.7 \times 10^{-2}$, with a maximum of $2.5 \times 10^{-1}$ and $\|G_{\text{off}}\|_F \approx 7.6 \times 10^{-1}$. For modality $Y$, the corresponding values were approximately $5.7 \times 10^{-2}$, $2.1 \times 10^{-1}$, and $6.5 \times 10^{-1}$. These numbers confirm that, in the absence of the orthogonality term, the learned latent functions are strongly correlated in RKHS, and several axes effectively capture overlapping structure.

When $\lambda_{\text{ortho}} = 1$, the off–diagonal entries of $G_X$ and $G_Y$ collapsed to numerical zero: $\|G_{\text{off}}\|_F$ was on the order of $10^{-15}$, and the mean absolute off–diagonal entries were on the order of $10^{-16}$ for both modalities. This is consistent with the intended effect of the penalty: different latent functions become RKHS-orthogonal in practice, and the model no longer allocates capacity to redundant, collinear directions.

For convenience, Table A16 summarizes the rank and redundancy diagnostics averaged over random seeds.

***Avoiding collapsed embeddings***

It also prevents embedding collapse due to graph Laplacian regularization. Without the orthogonality penalty (i.e. with $\lambda_{\text{ortho}} = 0$), the GROTIA objective reduces to

$$\mathcal{L}_{\text{OT}}(\widetilde{X}, \widetilde{Y}) + \lambda \left[ \operatorname{Tr}(\widetilde{X}^\top L_X \widetilde{X}) + \operatorname{Tr}(\widetilde{Y}^\top L_Y \widetilde{Y}) \right].$$

Both the optimal transport loss $\mathcal{L}_{\text{OT}}$ and the graph Laplacian terms are nonnegative. In particular, if we take

$$\widetilde{X} = 0, \qquad \widetilde{Y} = 0 \quad \Longleftrightarrow \quad P_X = 0, \; P_Y = 0,$$

then every term in the expression above is exactly zero. Thus $(P_X, P_Y) = (0, 0)$, i.e. a fully collapsed embedding where all cells are mapped to the origin, is one of the global minimizers of the graph-regularized OT objective when the orthogonality constraint is absent.

The RKHS orthogonality penalty is specifically designed to rule out such collapsed solutions. It adds

$$\lambda_{\text{ortho}} \left( \|P_X^\top K_X P_X - I_k\|_F^2 + \|P_Y^\top K_Y P_Y - I_k\|_F^2 \right)$$

to the objective. At the collapsed solution $P_X = 0, P_Y = 0$ we have

$$P_X^\top K_X P_X = 0, \qquad P_Y^\top K_Y P_Y = 0,$$

so the penalty becomes

$$\lambda_{\text{ortho}} \left( \|0 - I_k\|_F^2 + \|0 - I_k\|_F^2 \right) = 2\lambda_{\text{ortho}} \|I_k\|_F^2,$$

which is large and strictly positive. In contrast, for non-collapsed embeddings where the latent RKHS functions are close to orthonormal (so that $P_X^\top K_X P_X \approx I_k$ and $P_Y^\top K_Y P_Y \approx I_k$), this penalty is close to zero.

Consequently, once the orthogonality term is active ($\lambda_{\text{ortho}} > 0$), the trivial collapsed solution $\widetilde{X} = \widetilde{Y} = 0$ is no longer optimal: any embedding that satisfies the orthogonality constraint reasonably well will achieve a strictly lower total objective value. In this simple sense, the RKHS orthogonality constraint stabilizes the model against collapse and enforces a non-degenerate latent embedding.

### *Hyperparameter Robustness Analysis*

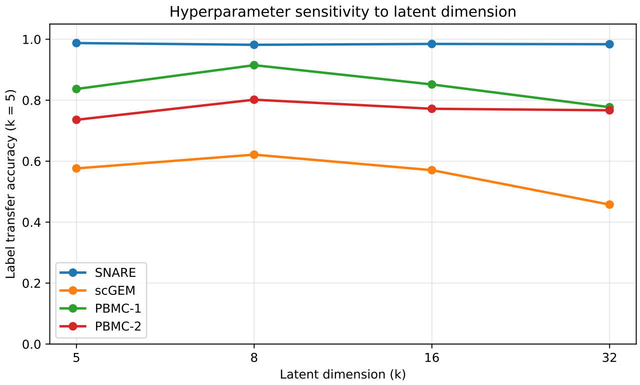


**Figure A.3.** Hyperparameter robustness with respect to latent dimension. Label transfer accuracy (k = 5) is shown for SNARE, scGEM, PBMC-1, and PBMC-2 across latent embedding dimensions $k \in \{5, 8, 16, 32\}$.

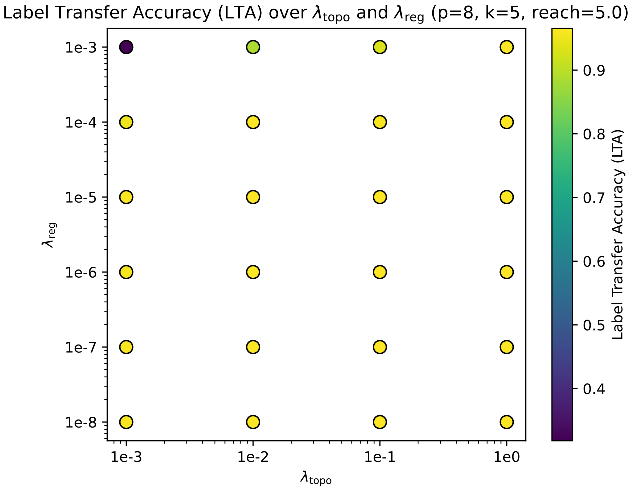


**Figure A.4.** Hyperparameter grid over regularization strengths. Label transfer accuracy (k = 5) is shown as a function of the orthogonality weight $\lambda_{topo}$ and the graph Laplacian weight $\lambda_{reg}$ (both on logarithmic scales) for a fixed latent dimension and reach. GROTIA attains high accuracy across a broad plateau in $(\lambda_{topo}, \lambda_{reg})$, indicating robustness of performance to these regularization strengths.

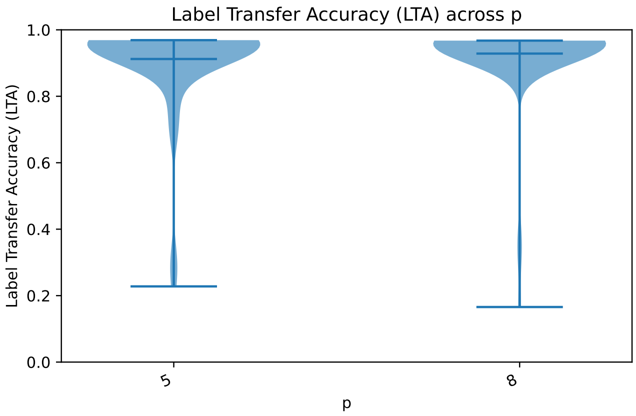


**Figure A.5.** Robustness with respect to latent dimension. For each latent dimension $p \in \{5, 8\}$, the violin shows the distribution of label transfer accuracy (k = 5) across all combinations of $(\lambda_{topo}, \lambda_{reg}, \text{reach})$ in the grid search. Accuracy remains high and comparable for both values of $p$, indicating that GROTIA does not require fine tuning of the latent dimensionality.

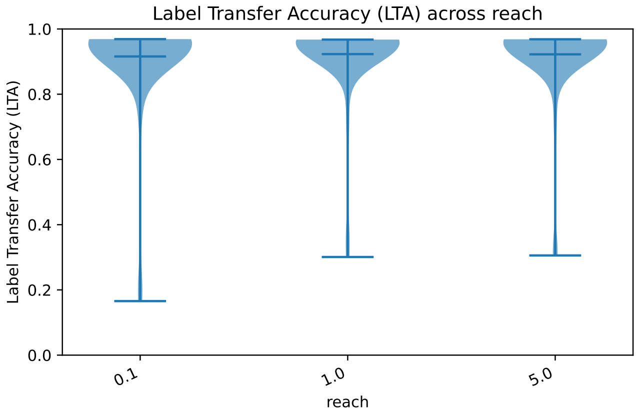


**Figure A.6.** Robustness with respect to the Sinkhorn interaction scale. For each value of the `reach` parameter $\in \{0.1, 1.0, 5.0\}$, the violin shows the distribution of label transfer accuracy (k = 5) across all combinations of $(p, \lambda_{topo}, \lambda_{reg})$ in the grid search. The similar, high-accuracy distributions indicate that GROTIA is insensitive to the precise choice of `reach`.

In all experiments, we tuned GROTIA's hyperparameters by monitoring Label Transfer Accuracy (LTA, $k = 5$). First, we examined the effect of the latent dimension $p$ on each dataset (SNARE, scGEM, PBMC-1, PBMC-2; Fig. A.3). As shown in Fig. A.3, latent dimensions $p = 5$ and $p = 8$ consistently achieve the best or near-best label transfer accuracy across all evaluated datasets, whereas performance gradually degrades at higher dimensions ($p = 16$ and $p = 32$). We therefore restrict $p$ to 5 or 8 in all reported experiments with GROTIA. Next, we examine the effect of hyperparameters on the SNARE dataset. To assess sensitivity to the regularization strengths, we then performed a grid search over $\lambda_{topo}$ and $\lambda_{reg}$ for a representative setting (Fig. A.4). The resulting 2D map shows a wide plateau of configurations with LTA > 0.9, confirming that performance is stable across several orders of magnitude in both penalties, except for a small corner with very strong graph regularization and very weak orthogonality. Finally, the violin plots (Figs. A.5 and A.6) summarize robustness by aggregating LTA across all combinations of the remaining hyperparameters: for both choices of $p$ and for all values of the Sinkhorn reach parameter (reach $\in \{0.1, 1.0, 5.0\}$), the distributions are tightly concentrated near high LTA values, with only rare low-performing outliers. Together, these diagnostics indicate that GROTIA is robust to hyperparameter choices and that our chosen defaults lie in a broad region of stable performance.

Click here to access/download
**Supplementary Material**
supplementary.pdf

Click here to access/download
**Supplementary Material**
GigaScience_Response.docx

DEPARTMENT of BIOSTATISTICS EPIDEMIOLOGY & INFORMATICS
Perelman School of Medicine UNIVERSITY of PENNSYLVANIA

December 24, 2025

Dear Respected Editors,

This is a revised manuscript GIGA-D-25-00229R1 "An Interpretable Graph-Regularized Optimal Transport Framework for Diagonal Single-Cell Integrative Analysis" for consideration in **GigaScience**.

We thank our reviewers for their comments and suggestions and have adopted them into the revision. The responses are included in this submission. We would like to thank you and the reviewers for the constructive review of the manuscript. We feel that we have been able to address the issues raised by the reviewers within the scope of the present investigation and data available to us at this time. We greatly appreciate the thoughtful questions and comments and believe that the reviewer's suggestions have significantly improved the manuscript.

We thank you for considering this work for the **GigaScience** journal and hope for a favorable response.

Sincerely,

Li Shen, Ph.D., FAIMBE, FACMI
Professor of Informatics, Radiology, and CIS
Perelman School of Medicine, University of Pennsylvania

B306 Richards Building, 3700 Hamilton Walk • Philadelphia, PA 19104-6021 •
Li.Shen@pennmedicine.upenn.edu

Graphical Abstract

Click here to access/download;Graphical Abstract;Graphical Abstract.pdf

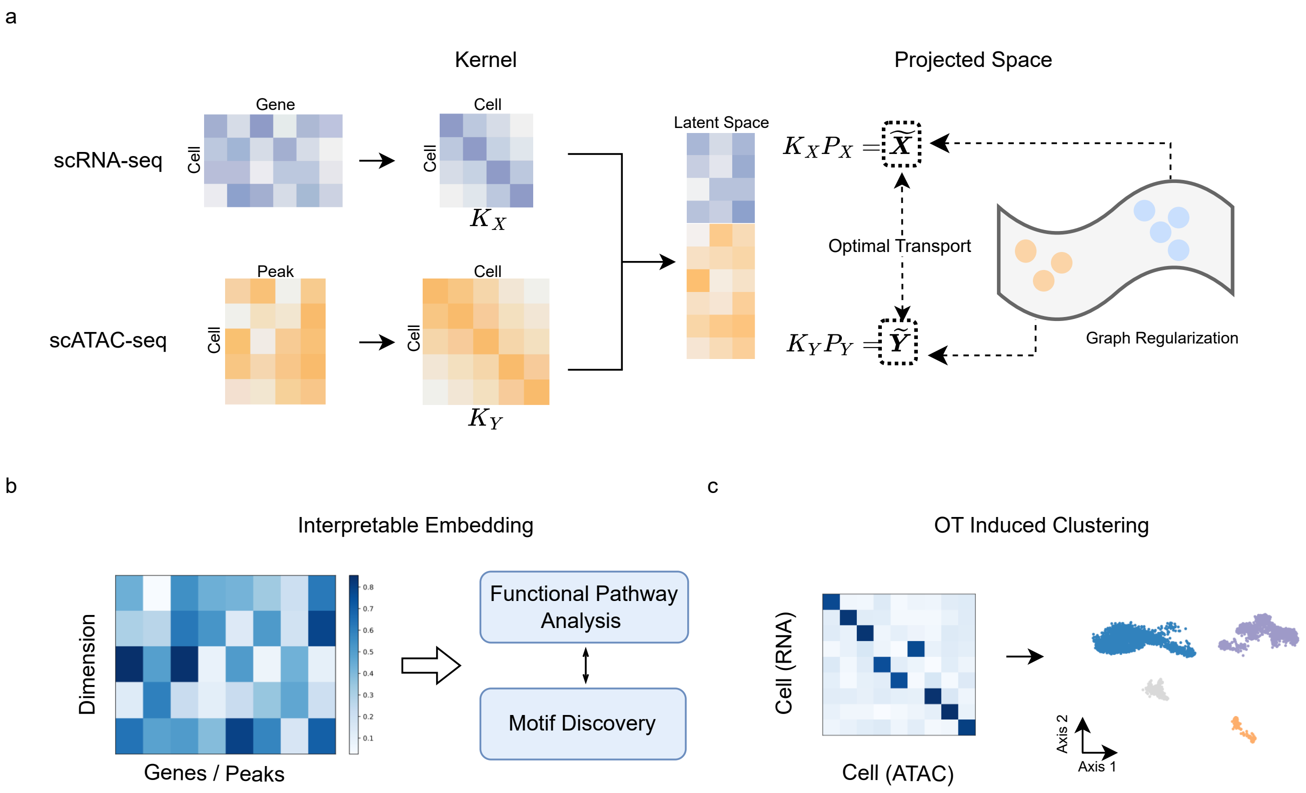

Supplement: giag012_GIGA-D-25-00229_Revision_1 [file giag012_giga-d-25-00229_revision_1.pdf]
