# Supplementary material for: An interpretable Graph-Regularized Optimal Transport Framework for Diagonal Single-Cell Integrative Analysis
Source: Gigascience. 2026 Feb 9;15:giag012. doi: 10.1093/gigascience/giag012 (PMC12970605; doi:10.1093/gigascience/giag012)
Supplement: giag012_Authors_Response_To_Reviewer_Comments_original_submission [file giag012_authors_response_to_reviewer_comments_original_submission.pdf]

Author's Response To Reviewer Comments

We sincerely appreciate the editors and reviewers for their valuable comments, which have significantly improved the quality of our manuscript. All comments have been thoughtfully addressed in the revised manuscript. To aid the reviewing process, we have highlighted the revised parts in blue and provided detailed point-by-point responses below.

****
Reviewers' Comments to Author:
****
Reviewer: 1
****

Comments:

The manuscript presents a well-motivated and technically elegant approach to diagonal single-cell data integration, combining optimal transport with graph-based regularization to achieve a balance between global and local structure alignment. The method addresses an important challenge in single-cell data integration, where existing approaches still leave room for improvement. Its embedding design offers the potential for interpretable feature-level insights, a particularly desirable quality in single-cell multi-omics integration where biological interpretability is especially important.

Response:

Thank you for this positive and encouraging feedback. We appreciate the reviewer's recognition of the motivation, technical design, and interpretability of our approach, and we are glad that the balance between global and local structure alignment, as well as the potential for feature-level biological insights, is viewed as a meaningful contribution to single-cell multi-omics integration.

—---------------------------------------

Comments:

That said, the manuscript would be substantially strengthened by deeper validation and a clearer demonstration of reproducibility. Some claims would benefit from stronger empirical support in the presented results, and a more thorough evaluation of the method's added value relative to unimodal alternatives, particularly in the context of marker gene discovery and the identification of cell types or subpopulations, could further enhance the manuscript. Additionally, the impact of key parameter choices, such as kernel bandwidth selection, the number of nearest neighbors (k), and sensitivity to hyperparameters (λ, ρ), should be more fully explored, reported, or justified. Reproducibility could be improved by providing scripts and a computational environment or container to replicate all analyses and figures presented in the manuscript. Usability would also be improved by providing the method as an installable Python package, rather than limiting implementation to a Jupyter Notebook.

Response:

Thank you for these constructive suggestions. We agree that stronger validation, clearer reproducibility, and improved usability would further strengthen the manuscript. Many of the points raised here (e.g., additional empirical support, comparison to unimodal alternatives for marker discovery and subpopulation identification, sensitivity to key parameters such as kernel bandwidth, k, and hyperparameters including λ and ρ, and improvements to code organization, environment specification, and packaging) overlap with the reviewer's detailed comments. We therefore address each item step by step in our responses below, and have revised the manuscript and repository accordingly to improve clarity, transparency, and reproducibility.

—---------------------------------------

Comments:

Overall, the manuscript introduces a compelling methodological framework with meaningful potential for applications in single-cell integration. The suggestions that follow are intended to help the authors strengthen their contribution in alignment with GigaScience's emphasis on openness, reproducibility, and FAIR principles. I hope these suggestions will help strengthen the support for the authors' conclusions, clarify the reasoning behind key arguments, and improve the clarity and interpretability of the figures and descriptions.

Response:

Thank you for this constructive feedback. We appreciate the reviewer’s emphasis on openness, reproducibility, and clarity, and have revised the manuscript and supporting materials accordingly to strengthen the presentation and better support our conclusions.

—---------------------------------------

Detailed Comments:

1. Reproducibility

Reproducibility is impeded by the absence of clearly organized scripts or workflow files to regenerate the results, figures, and tables presented in the manuscript. While some outputs are shown or alluded to in the Jupyter Notebook found in the linked GitHub repository, they are not clearly cross-referenced with the paper's results, making it difficult to confirm how specific figures or tables were produced. Furthermore, no computational environment specification is provided, which makes replication with confidence impossible. Certain aspects of the manuscript fall short of best practices for transparent and reproducible research. Analysis scripts are incomplete or undocumented, and key portions of the software pipeline are either insufficiently described or lack proper attribution. These limitations hinder reproducibility and reduce reusability. Figures would also benefit from clearer annotation. Collectively, these shortcomings detract from alignment with the FAIR principles emphasized by GigaScience. Reproducibility would be significantly improved by packaging the software, versioning the code, defining and documenting the computational environment, and depositing all components of the analysis pipeline, including preprocessing scripts, evaluation code, and figure generation, in a publicly accessible repository.

Response:

We thank the reviewer for their comments regarding reproducibility and would like to clarify one important point. Although the code is distributed in Jupyter Notebook format, all experiments were designed and executed in Google Colab, rather than in a locally configured Jupyter environment. Google Colab provides a standardized and stable execution environment, which substantially reduces system-dependent variability and supports reproducibility.

In response to the reviewer’s comments, we have taken several concrete steps to strengthen reproducibility and reusability; all corresponding code updates are available at https://github.com/PennShenLab/GROTIA :
- We now provide an explicit environment.txt file specifying all required packages and versions.
- We include fully documented and self-contained notebooks implementing our integration method, covering interpretable embeddings for both RNA and ATAC modalities as well as OT-based co-clustering.
- We include scripts for running all baseline methods used in the manuscript to facilitate fair comparison and independent verification.

We have added detailed explanations for each function within the notebooks, complemented by in-code documentation comments.

—--------------------------------------

Comments:

Additionally, while it is generally clear how the data were collected and curated, the rationale for using preprocessed datasets, particularly those sourced from external repositories, could be more clearly explained. The data are shared via a Google Drive link provided in the GitHub repository, which is convenient, though it may benefit from a more transparent and persistent form of distribution. The manuscript states that "All data used in this manuscript is publicly available and can be found at Liu et al. [11], Cheow et al. [16], Demetci et al. [12], Chen et al. [17], Cao et al. [14], and Samaran et al. [13].", but it appears that preprocessed versions of these datasets were used, rather than the original raw data. Clarifying this point would help improve transparency and reproducibility.

Response:

We clarify that all datasets were used exactly as released by the original benchmarked integration methods to ensure fair and consistent comparison, and no customized or alternative preprocessing pipelines were introduced in this work.

Specifically, datasets from Liu et al. [11], Cheow et al. [16], Demetci et al. [12], and Chen et al. [17] are only publicly available in preprocessed form as provided by the original authors, and we use these versions without modification. In contrast, datasets from Cao et al. [14] and Samaran et al. [13] are released as raw data, and we preprocess them strictly following the procedures described by the original authors, using the same steps and parameters as documented in their tutorials.

This is now explicitly stated in the Training Details section:

“The input to GROTIA follows standard dataset preprocessing practices and does not require method-specific processing. For all simulated datasets, features were z-score normalized prior to alignment, following the procedure used in \citet{liu2019jointly}. For the scGEM and SNARE-seq datasets, we downloaded the preprocessed datasets provided by \citet{demetci2022scot} and applied the same unit-normalization procedure described in that work. For the PBMC-1 and PBMC-2 datasets, we strictly followed the preprocessing pipelines used by the original authors of the corresponding benchmarked methods \citep{samaran2024scconfluence,cao2022unified}. Specifically, we downloaded the raw data and applied the preprocessing scripts provided by the respective authors. Consequently, all datasets were used exactly as released by the original benchmarked integration methods to ensure fair and consistent comparison, and no customized or alternative preprocessing pipelines were introduced in this work.”

—--------------------------------------

Comments:

The manuscript also describes custom preprocessing procedures for scRNA-seq and scATAC-seq data, including PCA, TF-IDF normalization, and gene filtering, that appear inconsistent with the properties of the datasets used. Without access to preprocessing scripts or further clarification, it is unclear whether these procedures were performed as described. Clarifying these discrepancies would strengthen transparency and ensure fair benchmarking comparisons. In addition, to improve transparency and reproducibility, it would be helpful to provide the scripts or commands used to run these baseline methods, along with the evaluation code for computing the reported metrics.

Response:

We clarify that the preprocessing steps described in the manuscript for scRNA-seq and scATAC-seq data—including PCA, TF-IDF normalization, and gene filtering—are not custom procedures introduced by our work, but instead strictly follow the preprocessing pipelines used by the original authors of the corresponding benchmarked methods

This is now explicitly stated in the Training Details section (same as previous response):

“The input to GROTIA follows standard dataset preprocessing practices and does not require method-specific processing. For all simulated datasets, features were z-score normalized prior to alignment, following the procedure used in \citet{liu2019jointly}. For the scGEM and SNARE-seq datasets, we downloaded the preprocessed datasets provided by \citet{demetci2022scot} and applied the same unit-normalization procedure described in that work. For the PBMC-1 and PBMC-2 datasets, we strictly followed the preprocessing pipelines used by the original authors of the corresponding benchmarked methods \citep{samaran2024scconfluence,cao2022unified}. Specifically, we downloaded the raw data and applied the preprocessing scripts provided by the respective authors. Consequently, all datasets were used exactly as released by the original benchmarked integration methods to ensure fair and consistent comparison, and no customized or alternative preprocessing pipelines were introduced in this work.”

—--------------------------------------

Comments:

Finally, several methodological details underlying downstream analyses are insufficiently described to allow confident reproduction or interpretation. For instance, it is unclear which dataset was used to obtain the results in "GROTIA Reveals Gene-Specific Contributions and Key Biological Processes in the RNA Embedding" and Figure 4 and 5. Additionally, the motif discovery step using GimmeMotifs should be expanded, since it is currently not entirely clear how motifs were matched to known transcription factors, and the process described in the text does not fully align with what is shown in Figure 5A. Clarifying these points would help improve the reproducibility and interpretability of the manuscript's key biological findings.

Response:

Regarding downstream analyses and motif discovery, we thank the reviewer for highlighting the need for additional clarification and reproducibility.

First, we clarify that all analyses presented in “GROTIA Reveals Gene-Specific Contributions and Key Biological Processes in the RNA Embedding”, as well as Figures 4 and 5, are conducted on the PBMC-1 dataset.

In the revised manuscript, we add the following statement
“The PBMC-1 dataset is used to demonstrate the following experiments.”
at the beginning of the three sections respectively titled
- “GROTIA Reveals Gene-Specific Contributions and Key Biological Processes in the RNA Embedding”,
- “GROTIA Identifies High-Impact Peaks and Regulatory Mechanisms in the ATAC Embedding”, and
- “GROTIA Enables Identification of Cellular Subpopulations in the Integrated Space”:

Second, regarding motif discovery, we confirm that the use of GimmeMotifs is fully consistent with the workflow illustrated in Figure 5A. To improve transparency and alignment between the text and the figure, We have revised the Interpretable Embeddings of GROTIA section to explicitly detail each step of the motif discovery and annotation process, as follows:

“We then identified transcription factor binding sites (TFBS) by performing de novo motif discovery on the most influential ATAC-seq peaks associated with each latent dimension using GimmeMotifs \citep{bruse2018gimmemotifs}, applying a false discovery rate (FDR) threshold of < 0.001. This analysis yields enriched DNA sequence motifs representing putative TF binding sites in accessible chromatin and, through motif annotation, provides a set of candidate transcription factors (TFs) associated with each motif. Specifically, each de novo transcription factors binding site (TFBS) was annotated by matching its position weight matrix (PWM) to reference motif databases (including JASPAR, HOCOMOCO, CIS-BP, and ENCODE) using Pearson correlation–based similarity scores, thereby assigning candidate transcription factors (TFs) to each TFBS based on similarity to known binding preferences. We next linked individual TFBS-containing peaks to proximal genes by assigning each site to the nearest gene within a ±20 kb

window \citep{kan2022multiomics, diwadkar2020facilitating}. Finally, these candidate target genes were filtered by intersecting them with the top RNA-expressed genes associated with the same latent dimension, yielding dimension-specific TF–gene regulatory pairs.”

—-------------------------------------

Comments:

2. Usability

The code repository is easy to find on GitHub, available under the MIT license, following the link presented in the manuscript. However, the currently presented implementation is provided as a Jupyter Notebook that demonstrates the basic usage of the method, and technically allows users to replicate the process using their own data. Usability is currently limited by sparse documentation and could benefit from guidance on input requirements, parameter configuration, and expected output formats. To improve usability, the authors should supplement the notebook with detailed explanations, comments, and a README or user guide that explains how to prepare input data, adjust key parameters, interpret outputs, and run the method on other datasets. Wrapping core functionality into a small, importable Python module or script would further reduce friction for adoption and integration into pipelines.

Response:

We thank the reviewer for the constructive suggestions regarding code usability and documentation. Although the implementation is presented in Jupyter Notebook format, the workflow is designed to run on Google Colab, providing a stable and environment-independent execution setting that lowers the barrier for reproduction and modification.

In response to the reviewer’s comments, we have substantially improved the usability and documentation of the codebase. We have expanded the documentation to clearly describe input requirements, parameter configurations, and expected output formats, and each notebook now includes step-by-step guidance for running the method and interpreting the results. In addition, all core functionalities have been refactored into standalone, importable .py modules, with major functions consolidated into a dedicated GROTIA_utils.py file that is directly imported by the notebooks. This modular design enables flexible reuse of individual components and easier integration into custom analysis pipelines. All of the above changes are available at https://github.com/PennShenLab/GROTIA.

—-------------------------------------

Comments:

3. Attribution and Software Transparency

The GitHub repository includes an evals.py script originally authored by the creators of SCOT (Pinar Demetci, Rebecca Santorella, and Ritambhara Singh), with attribution preserved within the file. However, the manuscript itself does not mention that components of the evaluation pipeline were adapted from this prior work. Given that this script supports benchmarking comparisons central to the paper's conclusions, explicit acknowledgment in the text would improve transparency and ensure appropriate credit is given.

Response:

We thank the reviewer for pointing this out. The evals.py script in our repository is adapted from the evaluation code released with SCOT \citep{demetci2022scot}, with attribution preserved in the source file. We have now explicitly acknowledged this in the manuscript under the Evaluated Metrics section as followed:
“The second metric is Label transfer accuracy (LTA) and it evaluates how well cell-type (or other categorical) labels can be transferred from domain X to domain Y in the integrated space \citep{demetci2022scot}.”

—--------------------------------------

Comments:

4a. Support for Claims and Biological Interpretation

Several key claims would benefit from additional evidence or clarification. I divide this into subsections "4a. Methodological Claims," "4b. Biological Interpretation," and "4c. Clustering Evaluation" for extra clarity and readability.

4a. Methodological Claims
- The claim "we selected the latent dimension to be either 5 or 8 and observed that GROTIA remained robust to this choice" is not substantiated by any reported results or sensitivity analysis.

Response:

The choice of latent dimension was guided by prior kernel-based integration methods, particularly MMD-MA, which commonly uses a latent dimension of 5. Following this established practice, we selected latent dimensions of 5 or 8 in our main experiments.

To explicitly assess robustness, we additionally evaluated a broader range of latent dimensions (5, 8, 16, and 32). We now include a Hyperparameter Robustness Analysis section in the Appendix. As shown in Figure A.3, latent dimensions of 5 and 8 consistently achieve the best or near-best label transfer accuracy across all evaluated datasets (SNARE, scGEM, PBMC-1, and PBMC-2). While performance gradually degrades at higher dimensions (16 and 32), no abrupt instability is observed, indicating that GROTIA is robust to reasonable variations in latent dimension.

We have revised the Training Details section to explicitly reference this analysis, stating:
“A more detailed explanation is provided in the Appendix: Optimization Details.”

—--------------------------------------

Comments:

- The claim that GROTIA is computationally efficient would be more compelling if runtime comparisons included system specifications, analysis on larger (potentially synthetic) datasets, memory usage, and scalability assessments across CPU and GPU modes. Directly referencing Table A1 for the current runtime evaluation and adding the additional metrics mentioned above would provide a more comprehensive evaluation.

Response:

We thank the reviewer for this constructive suggestion. To address this point, we have added wall-clock runtime, peak GPU memory allocated, and peak CPU resident memory (RSS) for all benchmarked methods on the PBMC-1 dataset (9,378 cells) in the Appendix: Metric Performance section.

As shown in Table A1, while GROTIA does not achieve lower memory usage than batched methods such as UniPort and scConfluence, it remains substantially more efficient than other full-matrix approaches (e.g., SCOT, UnionCom, and MMD-MA) in both runtime and memory consumption. This highlights that, despite adopting a full-matrix formulation, GROTIA achieves a favorable efficiency–accuracy trade-off at practical single-cell scales. Providing a more detailed GPU scalability analysis is a promising direction for future work.

In addition, we revised the Discussion section as follows:

“Benchmarking against state-of-the-art methods in both unsupervised and semi-supervised settings,

GROTIA delivers comparable or superior performance while offering a computationally efficient solution (see Appendix: Metric Performance).”
We have also added system configuration details to the Training Details section, as follows:
“All experiments were run on a single NVIDIA A100 GPU.”

—--------------------------------------

Comments:

- The manuscript asserts "Notably, unlike methods that require shared features across modalities, GROTIA only assumes that cells (rather than individual genes or peaks) follow a similar distribution if they belong to the same type or lineage—thus broadening its applicability to complex datasets." This claim would be more convincing if supported by analyses on more complex datasets, such as those with technical variability across origin sites, donors, or protocols; mosaic structures with missing observations; nested batch effects; or significant differences in data quality. Additionally, this statement may appear in tension with the claim that GROTIA depends on the presence of a shared underlying biology, which would not hold in many complex or heterogeneous settings. Clarifying how "complexity" is defined in the context of GROTIA's assumptions, and empirically substantiating the method's generalizability to such settings would improve both the precision and credibility of this claim.

Response:

We thank the reviewer for pointing out that our statement about “not requiring shared features” and “complex datasets” needed clearer justification. In GROTIA, we work with two modalities X and Y that can live in completely different feature spaces (for example, RNA expression vs chromatin accessibility). For each modality, we construct a cell–cell similarity matrix (a kernel matrix) from the high-dimensional feature profiles. What GROTIA does assume is shared cell-state structure, not shared features. Specifically, cells of the same type or lineage should form coherent neighborhoods in each modality, and the relative arrangement of these neighborhoods (which types are closer or more distinct) should be comparable across modalities. The kernels and graph regularization preserve this intra- and inter-type structure in both domains, and OT matches regions with similar internal geometry and relative position. This also explains how GROTIA behaves when one modality has more cell types than the other.

To further validate this behavior, we include an additional benchmark in which the RNA modality contains HeLa and HCT cancer cell lines, while the ATAC modality contains HeLa, HCT, and K562. This “2-versus-3 lineage” configuration allows us to assess whether GROTIA can correctly align the shared HeLa and HCT populations without spuriously matching the K562 population, which is present only in one modality.

We have described this setting in the Real World Datasets section as follows:

“In addition to the primary datasets described above, we evaluated GROTIA on an additional dataset with unbalanced cell populations to assess robustness, with full details and results provided in the Appendix: Additional Dataset.”

In the Appendix: Additional Dataset, we revised the description as follows:

“To evaluate robustness under unbalanced cell population settings, we considered a benchmark scenario in which one shared cell population is entirely missing from one modality. Specifically, we used a paired scRNA–scATAC dataset in which the scRNA-seq modality contains HeLa and HCT cell lines, while the scATAC-seq modality contains HeLa, HCT, and K562 cell lines. This setup creates an unbalanced integration scenario with mosaic structure and missing observations across modalities, mimicking realistic diagonal integration settings. Full details of the dataset could be found at \citet{samaran2024scconfluence}.

To evaluate robustness under unbalanced cell population settings, we considered a benchmark scenario in which one shared cell population is entirely missing from one modality. Specifically, we used a paired scRNA–scATAC dataset in which the scRNA-seq modality contains HeLa and HCT cell lines, while the scATAC-seq modality contains HeLa, HCT, and K562 cell lines. This setup creates an unbalanced integration

scenario with mosaic structure and missing observations across modalities, mimicking realistic diagonal integration settings. Full details of the dataset could be found at \citet{samaran2024scconfluence}.

Quantitative results on the cell-line dataset demonstrate that GROTIA is robust to variability in cell population proportions. In the unsupervised setting, GROTIA achieves the highest label-transfer accuracy (0.954), slightly outperforming the next best method, UnionCom (0.947). Its FOSCTTM score (0.318) is the second best and close to the best score achieved by UnionCom (0.287), indicating competitive alignment quality without supervision.

In the semi-supervised setting, GROTIA attains a label-transfer accuracy of 0.985, matching the best-performing methods (MMD-MA and scConfluence with prior, both 0.985). Its FOSCTTM score (0.323) is also comparable to other strong baselines (0.280 for UnionCom and 0.272 for scConfluence with prior). Overall, these results show that even when the two modalities differ in feature space and cell-type composition, GROTIA reliably recovers shared cell-state structure without requiring shared features, while appropriately handling cell populations present in only one modality."

—---------------------------------------

Comments:

- While the manuscript assesses alignment quality using Fraction of Samples Closer Than the True Match (FOSCTTM) and Label Transfer Accuracy (LTA), capturing local alignment and biological label concordance, these metrics do not directly evaluate preservation of global structure. Since GROTIA is designed to balance both global and local alignment, it would be helpful to include an explicit global alignment metric to confirm that this objective is being met. Some of the provided figures (e.g., Fig. 2c, right panel, and Fig. 3b after alignment) suggest global structure is preserved, but incorporating a dedicated metric or discussion would strengthen the evidence and provide a more complete evaluation of alignment quality.

Response:

We thank the reviewer for this comment. Both FOSCTTM and label transfer accuracy (LTA) are best viewed as local–global hybrid metrics, and they are widely used in the single-cell integration literature to evaluate complementary aspects of alignment quality.

FOSCTTM evaluates local neighborhood correctness by comparing, for each cell, the distance to its true cross-modal match against other samples, and reports the average score across all cells, providing a dataset-level summary of local alignment fidelity. LTA is computed via local kNN voting but assesses global biological consistency by measuring cross-modal agreement of cell-type identities across the entire dataset. Developing more explicit and comprehensive global geometric alignment metrics is an important direction for future work.

—---------------------------------------

Comments:

- Likewise, the manuscript states that GROTIA employs orthogonality constraints within the Reproducing Kernel Hilbert Space (RKHS) to enhance interpretability and stability. The use of these constraints for interpretability is illustrated through feature importance analyses; however, there is no direct comparison showing that this approach yields improved interpretability relative to unimodal analyses. Additionally, the effect of orthogonality constraints on embedding stability is not clearly assessed. Providing empirical evidence that these constraints improve the consistency of the embeddings or the quality of feature discovery, particularly in relation to single-modality methods, would help confirm the added value of this design choice and support several of the broader claims made regarding marker gene discovery and cell population characterization.

Response:

We appreciate the reviewer's comment. In GROTIA, the orthogonality constraints imposed in the RKHS were previously described as improving interpretability; however, we agree that this wording may be misleading. We have therefore revised the manuscript to remove claims of improved interpretability and instead emphasize the role of these constraints in enhancing embedding stability.

Specifically, the RKHS orthogonality constraint helps prevent rank degeneracy, reduces redundancy between latent axes, and avoids collapsed embeddings arising from graph Laplacian regularization. The effects of this constraint are now explicitly analyzed in the Appendix: Effect of the RKHS Orthogonality Constraint. We have added the following context to the GROTIA Algorithm section:

"For details on the effect of this constraint, we refer the reader to the Appendix: Effect of the RKHS Orthogonality Constraint."

—---------------------------------------

Comments:

- The decision to exclude scConfluence from the scGEM and SNARE evaluations due to prior dimensionality reduction could be better substantiated. Since raw data for both datasets are publicly available (e.g., SNARE-seq on GEO, scGEM on SRA), it would be helpful to explain why reprocessing the data was not feasible or appropriate.

Response:

We thank the reviewer for pointing this out and for noting that raw scGEM and SNARE-seq data are publicly available. We agree that, in principle, scConfluence can be run in a "prior-informed" mode when one can meaningfully define a shared gene space (as done in the original paper via gene activity matrices for ATAC). However, in the specific case of scGEM, SNARE-seq, and our simulated datasets, we were not able to construct a sensible shared feature set that would make this configuration well defined and comparable across methods.

In line with the scConfluence paper, we attempted to follow their strategy and derive gene-level activity scores for ATAC and then subset both modalities to a common set of genes. However, the original article uses a heterogeneous collection of dataset-specific pipelines and tools for this step (Maestro, Signac, Cicero, etc.), and does not provide an explicit, unified recipe or code for constructing gene activities and shared genes for scGEM, SNARE-seq, or for the kinds of simulated data we consider. When we tried to reproduce a similar procedure for these datasets, we did not obtain a non-trivial, biologically meaningful overlap of genes that we were confident using as a prior. At the same time, choosing one particular peak-to-gene mapping tool and parameterization only for scConfluence (and only for these datasets) would introduce an extra layer of method- and dataset-specific engineering that no other baseline benefits from, making the comparison less fair and less reproducible.

We therefore adopted the following consistent strategy in our benchmarks: (i) for datasets where a natural shared feature space exists and the necessary metadata for gene-level alignment are already available (e.g. the PBMC1 and PBMC2 datasets used in the paper), we run scConfluence in its prior-informed mode and also report its diagonal (no-prior) variant; (ii) for scGEM, SNARE-seq, and the remaining simulated datasets, where a robust, interpretable shared gene set cannot be defined in a principled way, we only include the diagonal configuration of scConfluence, which uses the same standardized inputs as the other methods and does not rely on an additional peak-to-gene prior.

Finally, we note that all other benchmarked methods in our study are used in a fully unsupervised, no-prior setting. Adding a feature-level prior is therefore not required for a fair comparison. We chose to report the prior-informed configuration of scConfluence only in those datasets where it is naturally and reproducibly defined, to illustrate that GROTIA remains competitive even when a competing method is allowed to leverage additional prior information, while keeping the main benchmark results strictly comparable across methods in a prior-free, unsupervised regime.

We have added the following explanation to the Baseline Settings section:

“For the four simulated datasets as well as the scGEM and SNARE-seq datasets, the construction of a cross-modality distance matrix is non-trivial. Specifically, the simulated datasets lack meaningful cross-modal feature definitions, and computing such distances for scGEM and SNARE-seq is not feasible.”

—--------------------------------------

Comments:

4b. Biological Interpretation
- The reasoning in the statement "Notably, GROTIA requires no a priori matching of features across modalities, so these dimension-specific drivers offer an unbiased method to uncover potential marker genes" is somewhat unclear. While the method's ability to operate without explicit feature matching is a strength, it would be helpful to clarify how this property directly leads to unbiased marker discovery. In particular, elaborating on how the dimension-specific drivers compare to features identified through unimodal or matched-feature approaches, would strengthen the interpretation.

Response:

We thank the reviewer for this helpful comment and agree that our original wording was not sufficiently precise. In particular, the term “unbiased” was potentially misleading. In the revised manuscript, we have removed this shorthand and replaced it with “data-driven,” by which we mean agnostic to pre-defined cross-modal feature matching or marker selection. Concretely, GROTIA is trained without constructing peak-to-gene mappings, without restricting to a hand-picked shared gene set, and without supplying curated marker lists. Integration is performed purely at the cell level based on within-modality structure. Once the joint latent space is learned, we compute dimension-specific drivers by scoring all features in each modality with respect to each latent dimension. The resulting rankings are therefore determined by the learned joint geometry of the data, rather than by an externally engineered shared feature space.

To address the comparison requested by the reviewer, we provide the following explanation:
- Unimodal marker discovery (e.g. RNA-only workflows) identifies features using a single modality and does not perform multi-omic integration, so its markers cannot reflect relationships that become apparent only when multiple modalities are considered jointly.
- Matched-feature methods typically require both modalities to be represented in a common feature space (for example, by computing gene-activity matrices for ATAC and subsetting to “shared genes”). In these approaches, marker discovery is restricted to that engineered shared subset and can be influenced by the particular peak-to-gene mapping strategy, tool choice, and preprocessing parameters.
- GROTIA’s dimension-specific drivers are computed after multi-omic alignment, over the full original feature set of each modality, and without relying on any specific cross-modal feature matching pipeline or prior marker set. This avoids constraining marker discovery to a particular constructed shared gene space and provides a data-driven way to rank features along each latent dimension.

Because unimodal and matched-feature methods operate in fundamentally different feature spaces (single-modality genes versus engineered shared gene sets), their marker lists are not directly comparable to GROTIA’s dimension-specific drivers in a one-to-one fashion. Instead, we validate GROTIA’s feature rankings by examining internal multi-omic consistency. In particular, as shown in Fig. 5, for representative latent dimensions a large fraction of the top ATAC peaks lie in the vicinity of the top RNA genes for the same dimension, and these peak–gene pairs tend to be positively correlated across cells. This supports our interpretation that GROTIA provides data-driven, axis-wise feature importance derived from the learned joint geometry, without relying on pre-defined cross-modal feature matches or prior marker selection.

—--------------------------------------

Comments:

- Several statements related to cell-type-specific gene expression, such as "LYZ, ZEB2, PLXDC2 are highly

expressed in monocytes…", would benefit from appropriate citations. This applies to other claims throughout the manuscript regarding gene specificity for particular lineages or subtypes.

Response:

We thank the reviewer for this helpful suggestion. In the revised manuscript, we have added citations to established marker-gene resources, primarily drawing from the Human Protein Atlas database.

—--------------------------------------

Comments:

4c. Clustering Evaluation

- The claim that GROTIA achieves "comparable or better performance" than Louvain clustering is not fully supported. While ARI/NMI scores of 0.75-0.8 indicate reasonable alignment with reference annotations, clarity on how ground truth (reference) labels were defined, whether Louvain resolution parameters were tuned, and which dataset(s) were used would strengthen this comparison. Additionally, specifying which co-clustering algorithm was used from the cited Python package, along with its parameter settings, would improve reproducibility and interpretability.

Response:

We thank the reviewer for these detailed comments and agree that our original description of the Louvain and co-clustering baselines was not sufficiently specific. We have clarified the following points in the revised manuscript.
First, the “reference” or “ground truth” labels used to compute ARI, NMI, and Purity correspond to the cell-type annotations provided by the original studies for each dataset, as described in the Data section. The PBMC-1 dataset is used as a representative example to illustrate this evaluation procedure.

Second, for the Louvain baseline, we used the standard Louvain implementation from the Python ecosystem applied to the same cell–cell graph used in our other analyses. The resolution parameter was tuned over the grid {0.1, 0.3, 0.4, 0.5, 0.6, 1.0, 1.5}, and the value that maximized the sum of ARI, NMI, and Purity with respect to the ground truth cell-type labels was selected for each dataset. In the revised manuscript, we explicitly specify which datasets the reported ARI/NMI values (0.75 - 0.8) correspond to and list the associated resolution choices. This clarification ensures that the comparison between GROTIA and the Louvain baseline reflects a clearly defined tuning protocol rather than a single untuned run.

We have added the following clarification to the section GROTIA enables identification of cellular subpopulations in the integrated space:

“We tuned the resolution parameter over the grid resolution ∈ {0.1, 0.3, 0.4, 0.5, 0.6, 1.0, 1.5} and selected the value that maximized the sum of ARI, NMI, and Purity with respect to the ground truth cell type labels on that dataset.”

Third, for the co-clustering baseline, we used the algorithm “co-clustering by alternated maximization of bipartite graph modularity” from the cited Python package. In this method, the only nontrivial hyperparameter is the number of clusters. As described in the manuscript, we determine the number of clusters for each dataset using an elbow criterion on the modularity curve, and then prune any resulting clusters with fewer than 20 cells as noise.

We have revised the section “Co-Clustering Using the Optimal Transport Plan” as follows:

“GROTIA also enables post-integration analysis to identify data-driven clusters. Specifically, an optimal transport (OT) plan is first computed to quantify the flow between two distinct sets of entities (e.g., cells in the RNA and ATAC spaces). We then apply a co-clustering algorithm \citep{role2019coclust} based on alternating maximization of bipartite graph modularity directly to the resulting transport matrix, thereby

simultaneously grouping row and column entities. By treating the OT plan as a bipartite graph, this co-clustering approach identifies latent structural patterns that minimize within-cluster transport costs while maximizing separation between clusters. Following co-clustering, clusters containing fewer than 20 cells are pruned as noise."

—--------------------------------------

Comments:

- The claims that GROTIA can uncover finer structures and novel cellular states, as well as identify refined subpopulations aligned with major cell types, are intriguing but would benefit from additional support. As currently presented, the results do not highlight specific novel cell populations or provide examples of newly discovered subclusters.

Response:

We thank the reviewer for this comment and agree that, as originally written, the phrases "uncover finer structures and novel cellular states" and "identify refined subpopulations aligned with major cell types" could be interpreted as stronger biological claims than what is directly supported by our current analyses.

Our intent was not to claim that GROTIA has already identified entirely new or previously unreported cell states in these datasets. Rather, we aimed to emphasize two more specific and empirically supported properties of the method. First, the integrated latent space produced by GROTIA enables finer, unsupervised refinement of known cell populations, as evidenced by improved NMI, ARI, and Purity scores relative to existing methods, indicating closer agreement between unsupervised clustering results and expert cell-type annotations. Second, GROTIA supports practical analysis settings in which cell-type annotations are available in only one modality. This is facilitated by the co-clustering procedure applied to the optimal transport plan, which jointly groups cells across modalities and allows cell population structure to be inferred in the unannotated modality through its alignment with the annotated one.

We have therefore removed the statement "By clustering cells within GROTIA's integrated representation, we can uncover finer structures and novel cellular states that may otherwise remain masked by legacy annotations."

In its place, we have added the following sentence to the section "GROTIA enables identification of cellular subpopulations in the integrated space":

"By clustering cells in GROTIA's integrated latent space, we recover cell population structure that closely matches expert annotations and enable inference of corresponding cell labels in a second modality when annotations are available in only one modality."

—--------------------------------------

Comments:

5. Writing, organization, tables, and figures, and minor notes

- There is a typo in the heading "GROTIA integrated simulated datasets in both semi and unsuperviseed setting" where unsuperviseed should be unsupervised.

Response:

We thank the reviewer for pointing out this typo. We have corrected "unsuperviseed" to "unsupervised" in the revised manuscript.

—--------------------------------------

Comments:

-- Under this heading, the section describing Figure 2a in paragraph two and paragraph three largely overlap.

Response:

We thank the reviewer for pointing this out. We have revised the manuscript to remove the redundancy between the second and third paragraphs under this heading and clarified the description of Fig. 2a to avoid overlapping content.

—---------------------------------------

Comments:

- The results and interpretation of Figure 2 panel b and c are not described to the reader. The same is true for Figure 3 panels b and c.

Response:

We thank the reviewer for this comment. We have added detailed descriptions and interpretations of Fig. 2 panels b and c, as well as Fig. 3 panels b and c, in the revised manuscript to clarify the results shown in these figures.

—---------------------------------------

Comments:

- In Figure 3, the method is still labeled as GROT instead of GROTIA; this should be updated for consistency.

Response:

We thank the reviewer for pointing this out. We have corrected the label in Figure 3 from GROT to GROTIA to ensure consistency throughout the manuscript.

—---------------------------------------

Comments:

- In Figure 3, the abbreviations Semi Acc and Un Acc are not defined in the legend and should be clearly explained.

Response:

Label Transfer Accuracy is shown with semi-supervised results on the x-axis (Semi Acc) and unsupervised results on the y-axis (Un Acc)

—---------------------------------------

Comments:

- In Figure 3, the visual layout in panel b differs between datasets and may be confusing for scGEM and SNARE-seq, the left and right columns represent cell types from each modality, whereas for PBMC, they reflect cell type and modality origin from a single, combined dataset. The PBMC-style presentation is more effective for visually assessing global alignment and should either be used consistently or more clearly explained.

Response:

We thank the reviewer for this helpful observation. We agree that the visual layouts in Fig. 3b differs across datasets. For the scGEM and SNARE-seq datasets, we intentionally retained the original visualization style used in the corresponding benchmark methods in order to maintain consistency with prior work and to facilitate direct comparison when readers refer to the original references.

—--------------------------------------

Comments:

- In Figure 3, legends are also missing descriptions of the color schemes used to denote modality.

Response:

We thank the reviewer for pointing this out. We have updated the legend of Figure 3 to explicitly describe the color scheme used to denote different modalities, ensuring that the figure is self-contained and easier to interpret.

—--------------------------------------

Comments:

- In panel c of both Figures 2 and 3, it should be specified whether the results correspond to semi-supervised or unsupervised alignment.

Response:

We thank the reviewer for this comment. We have clarified in the figure legends that the results shown in panel c of both Figures 2 and 3 correspond to the unsupervised alignment setting.

—--------------------------------------

Comments:

- In statements such as "Figure 4b presents UMAP visualizations of the top gene expression patterns for Dimensions 1 and 3", the wording could be clarified to avoid confusion. Specifically, it would help to state that gene expression patterns are overlaid on a UMAP projection of the scRNA-seq data, and that the genes visualized were selected based on their importance in Dimensions 1 and 3 of the RBF kernel embeddings (not UMAP axes).

Response:

We thank the reviewer for this helpful suggestion. We have revised the wording to clarify that gene expression patterns are overlaid on a UMAP projection of the scRNA-seq data, and that the genes shown were selected based on their importance in Dimensions 1 and 3 of the RBF kernel embeddings rather than the UMAP axes.

—--------------------------------------

Comments:

- In Figure 4 panel a, it appears that several genes from D1-4 have higher importance in D5. Is this due to scaling, or does it have some biological interpretation?

Response:

We thank the reviewer for this observation. This effect arises from the way Fig. 4a is constructed to provide a diagonal-style comparison across latent dimensions. Some genes therefore appear more than once across different dimensions, and higher importance values outside the highlighted (red-boxed) dimension correspond to the same genes selected within the box. This design choice was made to facilitate visual comparison across dimensions

—--------------------------------------

Comments:

- In Figure 4, panel c, the colorbar should be labeled.

Response:

We thank the reviewer for pointing this out. We have added an explicit label to the colorbar in Figure 4c in the revised manuscript.

—--------------------------------------

Comments:

- In Figure 5, panel a, only chromosome identifiers are shown, making the peak information incomplete and difficult to interpret. Including specific peak coordinates would improve clarity.

Response:

We thank the reviewer for this comment. Figure 5a is intended as an illustrative overview rather than a detailed genomic annotation, and therefore displays only chromosome-level identifiers to maintain visual clarity.

—--------------------------------------

Comments:

- In Figure 5d, it is not clear how accessibility is quantified for a specific gene, this should be described in the Methods section and reiterated in the results description.

Response:

We thank the reviewer for pointing this out. We clarify that panel 5d displays gene expression values, not chromatin accessibility. We have corrected the terminology in the figure.
—--------------------------------------

Comments:

- While the context makes it clear, explicitly noting that SPI1 is also known as PU.1 could improve clarity for readers less familiar with the nomenclature.

Response:

We thank the reviewer for this helpful suggestion. We have revised the manuscript to explicitly note that SPI1 is also known as PU.1, improving clarity for readers less familiar with the nomenclature.

—--------------------------------------

Comments:

- The explanation of the proposed regulatory relationship between CEBPB and KLF4 could be strengthened. The manuscript notes that both factors cooperate with PU.1, but no direct link between CEBPB and KLF4 is established, aside from their shared involvement in monocyte development and differentiation.

Response:

We thank the reviewer for raising this point. We clarify that our discussion does not intend to assert a direct regulatory relationship between CEBPB and KLF4. Instead, KLF4 is treated as the target transcription factor, and PU.1 (SPI1) and CEBPB are discussed as upstream factors that independently interact with or cooperate in regulatory programs relevant to monocyte differentiation. Therefore, establishing a direct interaction between CEBPB and KLF4 is not required for the interpretation presented. We have clarified this point in the revised manuscript to avoid any potential misunderstanding.

—---------------------------------------

Comments:

- The statement that "co-expression networks further link CLEC7A with an IRF8-centered module" would be more convincing with a supporting citation or additional methodological detail on how this link was established.

Response:

We thank the reviewer for this suggestion. We have added appropriate citations supporting the statement that co-expression networks link CLEC7A with an IRF8-centered module, and have clarified the relevant methodological details in the revised manuscript.

—---------------------------------------

Comments:

- The description of Figure 5c could be expanded. The current phrasing, "validated through literature. For instance, FOS are implicated as potential regulators of KLF4 in Dimension 1 and CEBPB of FCR1G in Dimension 2" would be better placed in the main text, supported by citations, and more clearly connected to the results.

Response:

We thank the reviewer for this comment. We have clarified the phrasing without expanding the analysis and ensured the text is consistent with the presented results.

—---------------------------------------

Comments:

- It would strengthen the interpretation if claims about the cell-type specificity of TF-target pairs were explicitly linked to the expression patterns shown in Figure 5, panel d.

Response:

We thank the reviewer for this comment. We clarify that our interpretation does not make any claims about cell-type specificity of the TF–target pairs. Rather, the analysis in Figure 5 focuses on illustrating dimension-specific associations identified by GROTIA, not on assigning TF–target specificity to particular cell types.

—---------------------------------------

Comments:

- Figure 5 panel d is missing a label on the color bar.

Response:

We thank the reviewer for pointing this out. We have added an explicit label to the colorbar in Figure 5 panel d in the revised manuscript.

—--------------------------------------

Comments:

- "gene" in "as potential regulators of Gene KFL4" in the legend of Figure 5 should not be capitalized.

Response:

We thank the reviewer for noting this. We have corrected the capitalization in the Figure 5 legend accordingly.

—--------------------------------------

Comments:

- The section identifier is missing from the statement "For further details, please refer to Section ."

Response:

We thank the reviewer for catching this oversight. We have added the correct section reference in the revised manuscript.

—--------------------------------------

Comments:

- Figure panel 6b is missing a label for RNA on the y-axis.

Response:

We thank the reviewer for pointing this out. We have added an explicit label to panel 6b in the revised manuscript.

—--------------------------------------

Comments:

- The referencing in a few instances could be strengthened for clarity and accuracy. For example, the statement "Lots of computational methods have recently been developed to integrate data across multiple modalities [4, 5]" cites only two methods, which may not sufficiently support the breadth implied. Either citing additional representative methods or rephrasing the sentence to more accurately reflect the scope would improve the credibility of the claim.

Response:

We thank the reviewer for this helpful suggestion. We have added additional representative citations to better reflect the breadth of recent multimodal integration methods and revised the phrasing accordingly to

improve clarity and accuracy.

—--------------------------------------

Comments:

- To further support the claim that "GROTIA delivers comparable or superior performance," the authors might consider including comparisons to other recent diagonal integration methods such as Pamona and the updated version of SCOT: SCOTv2.

Response:

We thank the reviewer for this helpful suggestion. We considered including SCOTv2 and other recent diagonal integration methods; however, we found inconsistent performance reports across datasets between SCOTv1 and SCOTv2. In particular, SCOTv2 performs better on highly disproportionate datasets, whereas SCOTv1 handles both balanced and unbalanced settings more reliably, albeit with a tradeoff. Because SCOTv1 provides a more stable baseline across all datasets used in our benchmark, we chose to include SCOTv1 for consistency and fairness. We have added a brief clarification in the manuscript to explain this choice.

****
Reviewers' Comments to Author:
****
Reviewer: 2
****

Comments:

This paper introduces a graph-regularized optimal transport framework, GROTIA, for aligning multi-omics datasets. It is a diagonal integration method capable of aligning single cells without requiring direct cell-cell correspondences. The interpretable embeddings produced by GROTIA are particularly impressive and broaden the applicability of diagonal integration approaches. Overall, the paper is clearly written and well-structured. I only have a few minor comments:

Response:

We thank the reviewer for the positive assessment of our work and for the thoughtful and constructive comments. We address each of the minor points raised below.

—--------------------------------------

Comments:

Kernel-based methods are typically limited in scalability since they require optimization over the entire kernel matrix. How do the authors address this issue? Can the authors also provide more details on the computational efficiency of the model?

Response:

In its current form, GROTIA is a full-kernel method, and its time and memory complexity scale quadratically with the number of cells, as is typical for kernel and full-matrix OT approaches. Let n_x and n_y be the numbers of cells in X and Y, d_x and d_y their feature dimensions, k the latent dimension, S the number of Sinkhorn iterations, and I the number of outer optimization iterations. Computing the Gaussian kernels K_X and K_Y costs $O(n_x^2 d_x + n_y^2 d_y)$ time and requires $O(n_x^2 + n_y^2)$ memory to store the kernel matrices. In each gradient step, forming the latent embeddings costs $O(n_x^2$

k + n_y^2 k); computing the pairwise cost matrix and running S Sinkhorn iterations on the transport plan T (of size n_x × n_y) costs O(n_x n_y (k + S)) time and O(n_x n_y) memory. In the balanced case n_x ≈ n_y ≈ n, this yields an overall complexity of O(I · n^2 (k + S)) time and O(n^2) memory, dominated by storing the kernel matrices and the OT plan.

To give a concrete picture of computational efficiency, we have added a runtime / memory table (Appendix, Table A.1) for the PBMC-1 dataset (9,378 cells). On this dataset, GROTIA runs in 15 minutes with a peak GPU memory allocation of 9.8 GB and peak CPU resident memory of 5.2 GB. This is substantially faster than other full-kernel optimal transport baselines such as UnionCom (65 minutes), SCOT (185 minutes), and MMD-MA (470 minutes), while having a total memory footprint that is smaller than SCOT, Unioncom, MMD-MA and comparable to the other batched methods. Compared to a mini-batch / non-kernel method such as UniPort and Scconfluence, GROTIA's runtime is very similar (15 vs 17 minutes and 21 minutes), with GPU memory moderately higher total memory usage (15 GB vs 9.2 GB and 5.3 GB). Thus, within the class of full-kernel diagonal integration methods, GROTIA is among the most computationally efficient in our experiments.

We agree that truly large-scale single-cell datasets will require additional approximations. Extending GROTIA with kernel approximations (e.g. Nyström, random features) or mini-batch / sparse variants to reduce the memory cost is a natural next step, and we now explicitly mention this as an important direction for future work in the Discussion section.

—--------------------------------------

Comments:

The optimization procedure for Equation (9) is not sufficiently clear. A more detailed algorithmic description can be very helpful.

Response:

We thank the reviewer for this suggestion. In the revised manuscript, we have added an "Optimization Details" section in the Appendix that explains the optimization procedure and parameter settings.

We have also referenced this section in the main manuscript under Training Details with the statement:

"A more detailed explanation is provided in the Appendix: Optimization Details."

—--------------------------------------

Comments:

Can the interpretable embeddings introduced here be generalized to other kernel-based methods, such as MMD-MA?

Response:

Yes. Since both GROTIA and MMD-MA learn embeddings via projections in an RKHS defined by kernel matrices, similar downstream analyses and utilities can be incorporated, making the approach broadly applicable to kernel-based alignment methods.

—--------------------------------------

Comments:

A more comprehensive robustness analysis with respect to parameter choices can be helpful

Response:

We thank the reviewer for this suggestion. In the revised manuscript we added a dedicated Appendix section on hyperparameter sensitivity, where we now report four complementary experiments:

Latent dimension (p). For each dataset (SNARE, scGEM, PBMC-1, PBMC-2) we sweep $p \in \{5,8,16,32\}$ and report Label Transfer Accuracy (LTA). Dimensions p=5 or p=8 consistently give the best or near-best LTA, with only modest degradation at higher dimensions, so we restrict p to 5 or 8 in all main experiments.

Regularization strengths (lambda_topo and lambda_reg). We perform a grid search over lambda_topo and lambda_reg and visualize LTA as a function of these two parameters. The resulting map shows a broad plateau with LTA > 0.9 across several orders of magnitude in both penalties, except for a small corner with extremely strong graph regularization and very weak orthogonality.

Robustness aggregated over latent dimensions. We summarize the effect of p by plotting the distribution of LTA across all combinations of (lambda_topo, lambda_reg, reach) separately for p=5 and p=8. The violin plots are tightly concentrated near high LTA for both values, with only rare low-performing outliers.

Sinkhorn interaction scale (reach). We perform an analogous aggregation over reach ∈ {0.1, 1.0, 5.0}, pooling across all other hyperparameters. The LTA distributions overlap closely and remain high for all three values, indicating that GROTIA is insensitive to the precise choice of the Sinkhorn scale.

These four analyses are summarized in Appendix: Hyperparameter Robustness Analysis.

Editorial Manager®

Cookie Preference Center

We use cookies which are necessary to make our site work. We may also use additional cookies to analyze, improve and personalize our content and your digital experience. For more information, see our Cookie Policy.

You may choose not to allow some types of cookies. However, blocking some types may impact your experience of our site and the services we are able to offer. See the different category headings below to find out more or change your settings.

Allow all

Manage Consent Preferences

Strictly Necessary Cookies

Always active

These cookies are necessary for the website to function and cannot be switched off in our systems. They are usually only set in response to actions made by you which amount to a request for services, such as setting your privacy preferences, logging in or filling in forms. You can set your browser to block or alert you about these cookies, but some parts of the site will not then work.

Functional Cookies

These cookies enable the website to provide enhanced functionality and personalisation. They may be set by us or by third party providers whose services we have added to our pages. If you do not allow these cookies then some or all of these services may not function properly.

Performance Cookies

These cookies allow us to count visits and traffic sources so we can measure and improve the performance of our site. They help us to know which pages are the most and least popular and see how visitors move around the site.

Targeting Cookies

These cookies may be set through our site by our advertising partners. They may be used by those companies to build a profile of your interests and show you relevant adverts on other sites. If you do not allow these cookies, you will experience less targeted advertising.

Cookie List

Clear

Apply Cancel

Consent Leg.Interest

Confirm my choices

Powered by onetrust
