# Supplementary material for: An interpretable Graph-Regularized Optimal Transport Framework for Diagonal Single-Cell Integrative Analysis
Source: Gigascience. 2026 Feb 9;15:giag012. doi: 10.1093/gigascience/giag012 (PMC12970605; doi:10.1093/gigascience/giag012)
Supplement: giag012_Reviewer_1_Report_original_submission — Reviewer 1 -- 7/142025 [file giag012_reviewer_1_report_original_submission.pdf]

Reviewer Report

Title: An Interpretable Graph-Regularized Optimal Transport Framework for Diagonal Single-Cell Integrative Analysis

Version: Original Submission **Date:** 7/14/2025

Reviewer name: Reviewer 1

Reviewer Comments to Author:

The manuscript presents a well-motivated and technically elegant approach to diagonal single-cell data integration, combining optimal transport with graph-based regularization to achieve a balance between global and local structure alignment. The method addresses an important challenge in single-cell data integration, where existing approaches still leave room for improvement. Its embedding design offers the potential for interpretable feature-level insights, a particularly desirable quality in single-cell multi-omics integration where biological interpretability is especially important.That said, the manuscript would be substantially strengthened by deeper validation and a clearer demonstration of reproducibility. Some claims would benefit from stronger empirical support in the presented results, and a more thorough evaluation of the method's added value relative to unimodal alternatives, particularly in the context of marker gene discovery and the identification of cell types or subpopulations, could further enhance the manuscript. Additionally, the impact of key parameter choices, such as kernel bandwidth selection, the number of nearest neighbors (k), and sensitivity to hyperparameters (Î», Ï[illegible]), should be more fully explored, reported, or justified. Reproducibility could be improved by providing scripts and a computational environment or container to replicate all analyses and figures presented in the manuscript. Usability would also be improved by providing the method as an installable Python package, rather than limiting implementation to a Jupyter Notebook.Overall, the manuscript introduces a compelling methodological framework with meaningful potential for applications in single-cell integration. The suggestions that follow are intended to help the authors strengthen their contribution in alignment with GigaScience's emphasis on openness, reproducibility, and FAIR principles. I hope these suggestions will help strengthen the support for the authors' conclusions, clarify the reasoning behind key arguments, and improve the clarity and interpretability of the figures and descriptions.1. ReproducibilityReproducibility is impeded by the absence of clearly organized scripts or workflow files to regenerate the results, figures, and tables presented in the manuscript. While some outputs are shown or alluded to in the Jupyter Notebook found in the linked GitHub repository, they are not clearly cross-referenced with the paper's results, making it difficult to confirm how specific figures or tables were produced. Furthermore, no computational environment specification is provided, which makes replication with confidence impossible. Certain aspects of the manuscript fall short of best practices for transparent and reproducible research. Analysis scripts are incomplete or undocumented, and key portions of the software pipeline are either insufficiently described or lack proper attribution. These limitations hinder reproducibility and reduce reusability. Figures would also benefit from clearer annotation. Collectively, these shortcomings detract from alignment with the FAIR principles emphasized by GigaScience. Reproducibility would be significantly improved by packaging the software,

versioning the code, defining and documenting the computational environment, and depositing all components of the analysis pipeline, including preprocessing scripts, evaluation code, and figure generation, in a publicly accessible repository.Additionally, while it is generally clear how the data were collected and curated, the rationale for using preprocessed datasets, particularly those sourced from external repositories, could be more clearly explained. The data are shared via a Google Drive link provided in the GitHub repository, which is convenient, though it may benefit from a more transparent and persistent form of distribution. The manuscript states that "All data used in this manuscript is publicly available and can be found at Liu et al. [11], Cheow et al. [16], Demetci et al. [12], Chen et al. [17], Cao et al. [14], and Samaran et al. [13].", but it appears that preprocessed versions of these datasets were used, rather than the original raw data. Clarifying this point would help improve transparency and reproducibility.The manuscript also describes custom preprocessing procedures for scRNA-seq and scATAC-seq data, including PCA, TF-IDF normalization, and gene filtering, that appear inconsistent with the properties of the datasets used. Without access to preprocessing scripts or further clarification, it is unclear whether these procedures were performed as described. Clarifying these discrepancies would strengthen transparency and ensure fair benchmarking comparisons. In addition, to improve transparency and reproducibility, it would be helpful to provide the scripts or commands used to run these baseline methods, along with the evaluation code for computing the reported metrics.Finally, several methodological details underlying downstream analyses are insufficiently described to allow confident reproduction or interpretation. For instance, it is unclear which dataset was used to obtain the results in "GROTIA Reveals Gene-Specific Contributions and Key Biological Processes in the RNA Embedding" and Figure 4 and 5. Additionally, the motif discovery step using GimmeMotifs should be expanded, since it is currently not entirely clear how motifs were matched to known transcription factors, and the process described in the text does not fully align with what is shown in Figure 5A. Clarifying these points would help improve the reproducibility and interpretability of the manuscript's key biological findings.2. UsabilityThe code repository is easy to find on GitHub, available under the MIT license, following the link presented in the manuscript. However, the currently presented implementation is provided as a Jupyter Notebook that demonstrates the basic usage of the method, and technically allows users to replicate the process using their own data. Usability is currently limited by sparse documentation and could benefit from guidance on input requirements, parameter configuration, and expected output formats. To improve usability, the authors should supplement the notebook with detailed explanations, comments, and a README or user guide that explains how to prepare input data, adjust key parameters, interpret outputs, and run the method on other datasets. Wrapping core functionality into a small, importable Python module or script would further reduce friction for adoption and integration into pipelines.3. Attribution and Software TransparencyThe GitHub repository includes an evals.py script originally authored by the creators of SCOT (Pinar Demetci, Rebecca Santorella, and Ritambhara Singh), with attribution preserved within the file. However, the manuscript itself does not mention that components of the evaluation pipeline were adapted from this prior work. Given that this script supports benchmarking comparisons central to the paper's conclusions, explicit acknowledgment in the text would improve transparency and ensure appropriate credit is given.4. Support for Claims and Biological InterpretationSeveral key claims would benefit from additional evidence or clarification. I divide this into subsections "4a. Methodological Claims," "4b. Biological Interpretation," and "4c. Clustering Evaluation" for extra clarity and readability.4a.

Methodological Claims- The claim "we selected the latent dimension to be either 5 or 8 and observed that GROTIA remained robust to this choice" is not substantiated by any reported results or sensitivity analysis.- The claim that GROTIA is computationally efficient would be more compelling if runtime comparisons included system specifications, analysis on larger (potentially synthetic) datasets, memory usage, and scalability assessments across CPU and GPU modes. Directly referencing Table A1 for the current runtime evaluation and adding the additional metrics mentioned above would provide a more comprehensive evaluation.- The manuscript asserts "Notably, unlike methods that require shared features across modalities, GROTIA only assumes that cells (rather than individual genes or peaks) follow a similar distribution if they belong to the same type or lineageâ€”thus broadening its applicability to complex datasets." This claim would be more convincing if supported by analyses on more complex datasets, such as those with technical variability across origin sites, donors, or protocols; mosaic structures with missing observations; nested batch effects; or significant differences in data quality. Additionally, this statement may appear in tension with the claim that GROTIA depends on the presence of a shared underlying biology, which would not hold in many complex or heterogeneous settings. Clarifying how "complexity" is defined in the context of GROTIA's assumptions, and empirically substantiating the method's generalizability to such settings would improve both the precision and credibility of this claim.- While the manuscript assesses alignment quality using Fraction of Samples Closer Than the True Match (FOSCTTM) and Label Transfer Accuracy (LTA), capturing local alignment and biological label concordance, these metrics do not directly evaluate preservation of global structure. Since GROTIA is designed to balance both global and local alignment, it would be helpful to include an explicit global alignment metric to confirm that this objective is being met. Some of the provided figures (e.g., Fig. 2c, right panel, and Fig. 3b after alignment) suggest global structure is preserved, but incorporating a dedicated metric or discussion would strengthen the evidence and provide a more complete evaluation of alignment quality.- Likewise, the manuscript states that GROTIA employs orthogonality constraints within the Reproducing Kernel Hilbert Space (RKHS) to enhance interpretability and stability. The use of these constraints for interpretability is illustrated through feature importance analyses; however, there is no direct comparison showing that this approach yields improved interpretability relative to unimodal analyses. Additionally, the effect of orthogonality constraints on embedding stability is not clearly assessed. Providing empirical evidence that these constraints improve the consistency of the embeddings or the quality of feature discovery, particularly in relation to single-modality methods, would help confirm the added value of this design choice and support several of the broader claims made regarding marker gene discovery and cell population characterization.- The decision to exclude scConfluence from the scGEM and SNARE evaluations due to prior dimensionality reduction could be better substantiated. Since raw data for both datasets are publicly available (e.g., SNARE-seq on GEO, scGEM on SRA), it would be helpful to explain why reprocessing the data was not feasible or appropriate.4b. Biological Interpretation- The reasoning in the statement "Notably, GROTIA requires no a priori matching of features across modalities, so these dimension-specific drivers offer an unbiased method to uncover potential marker genes" is somewhat unclear. While the method's ability to operate without explicit feature matching is a strength, it would be helpful to clarify how this property directly leads to unbiased marker discovery. In particular, elaborating on how the dimension-specific drivers compare to features identified through unimodal or matched-feature approaches, would strengthen the interpretation.- Several statements related to cell-

type-specific gene expression, such as "LYZ, ZEB2, PLXDC2 are highly expressed in monocytes…", would benefit from appropriate citations. This applies to other claims throughout the manuscript regarding gene specificity for particular lineages or subtypes.4c. Clustering Evaluation- The claim that GROTIA achieves "comparable or better performance" than Louvain clustering is not fully supported. While ARI/NMI scores of 0.75-0.8 indicate reasonable alignment with reference annotations, clarity on how ground truth (reference) labels were defined, whether Louvain resolution parameters were tuned, and which dataset(s) were used would strengthen this comparison. Additionally, specifying which co-clustering algorithm was used from the cited Python package, along with its parameter settings, would improve reproducibility and interpretability.- The claims that GROTIA can uncover finer structures and novel cellular states, as well as identify refined subpopulations aligned with major cell types, are intriguing but would benefit from additional support. As currently presented, the results do not highlight specific novel cell populations or provide examples of newly discovered subclusters.5. Writing, organization, tables, and figures, and minor notes- There is a typo in the heading "GROTIA integrated simulated datasets in both semi and unsuperviseed setting" where unsuperviseed should be unsupervised.- Under this heading, the section describing Figure 2a in paragraph two and paragraph three largely overlap.- The results and interpretation of Figure 2 panel b and c are not described to the reader. The same is true for Figure 3 panels b and c.- In Figure 3, the method is still labeled as GROT instead of GROTIA; this should be updated for consistency.- In Figure 3, the abbreviations Semi Acc and Un Acc are not defined in the legend and should be clearly explained.- In Figure 3, the visual layout in panel b differs between datasets and may be confusing for scGEM and SNARE-seq, the left and right columns represent cell types from each modality, whereas for PBMC, they reflect cell type and modality origin from a single, combined dataset. The PBMC-style presentation is more effective for visually assessing global alignment and should either be used consistently or more clearly explained.- In Figure 3, legends are also missing descriptions of the color schemes used to denote modality.- In panel c of both Figures 2 and 3, it should be specified whether the results correspond to semi-supervised or unsupervised alignment.- In statements such as "Figure 4b presents UMAP visualizations of the top gene expression patterns for Dimensions 1 and 3", the wording could be clarified to avoid confusion. Specifically, it would help to state that gene expression patterns are overlaid on a UMAP projection of the scRNA-seq data, and that the genes visualized were selected based on their importance in Dimensions 1 and 3 of the RBF kernel embeddings (not UMAP axes).- In Figure 4 panel a, it appears that several genes from D1-4 have higher importance in D5. Is this due to scaling, or does it have some biological interpretation?- In Figure 4, panel c, the colorbar should be labeled.- In Figure 5, panel a, only chromosome identifiers are shown, making the peak information incomplete and difficult to interpret. Including specific peak coordinates would improve clarity.- In Figure 5d, it is not clear how accessibility is quantified for a specific gene, this should be described in the Methods section and reiterated in the results description.- While the context makes it clear, explicitly noting that SPI1 is also known as PU.1 could improve clarity for readers less familiar with the nomenclature.- The explanation of the proposed regulatory relationship between CEBPB and KLF4 could be strengthened. The manuscript notes that both factors cooperate with PU.1, but no direct link between CEBPB and KLF4 is established, aside from their shared involvement in monocyte development and differentiation.- The statement that "co-expression networks further link CLEC7A with an IRF8-centered module" would be more convincing with a supporting citation or additional methodological detail on how this link was established.- The

description of Figure 5c could be expanded. The current phrasing, "validated through literature. For instance, FOS are implicated as potential regulators of KLF4 in Dimension 1 and CEBPB of FCR1G in Dimension 2" would be better placed in the main text, supported by citations, and more clearly connected to the results.- It would strengthen the interpretation if claims about the cell-type specificity of TF-target pairs were explicitly linked to the expression patterns shown in Figure 5, panel d.- Figure 5 panel d is missing a label on the color bar.- "gene" in "as potential regulators of Gene KFL4" in the legend of Figure 5 should not be capitalized.- The section identifier is missing from the statement "For further details, please refer to Section ."- Figure panel 6b is missing a label for RNA on the y-axis.- The referencing in a few instances could be strengthened for clarity and accuracy. For example, the statement "Lots of computational methods have recently been developed to integrate data across multiple modalities [4, 5]" cites only two methods, which may not sufficiently support the breadth implied. Either citing additional representative methods or rephrasing the sentence to more accurately reflect the scope would improve the credibility of the claim.- To further support the claim that "GROTIA delivers comparable or superior performance," the authors might consider including comparisons to other recent diagonal integration methods such as Pamona and the updated version of SCOT: SCOTv2.

Level of Interest

Please indicate how interesting you found the manuscript: Choose an item.

Quality of Written English

Please indicate the quality of language in the manuscript: Choose an item.

Declaration of Competing Interests

Please complete a declaration of competing interests, considering the following questions:

- Have you in the past five years received reimbursements, fees, funding, or salary from an organisation that may in any way gain or lose financially from the publication of this manuscript, either now or in the future?
- Do you hold any stocks or shares in an organisation that may in any way gain or lose financially from the publication of this manuscript, either now or in the future?
- Do you hold or are you currently applying for any patents relating to the content of the manuscript?
- Have you received reimbursements, fees, funding, or salary from an organization that holds or has applied for patents relating to the content of the manuscript?
- Do you have any other financial competing interests?
- Do you have any non-financial competing interests in relation to this paper?

If you can answer no to all of the above, write 'I declare that I have no competing interests' below. If your reply is yes to any, please give details below.

I declare that I have no competing interests.

I agree to the open peer review policy of the journal. I understand that my name will be included on my report to the authors and, if the manuscript is accepted for publication, my named report including any attachments I upload will be posted on the website along with the authors' responses. I agree for my report to be made available under an Open Access Creative Commons CC-BY license (http://creativecommons.org/licenses/by/4.0/). I understand that any comments which I do not wish to be included in my named report can be included as confidential comments to the editors, which will not be published.

Choose an item.

To further support our reviewers, we have joined with Publons, where you can gain additional credit to further highlight your hard work (see: https://publons.com/journal/530/gigascience). On publication of this paper, your review will be automatically added to Publons, you can then choose whether or not to claim your Publons credit. I understand this statement.

Yes Choose an item.
